# Homologues of epigenetic pyrimidines: 5-alkyl-, 5-hydroxyalkyl and 5-acyluracil and -cytosine nucleotides: synthesis, enzymatic incorporation into DNA and effect on transcription with bacterial RNA polymerase†

**DOI:** 10.1039/d2cb00133k

**Published:** 2022-06-30

**Authors:** Filip Gracias, Olatz Ruiz-Larrabeiti, Viola Vaňková Hausnerová, Radek Pohl, Blanka Klepetářová, Veronika Sýkorová, Libor Krásný, Michal Hocek

**Affiliations:** Institute of Organic Chemistry and Biochemistry, Czech Academy of Sciences, Flemingovo nam. 2 CZ-16000 Prague 6 Czech Republic hocek@uochb.cas.cz; Lab. of Microbial Genetics and Gene Expression, Institute of Microbiology, Czech Academy of Sciences, Vídeňská 1083 CZ-14220 Prague 4 Czech Republic krasny@biomed.cas.cz; Department of Organic Chemistry, Faculty of Science, Charles University, Hlavova 8 CZ-12843 Prague 2 Czech Republic

## Abstract

Homologues of natural epigenetic pyrimidine nucleosides and nucleotides were designed and synthesized. They included 5-ethyl-, 5-propyl-, 5-(1-hydroxyethyl)-, 5-(1-hydroxypropyl)- and 5-acetyl- and 5-propionylcytosine and -uracil 2′-deoxyribonucleosides and their corresponding 5′-*O*-triphosphates (dN^X^TPs). The epimers of 5-(1-hydroxyethyl)- and 5-(1-hydroxypropyl)pyrimidine nucleosides were separated and their absolute configuration was determined by a combination of X-ray and NMR analysis. The modified dN^X^TPs were used as substrates for PCR synthesis of modified DNA templates used for the study of transcription with bacterial RNA polymerase. Fundamental differences in transcription efficiency were observed, depending on the various modifications. The most notable effects included pronounced stimulation of transcription from 5-ethyluracil-bearing templates (200% transcription yield compared to natural thymine) and an enhancing effect of 5-acetylcytosine *versus* inhibiting effect of 5-acetyluracil. In summary, these results reveal that RNA polymerase copes with dramatically altered DNA structure and suggest that these nucleobases could potentially play roles as artificial epigenetic DNA nucleobases.

## Abstract 2

Nucleotides derived from homologues of epigenetic pyrimidine bases were prepared and used for polymerase synthesis of modified DNA templates. Interesting effects of the substituents on PCR and transcription have been observed.

## Graphical abstract

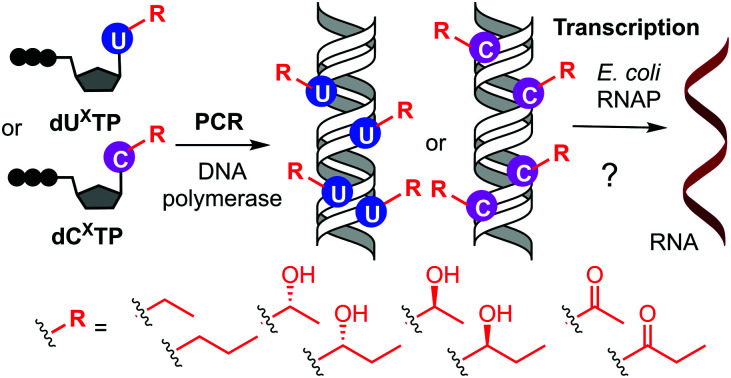

## Introduction

Epigenetic modifications of histones and DNA are important regulators of gene expression.^1–3^ In eukaryotic genomic DNA, the major epigenetic modification is 5-methylcytosine (5mC),^4^ which is formed through cytosine methylation by DNA methyltransferases^5^ and downregulates transcription when present at high levels. The oxidized derivatives,^6^ 5-hydroxymethylcytosine (5hmC),^7,8^ 5-formylcytosine (5fC)^9^ and 5-carboxycytosine (5caC),^10^ are rarer modifications formed through oxidation of 5mC by ten-eleven translocation (TET) enzymes.^11–13^ They are intermediates in an active demethylation process^14,15^ but also stable epigenetic marks^16,17^ with their own role in regulation of gene expression.^18–20^ 5-Hydroxymethyluracil (5hmU) is another rare natural DNA modification present in human stem cells,^21^ cancer cells,^22^ protozoan parasites^23^ and the genomes of certain bacteriophages, where 5hmU almost completely replaces thymine;^24,25^ yet its biological role is not fully understood.^26^ Another modification, 5-formyluracil (5fU), can be formed in DNA as a product of oxidative damage of thymine and is known to cause mutations due to base-pairing with both A and G.^27,28^ In our previous systematic study of the influence of non-natural and natural modifications in DNA on transcription with *Escherichia coli* RNA polymerase (RNAP), we found that some non-natural nucleobase modifications^29^ can be tolerated by RNAP and, surprisingly, the presence of 5hmU in the Pveg promoter significantly increased the transcription efficiency.^30^ Later on, we developed transcription switches based on photocaging and the release of 5hmU or 5hmC in DNA.^31,32^

Furthermore, there are even some examples of very rare natural pyrimidine DNA nucleobases bearing even more bulky modifications, *e.g.* glycine, 2-aminoethyl^33^ and several types of conjugates of 5hmU or 5hmC with glucose,^34–36^ amino acids, amines *etc.*^33^ The role of these modifications is either unknown or elusive. Recently, the K. Islam group has published^37^ an intriguing work showing that the TET2 enzyme can even oxidize non-natural 5-ethylC to 5-(1-hydroxyethyl)cytosine, which can be further chemically oxidized to 5-acetylcytosine or enzymatically glucosylated.^37^ These recent pioneering works prompted us to design and synthesize several more complex homologues of epigenetic pyrimidine nucleotides and study their enzymatic incorporation into DNA and their influence on transcription.

## Results and discussion

The target homologues of epigenetic pyrimidine nucleotides were derived from 5-ethyl- and 5-propyluracil and -cytosine and included their oxidized congeners, *i.e.* 1-hydroxyalkyl and 1-oxoalkyl derivatives. For comparison, we also included natural 5-formylpyrimidines^38^ as well as previously studied 5-vinyl- and 5-ethynylpyrimidines.^29,39,40^ These modifications were attached to the 5-position of 2′-deoxyuridine (dU) and 2′-deoxycytidine (dC).

**Scheme 1:**
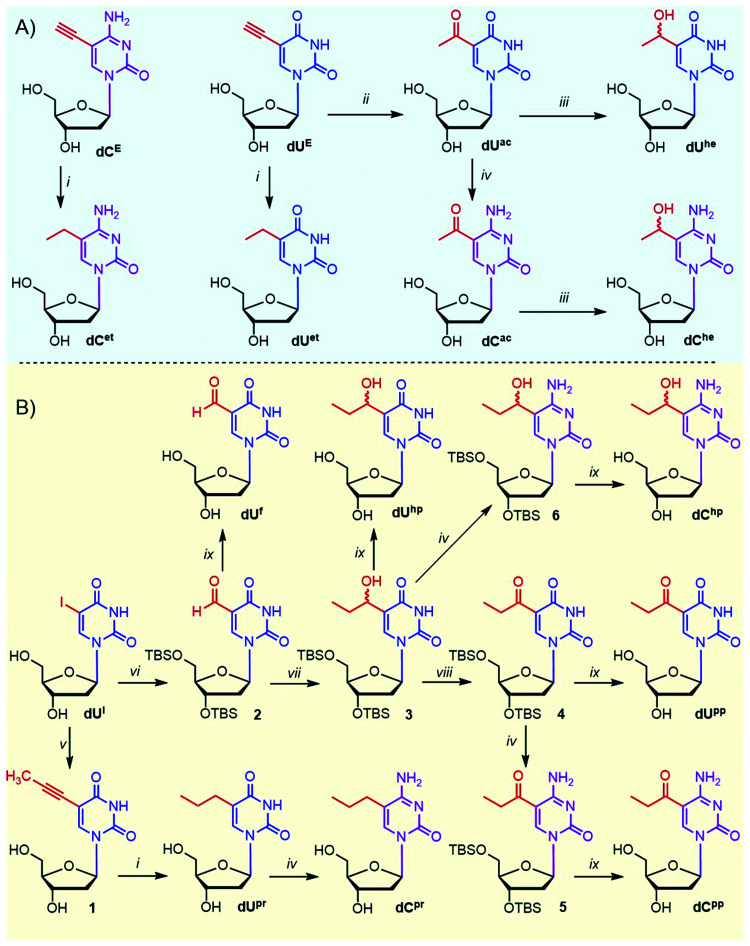
Reaction overview of nucleoside synthesis. Conditions: (i) 10% Pd/C, H_2_, and MeOH, at 23 °C, for 40 h; (ii) H_2_SO_4_, MeOH, and H_2_O, at 75 °C, for 3 h; (iii) NaBH_4_, CeCl_3_·7H_2_O, and MeOH, at 23 °C, for 2 h; (iv) PyAOP, DBU, NH_4_OH, and DMF, at 23 °C, for 2 h; (v) propyne, Pd(PPh_3_)_4_, CuI, Et_3_N and DMF, at 23 °C, for 2 h; (vi) TBSCl, imidazole, and DMF, at 23 °C, for 18 h, then Pd(PPh_3_)_4_, CO, Bu_3_SnH, and toluene, at 60 °C, for 18 h; (vii) EtMgBr, THF, at −78 °C, for 4 h; (viii) DMP, DCM, at 23 °C, for 3 h; (ix) Et_3_N*3HF, THF, at 23 °C, for 18 h.

Although the synthesis of several ethyl-based pyrimidine nucleosides is known,^41–43^ we prepared some of them in a different and more efficient way (Scheme 1A). Catalytic hydrogenation of 5-ethynylpyrimidine nucleosides dU^E^ (ref. 44) and dC^E^ gave ethyl derivatives dU^et^ and dC^et^ in 84 and 60% yields, respectively. Acid-catalyzed hydration of a terminal triple bond of 5-ethynyl-2′-deoxyuridine dU^E^ with dilute sulfuric acid in methanol gave acetyl derivative dU^ac^ (69%)^45^ that was the key intermediate for the synthesis of other required derivatives. The Luche reduction^46^ of dU^ac^ with NaBH_4_ and CeCl_3_ afforded 5-(1-hydroxyethyl)uracil nucleoside dU^he^ in a 49% yield (34% overall from dU^E^). Amination^47^ of dU^ac^ at position 4 with (7-azabenzotriazol-1-yloxy)trispyrrolidinophosphonium hexafluorophosphate (PyAOP) and NH_4_OH in the presence of DBU gave acetylcytosine nucleotide dC^ac^ (67% yield) that was reduced to dC^he^ (51% yield) using the Luche reduction (overall yield 24% from dU^E^).

The propyl-based nucleoside series was synthesized from 5-iodo-2′-deoxyuridine (dU^I^) in two different pathways (Scheme 1B). The first pathway consisted of the Sonogashira reaction of dU^I^ with generated propyne gas to produce 5-(prop-1-ynyl)-dU (1),^48^ which was hydrogenated to propyl-dU (dU^pr^),^49^ which was further transformed to cytidine derivative dC^pr^ through the above-mentioned amination. The main intermediate of the second pathway was TBS-protected 5-(1-hydroxypropyl)-dU (3) synthesized from dU^I^ by TBS protection, Pd-catalyzed carbonylation yielding the protected formyl-dU (2),^50^ which was further reacted with EtMgBr. Hydroxypropylpyrimidine intermediate 3 was obtained in an overall 51% yield and was further oxidized by Dess–Martin periodate and either deprotected or aminated and deprotected to give 5-(propionyl)-dU and -dC nucleosides (dU^pp^ and dC^pp^). The same intermediate 3 was also directly deprotected or aminated and deprotected to give 5-(1-hydroxypropyl)-dU and -dC (dU^hp^ and dC^hp^). Known dU^f^ (ref. 14) was prepared by deprotection of 2.

**Fig. 1:**
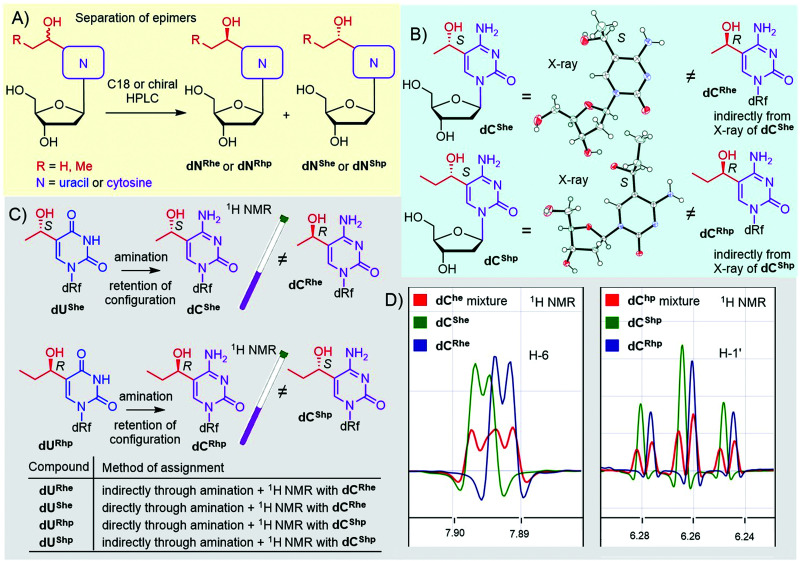
Separation and assignment of epimers of dN^he^ and dN^hp^ nucleosides. (A) separation of epimers; (B) X-ray assignment of dC^he^ and dC^hp^: CCDC 2166141 – dC^She^, CCDC 2166142 – dC^Shp^; (C and D) NMR assignment of dU^he^ and dU^hp^ nucleosides.

In the case of hydroxy derivatives dN^he^ and dN^hp^, a mixture of two diastereoisomers (epimers) was obtained in each case. To study the influence of each epimer on transcription separately, we separated both epimers by HPLC, using either non-chiral or chiral columns (Fig. 1A and Fig. S13–S16 in ESI†). As the new chiral center is distant from the deoxyribose, the determination of the relative and absolute configuration was non-trivial. Fortunately, we succeeded in crystallization of two cytosine derivatives dC^She^ and dC^Shp^ and determined their configuration by X-ray diffraction (Fig. 1B, Fig. S39 and S40 in ESI†), which also indirectly revealed the configuration of the complementary epimers dC^Rhe^ and dC^Rhp^. Uridine derivatives were assigned by an amination reaction (Scheme 1, step iv) on a single epimer of uridine derivative to acquire the cytidine analog (Fig. 1C). The obtained cytidine derivative was then mixed in a single NMR tube with one of the cytidine derivatives of known configuration and the measured ^1^H NMR showed whether the configuration matched or not (Fig. 1C and D). This procedure was performed for dU^She^ and dU^Rhp^ epimers to assign the absolute configuration to all four hydroxyalkyluridine derivatives.

**Scheme 2:**
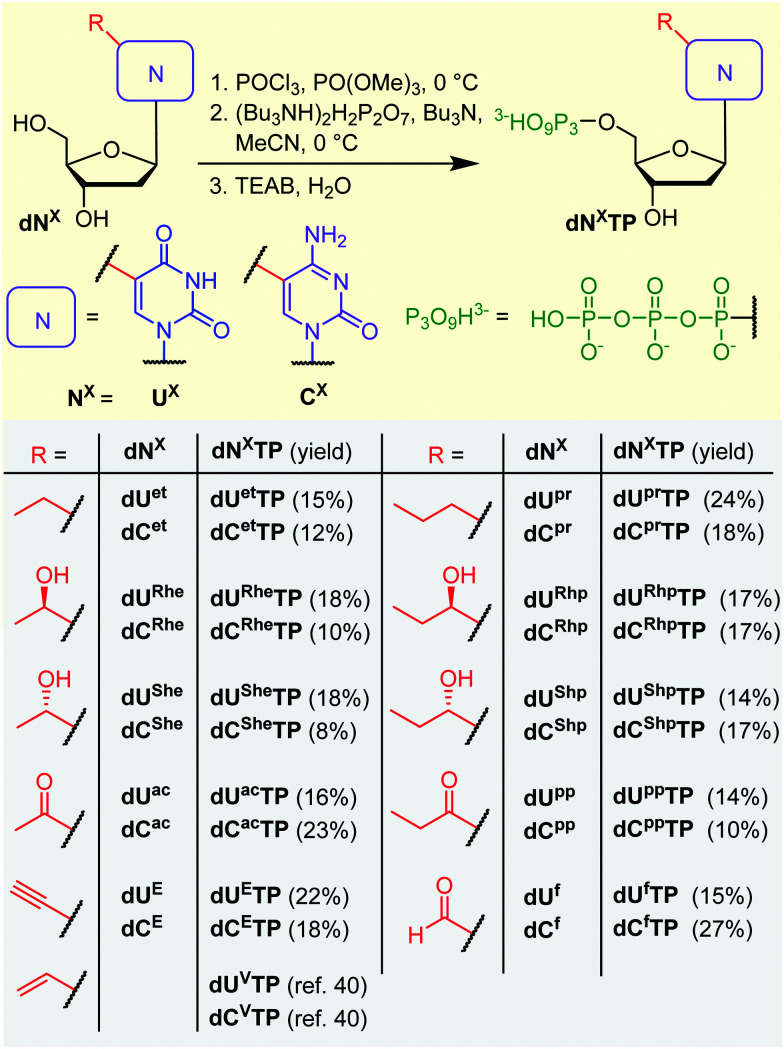
Triphosphorylation of modified dN^X^ nucleosides.

All the prepared modified nucleosides were triphosphorylated to nucleoside triphosphates (dN^X^TPs) in one pot synthesis using slightly modified standard conditions,^51^ with yields ranging from 8 to 27% (Scheme 2). In the case of some uridine derivatives, a non-nucleophilic base proton sponge was used. This was particularly important for the prevention of unwanted acid-catalyzed epimerization of the benzylic chiral center during the initial phosphorylation step of dU^Rhp^ and dU^Shp^ (partial epimerization was observed in the absence of the proton sponge, data not shown). Protection of the hydroxyalkyl group at position 5 was not necessary.

**Fig. 2:**
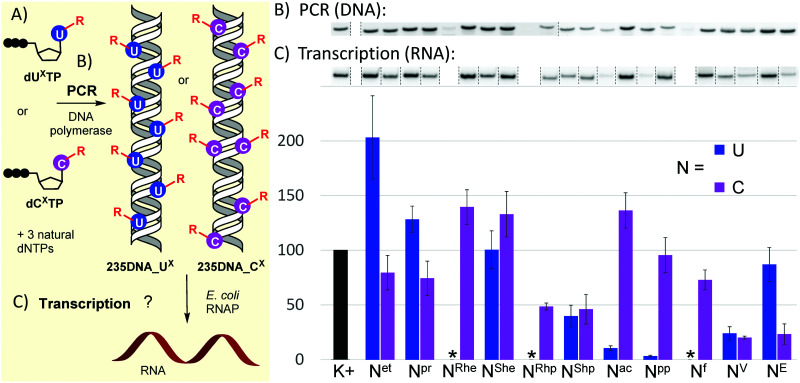
(A) Scheme of PCR synthesis of modified DNA templates and transcription; (B) agarose gel electrophoresis of PCR synthesis of modified 235DNA_N^X^ DNA templates; (C) relative transcription from the modified DNA templates (K+ = non-modified DNA, * = DNA template was not obtained by PCR). For original uncut gels, see the ESI.†

With the full series of modified dN^X^TPs in hand, we tested their substrate activity in enzymatic incorporation into DNA by primer extension reaction (PEX). All the prepared nucleoside triphosphates were good substrates for KOD XL DNA polymerase, as confirmed by both denaturing polyacrylamide gel electrophoresis (PAGE, see Fig. S6 and S7 in ESI†) and mass spectroscopy analysis (see Table S5 and Fig. S17–S38 in ESI†). Subsequently, we used each of them for PCR synthesis of DNA templates for transcription studies. We used a 235-bp template containing the Pveg promoter region as previously described^36,52^ (Fig. 2A). Almost all the modified nucleotides were successfully incorporated by KOD XL DNA polymerase in PCR (Fig. 2B, Fig. S9 in ESI†), although in most cases some optimization together with higher amounts of dN^X^TP and DNA polymerase was needed to prepare full length products in sufficient yields. Only in the case of dC^E^TP we used Vent (exo^−^) polymerase (KOD XL failed to give sufficient amount of PCR amplicon). With three nucleotides: dU^Rhe^TP, dU^Rhp^TP and dU^f^TP, the PCR did not give the desired amplicons with either KOD XL or other DNA polymerases (Vent (exo^−^), Pwo or Taq DNA polymerases, data not shown). In the case of dU^f^TP, this was caused probably by its ability to mispair with both A or G^27,28^ or by formation of Schiff-base cross-links with the polymerase.^53^ Therefore, these modifications were not studied further. Interestingly, *S*-epimers of dU (dU^She^TP, dU^Shp^TP) were good substrates for KOD XL, even under PCR conditions, whereas *R*-epimers (dU^Rhe^TP, dU^Rhp^TP) were very poor substrates and the PCR products were not obtained even after optimization. This stereoselective discrimination in substrate activity was not observed for hydroxyalkylcytidine derivatives, where both epimeric series of nucleotides were successfully incorporated in PCR. At this moment, we do not have any structural explanation for the dichotomy. The PCR products were quantified in order to use them as modified DNA templates for transcription. The quantification was performed with 6-fluorescein-labelled primers or GelRed staining as these methods provide a good balance between accuracy and ease of preparation, avoiding potentially dangerous manipulation with radiolabeled DNA.^31,32,36^

The prepared modified DNA templates containing modified nucleobase U^X^ or C^X^ fully replacing the natural T or C in the whole sequence except for the primers were confirmed by sequencing (see ESI†) and were then tested in multiple round transcription assays, using *E. coli* RNAP in a reaction supplemented with α-^32^P-UTP to label the transcript (Fig. 2B and C; for original uncut gels, see Fig. S11 and S12 in ESI†). The transcription products were quantified and the yields normalized for the relative amount of the DNA template and compared to those obtained with a non-modified natural DNA template (K+). Ethyl modification at U (U^et^) significantly increased transcription efficiency (*ca.* 200%), while 5-ethynyl-dC had a slightly suppressing effect (79%). The more bulky propyl modification on U also had a modest stimulatory effect (128%), while on C it moderately decreased the transcription efficiency (74%). The 1-hydroxyethyl modification on U had no apparent effect (U^She^, *ca.* 100%), whereas it slightly enhanced the transcription when attached to C (C^Rhe^ at 139%, C^She^ at 133%). No significant difference was observed between the *R*- or *S*-epimer in the deoxycytidine series. A similar lack of stereodiscrimination was observed in 1-hydroxypropyl modification, albeit with a suppressing effect on both U (U^Shp^, 40%) and C (C^Rhp^ at 49%, C^Shp^ at 46%). The most pronounced effect on transcription was observed with acylpyrimidine derivatives. The presence of an acetyl or propionyl group at U almost completely inhibited transcription (U^ac^ 11%, U^pp^ 3%), while the acetyl group attached to C had a slightly enhancing effect (C^ac^ at 136%) and the propionyl-modified C allowed similar transcription as natural DNA (C^pp^ at 96%). The formyl group at C exerted only a minor suppression effect (C^f^, 73%). Both vinyl- and ethynyl-modified templates were previously studied with a longer 339-bp template containing the same Pveg promoter,^29,30^ so they were also included in this study for comparison. Vinyl modification of both U and C had a strong suppressing effect on transcription (U^V^, 24%; C^V^, 20%), whereas the ethynyl group at C or U showed differential effects: strong suppression when present at C (C^E^, 23%) and only weak suppression when present at U (U^E^, 87%).

In conclusion, we have designed and prepared 5-ethyl-, 5-propyl-, epimeric 5-(1-hydroxyethyl)- and 5-(1-hydroxypropyl)-, as well as 5-acetyl and 5-propionyl-uracil and -cytosine 2′-deoxyribonucleosides and their corresponding dN^X^TPs as homologues of natural epigenetic pyrimidines derived from 5mC and its oxidized congeners. We have also successfully separated and identified individual epimers of 5-(1-hydroxyethyl)- and 5-(1-hydroxypropyl)pyrimidine nucleosides based on X-ray and NMR analysis. Most of the dN^X^TPs were good substrates for DNA polymerases and were used in PCR synthesis of modified 235-bp DNA templates. Finally, we systematically studied their effect on transcription with bacterial RNAP.

Although the studied pyrimidine modifications have not been detected among the natural epigenetic modifications in genomic DNA (at least not yet), this work brings several important and biologically relevant insights and suggests some prospective applications. We revealed how amazingly robust RNAP is in its ability to interact with DNA decorated with complex modifications and identified both stimulatory and inhibitory effects of some modifications. The surprisingly strong enhancing effect of 5-ethyuracil (200% transcription compared to T) could be then used in biotechnology to increase the transcription efficiency from modified plasmids, possibly even in production of certain therapeutic RNAs. Moreover, the 5-ethyl-2′-deoxyuridine nucleoside could be a potential epigenetic regulator with the opposite effect to 5-aza-dC. Interestingly, some of these modifications are similar in size and functionality to some recently discovered rare DNA nucleobases, so the effects described here may correspond with their roles in Nature. The dichotomy in the effect of acetylpyrimidine derivatives opens the possibility of turning OFF transcription through deamination of C^Ac^ to U^Ac^ by activation-induced cytidine deaminase previously shown to deaminate C^he^.^37^ Conversely, the ten-eleven translocation 2 (TET2) enzyme that was recently shown^37^ to oxidize C^et^ to C^he^ could be used to slightly increase the transcription. These potential applications, studies of mechanistic aspects of the effects of the modified nucleobases on transcription, and studies of these modifications in eukaryotic transcription systems will be further pursued in our labs.

## Conflicts of interest

There are no conflicts to declare.

## Supplementary Material

CB-003-D2CB00133K-s001

CB-003-D2CB00133K-s002
