# Supplementary material for: Homologues of epigenetic pyrimidines: 5-alkyl-, 5-hydroxyalkyl and 5-acyluracil and -cytosine nucleotides: synthesis, enzymatic incorporation into DNA and effect on transcription with bacterial RNA polymerase
Source: RSC Chem Biol. 2022 Jun 30;3(8):1069–75. doi: 10.1039/d2cb00133k (PMC9347353; doi:10.1039/d2cb00133k)
Supplement: CB-003-D2CB00133K-s001 [file CB-003-D2CB00133K-s001.pdf]

Electronic Supplementary Material (ESI) for RSC Chemical Biology.
This journal is © The Royal Society of Chemistry 2022

Electronic Supporting Information

Homologues of epigenetic pyrimidines: 5-alkyl-, 5-hydroxyalkyl and 5-acyluracil and –cytosine nucleotides. Synthesis, enzymatic incorporation into DNA and effect on transcription with bacterial RNA polymerase

Filip Gracias,[a] Olatz Ruiz-Larrabeiti,[b] Viola Vaňková Hausnerová,[b] Radek Pohl,[a] Blanka Klepetářová,[a] Veronika Sýkorová,[a] Libor Krásný,*[b] and Michal Hocek*[a,c]

a) Institute of Organic Chemistry and Biochemistry, Czech Academy of Sciences, Flemingovo nam. 2, CZ-16000 Prague 6, Czech Republic; hocek@uochb.cas.cz

b) Lab. of Microbial Genetics and Gene Expression, Institute of Microbiology, Czech Academy of Sciences, Vídeňská 1083, CZ-14220 Prague 4, Czech Republic; krasny@biomed.cas.cz

c) Department of Organic Chemistry, Faculty of Science, Charles University, Hlavova 8, CZ-12843 Prague 2, Czech Republic.

Table of contents

1. **Experimental section – organic chemistry part**
   - 1.1. General remarks – organic synthesis
   - 1.2. Synthetic schemes
   - 1.3. Synthesis of nucleosides
     - 1.3.1. Synthesis of ethyl derivatives
     - 1.3.2. Synthesis of propyl and formyl derivatives
   - 1.4. Synthesis of nucleoside triphosphates
     - 1.4.1. Synthesis of **dU^{V}TP** and **dC^{V}TP**
   - 1.5. Assignment of epimers
     - 1.5.1. Crystallization of epimers
2. **Experimental section – biochemistry part**
   - 2.1. General remarks – biochemistry
   - 2.2. Procedure for single strand DNA generation by streptavidin magnetic beads
   - 2.3. Procedure for purification of DNA on Agencourt AMPure XP magnetic particles
   - 2.4. Quantification of PCR products
   - 2.5. Enzymatic synthesis (PEX) of **dU^{X}**/**dC^{X}**-modified DNA
     - 2.5.1. Single incorporation of one modified **dN^{X}TP** using 19-mer template – analytical scale
     - 2.5.2. Single incorporation of one modified **dN^{X}TP** using 19-mer template – semi-preparative scale
   - 2.6. Polymerase chain reaction (PCR)
     - 2.6.1. Preparation of **Temp^{235}**
     - 2.6.2. PCR using **Temp^{235}** template and **dU^{X}TPs/dC^{X}TPs**
     - 2.6.3. PCR using **235DNA_U^{X}** and **235DNA_C^{X}** templates
   - 2.7. Transcription studies
     - 2.7.1. Multiple round transcription experiments
3. **Copies of HPLC chromatograms**
4. **MALDI-TOF measurements**
   - 4.1. Copies of MALDI-TOF spectra
5. **X-ray measurements**

6. **Copies of NMR spectra**

6.1. NMR spectra for full characterizations

6.2. NMR spectra used for the assignment of epimers

7. **Sanger sequencing**

7.1. Results of Sanger sequencing

8. **References**

1. Experimental section – organic chemistry part

In this work, 22 nucleoside triphosphates were synthetized (Figure S1). The synthesis of ethyl derivatives and congeners is summarized in Figure S2, and the propyl derivatives and congeners, together with formyl derivative in Figure S3. Vinyl- (Figure S4) and ethynyl-modified triphosphates were also prepared for direct comparison with our previous study of transcription.[1]

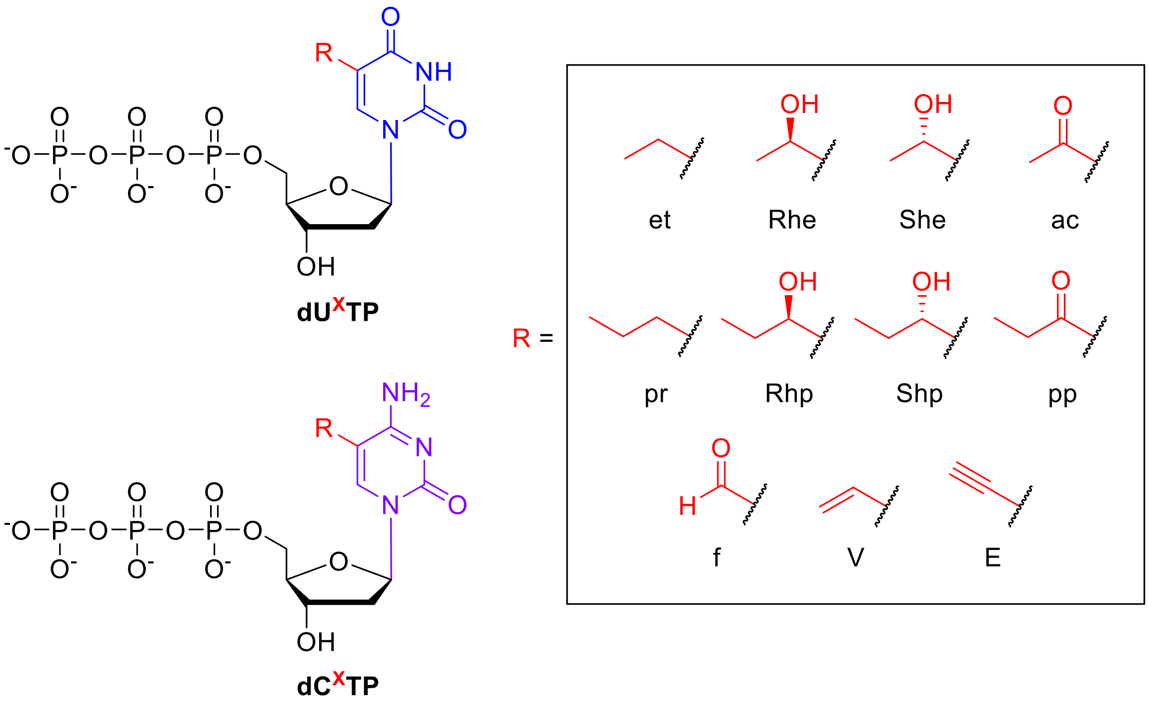

Figure S1. Structures of nucleoside triphosphates used in transcription studies

1.1. General remarks – synthetic part

Reagents and solvents were purchased from commercial suppliers (Fluorochem, Sigma–Aldrich, Acros Organics and Alfa Aesar), and were used without further purification unless stated otherwise. Phosphoryl chloride ($POCl_3$) and trimethyl phosphate [$PO(OMe)_3$] were distilled prior to use. Tetrahydrofuran (THF) was dried by distillation over sodium metal. Other dried solvents were purchased from Acros Organics. Unless stated otherwise, all reactions were performed in a heatgun-dried glassware under argon atmosphere, using standard septa techniques. The reactions were monitored by thin-layer chromatography (TLC) using silica gel 60 F254 plates (Merck) and visualized by UV (254 nm) or with Advion Expression Compact Mass Spectrometer connected with Plate Express® TLC Plate Reader using electrospray ionization. Column chromatography was performed using silica gel (40–63 μm, Fluorochem) either by hand or by flash liquid chromatography system (FLC) Teledyne ISCO CombiFlash Rf 200 or 300. Reverse phase (RP) and diol-modified columns for FLC were purchased from Teledyne ISCO. Purifications of nucleoside triphosphates and epimer separations were performed using HPLC (Waters modular HPLC system), using Phenomenex Kinetex column (Kinetex® 5 μm EVO C18 100 Å, AXIA Packed LC Column 250 x 21.2 mm), Luna column (Luna Omega 5 μm Polar C18 100 Å, AXIA Packed, LC Column 150 x 21.2 mm), Waters X-Bridge (XBridge BEH Shield RP18 OBD Prep Column, 130Å, 5 μm, 19 x 150 mm) and POROS HQ 50 (lab-packed, 26×120 mm). Chiral separation was done on Interchim HPLC system, using Daicel column (DAICEL CHIRALPAK IE, 20 x 250 mm). Crude separation of some nucleoside triphosphates was done on Biotage SP1 apparatus, using DEAE SEPHADEX A-25 sodium form column. NMR spectra were measured on Bruker AVANCE 400 III HD (^{1}H at 401.0 MHz and ^{13}C at 100.8 MHz), Bruker AVANCE 500 III HD (^{1}H at 500.0 MHz, ^{13}C at 125.7 MHz and ^{31}P at 202.4 MHz), Bruker AVANCE 600 III HD (^{1}H at 600.0 MHz and ^{13}C at 150.9 MHz) and JEOL ECZR 500 (^{1}H at 500.2 MHz, ^{13}C at 125.8 MHz, ^{31}P at 202.5 MHz) in CD_3OD, $CDCl_3$ or D_2O solutions at 25 °C. Chemical shifts (in ppm, δ scale) were referenced to the residual solvent signal in ^{1}H spectra (δ (CHD_2OD) = 3.31 ppm, δ ($CHCl_3$) = 7.26 ppm) or to the solvent signal in ^{13}C spectra (δ (CD_3OD) = 49.0 ppm, δ ($CDCl_3$) = 77.0 ppm. tBuOH was used as an internal standard for D_2O solutions (1.24 ppm for ^{1}H and 32.4 ppm for ^{13}C). Coupling constants (J) are given in Hz. The complete assignment of ^{1}H and ^{13}C signals was performed by an analysis of the correlated homonuclear H,H-COSY, heteronuclear

H,C-HSQC and H,C-HMBC spectra. High resolution mass spectra were measured on LTQ Orbitrap XL spectrometer (Thermo Fisher Scientific). Compounds **dU^{E}**, **dC^{E}**, TBS-protected **dU^{I}**, **dC^{f}** and **dU^{E}TP** were prepared according to published procedures.[2,3,4,5,6]

1.2. Synthetic schemes

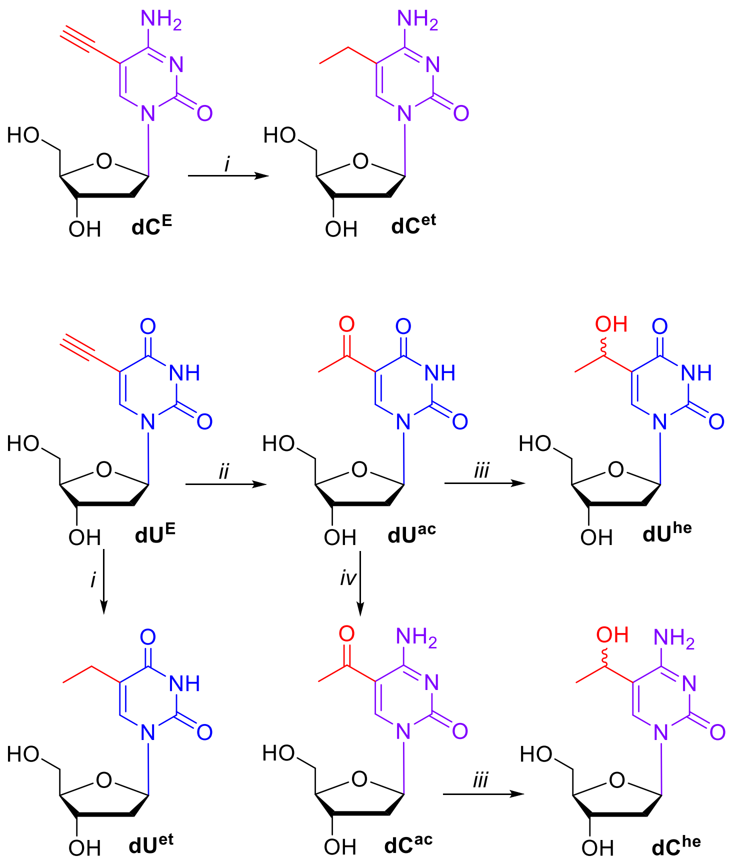

Figure S2. Synthetic overview of ethyl derivatives. Conditions: *i*) 10% Pd/C, H_2, MeOH, 23 °C, 40 h; *ii*) H_2SO_4, MeOH, H_2O, 75 °C, 3 h; *iii*) $NaBH_4$, $CeCl_3$*$7H_2O$, MeOH, 23 °C, 2 h; *iv*) PyAOP, DBU, NH_4OH, DMF, 23 °C, 2 h. For more information, see section 1.3.1.

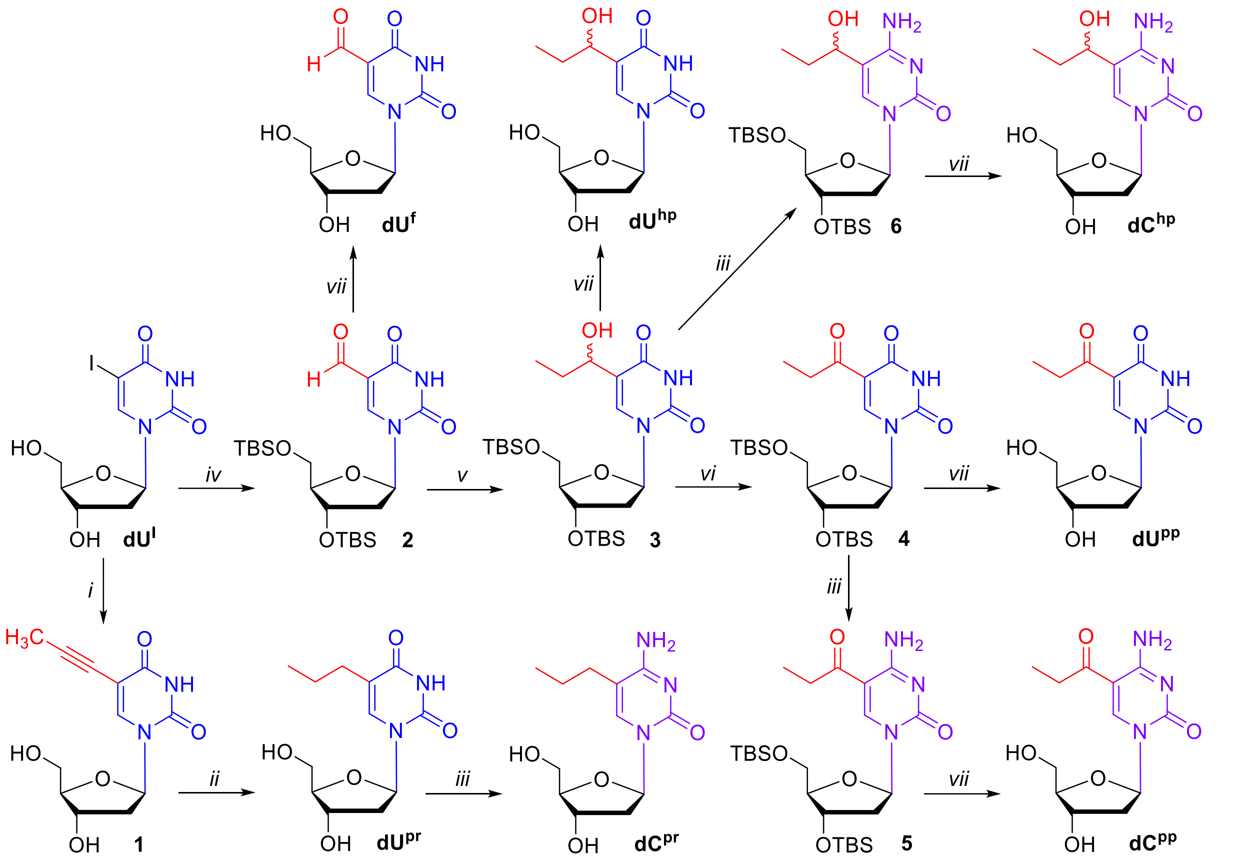

Figure S3. Synthetic overview of propyl and formyl derivatives. Conditions: *i*) propyne, $Pd(PPh_3)_4$, CuI, DMF, 23 °C, 2 h; *ii*) 10% Pd/C, H_2, MeOH, 23 °C, 40 h; *iii*) PyAOP, DBU, NH_4OH, DMF, 23 °C, 2 h; *iv*) TBSCl, imidazole, DMF, 23 °C, 18 h, then $Pd(PPh_3)_4$, CO, Bu_3SnH, toluene, 60 °C, 18 h; *v*) EtMgBr, THF, -78 °C, 4 h; *vi*) DMP, DCM, 23 °C, 3 h; *vii*) Et_3N*3HF, THF, 23 °C, 18 h. For more information, see section 1.3.2.

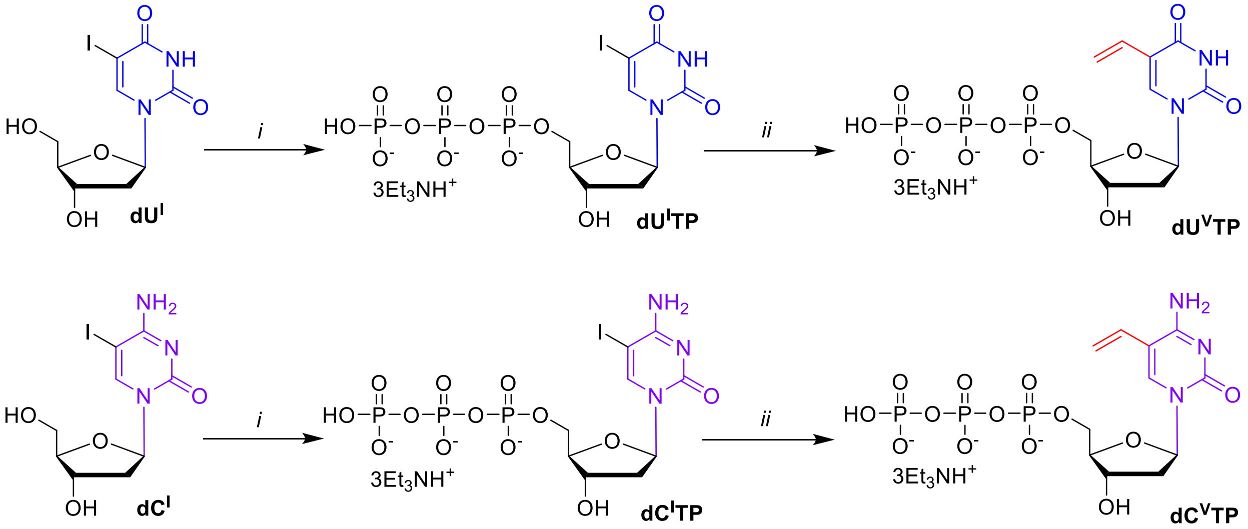

Figure S4. Reaction scheme of vinyl-modified triphosphates. Conditions: *i*) $POCl_3$, $PO(OMe)_3$, MeCN, Bu_3N, $(Bu_3NH)_2H_2P_2O_7$, TEAB, 0 °C. *ii*) vinyl-BF_3K, Cs_2CO_3, $Pd(OAc)_2$, TPPTS, H_2O/MeCN, 80 °C. For more information, see section 1.4.

1.3. Synthesis of nucleosides

1.3.1. Synthesis of ethyl derivatives

General procedure for hydrogenation (GP1)

Starting material was dissolved in MeOH (c = 0.1 M) and deggased for 5 min by constant flow of argon through the solvent. 10% Pd/C (0.2 equiv.) was added, the flask was evacuated and backfilled with hydrogen gas using a balloon. The reaction was then stirred at 23 °C for 18 hours under hydrogen atmosphere. After the reaction has gone to completion, the flask was purged with argon for 5 min, solution was filtered over a celite plug and the solvent was evaporated under vacuum. The crude product was purified either by FLC or RP-FLC or combination of both.

5-Ethyl-2′-deoxyuridine (dU^{et})

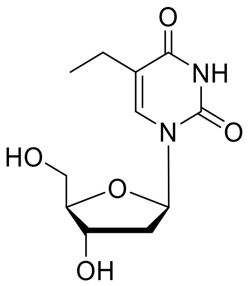

The compound was prepared according to **GP1**, starting from **dU^{E}** (370 mg, 1.47 mmol). The product was purified by FLC (5 to 20% MeOH in DCM) affording a white powder (317 mg, 84%). NMR data were in accordance with the literature.[7]

5-Ethyl-2′-deoxycytidine (dC^{et})

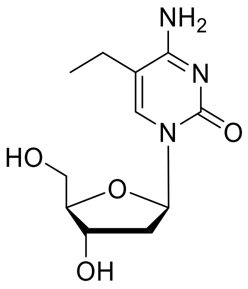

The compound was prepared according to **GP1**, starting from **dC^{E}** (400 mg, 1.59 mmol). After FLC (10 to 30% MeOH in DCM), pure product was acquired as a white powder (238 mg, 60%). NMR data were in accordance with the literature.[8]

5-Acetyl-2′-deoxyuridine (dU^{ac})

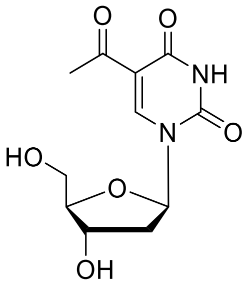

Product was syntetized according to published procedure.[9] **dU^{E}** (1.5 g, 5.95 mmol) was transfered into 500 mL flask and suspended in a mixture of MeOH (216 mL) and H_2O (24 mL). The reaction mixture was heated in an oil bath at 75 °C. When the reaction mixture reached the temperature, H_2SO_4 (0.34 mL, 1 equiv.) was added and the reaction was stirred for 3 hours. The progress of the reaction was monitored by TLC/MS. After complete comsumption of the starting material, the reaction was taken out of the bath and allowed to cool down to 23 °C. The reaction mixture was neutralized with saturated solution of $NaHCO_3$ to a neutral pH. A white precipitate was filtrated and the solvents were evaporated under vacuum. FLC (0 to 50% MeOH in DCM) afforded an off-white solid product (1.10 g, 69%). NMR data were in accordance with the literature.[9]

General procedure for uracil amination to cytosine (GP2)

This method was following a published procedure.[10] Starting material (1 equiv.) and (7-azabenzotriazol-1-yloxy)trispyrrolidinophosphonium hexafluorophosphate (PyAOP, 1.7 equiv.) were dried together in high vacuum for 15 min followed by addition of DMF (c = 0.33 M) at 23 °C. Next, 1,8-diazabicyclo[5.4.0]undec-7-ene (DBU, 1.7 equiv.) was added quickly. After 1 minute, aqueous ammonia (25%, 4 equiv.) was added, the reaction was stirred for another 2 hours and then stopped by addition of saturated solution of NH_4Cl. The reaction mixture was evaporated under reduced pressure and co-distilled twice with water.

5-Acetyl-2′-deoxycytidine (dC^{ac})

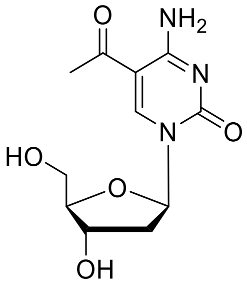

The compound was synthesized using **GP2**, starting from **dU^{ac}** (0.93 g, 3.44 mmol). FLC (5 to 30% MeOH in DCM) afforded the product as an off-white powder (0.62 g, 67%). NMR data were in accordance with the literature.[9]

5-(1-Hydroxyethyl)- 2′-deoxyuridine (dU^{he})

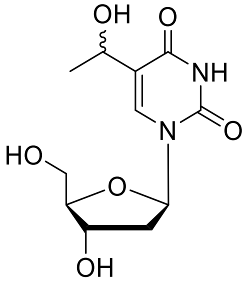

dU^{ac} (491 mg, 1.82 mmol) was dissolved in MeOH (18 mL). Then, $CeCl_3$ heptahydrate (2.03 g, 3 equiv.) was added. Mixture was stirred for 15 minutes at 23 °C and then cooled to 0 °C using an

ice bath. $NaBH_4$ (83 mg, 1.2 equiv.) was added in small portions over 5 min. After additional 30 min of stirring, the mixture was warmed to 23 °C and stirred for extra 1 hour. The reaction was stopped by addition of saturated solution of NH_4Cl. The solvents were evaporated and the mixture was separated by RP-FLC (10 to 40% MeOH in H_2O) providing the mixture of both epimers as an off-white solid (242 mg, 49%, epimeric ratio 1:1). Both epimers were separated by HPLC (0 to 40% MeOH in H_2O, 15 mL/min, Phenomenex Kinetex EVO C18) (see section 3, Figure S13). NMR data were in accordance with the literature.[11]

5-(1-Hydroxyethyl)- 2′-deoxycytidine (dC^{he})

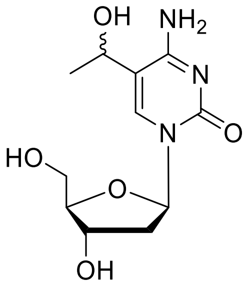

The compound was prepared in the same manner as **dU^{he}** using 400 mg (1.49 mmol) of **dC^{ac}**. FLC (10 to 50% MeOH in DCM) afforded the mixture of both epimers as an off-white solid (203 mg, 51%, epimeric ratio 1:1). Both epimers were separated by HPLC (0 to 40% MeOH in H_2O, 15 mL/min, Phenomenex Kinetex EVO C18) (see Section 3, Figure S14). NMR data were in accordance with the literature.[12]

1.3.2. Synthesis of propyl and formyl derivatives

5-(Prop-1-ynyl)- 2′-deoxyuridine (1)

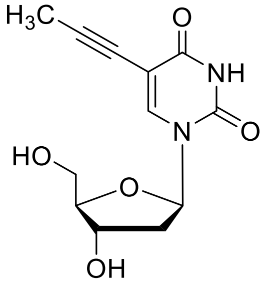

The compound was prepared according to published procedure.[13] The starting compound **dU^{I}** (708 mg, 2 mmol) was dissolved in DMF (10 mL) and the solution was deggased by argon for 20 min. Then, $Pd(PPh_3)_4$ (231 mg, 0.2 mmol), CuI (76 mg, 0.4 mmol) and Et_3N (560 µL, 2 equiv.) were added respectively and the mixture was stirred at 23 °C for 2 min. Next, propyne (prepared according to published procedure from 1,2-dibromopropane[14]) was added and the reaction mixture was stirred for 4 hours at 23 °C. Solvents were evaporated under vacuum and after purification by FLC (0 to 15% MeOH in DCM), the reaction yielded a white solid product (359 mg, 67%). NMR data were in accordance with the literature.[13]

5-Propyl-2′-deoxyuridine (dU^{pr})

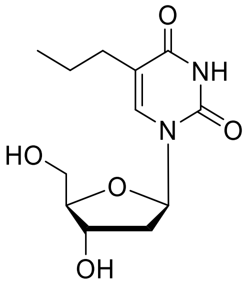

The compound was prepared according to **GP1**, starting from **1** (80 mg, 0.3 mmol). FLC (0 to 20% MeOH in DCM) yielded the product as a white powder (60 mg, 74%). NMR data are in accordance with the literature.[15]

NMR: ^{1}H NMR (401.0 MHz, CD_3OD): 0.94 (t, 3H, J_{vic} = 7.4, C**H**$_3$CH$_2$CH$_2$); 1.53 (h, 2H, J_{vic} = 7.4, CH$_3$C**H**$_2$CH$_2$); 2.14 - 2.34 (m, 4H, CH$_3$CH$_2$C**H**$_2$; H-2′); 3.73 (dd, 1H, J_{gem} = 12.0, $J_{5'b,4'}$ = 3.5,

H-5′b); 3.80 (dd, 1H, J_{gem} = 12.0, $J_{5'a,4'}$ = 3.1, H-5′a); 3.92 (q, 1H, J_{vic} = 3.3, H-4′); 4.37 - 4.44 (m, 1H, H-3′); 6.29 (dd, 1H, $J_{1',2'b}$ = 7.0, $J_{1',2'a}$ = 6.4, H-1′); 7.84 (t, 1H, $J_{6,CH3CH2C\mathbf{H}2}$ = 1.0, H-6). ^{13}C NMR (100.8 MHz, CD_3OD): 13.95 (**C**$H_3CH_2CH_2$); 22.74 (CH_3**C**H_2CH_2); 29.79 (CH_3CH_2**C**H_2); 41.33 (CH_2-2′); 62.79 (CH_2-5′); 72.25 (CH-3′); 86.36 (CH-1′); 88.87 (CH-4′); 115.69 (C-4); 138.30 (CH-6); 152.29 (C-2); 166.07 (C-4).

HRMS (ESI^+): *m/z* calcd for $C_{12}H_{18}O_5N_2Na$ [M + Na^+] 293.11079; found: 293.11049.

5-Propyl-2′-deoxycytidine (dC^{pr})

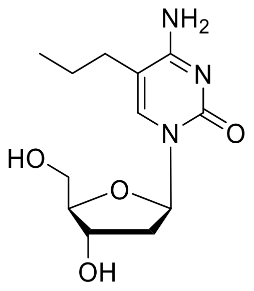

The compound was synthesized using **GP2**, starting from **dU^{pr}** (100 mg, 0.370 mmol). After the addition of NH_4OH, reaction was stirred for 3 hours at 23 °C. Purification by FLC (0 to 20% MeOH in DCM) followed by RP-FLC (10 to 50% MeOH in H_2O, C18 column) yielded an off-white solid product (57 mg, 57%). NMR data are in accordance with the literature.[15]

NMR: ^{1}H NMR (401.0 MHz, CD_3OD): 0.98 (t, 3H, J_{vic} = 7.3, C**H**$_3CH_2CH_2$); 1.57 (tq, 2H, J_{vic} = 8.1, 7.3, CH_3C**H**$_2CH_2$); 2.15 (dt, 1H, J_{gem} = 13.4, $J_{2'b,1'}$ = $J_{2'b,3'}$ = 6.5, H-2′b); 2.31 (t, 2H, J_{vic} = 8.1, CH_3CH_2C**H**$_2$); 2.33 (ddd, 1H, J_{gem} = 13.4, $J_{2'a,1'}$ = 6.2, $J_{2'a,3}$ = 3.9, H-2′a); 3.74 (dd, 1H, J_{gem} = 12.0, $J_{5'b,4'}$ = 3.6, H-5′b); 3.82 (dd, 1H, J_{gem} = 12.0, $J_{5'a,4'}$ = 3.1, H-5′a); 3.93 (ddd, 1H, $J_{4',3'}$ =3.9, $J_{4',5'}$ = 3.6, 3.1, H-4′); 4.38 (dt, 1H, $J_{3',2'}$ = 6.5, 3.9, $J_{3',4'}$ = 3.9, H-3′); 6.27 (dd, 1H, J_{vic} = 6.5, 6.2, H-1′); 7.90 (t, 1H, $J_{6,CH3CH2C\mathbf{H}2}$ = 1.0, H-6). ^{13}C NMR (100.8 MHz, CD_3OD): 13.87 (**C**$H_3CH_2CH_2$); 22.27 (CH_3**C**H_2CH_2); 30.09 (CH_3CH_2**C**H_2); 42.14 (CH_2-2′); 62.62 (CH_2-5′); 71.89 (CH-3′); 87.45 (CH-1′); 88.82 (CH-4′); 108.37 (C-5); 140.12 (CH-6); 157.93 (C-2); 166.66 (C-4).

HRMS (ESI^+): *m/z* calcd for $C_{12}H_{20}O_4N_3$ [M + H^+] 270.14483; found: 270.14470.

3', 5'-Di(*tert*-butyldimethylsilyl)-5-formyl-2′-deoxyuridine (2)

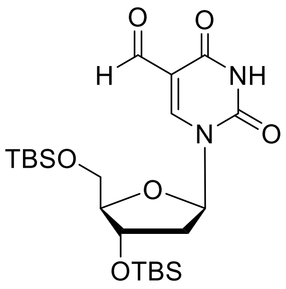

The compound was prepared according to published procedure.[4] TBS-protected **dU^I^** (11.02 g, 18.9 mmol) was dried in high vacuum overnight. Next day, degassed toluene (120 mL) was added followed by $Pd(PPh_3)_4$ (2.18 g, 0.1 equiv.). Carbon monoxide (CO) was then passed through the solution for 10 minutes with strong stirring. The reaction mixture was heated to 60 °C, pressurized to 3.4 bar using a CO gas and Bu_3SnH (5.6 mL, 1.1 equiv.) was added slowly via syringe pump over 10 hours followed by additional 18 hours of stirring. The mixture was allowed to reach room temperature, the flask was de-pressurized and the source of CO was removed. The solvent was evaporated and the crude product was purified by FLC (10 to 50% EtOAc in cHex). Crystallization from hot cyclohexane yielded yellowish solid product (7.01 g, 77%). NMR data were in accordance with the literature.[4]

3', 5'-Di(*tert*-butyldimethylsilyl)-5-(1-hydroxypropyl)- 2′-deoxyuridine (3)

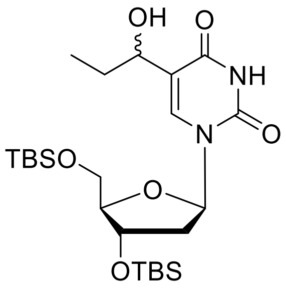

Compound **2** (6.0 g, 12.4 mmol) was dissolved in THF (83 mL) and cooled down to -78 °C. After 10 min, a solution of EtMgBr (3 M in Et_2O, 9.1 mL, 2.2 equiv.) was added dropwise over 5 min. The reaction mixture was stirred for 2 hours followed by another addition of EtMgBr (3 M in Et_2O, 2 mL, 0.5 equiv.). The reaction was stirred for additional 1 hour, then stopped by addition of 1 M HCl (33 mL) and allowed to reach 23 °C over 30 min before neutralizing the reaction by

addition of saturated solution of $NaHCO_3$. THF was evaporated and the precipitated crude product was purified by FLC (0 to 10% MeOH in DCM) affording a light yellow solid (4.5 g, 70%, epimeric ratio 5:4).

NMR: ^{1}H NMR (500.0 MHz, $CDCl_3$): 0.070, 0.072, 0.077, 0.080, 0.96, 0.099 (6 × s, 24H, CH_3Si); 0.89, 0.91, 0.92 (3 × s, 36H, $(CH_3)_3CSi$); 0.95, 0.97 (2 × t, 2 × 3H, J_{vic} = 7.6, C**H₃**CH₂CHOH); 1.68 – 1.88 (m, 4H, CH₃C**H₂**CHOH); 1.94 – 2.03 (m, 2H, H-2′b); 2.23 – 2.31 (m, 2H, H-2′a); 2.96, 3.08 (2 × bs, 2 × 1H, CH₃CH₂CHO**H**); 3.75 (dd, 2H, J_{gem} = 11.3, $J_{5'b,4'}$ = 3.2, H-5′b); 3.80, 3.81 (2 × dd, 2 × 1H, J_{gem} = 11.3, $J_{5'a,4'}$ = 3.2, H-5′a); 3.92 – 3.95 (m, 2H, H-4′); 4.28 (dd, 1H, J_{vic} = 7.3, 5.9, CH₃CH₂C**H**OH); 4.36 (dd, 1H, J_{vic} = 7.9, 5.3, CH₃CH₂C**H**OH); 4.39 4.41 (2 × dt, 2 × 1H, $J_{3',2'}$ = 5.4, 2.4, $J_{3',4'}$ = 2.4, H-3′); 6.29, 6.30 (2 × dd, 1H, $J_{1',2'}$ = 8.0, 5.4, H-1′); 7.52, 7.53 (2 × d, 2 × 1H, ^4J = 0.7, H-6); 8.87, 8.88 (2 × bs, 2 × 1H, NH). ^{13}C NMR (125.7 MHz, $CDCl_3$): -5.43, -5.40, -5.38, -4.85, -4.69, -4.66 (CH_3Si); 10.22, 10.29 (**C**H₃CH₂CHOH); 17.98, 17.99, 18.44 ((CH₃)₃**C**Si); 25.73, 25.95, 25.96 ((**C**H₃)₃CSi); 29.08, 29.37 (CH₃**C**H₂CHOH); 41.17, 41.28 (CH₂-2′); 63.01, 63.10 (CH₂-5′); 70.73, 71.60 (CH₃CH₂**C**HOH); 72.15, 72.28 (CH-3′); 85.09, 85.16 (CH-1′); 87.88, 87.92 (CH-4′); 116.22, 116.46 (C-5); 135.90, 135.93 (CH-6); 149.70, 149.73 (C-2); 163.21 (C-4).

HRMS (ESI$^+$): *m/z* calcd for $C_{24}H_{46}O_6N_2Si_2$ [M + H$^+$] 537.27866; found: 537.27783.

3’, 5’-Di(*tert*-butyldimethylsilyl)-5-(1-oxopropyl)- 2′-deoxyuridine (4)

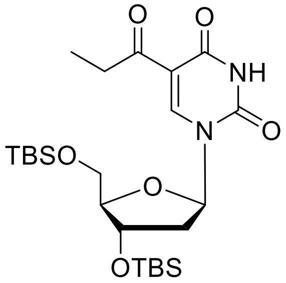

Hydroxyl derivative **3** (440 mg, 0.885 mmol) was dissolved in DCM (10 mL) and Dess-Martin periodinate (DMP, 544 mg, 1.5 equiv.) was added in one portion. The reaction mixture was stirred for 3 hours at 23 °C and then stopped by addition of saturated solution of $NaHCO_3$ (5 mL). The layers were separated and the aqueous layer was extracted with DCM (10 mL). The combined organic layers were washed with water (10 mL), brine (10 mL), dried over Na_2SO_4,

filtered through a celite plug, evaporated and purified by FLC (0 to 30% EtOAc in cHex) affording the pure product as a white powder (365 mg, 83%).

NMR: ^{1}H NMR (500.0 MHz, $CDCl_3$): 0.08, 0.09, 0.10 (3 × s, 12H, CH_3Si); 0.87, 0.89 (2 × s, 2 × 9H, $(CH_3)_3CSi$); 1.11 (t, 3H, J_{vic} = 7.2, C**H₃**CH_2CO); 2.06 (ddd, 1H, J_{gem} = 13.2, $J_{2'b,1'}$ = 7.8, $J_{2'b,3'}$ = 5.8, H-2′b); 2.41 (ddd, 1H, J_{gem} = 13.2, $J_{2'a,1'}$ = 5.7, $J_{2'a,3'}$ = 2.2, H-2′a); 2.95 – 3.08 (m, 2H, CH_3C**H₂**CO); 3.77 (dd, 1H, J_{gem} = 11.3, $J_{5'b,4'}$ = 3.2, H-5′b); 3.82 (dd, 1H, J_{gem} = 11.3, $J_{5'a,4'}$ = 3.6, H-5′a); 4.04 (ddd, 1H, $J_{4',5'}$ = 3.6, 3.2, $J_{4',3'}$ = 2.2, H-4′); 4.41 (dt, 1H, $J_{3',2'}$ = 5.8, 2.2, $J_{3',4'}$ = 2.2, H-3′); 6.22 (dd, 1H, $J_{1',2'}$ = 7.8, 5.7, H-1′); 8.55 (s, 1H, H-6); 8.89 (bs, 1H, NH). ^{13}C NMR (125.7 MHz, $CDCl_3$): -5.67, -5.54, -4.85, -4.71 (CH_3Si); 7.84 (**C**H_3CH_2CO); 17.99, 18.35 ($(CH_3)_3$**C**Si); 25.72, 25.89 ((**C**$H_3)_3CSi$); 36.01 (CH_3**C**H_2CO); 41.97 (CH_2-2′); 63.10 (CH_2-5′); 72.83 (CH-3′); 86.87 (CH-1′); 88.90 (CH-4′); 112.42 (C-5); 146.52 (CH-6); 149.48 (C-2); 160.98 (C-4); 196.54 (CH_3CH_2**C**O).

HRMS (ESI$^+$): *m/z* calcd for $C_{24}H_{45}O_6N_2Si_2$ [M + H$^+$] 513.28107; found: 513.28060.

3’, 5’-Di(*tert*-butyldimethylsilyl)-5-(1-hydroxypropyl)- 2′-deoxycytidine (5)

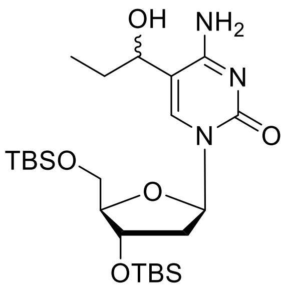

The compound was prepared according to **GP2,** starting from **3** (520 mg, 1.01 mmol). After the reaction was finished, the mixture was diluted with water (10 mL) and extracted with EtOAc (2 x 20 mL). The combined organic layers were washed with water (10 mL), brine (10 mL) and dried over Na_2SO_4. The solvent was evaporated and the mixture was purified by FLC (0 to 30% MeOH in EtOAc) affording the product as a white foam (391 mg, 75%, epimeric ratio 5:4).

NMR: ^{1}H NMR (500.0 MHz, CD_3OD): 0.11, 0.12, 0.132, 0,135 (4 × s, 4 × 6H, CH_3Si); 0.88 – 0.98 (m, 42H, C**H₃**CH_2CH, $(CH_3)_3CSi$); 1.67 – 1.82 (m, 4H, CH_3C**H₂**CH); 2.02 – 2.11 (m, 2H, H-2′b); 2.28 – 2.35 (m, 2H, H-2′a); 3.80 – 3.85 (m, 4H, H-5′); 3.93 – 3.98 (m, 2H, H-4′); 4.42 – 4.48 (m, 4H, H-3′, CH_3CH_2C**H**); 6.30 (dd, 2H, $J_{1',2'}$ = 7.9, 5.8, H-1′); 7.69, 7.72 (s, 1H, H-6).

^{13}C NMR (125.7 MHz, CD_3OD): -5.19, -5.17, -5.16, -5.15, -4.63, -4.50 (CH_3Si); 10.48 (**C**H_3CH_2CH); 18.82, 19.25, 19.28 ($(CH_3)_3$**C**Si); 26.27, 26.52, 26.54 ((**C**$H_3)_3CSi$); 29.94, 29.98 (CH_3**C**H_2CH); 42.14, 42.26 (CH_2-2′); 64.31, 64.41 (CH_2-5′); 71.44, 71.85 (CH_3CH_2**C**H); 73.74, 73.94 (CH-3′); 87.10, 87.22 (CH-1′); 89.33, 89.49 (CH-4′); 111.21, 111.43 (C-5); 139.07, 139.15 (CH-6); 157.22 (C-2); 164.91, 164.93 (C-4).

HRMS (ESI^+): *m/z* calcd for $C_{24}H_{48}O_5N_3Si_2$ [M + H^+] 514.31270; found: 514.31187.

3’, 5’-Di(*tert*-butyldimethylsilyl)-5-(1-oxopropyl)- 2′-deoxycytidine (6)

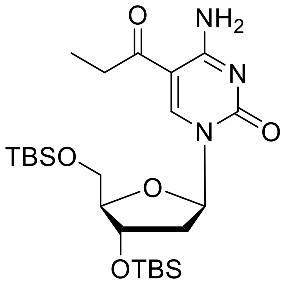

The compound was prepared according to **GP2,** starting from **4** (580 mg, 1.13 mmol). After the reaction was finished, the mixture was diluted with water (10 mL) and extracted with EtOAc (2 x 20 mL). The combined organic layers were washed with water (10 mL), brine (10 mL) and dried over Na_2SO_4. The solvents were evaporated and the crude product was purified by FLC (30 to 100% EtOAc in cHex followed by 0 to 20% MeOH in EtOAc, diol column) aquiring the pure product as a white foam (205 mg, 35%).

NMR: ^{1}H NMR (401.0 MHz, CD_3OD): 0.10, 0.11, 0.13, 0.14 (4 × s, 4 × 3H, CH_3Si); 0.89, 0.94 (2 × s, 2 × 9H, $(CH_3)_3CSi$); 1.14 (t, 3H, J_{vic} = 7.3, C**H₃**CH_2CO); 2.14 (ddd, 1H, J_{gem} = 13.4, $J_{2'b,1'}$ = 7.6, $J_{2'b,3'}$ = 5.7, H-2′b); 2.57 (ddd, 1H, J_{gem} = 13.6, $J_{2'a,1'}$ = 5.9, $J_{2'a,3'}$ = 2.0, H-2′a); 2.80 – 2.89 (m, 2H, CH_3C**H**$_2$CO); 3.86 (dd, 1H, J_{gem} = 11.5, $J_{5'b,4'}$ = 4.0, H-5′b); 3.91 (dd, 1H, J_{gem} = 11.5, $J_{5'a,4'}$ = 4.0, H-5′a); 4.14 (td, 1H, $J_{4',5'}$ = 4.0, $J_{4',3'}$ = 2.0, H-4′); 4.45 (dt, 1H, $J_{3',2'}$ = 5.7, 2.0, $J_{3',4'}$ = 2.0, H-3′); 6.12 (dd, 1H, $J_{1',2'}$ = 7.6, 5.9, H-1′); 8.71 (s, 1H, H-6). ^{13}C NMR (100.8 MHz, CD_3OD): -5.23, -5.21, -4.66, -4.55 (CH_3Si); 8.37 (**C**H_3CH_2CO); 18.89, 19.24 ($(CH_3)_3$**C**Si); 26.25, 26.39 ((**C**$H_3)_3CSi$); 31.85 (CH_3**C**H_2CO); 43.48 (CH_2-2′); 64.79 (CH_2-5′); 74.76 (CH-3′); 89.85 (CH-1′); 90.75 (CH-4′); 104.68 (C-5); 149.35 (CH-6); 156.01 (C-2); 165.26 (C-4); 199.59 (CH_3CH_2**C**O).

HRMS (ESI^+): *m/z* calcd for $C_{24}H_{46}O_5N_3Si_2$ [M + H^+] 512.29705; found: 512.29636.

General procedure for TBS deprotection (GP3)

TBS-protected starting material was dissolved in THF (c = 0.15 M) and Et_3N*3HF was added (3.4 equiv., 1.7 equiv. per TBS group). The reaction was stirred for 18 hours at 23 °C and monitored by TLC. The reaction was stopped by addition of saturated solution of $NaHCO_3$ until the gas generation stopped. The solvents were evaporated and the crude mixture was separated by FLC, in some cases followed by RP-HPLC (**dU^{f}**, **dU^{hp}**).

5-Formyl-2′-deoxyuridine (dU^{f})

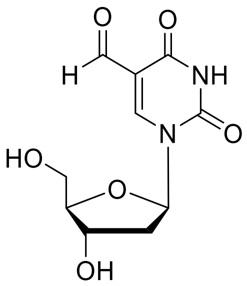

The compound was synthesized using **GP3**, starting from **2** (300 mg, 0.619 mmol). Separation of the crude mixture by FLC (0 to 15% MeOH in DCM) followed by purification using HPLC (0 to 30% MeOH in H_2O) provided the pure product as a white solid (61 mg, 39%). NMR data were in accordance with the literature.[16]

5-(1-Hydroxypropyl)- 2′-deoxyuridine (dU^{hp})

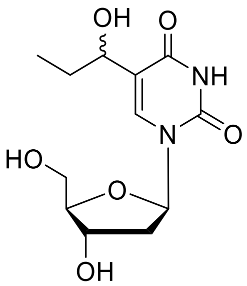

The compound was synthesized using **GP3**, starting from **3** (600 mg, 0.858 mmol). Separation of the crude mixture by FLC (0 to 15% MeOH in DCM) provided the pure product as a white solid (295 mg, 88%, epimeric ratio 5:4). The epimers were separated by HPLC (0 to 40% MeOH in H_2O, 15 mL/min, Phenomenex Kinetex EVO C18) (see Section 3, Figure S15).

NMR: ^{1}H NMR (500.2 MHz, CD_3OD): 0.94, 0.95 (2 × t, 2 × 3H, J_{vic} = 7.4, C**H₃**CH₂CHOH); 1.56 – 1.66, 1.74 – 1.84 (2 × m, 2 × 2H, CH₃C**H₂**CHOH); 2.18 – 2.32 (m, 4H, H-2′); 3.72, 3.73 (2 × dd, 2 × 1H, J_{gem} = 11.9, $J_{5'b,4'}$ = 4.0, H-5′b); 3.78, 3.79 (2 × dd, 2 × 1H, J_{gem} = 11.9, $J_{5'a,4'}$ = 4.2, H-5′a); 3.91 – 3.95 (m, 2H, H-4′); 4.38 – 4.42 (m, 2H, H-3′); 4.510, 4.512 (2 × dd, 2 × 1H, J_{vic} = 7.8, 4.7, CH₃CH₂C**H**OH); 6.30, 6.31 (2 × t, 2 × 1H, $J_{1',2'}$ = 6.5, H-1′); 7.90, 7.92 (2 × d, 2 × 1H, ^{4}J = 0.9, H-6). ^{13}C NMR (125.8 MHz, CD_3OD): 10.23, 10.28 (**C**H₃CH₂CHOH); 30.16, 30.23 (CH₃**C**H₂CHOH); 41.13, 42.28 (CH₂-2′); 62.94, 62.97 (CH₂-5′); 69.22, 69.24 (CH₃CH₂**C**HOH); 72.37 (CH-3′); 86.49, 86.61 (CH-1′); 88.84, 88.89 (CH-4′); 118.54, 118.63 (C-5); 138.22 (CH-6); 152.09 (C-2); 164.92, 164.94 (C-4).

HRMS (ESI$^+$): *m/z* calcd for $C_{12}H_{18}O_6N_2Na$ [M + Na$^+$] 309.10571; found: 309.10538.

5-(1-Oxopropyl)- 2′-deoxyuridine (dU^{pp})

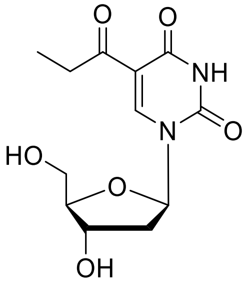

The compound was synthesized using **GP3**, starting from **4** (327 mg, 0.71 mmol). Separation of the crude mixture by FLC (0 to 20% MeOH in DCM) provided the pure product as a white solid (129 mg, 71%).

NMR: ^{1}H NMR (500.2 MHz, CD_3OD): 1.08 (t, 3H, J_{vic} = 7.3, C**H₃**CH₂CO); 2.27 (ddd, 1H, J_{gem} = 13.7, $J_{2'b,1'}$ = 6.6, $J_{2'b,3'}$ = 6.2, H-2′b); 2.38 (ddd, 1H, J_{gem} = 13.7, $J_{2'a,1'}$ = 6.3, $J_{2'a,3'}$ = 3.8, H-2′a); 2.98 (q, 2H, J_{vic} = 7.3, C**H₃**CH₂CO); 3.74 (dd, 1H, J_{gem} = 11.9, $J_{5'b,4'}$ = 3.7, H-5′b); 3.81 (dd, 1H, J_{gem} = 11.9, $J_{5'a,4'}$ = 3.4, H-5′a); 3.98 (dt, 1H, $J_{4',5'}$ = 3.7, 3.4, $J_{4',3'}$ = 3.4, H-4′); 4.40 (ddd, 1H, $J_{3',2'}$ = 6.2, 3.8, $J_{3',4'}$ = 3.4, H-3′); 6.25 (dd, 1H, $J_{1',2'}$ = 6.6, 6.3, H-1′); 8.84 (s, 1H, H-6). ^{13}C NMR (125.8 MHz, CD_3OD): 8.30 (**C**H₃CH₂CO); 36.37 (CH₃**C**H₂CO); 41.97 (CH₂-2′); 62.65 (CH₂-5′); 72.16 (CH-3′); 87.73 (CH-1′); 89.43 (CH-4′); 113.31 (C-5); 148.60 (CH-6); 151.46 (C-2); 163.13 (C-4); 199.11 (CH₃CH₂**C**O).

HRMS (ESI$^+$): *m/z* calcd for $C_{12}H_{16}O_6N_2Na$ [M + Na$^+$] 307.09006; found: 307.08992.

5-(1-Hydroxypropyl)- 2′-deoxycytidine (dC^{hp})

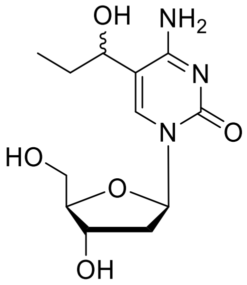

The compound was synthesized using **GP3**, starting from **5** (690 mg, 1.34 mmol). Separation of the crude mixture by FLC (0 to 20% MeOH in DCM) provided the product as a white solid (294 mg, 77%, epimeric ratio 5:4). The epimers were separated by HPLC using a chiral column (30 to 40% isopropanol in MTBE with 0.5% Et_2NH, over 16 min, 20 mL/min, DAICEL Chiralpak IE column) (see Section 3, Figure S16).

NMR: ^{1}H NMR (600.1 MHz, CD_3OD): 0.93, 0.94 (2 × t, 2 × 3H, J_{vic} = 7.4, C**H₃**CH₂CHOH); 1.72 – 1.80 (m, 4H, CH₃C**H₂**CHOH); 2.10 – 2.16 (m, 2H, H-2′b); 2.34 – 2.38 (m, 2H, H-2′a); 3.73, 3.74 (2 × dd, 2 × 1H, J_{gem} = 12.0, $J_{5'b,4'}$ = 3.7, H-5′b); 3.809, 3.810 (2 × dd, 2 × 1H, J_{gem} = 12.0, $J_{5'a,4'}$ = 3.2, H-5′a); 3.93 – 3.95 (m, 2H, H-4′); 4.35 – 4.41 (m, 4H, H-3′, CH₃CH₂C**H**OH); 6.25, 6.26 (2 × dd, 2 × 1H, $J_{1',2'}$ = 6.4, 3.4, H-1′); 8.018, 8.021 (2 × d, 2 × 1H, ^4J = 0.7, H-6). ^{13}C NMR (150.9 MHz, CD_3OD): 10.59, 10.62 (**C**H₃CH₂CHOH); 29.65 (CH₃**C**H₂CHOH); 42.23, 42.25 (CH₂-2′); 62.63, 62.68 (CH₂-5′); 71.67, 71.84 (CH₃CH₂**C**HOH); 71.90, 71.96 (CH-3′); 87.59, 87.60 (CH-1′); 88.91 (CH-4′); 110.38, 110.51 (C-5); 139.88, 139.90 (CH-6); 157.79, 157.80 (C-2); 166.04, 166.07 (C-4).

HRMS (ESI$^+$): *m/z* calcd for $C_{12}H_{20}O_5N_3$ [M + H$^+$] 286.13975; found: 286.13951.

5-(1-Oxopropyl)- 2′-deoxycytidine (dC^pp^)

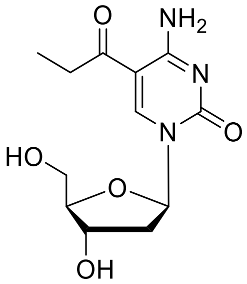

The compound was synthesized using **GP3**, starting from **6** (205 mg, 0.401 mmol). Separation of the crude mixture by FLC (0 to 20% MeOH in DCM) provided the product as a white powder (103 mg, 90%).

NMR: ^{1}H NMR (600.1 MHz, CD_3OD): 1.13 (t, 3H, J_{vic} = 7.3, C**H**$_3$CH$_2$CO); 2.27 (ddd, 1H, J_{gem} = 13.7, $J_{2'b,3'}$ = 6.3, $J_{2'b,1'}$ = 5.1, H-2′b); 2.49 (ddd, 1H, J_{gem} = 13.7, $J_{2'a,1'}$ = 6.5, $J_{2'a,3'}$ = 5.2, H-2′a); 2.84, 2.88 (2 × dq, 2 × 1H, J_{gem} = 17.2, J_{vic} = 7.3, C**H**$_3$CH$_2$CO); 3.79, 3.92 (2 × dd, 2 × 1H, J_{gem} = 12.0, $J_{5',4'}$ = 2.8, H-5′); 4.01 (dt, 1H, $J_{4',3'}$ = 4.5, $J_{4',5'}$ = 2.8, H-4′); 4.42 (ddd, 1H, $J_{3',2'}$ = 6.3, 5.2, $J_{3',4'}$ = 4.5, H-3′); 6.23 (dd, 1H, $J_{1',2'}$ = 6.5, 5.1, H-1′); 9.31 (s, 1H, H-6). ^{13}C NMR (150.9 MHz, CD_3OD): 8.79 (**C**H$_3$CH$_2$CO); 32.10 (CH$_3$**C**H$_2$CO); 42.95 (CH$_2$-2′); 61.75 (CH$_2$-5′); 70.94 (CH-3′); 88.53 (CH-1′); 89.30 (CH-4′); 104.83 (C-5); 150.61 (CH-6); 156.22 (C-2); 165.34 (C-4); 200.66 (CH$_3$CH$_2$**C**O).

HRMS (ESI$^+$): *m/z* calcd for $C_{12}H_{17}O_5N_3Na$ [M + Na$^+$] 306.10604; found: 306.10589.

1.4. Synthesis of nucleoside triphosphates

General procedure for triphosphorylation (GP4)

Starting material (SM, 1 equiv.) was dried in a flask overnight under high vacuum, optionally with Proton Sponge (1.5 – 6 equiv., see Table S1). Next morning, the flask was flushed with argon, $PO(OMe)_3$ (c = 0.1 – 0.5 M, see Table S1) was added and the suspension was stirred for 10 min at 23 °C, subsequently cooled down using an ice bath (0 °C, for some compounds -5 °C, see Table S1) and after 5 min, $POCl_3$ (1.2 equiv.) was injected slowly in one portion. The reaction was stirred at the designated temperature (0 °C, for some compounds -5 °C, see Table S1) for 2 – 6 hours (first step) followed by addition of Bu_3N (5 equiv.) and an ice-cold solution of

$(Bu_3NH)_2H_2P_2O_7$ (0.5 M in acetonitrile, 4 equiv.) respectively and the reaction was stirred for another 90 minutes at the designated temperature (second step). Finally, a solution of triethylammonium bicarbonate (TEAB, 1 M, 2 mL per 0.5 mL of $PO(OMe)_3$ used) was added and the reaction was stirred for additional 5 min and then allowed to reach 23 °C over 15 min. The solvents were evaporated and the remaining liquid was co-distilled with water (3 x 10 mL). The crude mixture was separated by HPLC using solvent system A (0.1 M TEAB in H_2O) and B (0.1 M TEAB in 50% MeOH). Purified nucleoside triphosphate was optionally converted to sodium salt by passing through a column of Dowex 50WX8 (Na^+ form, see Table S1). Lyophilization from water gave pure product **dN^{X}TP** as a white powder.

Table S1. **Reaction conditions of triphosphorylation reaction in details.**

Nucleoside triphosphate	SM concentration in $PO(OMe)_3$ [M]	Proton sponge [equiv.]	First step [h]	Reaction temperature [°C]	Salt form
**$dU^{et}TP$**	0.2	0	4	0	Na^+
**$dU^{ac}TP$**	0.2	1.5	4	0	Na^+
**$dC^{et}TP$**	0.1	0	2	0	Na^+
**$dC^{ac}TP$**	0.1	0	2	0	Na^+
**$dU^{Rhe}TP$**	0.2	1.5	6	-5	Na^+
**$dU^{She}TP$**	0.2	1.5	6	-5	Na^+
**$dC^{Rhe}TP$**	0.2	0	5.5	-5	Et_3NH^+
**$dC^{She}TP$**	0.2	0	4	-5	Et_3NH^+
**$dU^{pr}TP$**	0.2	0	4	0	Na^+
**$dU^{pp}TP$**	0.2	1.6	4	0	Na^+
**$dC^{pr}TP$**	0.1	0	2	0	Na^+
**$dC^{pp}TP$**	0.1	0	2	0	Na^+
**$dU^{Rhp}TP$**	0.2	4	5	-5	Et_3NH^+
**$dU^{Shp}TP$**	0.2	5	5	-5	Et_3NH^+
**$dC^{Rhp}TP$**	0.2	0	2.5	-5	Et_3NH^+
**$dC^{Shp}TP$**	0.2	0	2.5	-5	Et_3NH^+
**$dU^{f}TP$**	0.18	1.5	6	0	Et_3NH^+
**$dC^{f}TP$**	0.18	0	2	0	Et_3NH^+
**$dU^{I}TP$**	0.26	0	6	0	Et_3NH^+
**$dC^{I}TP$**	0.26	0	6	0	Et_3NH^+
**$dC^{E}TP$**	0.5	0	2	0	Et_3NH^+

Note: SM = starting material

5-Ethyl-2′-deoxyuridine-5′-*O*-triphosphate, sodium salt (dU^et^TP)

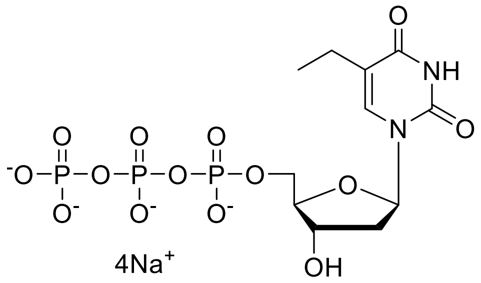

dU^et^TP was synthesized from its corresponding nucleoside **dU^et^** (50.5 mg, 0.197 mmol) using **GP4**. $PO(OMe)_3$ (1 mL, 0.2 M); first step 4 hours at 0 °C, second step at 0 °C. HPLC (0 to 50% B in A, 80 min, Phenomenex Omega Luna). The pure product was converted to sodium form followed by lyophilization affording **dU^et^TP** as a white solid (17 mg, 15%).

NMR: ^{1}H NMR (500.0 MHz, D_2O, ref(*t*BuOH) = 1.24 ppm): 1.09 (t, 3H, J_{vic} = 7.5, C**H$_3$**CH$_2$); 2.31 – 2.44 (m, 4H, H-2′b, CH$_3$C**H$_2$**); 4.15 – 4.26 (bm, 3H, H-4′,5′); 4.67 (dt, 1H, $J_{3',2'}$ = 6.0, 3.0, $J_{3',4'}$ = 3.0, H-3′); 6.35 (dd, 1H, $J_{1',2'}$ = 7.9, 6.2, H-1′); 7.69 (t, 1H, ^4J = 1.1, H-6). ^{13}C NMR (125.7 MHz, D_2O, ref(*t*BuOH) = 32.43 ppm): 15.44 (**C**H$_3$CH$_2$); 22.66 (CH$_3$**C**H$_2$); 41.33 (CH$_2$-2′); 68.32 (d, $J_{C,P}$ = 5.6, CH$_2$-5′); 73.77 (CH-3′); 87.91 (CH-1′); 88.39 (d, $J_{C,P}$ = 9.1, CH-4′); 120.38 (C-5); 139.76 (CH-6); 154.55 (C-2); 169.04 (C-4). ^{31}P{^{1}H} NMR (202.4 MHz, D_2O): -22.99 (t, J = 19.9, P$_\beta$); -11.60 (d, J = 19.9, P$_\alpha$); -9.73 (bs, P$_\gamma$).

HRMS (ESI$^-$): *m/z* calcd for $C_{11}H_{18}O_{14}N_2P_3$ [M - H$^+$] 494.99764; found: 494.99751.

5-Acetyl-2′-deoxyuridine-5′-*O*-triphosphate, sodium salt (dU^ac^TP)

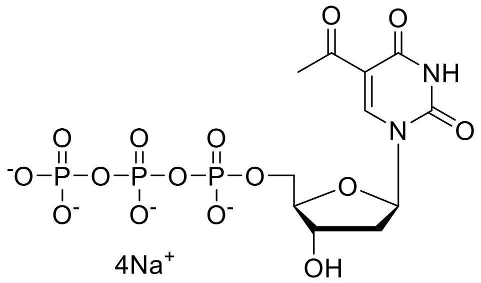

Compound **dU^ac^TP** was synthesized from its corresponding nucleoside **dU^ac^** (40.5 mg, 0.150 mmol) using **GP4**. Proton Sponge (48 mg, 1.5 equiv.) was used. $PO(OMe)_3$ (0.75 mL, 0.2 M); first step for 4 hours at 0 °C, second step at 0 °C. HPLC (0 to 50% B in A, 80 min, Phenomenex Omega Luna). The pure product was converted to sodium form followed by

lyophilization affording **dU^{ac}TP** as a white solid (14 mg, 16%). NMR are in accordance with literature.[17]

NMR: ^{1}H NMR (500.0 MHz, D_2O, ref(*t*BuOH) = 1.24 ppm): 2.44 (ddd, 1H, J_{gem} = 14.2, $J_{2'b,1'}$ = 7.1, $J_{2'b,3'}$ = 6.1, H-2′b); 2.51 (ddd, 1H, J_{gem} = 14.2, $J_{2'a,1'}$ = 6.3, $J_{2'a,3'}$ = 3.4, H-2′a); 2.55 (s, 3H, CH_3CO); 4.20 – 4.27 (bm, 2H, H-5′); 4.30 (qd, 1H, $J_{4',3'}$ = $J_{4',5'}$ = 3.4, $J_{H,P}$ = 1.8, H-4′); 4.66 (dt, 1H, $J_{3',2'}$ = 6.1, 3.4, $J_{3',4'}$ = 3.4, H-3′); 6.26 (dd, 1H, $J_{1',2'}$ = 7.1, 6.3, H-1′); 8.66 (s, 1H, H-6).

^{13}C NMR (125.7 MHz, D_2O, ref(*t*BuOH) = 32.43 ppm): 31.36 (**C**H_3CO); 42.32 (CH_2-2′); 68.24 (d, $J_{C,P}$ = 5.7, CH_2-5′); 73.65 (CH-3′); 89.20 (d, $J_{C,P}$ = 8.8, CH-4′); 89.89 (CH-1′); 115.00 (C-5); 151.44 (CH-6); 153.44 (C-2); 165.44 (C-4); 200.76 (CH_3**C**O). ^{31}P{^{1}H} NMR (202.4 MHz, D_2O): -23.01 (t, *J* = 19.8, P_β); -11.57 (d, *J* = 19.8, P_α); -10.07 (bd, *J* = 19.8, P_γ).

HRMS (ESI$^-$): *m/z* calcd for $C_{11}H_{16}O_{15}N_2P_3$ [M - H$^+$] 508.97690; found: 508.97674.

5-Ethyl-2′-deoxycytidine-5′-*O*-triphosphate, sodium salt (dC^{et}TP)

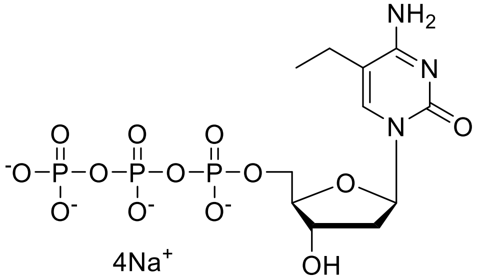

Compound **dC^{et}TP** was synthesized from its corresponding nucleoside **dC^{et}** (50 mg, 0.195 mmol) using **GP4**. $PO(OMe)_3$ (2 mL, 0.1 M); first step 2 hours at 0 °C, second step at 0 °C. HPLC (0 to 50% B in A, 80 min, Phenomenex Kinetex EVO C18). The pure product was converted to sodium form followed by lyophilization affording **dC^{et}TP** as a white solid (13.5 mg, 12%).

NMR: ^{1}H NMR (500.0 MHz, D_2O, ref(*t*BuOH) = 1.24 ppm): 1.15 (t, 3H, J_{vic} = 7.5, C**H$_3$**CH_2); 2.30 – 2.43 (m, 4H, H-2′, CH_3C**H$_2$**); 4.17 – 4.30 (m, 3H, H-4′,5′); 4.68 (dt, 1H, $J_{3',2'}$ = 6.3, 3.5, $J_{3',4'}$ = 3.5, H-3′); 6.34 (dd, 1H, $J_{1',2'}$ = 7.2, 6.4, H-1′); 7.73 (t, 1H, ^4J = 1.0, H-6). ^{13}C NMR (125.7 MHz, D_2O, ref(*t*BuOH) = 32.43 ppm): 14.87 (**C**H_3CH_2); 22.90 (CH_3**C**H_2); 41.98 (CH_2-2′); 68.02 (d, $J_{C,P}$ = 5.1, CH_2-5′); 73.31 (CH-3′); 88.42 (d, $J_{C,P}$ = 9.3, CH-4′); 88.49 (CH-1′); 113.61 (C-5); 140.72

(CH-6); 160.21 (C-2); 168.25 (C-4). $^{31}P\{^1H\}$ NMR (202.4 MHz, D_2O): -22.41 (bt, J = 19.6, P_β); -11.48 (d, J = 19.6, P_α); -6.62 (br, P_γ).

HRMS (ESI^-): *m/z* calcd for $C_{11}H_{19}O_{13}N_3P_3$ [M - H^+] 494.01362; found: 494.01352.

5-Acetyl-2′-deoxycytidine-5′-*O*-triphosphate, sodium salt (dC^{ac}TP)

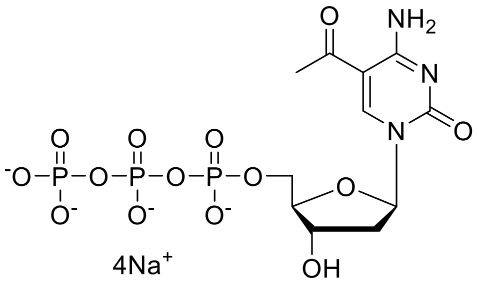

Compound **dC^{ac}TP** was synthesized from its corresponding nucleoside **dC^{ac}** (26 mg, 0.098 mmol) using **GP4**. $PO(OMe)_3$ (1 mL, 0.1 M); first step 2 hours at 0 °C, second step at 0 °C. HPLC (0 to 50% B in A, 80 min, Phenomenex Kinetex EVO C18). The pure product was converted to sodium form followed by lyophilization affording **dC^{ac}TP** as a white solid (13.3 mg, 23%).

NMR: ^1H NMR (500.0 MHz, D_2O, ref(*t*BuOH) = 1.24 ppm): 2.38 (dt, 1H, J_{gem} = 14.2, $J_{2'b,1'}$ = $J_{2'b,3'}$ = 6.4, H-2′b); 2.57 (ddd, 1H, J_{gem} = 14.2, $J_{2'a,1'}$ = 6.4, $J_{2'a,3'}$ = 3.7, H-2′a); 2.58 (s, 3H, CH_3CO); 4.23 – 4.32 (m, 2H, H-5′); 4.34 (m, 1H, H-4′); 4.65 (ddd, 1H, $J_{3',2'}$ = 6.4, 3.7, $J_{3',4'}$ = 3.0, H-3′); 6.24 (t, 1H, $J_{1',2'}$ = 6.4, H-1′); 8.83 (t, 1H, ^4J = 1.0, H-6). ^{13}C NMR (125.7 MHz, D_2O, ref(*t*BuOH) = 32.43 ppm): 29.08 (**C**H_3CO); 43.18 (CH_2-2′); 68.05 (d, $J_{C,P}$ = 5.6, CH_2-5′); 73.32 (CH-3′); 89.37 (d, $J_{C,P}$ = 8.9, CH-4′); 90.42 (CH-1′); 107.75 (C-5); 152.83 (CH-6); 158.48 (C-2); 166.42 (C-4); 202.24 (CH_3**C**O). $^{31}P\{^1H\}$ NMR (202.4 MHz, D_2O): -23.02 (bt, J = 19.8, P_β); -11.66 (d, J = 19.8, P_α); -9.55 (br, P_γ).

HRMS (ESI^-): *m/z* calcd for $C_{11}H_{17}O_{14}N_3P_3$ [M - H^+] 507.99288; found: 507.99319.

5-(R-1-Hydroxyethyl) -2′-deoxyuridine-5′-O-triphosphate, sodium salt (dU^{Rhe}TP)

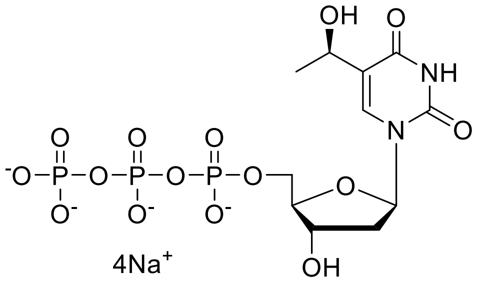

Compound **dU^{Rhe}TP** was synthesized from its corresponding nucleoside **dU^{Rhe}** (22 mg, 0.081 mmol) using **GP4**. Proton Sponge (26 mg, 1.5 equiv.) was used. $PO(OMe)_3$ (0.4 mL, 0.2 M); first step 6 hours at -5 °C, second step at -5 °C. HPLC (0 to 40% B in A, 80 min, Phenomenex Kinetex EVO C18). The pure product was converted to sodium form followed by lyophilization affording **dU^{Rhe}TP** as a white solid (8.6 mg, 18%).

NMR: ^{1}H NMR (500.0 MHz, D_2O, ref(tBuOH) = 1.24 ppm): 1.45 (d, 3H, J_{vic} = 6.6, C**H**$_3$CHOH); 2.36 – 2.46 (m, 2H, H-2′); 4.19 – 4.24 (m, 2H, H-4′,5′b); 4.29 (ddd, 1H, J_{gem} = 12.3, $J_{H,P}$ = 6.3, $J_{5'a,4'}$ = 4.1, H-5′a); 4.71 (ddd, 1H, $J_{3',2'}$ = 5.8, 4.7, $J_{3',4'}$ = 3.4, H-3′); 4.79 (qd, 1H, J_{vic} = 6.6, ^{4}J = 0.9, CH$_3$C**H**OH); 6.33 (t, 1H, $J_{1',2'}$ = 6.7, H-1′); 7.88 (d, 1H, ^{4}J = 0.9, H-6). ^{13}C NMR (125.7 MHz, D_2O, ref(tBuOH) = 32.43 ppm): 23.96 (**C**H$_3$CHOH); 41.61 (CH$_2$-2′); 66.41 (CH$_3$**C**HOH); 67.94 (d, $J_{C,P}$ = 5.5, CH$_2$-5′); 73.12 (CH-3′); 88.26 (CH-1′); 88.62 (d, $J_{C,P}$ = 9.1, CH-4′); 121.18 (C-5); 140.01 (CH-6); 154.62 (C-2); 167.80 (C-4). ^{31}P{^{1}H} NMR (202.4 MHz, D_2O): -21.49 (bm, Pβ); -10.57 (d, J = 19.5, Pα); -5.43 (bm, Pγ).

HRMS (ESI$^-$): m/z calcd for $C_{11}H_{18}O_{15}N_2P_3$ [M - H$^+$] 510.99255; found: 510.99238.

5-(*S*-1-Hydroxyethyl) -2′-deoxyuridine-5′-*O*-triphosphate, sodium salt (dU^{She}TP)

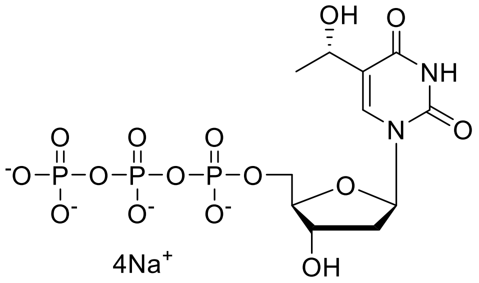

Compound **dU^{She}TP** was synthesized from its corresponding nucleoside **dU^{She}** (42 mg, 0.154 mmol) using **GP4**. Proton Sponge (49.6 mg, 1.5 equiv.) was used. $PO(OMe)_3$ (0.8 mL, 0.2 M); first step 6 hours at -5 °C, second step at -5 °C. HPLC (0 to 40% B in A, 80 min, Phenomenex Kinetex EVO C18). The pure product was converted to sodium form followed by lyophilization affording **dU^{She}TP** as a white solid (16.2 mg, 18%).

NMR: ^{1}H NMR (500.0 MHz, D_2O, ref(*t*BuOH) = 1.24 ppm): 1.44 (d, 3H, J_{vic} = 6.6, C**H**$_3$CHOH); 2.36 – 2.47 (m, 2H, H-2′); 4.18 – 4.23 (m, 2H, H-4′,5′b); 4.26 (ddd, 1H, J_{gem} = 12.3, $J_{H,P}$ = 6.2, $J_{5'a,4'}$ = 4.1, H-5′a); 4.70 (ddd, 1H, $J_{3',2'}$ = 5.7, 4.5, $J_{3',4'}$ = 3.2, H-3′); 4.78 (qd, 1H, J_{vic} = 6.6, ^4J = 0.9, CH$_3$C**H**OH); 6.34 (t, 1H, $J_{1',2'}$ = 6.7, H-1′); 7.88 (d, 1H, ^4J = 0.9, H-6). ^{13}C NMR (125.7 MHz, D_2O, ref(*t*BuOH) = 32.43 ppm): 24.11 (**C**H$_3$CHOH); 41.54 (CH$_2$-2′); 66.36 (CH$_3$**C**HOH); 68.07 (d, $J_{C,P}$ = 5.5, CH$_2$-5′); 73.34 (CH-3′); 88.21 (CH-1′); 88.52 (d, $J_{C,P}$ = 9.1, CH-4′); 121.40 (C-5); 139.83 (CH-6); 154.39 (C-2); 167.42 (C-4). ^{31}P{^{1}H} NMR (202.4 MHz, D_2O): -21.89 (bt, J = 19.2, P_β); -10.63 (d, J = 19.2, P_α); -7.71 (bs, P_γ).

HRMS (ESI$^-$): *m/z* calcd for $C_{11}H_{18}O_{15}N_2P_3$ [M - H$^+$] 510.99255; found: 510.99232.

5-(*R*-1-Hydroxyethyl)-2′-deoxycytidine-5′-*O*-triphosphate, tris(triethylammonium) salt (dC^{Rhe}TP)

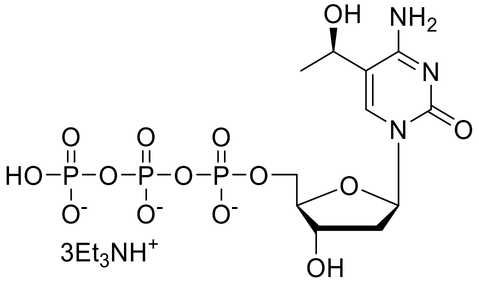

Compound **dC^{Rhe}TP** was synthesized from its corresponding nucleoside **dC^{Rhe}** (14 mg, 0.052 mmol) using **GP4**. $PO(OMe)_3$ (0.25 mL, 0.2 M); first step 5.5 hours at -5 °C, second step at

-5 °C. The crude mixture was purified by HPLC (0 to 40% B in A, 80 min, Phenomenex Kinetex EVO C18), followed by POROS HQ 50 (0 to 100% 400 mM TEAB in H_2O, 120 min). Lyophilization gave the product **dC^{Rhe}TP** as a white solid (4.3 mg, 10%).

NMR: ^{1}H NMR (600.1 MHz, D_2O, ref(*t*BuOH) = 1.24 ppm): 1.27 (t, 27H, J_{vic} = 7.3, C**H$_3$**CH$_2$N); 1.50 (d, 3H, J_{vic} = 6.6, C**H$_3$**CHOH); 2.32 (ddd, 1H, J_{gem} = 14.1, $J_{2'b,1'}$ = 7.4, $J_{2'b,3'}$ = 6.4, H-2′b); 2.42 (ddd, 1H, J_{gem} = 14.1, $J_{2'a,1'}$ = 6.2, $J_{2'a,3'}$ = 3.6, H-2′b); 3.20 (q, 18H, J_{vic} = 7.3, CH$_3$C**H$_2$**N); 4.18 – 4.267 (m, 3H, H-4′,5′); 4.65 (m, 1H, H-3′); 4.84 (qd, 1H, J_{vic} = 6.6, ^{4}J = 0.6, CH$_3$C**H**OH); 6.32 (dd, 1H, $J_{1',2'}$ = 7.4, 6.2, H-1′); 7.89 (d, 1H, ^{4}J = 0.6, H-6). ^{13}C NMR (150.9 MHz, D_2O, ref(*t*BuOH) = 32.43 ppm): 11.06 (**C**H$_3$CH$_2$N); 23.53 (**C**H$_3$CHOH); 42.31 (CH$_2$-2′); 49.50 (CH$_3$**C**H$_2$N); 67.43 (CH$_3$**C**HOH); 68.17 (d, $J_{C,P}$ = 5.7, CH$_2$-5′); 73.60 (CH-3′); 88.56 (d, $J_{C,P}$ = 9.1, CH-4′); 88.94 (CH-1′); 113.84 (C-5); 140.88 (CH-6); 159.84 (C-2); 167.20 (C-4). ^{31}P{^{1}H} NMR (202.5 MHz, D_2O): -22.57 (dd, J = 20.0, 19.6, P_β); -10.94 (d, J = 20.0, P_α); -9.57 (bd, J = 19.6, P_γ).

HRMS (ESI$^-$): *m/z* calcd for $C_{11}H_{19}O_{14}N_3P_3$ [M - H$^+$] 510.00853; found: 510.00840.

5-(*S*-1-Hydroxyethyl)-2′-deoxycytidine-5′-*O*-triphosphate, tris(triethylammonium) salt (dC^{She}TP)

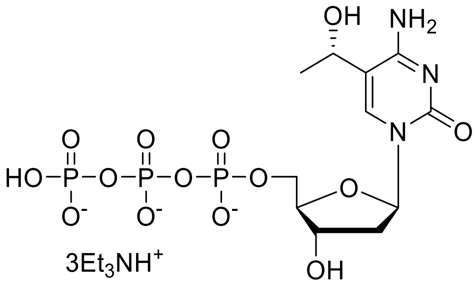

Compound **dC^{She}TP** was synthesized from its corresponding nucleoside **dC^{She}** (25 mg, 0.092 mmol) using **GP4**. PO(OMe)$_3$ (0.45 mL, 0.2 M); first step 4 hours at -5 °C, second step at -5 °C. The crude mixture was purified by HPLC (0 to 40% B in A, 80 min, Phenomenex Kinetex EVO C18). Lyophilization gave the product **dC^{She}TP** as a white solid (5.6 mg, 8%).

NMR: ^{1}H NMR (600.1 MHz, D_2O, ref(*t*BuOH) = 1.24 ppm): 1.28 (t, 27H, J_{vic} = 7.3, C**H$_3$**CH$_2$N); 1.50 (d, 3H, J_{vic} = 6.6, C**H$_3$**CHOH); 2.33 (ddd, 1H, J_{gem} = 14.0, $J_{2'b,1'}$ = 7.2, $J_{2'b,3'}$ = 6.2, H-2′b); 2.44 (ddd, 1H, J_{gem} = 14.0, $J_{2'a,1'}$ = 6.2, $J_{2'a,3'}$ = 3.5, H-2′b); 3.20 (q, 18H, J_{vic} = 7.3, CH$_3$C**H$_2$**N);

4.18 – 4.26 (m, 3H, H-4′,5′); 4.64 (ddd, 1H, $J_{3',2'}$ = 6.2, 3.5, $J_{3',4'}$ = 3.0, H-3′); 4.86 (qd, 1H, J_{vic} = 6.6, ^4J = 0.8, $CH_3C\mathbf{H}OH$); 6.30 (dd, 1H, $J_{1',2'}$ = 7.2, 6.2, H-1′); 7.91 (d, 1H, ^4J = 0.8, H-6).
^{13}C NMR (150.9 MHz, D_2O, ref(*t*BuOH) = 32.43 ppm): 11.06 ($\mathbf{C}H_3CH_2N$); 23.69 ($\mathbf{C}H_3CHOH$); 42.40 (CH_2-2′); 49.50 ($CH_3\mathbf{C}H_2N$); 67.45 ($CH_3\mathbf{C}HOH$); 68.16 (d, $J_{C,P}$ = 5.6, CH_2-5′); 73.60 (CH-3′); 88.65 (d, $J_{C,P}$ = 9.1, CH-4′); 89.13 (CH-1′); 113.70 (C-5); 141.11 (CH-6); 159.02 (C-2); 166.57 (C-4). ^{31}P{^{1}H} NMR (202.5 MHz, D_2O): -22.65 (dd, *J* = 20.0, 19.6, P_β); -10.94 (d, *J* = 20.0, P_α); -10.17 (d, *J* = 19.6, P_γ).
HRMS (ESI$^-$): *m/z* calcd for $C_{11}H_{19}O_{14}N_3P_3$ [M - H$^+$] 510.00853; found: 510.00831.

5-Propyl-2′-deoxyuridine-5′-*O*-triphosphate, sodium salt (dU^{pr}TP)

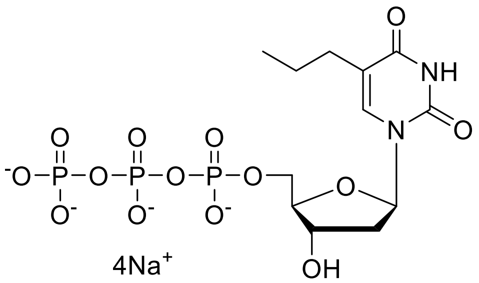

Compound **dU^{pr}TP** was synthesized from its corresponding nucleoside **dU^{pr}** (47.5 mg, 0.176 mmol) using **GP4**. $PO(OMe)_3$ (0.9 mL, 0.2 M); first step 4 hours at 0 °C, second step at 0 °C. HPLC (4 to 40% B in A, 80 min, Phenomenex Kinetex EVO C18). The pure product was converted to sodium form followed by lyophilization affording **dU^{pr}TP** as a white solid (25.4 mg, 24%).
NMR: ^{1}H NMR (500.0 MHz, D_2O, ref(*t*BuOH) = 1.24 ppm): 0.89 (t, 3H, J_{vic} = 7.4, $C\mathbf{H_3}CH_2CH_2$); 1.50 (hep, 2H, J_{vic} = 7.4, $CH_3C\mathbf{H_2}CH_2$); 2.27 – 2.44 (m, 4H, H-2′, $CH_3CH_2C\mathbf{H_2}$); 4.14 – 4.26 (bm, 3H, H-4′,5′); 4.67 (dt, 1H, $J_{3',2'}$ = 6.1, 3.1, $J_{3',4'}$ = 3.1, H-3′); 6.35 (dd, 1H, $J_{1',2'}$ = 7.8, 6.2, H-1′); 7.70 (t, 1H, ^4J = 0.8, H-6). ^{13}C NMR (125.7 MHz, D_2O, ref(*t*BuOH) = 32.43 ppm): 15.45 ($\mathbf{C}H_3CH_2CH_2$); 24.13 ($CH_3\mathbf{C}H_2CH_2$); 30.95 ($CH_3CH_2\mathbf{C}H_2$); 41.27 (CH_2-2′); 68.30 (d, $J_{C,P}$ = 6.0, CH_2-5′); 73.74 (CH-3′); 87.83 (CH-1′); 88.37 (d, $J_{C,P}$ = 9.2, CH-4′); 118.59 (C-5); 140.50 (CH-6); 154.56 (C-2); 169.11 (C-4). ^{31}P{^{1}H} NMR (202.4 MHz, D_2O): -22.91 (t, *J* = 19.9, P_β); -11.63 (d, *J* = 19.9, P_α); -9.53 (bs, P_γ).
HRMS (ESI$^-$): *m/z* calcd for $C_{12}H_{20}O_{14}N_2P_3$ [M - H$^+$] 509.01329; found: 509.01310.

5-(1-Oxopropyl)- 2′-deoxyuridine-5′-*O*-triphosphate, sodium salt (dU^{pp}TP)

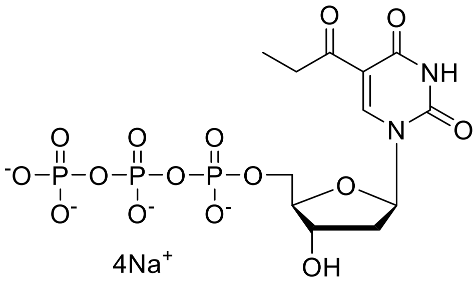

Compound **dU^{pp}TP** was synthesized from its corresponding nucleoside **dU^{pp}** (50 mg, 0.176 mmol) using **GP4**. Proton Sponge (60 mg, 1.6 equiv.) was added. $PO(OMe)_3$ (0.9 mL, 0.2 M); first step 4 hours at 0 °C, second step at 0 °C. HPLC (4 to 40% B in A, 80 min, Phenomenex Kinetex EVO C18). The pure product was converted to sodium form followed by lyophilization affording **dU^{pp}TP** as a white solid (15.6 mg, 14%).

NMR: ^{1}H NMR (500.0 MHz, D_2O, ref(*t*BuOH) = 1.24 ppm): 1.09 (t, 3H, J_{vic} = 7.2, C**H**$_3$CH$_2$CO); 2.44 (ddd, 1H, J_{gem} = 14.2, $J_{2'b,1'}$ = 7.2, $J_{2'b,3'}$ = 6.1, H-2′b); 2.50 (ddd, 1H, J_{gem} = 14.2, $J_{2'a,1'}$ = 6.3, $J_{2'a,3'}$ = 3.5, H-2′a); 2.98 (q, 2H, J_{vic} = 7.2, CH$_3$C**H**$_2$CO); 4.22 (ddd, 1H, J_{gem} = 11.6, $J_{H,P}$ = 5.2, $J_{5'b,4'}$ = 3.5, H-5′b); 4.26 (ddd, 1H, J_{gem} = 11.6, $J_{H,P}$ = 6.8, $J_{5'a,4'}$ = 3.5, H-5′b); 4.30 (qd, 1H, $J_{4',3'}$ = $J_{4',5'}$ = 3.5, $J_{H,P}$ = 1.7, H-4′); 4.67 (dt, 1H, $J_{3',2'}$ = 6.1, 3.5, $J_{3',4'}$ = 3.5, H-3′); 6.27 (dd, 1H, $J_{1',2'}$ = 7.2, 6.3, H-1′); 8.63 (s, 1H, H-6). ^{13}C NMR (125.7 MHz, D_2O, ref(*t*BuOH) = 32.43 ppm): 10.43 (**C**H$_3$CH$_2$CO); 37.16 (CH$_3$**C**H$_2$CO); 42.19 (CH$_2$-2′); 68.26 (d, $J_{C,P}$ = 5.7, CH$_2$-5′); 73.66 (CH-3′); 89.14 (d, $J_{C,P}$ = 8.9, CH-4′); 89.78 (CH-1′); 114.94 (C-5); 150.71 (CH-6); 153.49 (C-2); 165.52 (C-4); 203.87 (CH$_3$CH$_2$**C**O). ^{31}P{^{1}H} NMR (202.4 MHz, D_2O): -23.00 (t, J = 19.8, P$_\beta$); -11.58 (d, J = 19.8, P$_\alpha$); -9.79 (bs, P$_\gamma$).

HRMS (ESI$^-$): *m/z* calcd for $C_{12}H_{18}O_{15}N_2P_3$ [M - H$^+$] 522.99255; found: 522.99250.

5-Propyl-2′-deoxycytidine-5′-*O*-triphosphate, sodium salt (dC^{pr}TP)

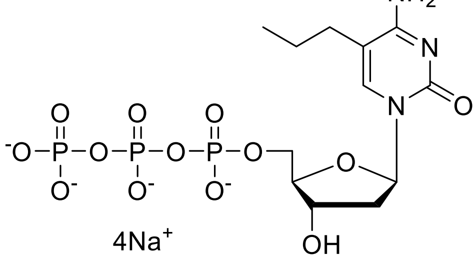

Compound **dC^{pr}TP** was synthesized from its corresponding nucleoside **dC^{pr}** (30 mg, 0.111 mmol) using **GP4**. $PO(OMe)_3$ (1.1 mL, 0.1 M); first step 2 hours at 0 °C, second step at 0 °C. HPLC (4 to 50% B in A, 80 min, Phenomenex Omega Luna). The pure product was converted to sodium form followed by lyophilization, affording **dC^{pr}TP** as a white solid (11.7 mg, 18%).

NMR: ^{1}H NMR (600.1 MHz, D_2O, ref(*t*BuOH) = 1.24 ppm): 0.91 (t, 3H, J_{vic} = 7.4, C**H3**CH_2CH_2); 1.54 (hep, 2H, J_{vic} = 7.4, CH_3C**H2**CH_2); 2.29 – 2.40 (m, 4H, H-2′, CH_3CH_2C**H2**); 4.16 – 4.21 (m, 2H, H-4′,5′b); 4.25 (m, 1H, H-5′a); 4.67 (dt, 1H, $J_{3',2'}$ = 6.3, 3.6, $J_{3',4'}$ = 3.6, H-3′); 6.34 (dd, 1H, $J_{1',2'}$ = 7.4, 6.3, H-1′); 7.72 (s, 1H, H-6). ^{13}C NMR (150.9 MHz, D_2O, ref(*t*BuOH) = 32.43 ppm): 15.52 (**C**$H_3CH_2CH_2$); 23.47 (CH_3**C**H_2CH_2); 31.31 (CH_3CH_2**C**H_2); 41.92 (CH_2-2′); 68.02 (d, $J_{C,P}$ = 5.5, CH_2-5′); 73.32 (CH-3′); 88.36 (d, $J_{C,P}$ = 9.3, CH-4′); 88.40 (CH-1′); 111.81 (C-5); 141.55 (CH-6); 160.19 (C-2); 168.34 (C-4). ^{31}P{^{1}H} NMR (202.4 MHz, D_2O): -22.46 (t, J = 20.0, P_b); -11.53 (d, J = 20.0, P_a); -6.98 (bs, P_γ).

HRMS (ESI$^-$): *m/z* calcd for $C_{12}H_{21}O_{13}N_3P_3$ [M - H$^+$] 508.02927; found: 508.02915.

5-(1-Oxopropyl)- 2′-deoxycytidine-5′-*O*-triphosphate, sodium salt (dC^{pp}TP)

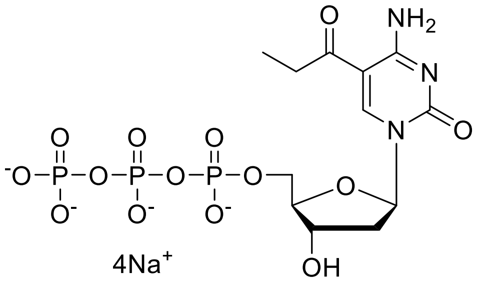

Compound **dC^{pp}TP** was synthesized from its corresponding nucleoside **dC^{pp}** (31 mg, 0.110 mmol) using **GP4**. $PO(OMe)_3$ (1.1 mL, 0.1 M); first step 2 hours at 0 °C, second step at 0 °C. HPLC (10 to 50% B in A, 80 min, Waters X-Bridge C18). The pure product was converted to sodium form followed by lyophilization affording **dC^{pp}TP** as a white solid (6.5 mg, 10%).

NMR: ^{1}H NMR (600.1 MHz, D_2O, ref(*t*BuOH) = 1.24 ppm): 1.13 (t, 3H, J_{vic} = 7.3, C**H3**CH_2CO); 2.38 (dt, 1H, J_{gem} = 14.2, $J_{2'b,1'}$ = $J_{2'b,3'}$ = 6.4, H-2′b); 2.55 (ddd, 1H, J_{gem} = 14.2, $J_{2'a,1'}$ = 6.4, $J_{2'a,3'}$ = 3.9, H-2′a); 2.96 – 3.06 (m, 2H, J_{vic} = 7.2, CH_3C**H2**CO); 4.26 (ddd, 1H, J_{gem} = 11.9, $J_{H,P}$ = 5.1, $J_{5'b,4'}$ = 3.3, H-5′b); 4.29 (ddd, 1H, J_{gem} = 11.9, $J_{H,P}$ = 6.5, $J_{5'a,4'}$ = 3.3, H-5′a); 4.33 (qd, 1H, $J_{4',3'}$ = $J_{4',5'}$ = 3.3, $J_{H,P}$ = 1.9, H-4′); 4.67 (ddd, 1H, $J_{3',2'}$ = 6.4, 3.9, $J_{3',4'}$ = 3.3, H-3′); 6.23 (t, 1H, $J_{1',2'}$ = 6.4, H-1′); 8.85 (s, 1H, H-6). ^{13}C NMR (150.9 MHz, D_2O, ref(*t*BuOH) = 32.43 ppm): 10.74

(**C**H_3CH_2CO); 34.22 (CH_3**C**H_2CO); 43.05 (CH_2-2′); 67.95 (d, $J_{C,P}$ = 5.3, CH_2-5′); 73.15 (CH-3′); 89.37 (d, $J_{C,P}$ = 8.8, CH-4′); 90.33 (CH-1′); 107.25 (C-5); 151.93 (CH-6); 158.48 (C-2); 166.60 (C-4); 205.12 (CH_3CH_2**C**O). $^{31}P\{^1H\}$ NMR (202.4 MHz, D_2O): -22.56 (t, J = 19.9, P_b); -11.62 (d, J = 19.9, P_a); -7.62 (bs, P_γ).

HRMS (ESI$^-$): *m/z* calcd for $C_{12}H_{19}O_{14}N_3P_3$ [M - H$^+$] 522.00853; found: 522.00841.

5-(*R*-1-Hydroxypropyl) -2′-deoxyuridine-5′-*O*-triphosphate, tris(triethylammonium) salt (dU^{Rhp}TP)

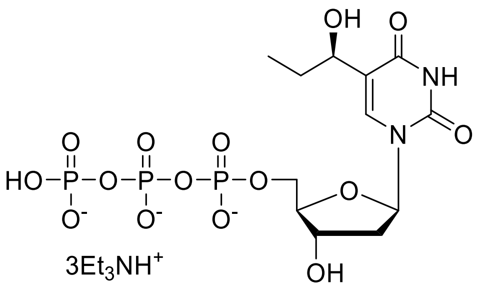

Compound **dU^{Rhp}TP** was synthesized from its corresponding nucleoside **dU^{Rhp}** (15 mg, 0.052 mmol) using **GP4**. Proton Sponge (44.9 mg, 4 equiv.) was added. $PO(OMe)_3$ (0.255 mL, 0.2 M); first step 5 hours at -5 °C, second step at -5 °C. The crude mixture was purified by HPLC (0 to 40% B in A, 80 min, Phenomenex Kinetex EVO C18). Lyophilization gave the product **dU^{Rhp}TP** as a white solid (7.3 mg, 17%).

NMR: ^1H NMR (600.1 MHz, D_2O, ref(*t*BuOH) = 1.24 ppm): 0.91 (t, 3H, J_{vic} = 7.4, C**H$_3$**CH_2CHOH); 1.28 (t, 27H, J_{vic} = 7.3, C**H$_3$**CH_2N); 1.68 – 1.88 (m, 2H, CH_3C**H$_2$**CHOH); 2.35 – 2.44 (m, 2H, H-2′); 3.20 (q, 18H, J_{vic} = 7.3, CH_3C**H$_2$**N); 4.17 – 4.28 (m, 3H, H-4′,5′); 4.54 (ddd, 1H, J_{vic} = 7.8, 5.7, ^4J = 0.7, CH_3CH_2C**H**OH); 4.68 (ddd, 1H, $J_{3′,2′}$ = 5.4, 4.3, $J_{3′,4′}$ = 2.8, H-3′); 6.34 (t, 1H, $J_{1′,2′}$ = 6.9, H-1′); 7.87 (d, 1H, ^4J = 0.7, H-6). ^{13}C NMR (150.9 MHz, D_2O, ref(*t*BuOH) = 32.43 ppm): 11.06 (**C**H_3CH_2N); 12.32 (**C**H_3CH_2CHOH); 31.03 (CH_3**C**H_2CHOH); 41.64 (CH_2-2′); 49.50 (CH_3**C**H_2N); 68.24 (d, $J_{C,P}$ = 5.6, CH_2-5′); 71.67 (CH_3CH_2**C**HOH); 73.58 (CH-3′); 88.28 (CH-1′); 88.58 (d, $J_{C,P}$ = 9.1, CH-4′); 120.02 (C-5); 140.74 (CH-6); 154.29 (C-2); 167.50 (C-4). $^{31}P\{^1H\}$ NMR (202.5 MHz, D_2O): -22.22 (bt, J = 19.8, P_β); -10.83 (d, J = 19.8, P_α); -8.89 (bs, P_γ).

HRMS (ESI$^-$): *m/z* calcd for $C_{12}H_{20}O_{15}N_2P_3$ [M - H$^+$] 525.00820; found: 525.00789.

5-(*S*-1-Hydroxypropyl) -2′-deoxyuridine-5′-*O*-triphosphate, tris(triethylammonium) salt (dU^{Shp}TP)

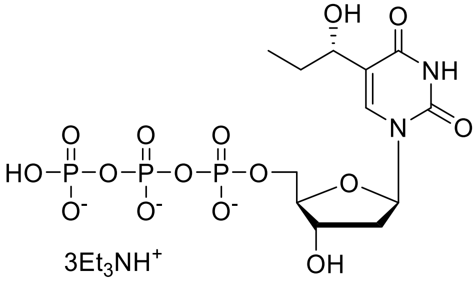

Compound **dU^{Shp}TP** was synthesized from its corresponding nucleoside **dU^{Shp}** (32 mg, 0.112 mmol) using **GP4**. Proton Sponge (120 mg, 5 equiv.) was added. PO(OMe)$_3$ (0.540 mL, 0.2 M); first step 5 hours at -5 °C, second step at -5 °C. The crude mixture was purified by HPLC (0 to 40% B in A, 80 min, Phenomenex Kinetex EVO C18). Lyophilization gave the product **dU^{Shp}TP** as a white solid (12.5 mg, 14%).

NMR: ^{1}H NMR (600.1 MHz, D$_2$O, ref(*t*BuOH) = 1.24 ppm): 0.92 (t, 3H, J_{vic} = 7.4, C**H$_3$**CH$_2$CHOH); 1.28 (t, 18H, J_{vic} = 7.3, C**H$_3$**CH$_2$N); 1.69, 1.83 (2 × m, 2 × 1H, CH$_3$C**H$_2$**CHOH); 2.36 – 2.45 (m, 2H, H-2′); 3.20 (q, 12H, J_{vic} = 7.3, CH$_3$C**H$_2$**N); 4.17 – 4.28 (m, 3H, H-4′,5′); 4.53 (ddd, 1H, J_{vic} = 7.9, 5.3, ^{4}J = 0.7, CH$_3$CH$_2$C**H**OH); 4.69 (ddd, 1H, $J_{3′,2′}$ = 5.4, 4.6, $J_{3′,4′}$ = 3.0, H-3′); 6.34 (t, 1H, $J_{1′,2′}$ = 6.8, H-1′); 7.86 (d, 1H, ^{4}J = 0.7, H-6). ^{13}C NMR (150.9 MHz, D$_2$O, ref(*t*BuOH) = 32.43 ppm): 11.06 (**C**H$_3$CH$_2$N); 12.32 (**C**H$_3$CH$_2$CHOH); 31.26 (CH$_3$**C**H$_2$CHOH); 41.55 (CH$_2$-2′); 49.50 (CH$_3$**C**H$_2$N); 68.13 (d, $J_{C,P}$ = 5.5, CH$_2$-5′); 71.84 (CH$_3$CH$_2$**C**HOH); 73.41 (CH-3′); 88.15 (CH-1′); 88.47 (d, $J_{C,P}$ = 9.1, CH-4′); 120.28 (C-5); 140.53 (CH-6); 154.36 (C-2); 167.46 (C-4). ^{31}P{^{1}H} NMR (202.5 MHz, D$_2$O): -22.15 (bt, J = 19.8, P$_{\beta}$); -10.73 (d, J = 19.8, P$_{\alpha}$); -8.61 (bs, P$_{\gamma}$).

HRMS (ESI$^-$): *m/z* calcd for $C_{12}H_{20}O_{15}N_2P_3$ [M - H$^+$] 525.00820; found: 525.00791.

5-(*R*-1-Hydroxypropyl)-2′-deoxycytidine-5′-*O*-triphosphate, tris(triethylammonium) salt (dC^{Rhp}TP)

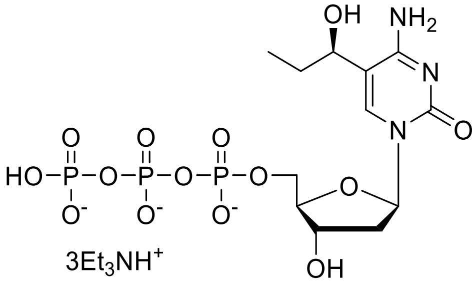

Compound **dC^{Rhp}TP** was synthesized from its corresponding nucleoside **dC^{Rhp}** (30 mg, 0.105 mmol) using **GP4**. $PO(OMe)_3$ (0.509 mL, 0.2 M); first step 2.5 hours at -5 °C, second step at -5 °C. The crude mixture was purified by HPLC (0 to 40% B in A, 60 min, Phenomenex Kinetex EVO C18) followed by POROS 50 HQ (0 to 100% 400 mM TEAB in H_2O, 120 min). Lyophilization gave the product **dC^{Rhp}TP** as a white solid (14.6 mg, 17%).

NMR: ^{1}H NMR (600.1 MHz, D_2O, ref(*t*BuOH) = 1.24 ppm): 0.90 (t, 3H, J_{vic} = 7.4, C**H3**CH_2CHOH); 1.27 (t, 27H, J_{vic} = 7.3, C**H3**CH_2N); 1.78 – 1.86 (m, 2H, CH_3C**H2**CHOH); 2.32 (ddd, 1H, J_{gem} = 14.1, $J_{2'b,1'}$ = 7.4, $J_{2'b,3'}$ = 6.3, H-2′b); 2.41 (ddd, 1H, J_{gem} = 14.1, $J_{2'a,1'}$ = 6.3, $J_{2'a,3'}$ = 3.6, H-2′a); 3.20 (q, 18H, J_{vic} = 7.3, CH_3C**H2**N); 4.18 – 4.27 (m, 3H, H-4′,5′); 4.57 (t, 1H, J_{vic} = 7.1, CH_3CH_2C**H**OH); 4.65 (ddd, 1H, $J_{3',2'}$ = 6.3, 3.6, $J_{3',4'}$ = 2.9, H-3′); 6.34 (dd, 1H, $J_{1',2'}$ = 7.4, 6.3, H-1′); 7.86 (d, 1H, ^4J = 0.7, H-6). ^{13}C NMR (150.9 MHz, D_2O, ref(*t*BuOH) = 32.43 ppm): 11.05 (**C**H_3CH_2N); 12.41 (**C**H_3CH_2CHOH); 30.34 (CH_3**C**H_2CHOH); 42.20 (CH_2-2′); 49.50 (CH_3**C**H_2N); 68.12 (d, $J_{C,P}$ = 5.6, CH_2-5′); 73.07 (CH_3CH_2**C**HOH); 73.48 (CH-3′); 88.49 (d, $J_{C,P}$ = 9.1, CH-4′); 88.76 (CH-1′); 112.64 (C-5); 141.52 (CH-6); 159.86 (C-2); 167.30 (C-4). ^{31}P{^{1}H} NMR (202.4 MHz, D_2O): -22.45 (bt, *J* = 20.1, P_β); -10.92 (d, *J* = 20.1, P_α); -8.97 (bs, P_γ).

HRMS (ESI$^-$): *m/z* calcd for $C_{12}H_{21}O_{14}N_3P_3$ [M - H^+] 524.02418; found: 524.02400.

5-(*S*-1-Hydroxypropyl) -2′-deoxycytidine-5′-*O*-triphosphate, tris(triethylammonium) salt (dC^{Shp}TP)

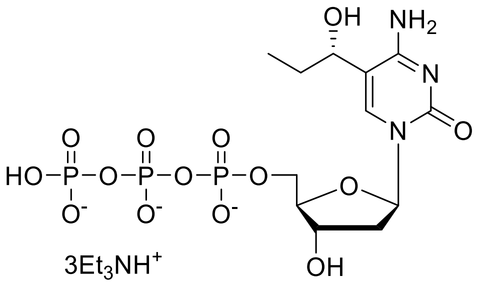

Compound **dC^{Shp}TP** was synthesized from its corresponding nucleoside **dC^{Shp}** (22 mg, 0.077 mmol) using **GP4**. $PO(OMe)_3$ (0.374 mL, 0.2 M); first step 2.5 hours at -5 °C, second step at -5 °C. The crude mixture was purified by HPLC (0 to 40% B in A, 60 min, Phenomenex Kinetex EVO C18) followed by POROS 50 HQ (0 to 100% 400 mM TEAB in H_2O, 120 min). Lyophilization gave the product **dC^{Shp}TP** as a white solid (10.6 mg, 17%).

NMR: ^{1}H NMR (500.0 MHz, D_2O, ref(*t*BuOH) = 1.24 ppm): 0.89 (t, 3H, J_{vic} = 7.4, C**H3**CH_2CHOH); 1.28 (t, 27H, J_{vic} = 7.3, C**H3**CH_2N); 1.76 – 1.86 (m, 2H, CH_3C**H2**CHOH); 2.34 (ddd, 1H, J_{gem} = 14.0, $J_{2'b,1'}$ = 6.9, $J_{2'b,3'}$ = 6.4, H-2′b); 2.43 (ddd, 1H, J_{gem} = 14.0, $J_{2'a,1'}$ = 6.3, $J_{2'a,3'}$ = 3.8, H-2′a); 3.20 (q, 18H, J_{vic} = 7.3, CH_3C**H2**N); 4.17 – 4.27 (m, 3H, H-4′,5′); 4.58 (t, 1H, J_{vic} = 7.1, CH_3CH_2C**H**OH); 4.66 (ddd, 1H, $J_{3',2'}$ = 6.4, 3.8, $J_{3',4'}$ = 2.9, H-3′); 6.31 (dd, 1H, $J_{1',2'}$ = 6.9, 6.3, H-1′); 7.89 (s, 1H, H-6). ^{13}C NMR (125.7 MHz, D_2O, ref(*t*BuOH) = 32.43 ppm): 11.06 (**C**H_3CH_2N); 12.42 (**C**H_3CH_2CHOH); 30.39 (CH_3**C**H_2CHOH); 42.34 (CH_2-2′); 49.50 (CH_3**C**H_2N); 68.01 (d, $J_{C,P}$ = 5.6, CH_2-5′); 73.37 (CH-3′); 73.58 (CH_3CH_2**C**HOH); 88.58 (d, $J_{C,P}$ = 9.2, CH-4′); 88.91 (CH-1′); 112.39 (C-5); 141.62 (CH-6); 159.87 (C-2); 167.22 (C-4). ^{31}P{^{1}H} NMR (202.4 MHz, D_2O): -22.39 (bt, J = 20.1, P_β); -10.94 (d, J = 20.1, P_α); -8.60 (bs, P_γ).

HRMS (ESI$^-$): *m/z* calcd for $C_{12}H_{21}O_{14}N_3P_3$ [M - H$^+$] 524.02418; found: 524.02396.

5-Formyl-2′-deoxyuridine-5′-*O*-triphosphate, tetrakis(triethylammonium) salt (dU^{f}TP)

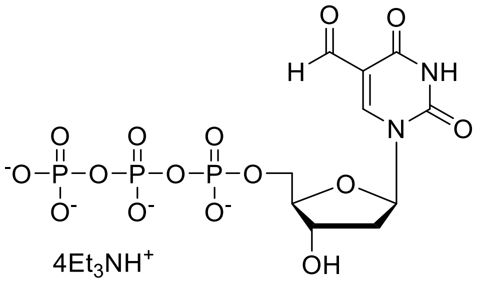

Compound **dU^f^TP** was synthesized from its corresponding nucleoside **dU^f^** (61 mg, 0.238 mmol) using **GP4**. Proton Sponge (51 mg, 1 equiv.) was added. $PO(OMe)_3$ (1.35 mL, 0.18 M); first step 6 hours at 0 °C, second step at 0 °C. The crude mixture was purified by HPLC (2 to 30% B in A, 60 min, Phenomenex Kinetex EVO C18). Lyophilization gave the product **dU^f^TP** as a white solid (33 mg, 15%).

NMR: ^{1}H NMR (500.0 MHz, D_2O, ref(*t*BuOH) = 1.24 ppm): 1.27 (t, 36H, J_{vic} = 7.3, C**H3**CH2N); 2.46 (ddd, 1H, J_{gem} = 14.1, $J_{2'b,3'}$ = 6.4, $J_{2'b,1'}$ = 6.0, H-2′b); 2.51 (ddd, 1H, J_{gem} = 14.1, $J_{2'a,1'}$ = 6.4, $J_{2'a,3'}$ = 5.1, H-2′a); 3.19 (q, 24H, J_{vic} = 7.3, CH3C**H2**N); 4.21 – 4.35 (m, 3H, H-4′,5′); 4.70 (ddd, 1H, $J_{3',2'}$ = 6.4, 5.1, $J_{3',4'}$ = 4.0, H-3′); 6.27 (d, 1H, $J_{1',2'}$ = 6.4, 6.0, H-1′); 8.76 (s, 1H, H-6); 9.63 (s, 1H, CHO). ^{13}C NMR (125.7 MHz, D_2O, ref(*t*BuOH) = 32.43 ppm): 11.06 (**C**H3CH2N); 42.27 (CH2-2′); 49.45 (CH3**C**H2N); 67.50 (d, $J_{C,P}$ = 5.5, CH2-5′); 72.29 (CH-3′); 88.89 (d, $J_{C,P}$ = 9.1, CH-4′); 89.47 (CH-1′); 114.72 (C-5); 154.53 (C-2); 156.50 (CH-6); 166.95 (C-4); 193.06 (CHO). ^{31}P{^{1}H} NMR (202.4 MHz, D_2O): -21.99 (t, *J* = 20.5, P_β); -10.85 (d, *J* = 20.5, P_α); -6.08 (bd, *J* = 20.5, P_γ).

HRMS (ESI$^-$): *m/z* calcd for $C_{10}H_{14}O_{15}N_2P_3$ [M - H$^+$] 494.96125; found: 494.96101.

5-Formyl-2′-deoxycytidine-5′-*O*-triphosphate, tris(triethylammonium) salt (dC^f^TP)

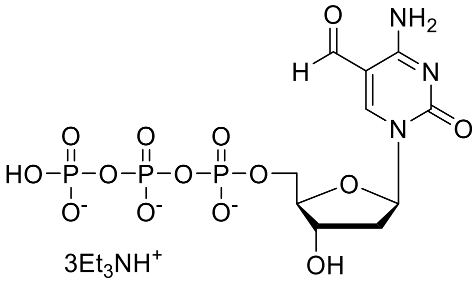

Compound **dC^f^TP** was synthesized from its corresponding nucleoside **dC^f^** (45 mg, 0.176 mmol) using **GP4**. $PO(OMe)_3$ (1 mL, 0.18 M); first step 2 hours at 0 °C, second step at 0 °C. The crude mixture was purified by HPLC (2 to 20% B in A, 60 min, Waters X-Bridge Shield C18). Lyophilization gave the product **dC^f^TP** as a white solid (37.8 mg, 27%). NMR data were in accordance with the literature.[18]

5-Iodo-2′-deoxyuridine-5′-*O*-triphosphate, tris(triethylammonium) salt (dU^I^TP)

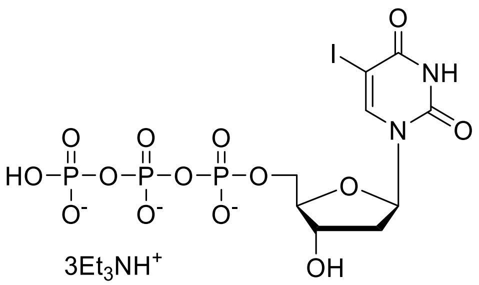

Compound **dU^{I}TP** was synthesized from its corresponding nucleoside **dU^{I}** (200 mg, 0.565 mmol) using **GP4**. $PO(OMe)_3$ (2.2 mL, 0.26 M); first step 6 hours at 0 °C, second step at 0 °C. Crude reaction mixture was separated by Sephadex DEAE (0 to 100% 2 M TEAB in H_2O) followed by HPLC (2 to 30% B in A, 60 min, Phenomenex Kinetex EVO C18). Lyophilization gave the product **dU^{I}TP** as a white solid (87 mg, 17%). NMR data were in accordance with the literature.[19]

5-Iodo-2′-deoxycytidine-5′-*O*-triphosphate, tris(triethylammonium) salt (dC^{I}TP)

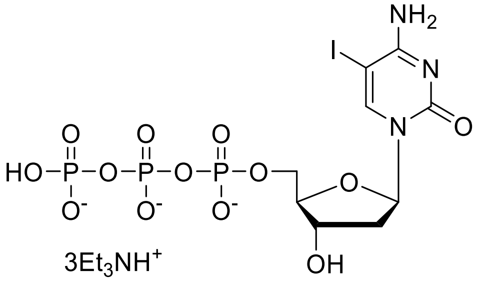

Compound **dC^{I}TP** was synthesized from its corresponding nucleoside **dC^{I}** (200 mg, 0.565 mmol) using **GP4**. $PO(OMe)_3$ (2.2 mL, 0.26 M); first step 6 hours at 0 °C, second step at 0 °C. Crude reaction mixture was separated by Sephadex DEAE (0 to 100% 2 M TEAB in H_2O) followed by HPLC (2 to 30% B in A, 60 min, Phenomenex Kinetex EVO C18). Lyophilization gave the product **dC^{I}TP** as a white solid (102 mg, 20%). NMR data were in accordance with the literature.[20]

5-Ethynyl-2′-deoxycytidine-5′-*O*-triphosphate, tris(triethylammonium) salt (dC^E^TP)

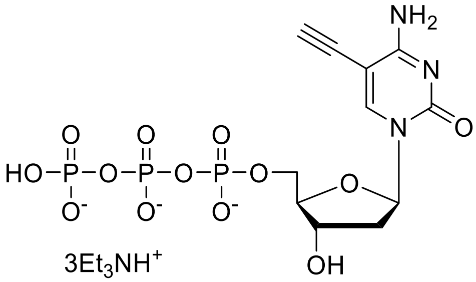

Compound **dC^E^TP** was synthesized from its corresponding nucleoside **dC^E^** (200 mg, 0.796 mmol) using **GP4**. $PO(OMe)_3$ (1.5 mL, 0.5 M); first step 2 hours at 0 °C, second step at 0 °C. The mixture was first separated by Sephadex (0 to 100% 2 M TEAB in H_2O) followed by HPLC (2 to 30% B in A, 60 min, Waters X-Bridge Shield C18). Lyophilization gave the product **dC^E^TP** as a white solid (112 mg, 18%). NMR data were in accordance with the literature.[21]

1.4.1. Synthesis of **dU^V^TP** and **dC^V^TP**

5-Vinyl-2′-deoxyuridine-5′-*O*-triphosphate, tris(triethylammonium) salt (dU^V^TP)

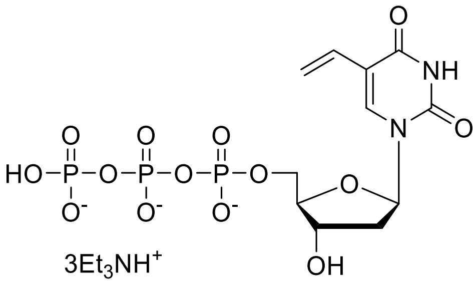

Compound **dU^V^TP** was synthesized according to published procedure.[22] A vial was charged with **dU^I^TP** (43.6 mg, 0.055 mmol), potassium vinyltrifluoroborate (8.8 mg, 1.2 equiv.), Cs_2CO_3 (89.6 mg, 5 equiv.), $Pd(OAc)_2$ (1.2 mg, 0.1 equiv.) and trisodium 3,3′,3″-phosphanetriyltri(benzene-1-sulfonate) (TPPTS, 9.4 mg, 0.3 equiv.). The flask was then evacuated and refilled with argon. Previously degassed solvent mixture (H_2O/MeCN, 1:1, 2 mL) was added and the reaction was stirred at 80 °C for 50 min, then cooled down to 23 °C. The reaction mixture was stopped by addition of EDTA solution (100 mM, 0.5 mL), stirred for additional 1 min and filtered through a celite plug. The solvents were evaporated and the crude mixture was separated by HPLC (2 to 30% B in A, 60 min, Phenomenex KINETEX EVO C18). Lyophilization gave the product **dU^V^TP** as a white solid (6.4 mg, 17%). NMR data were in accordance with the literature.[22]

5-Vinyl-2′-deoxycytidine-5′-*O*-triphosphate, tris(triethylammonium) salt (dC^{V}TP)

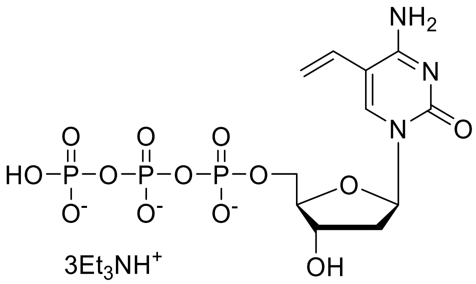

Compound **dC^{V}TP** was synthesized according to published procedure.[22] A vial was charged with **dC^{I}TP** (22.0 mg, 0.028 mmol), potassium vinyltrifluoroborate (4.5 mg, 1.2 equiv.), Cs_2CO_3 (45.2 mg, 5 equiv.), $Pd(OAc)_2$ (0.6 mg, 0.1 equiv.) and TPPTS (4.7 mg, 0.3 equiv.). The flask was then evacuated and refilled with argon. Previously deggased solvent mixture (H_2O/MeCN, 1:1, 1 mL) was added and the reaction was stirred at 80 °C for 50 min, then cooled down to 23 °C. The reaction mixture was stopped by addition of EDTA solution (100 mM, 0.25 mL), stirred for additional 1 min and filtered through a celite plug. The solvents were evaporated and the crude mixture was separated by HPLC (2 to 30% B in A, 60 min, Phenomenex KINETEX EVO C18). Lyophilization gave the product **dC^{V}TP** as a white solid (8.2 mg, 43%). NMR data were in accordance with the literature.[22]

1.5. Assignment of epimers

Epimers of 1-hydroxyethyl- and 1-hydroxypropyl-modified nucleosides (generated by $NaBH_4$ or Grignard reagent reduction, see section 1.3.1. and 1.3.2. respectively) were assigned using combined data from HPLC, X-ray and NMR spectroscopy according to following procedure:

1) Epimers were separated using either reverse phase column (Phenomenex Kinetex EVO C18, in case of **dU^{he}**, **dC^{he}** and **dU^{hp}**) or chiral column (DAICEL Chiralpak IE, **dC^{hp}**) (see section 3). Epimers were labelled as epimer A (epA) or epimer B (epB) according to the elution order from the column (Figure S5-A).

2) Crystallization of all purified epimers was attempted but was successful only in case of **dC^{he}_epA** and **dC^{hp}_epB** (for crystallization conditions, see section 1.5.1.).

3) After the assignment of **dC^{he}_epA** as **dC^{She}** and **dC^{hp}_epB** as **dC^{Shp}** by X-ray crystallography (Figure S5-B) (for more detail, see section 5), the opposite configurations of cytidine derivatives (**dC^{Rhe}** and **dC^{Rhp}**) were assigned accordingly (Table S2).

4) For uridine derivative **dU^{he}**, the assignment consisted of an amination reaction (**GP2**, see section 1.3.2.) of **dU^{he}_epA** to obtain cytidine derivative **dC^{he}** of the same configuration (Figure S5-C-c1). This compound was then combined with a pure epimer of cytidine (**dC^{Rhe}**) in a single NMR tube and ^{1}H NMR spectrum was measured showing if the two mixed epimers possess the same chirality. The signal of *N*-glycosidic hydrogen in ^{1}H NMR spectrum (around 6.2 ppm) appears as a triplet (in fact, it's doublet of doublet) but if both epimers are present, two triplets appear (Figure S5-C-c2). To visualize this better, apodization of the NMR spectra was applied: Exponential = -1.5 Hz, Gaussian = 1.0 Hz; MestReNova program). This experiment provided enough data for correct assignment of the absolute configuration of the starting uridine derivative **dU^{he}_epA**.
Step 4 was repeated for **dU^{hp}_epB** in the same manner (Figure S5-D). Experiment was performed for one epimer of each modification (see Table S2).

Table S2. **Summary of epimer assignments**

Compound	epimer by HPLC	method of assignment
**dU^{Rhe}**	epB	indirectly through amination + ^{1}H NMR
**dU^{She}**	epA	directly through amination + ^{1}H NMR
**dC^{Rhe}**	epB	indirectly by X-ray
**dC^{She}**	epA	directly by X-ray
**dU^{Rhp}**	epB	directly through amination + ^{1}H NMR
**dU^{Shp}**	epA	indirectly through amination + ^{1}H NMR
**dC^{Rhp}**	epA	indirectly by X-ray
**dC^{Shp}**	epB	directly by X-ray

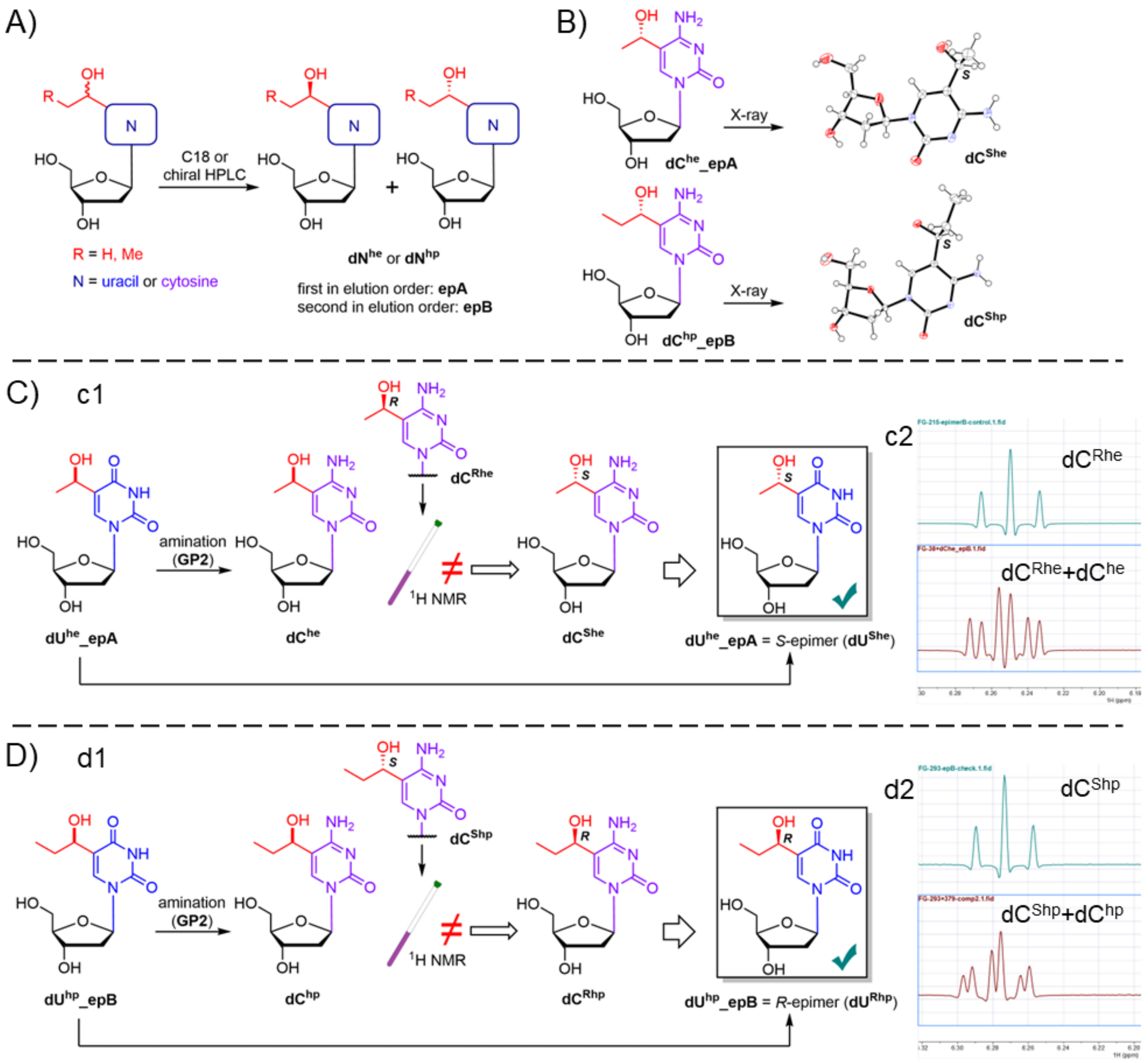

Figure S5. Assignment of absolute configurations of hydroxyethyl-dC and -dU derivatives: A) separation of epimers by HPLC; B) X-ray diffraction of one hydroxyethyl- and one hydroxypropyl-dC epimer; C) direct assignment of **dU^{he}_epA** via amination and comparison with **dC^{Rhe}** (c1) in ^{1}H NMR (c2); D) direct assignment of **dU^{hp}_epB** via amination and comparison with **dC^{Shp}** (d1) in ^{1}H NMR (d2).

1.5.1. Crystallization of epimers

A method of vapor diffusion was used for crystallization of **dC^{he}_epA** and **dC^{hp}_epB** according to following procedure: Pure epimer (approx. 20 mg) was transferred into a small vial without a lid. One drop of water was injected followed by a dropwise addition of MeOH until the compound has just dissolved. This vial was then inserted into a larger vial containing Et_2O

(1.0 mL). The large vial was capped with a lid and the whole system was placed into a fridge for 1 month or until a crystal suitable for X-ray crystallography was formed.

2. Experimental section – biochemistry part

2.1. General remarks – biochemistry

Synthetic oligonucleotides (non-labelled primers, 6-FAM-labelled primers with 6-carboxyfluorescein, and templates) were purchased from GeneriBiotech. Double biotinylated (2xbio) templates were purchased from Biomers. Natural 2′-deoxynucleoside triphosphates (dATP, dCTP, dGTP, dTTP) were purchased from New England Biolabs (NEB). KOD XL DNA polymerase was purchased from Merck, Vent (exo$^-$) and Q5 HotStart DNA polymerases from NEB. All chemicals were purchased from commercial suppliers and were of analytical or molecular biology (BioUltra) grade. Milli-Q water was used for buffers, UPLC-grade water was used in all reaction mixtures. Streptavidin magnetic beads were purchased from Roche. All PCR products and final DNA templates were purified on columns (QIAquick PCR Purification Kit and QIAquick Nucleotide Removal Kit from QIAGEN; E.Z.N.A. MinElute Gel Extraction Kit from Omega Bio-Tek) and/or on Agencourt AMPure XP magnetic particles (Beckman Coulter Life Science - GE Healthcare). Column purifications were done according to the manufactures' manuals. Agarose gels were optionally stained with GelRed (Biotinum, 10 000X in water). Samples after analytical primer extension (PEX) reactions were analyzed by 12.5% denaturing polyacrylamide gel (PAGE, acrylamide/bisacrylamide 19:1, Roth) under denaturing conditions (1 h, 50 °C, 1X TBE buffer). PAGE stop solution used after analytical PEX reactions contains: 95% [v/v] formamide, 0.5 mM EDTA, 0.025% [w/v] bromophenol blue, 0.025% [w/v] xylene cyanol FF, 0.025% [w/v] SDS. PCR reactions were analyzed on agarose gel (SERVA, 8 V/cm, 1 hod, 0.5X TBE buffer), using 6X loading dye (NEB), together with 100bp DNA ladder (NEB) as a reference. PAGE and agarose gels were scanned by fluorescence imaging using Typhoon FLA 9500 Gel Scanner (GE Healthcare). UV-Vis spectra (concentration of products) were measured at 23 °C on NanoDrop1000 (ThermoFisher Scientific). Mass spectra of oligonucleotides were measured on UltrafleXtreme MALDI-TOF/TOF (Bruker) mass spectrometer with 1 kHz smartbeam II laser. The matrix consisted of 3-hydroxypicolinic acid (HPA)/picolinic acid (PA)/ammonium tartrate in ratio 9/1/1. Sanger sequencing was done by SeqMe (Czech Republic).

Table S3. **List of all oligonucleotides used in this study**

Name	Sequence (5‘→ 3‘)[c,d]	Length (nt)
**Prim$^{FOR\text{-}235}$**	CGTCTTCAAGAATTCTAT	18
**Prim$^{FOR\text{-}235}$-FAM**[a]	CGTCTTCAAGAATTCTAT	18
**Prim$^{FOR\text{-}235\text{-}long}$**	CGTCTTCAAGAATTCTATTTGACA	24
**Prim$^{REV\text{-}235}$**	GGAGAGCGTTCACCGACA	18
**Prim$^{REV\text{-}235}$-FAM**[a]	GGAGAGCGTTCACCGACA	18
**Prim^{19ON}**	CATGGGCGGCATGGG	15
**Prim^{19ON}-FAM**[a]	CATGGGCGGCATGGG	15
**Temp^{19ON_T}**	CCCACCCATGCCGCCCATG	19
**Temp^{19ON_T}-2xbio**[b]	CCCACCCATGCCGCCCATG	19
**Temp^{19ON_C}**	CCCGCCCATGCCGCCCATG	19
**Temp^{19ON_C}-2xbio**[b]	CCCGCCCATGCCGCCCATG	19
**Temp^{235}** [e]	CGTCTTCAAGAATTC*TATTTGACAAAAATGGGCTCGTGTTGTACAATAAATGT*G<br>TCTAAGCTTGGGTCCCACCTGACCCCATGCCGAACTCAGAAGTGAAACGCC<br>GTAGCGCCGATGGTAGTGTGGGGTCTCCCCATGCGAGAGTAGGGAACTGC<br>CAGGCATCAAATAAAACGAAAGGCTCAGTCGAAAGACTGGGCCTTTCGTTT<br>TATCTGTTGTTTGTCGGTGAACGCTCTCC	235

[a] 5′- 6-FAM-labelled; [b] 5′- double biotinylated; [c] primer sequences in templates underlined; [d] promotor sequence in italic; [e] double-stranded DNA

2.2. Procedure for single-strand DNA generation by streptavidin magnetic beads

For each sample (containing 150 pmol of DNA in 50 µL volume), 75 µL of streptavidin beads were washed with binding buffer (3 x 200 µL; 10 mM Tris, 1 mM EDTA, 100 mM NaCl, pH = 7.5). The beads were suspended in the binding buffer (50 µL), PEX reaction mixture (50 µL) was added and the sample was incubated at 15 °C for 30 min at 900 rpm. The sample was then put into a magnetic holder and the beads were washed with washing buffer (3 x 200 µL; 10 mM Tris, 1 mM EDTA, 500 mM NaCl) followed by water (3 x 200 µL). Finally, water (40 µL) was added and the sample was denatured at 65 °C for 2 min at 900 rpm followed by quick magnetization of the beads and transferring of the solution into a clean tube. The concentration of prepared single-stranded DNA was measured by Nanodrop and then concentrated accordingly for MALDI-TOF measurement (see section 4).

2.3. Procedure for purification of DNA on Agencourt AMPure XP magnetic particles

To a sample after PCR, re-suspended Agencourt AMPure XP magnetic particles were added (beads/sample volume ratio of 1.8/1). The mixture was mixed 10 to 20 times with a pipette and incubated at 23 °C for 10 min. The microtube containing the mixture was placed into a magnetic holder, the solution was discarded and the magnetic beads were washed with 80% ethanol in water (2 x 200 μL). During the washing, the magnetic beads were incubated for 30-60 seconds at 23 °C with the ethanol. After the second washing and discarding of ethanol, the microtube was left opened and incubated for 5 min at 23 °C to allow residual ethanol to evaporate. Then, water (30 μL) was added and the suspension was mixed 10 to 20 times with pipette, incubated at 23 °C for 5 min and placed on a magnet for 2 min. The eluent was transferred into a new Eppendorf tube and its concentration was measured by Nanodrop.

2.4. Quantification of PCR products

Agarose gels (1.6%, 0.5X TBE) were used for determination of a DNA concentration of all modified PCR products using ImageJ quantification software. Natural DNA of the same length was used as a standard (prepared by protocol in section 2.6.1. and 2.6.4., known concentration, measured by NanoDrop). Samples were quantified either by 6-FAM fluorescence using labelled primers or by GelRed (pre-stained agarose gel with GelRed), using ImageJ software. This method follows previously published procedure.[23]

Table S4. **List of synthetized modified ssONs/dsDNAs**

Name	Sequence (5‘→ 3‘)[c, d]	Length (nt)
**19ON_U^X** [a]	CATGGGCGGCATGGGU*GGG	19
**19DNA_U^X** [a]	CATGGGCGGCATGGGU*GGG	19
**19ON_C^X** [b]	CATGGGCGGCATGGGC*GGG	19
**19DNA_C^X** [b]	CATGGGCGGCATGGGC*GGG	19
**235DNA_U^X** [a]	CGTCTTCAAGAATTCTATU*U*GACAAAAAU*GGGCU*CGU*GU*U*GU*ACAAU*AAA<br>U*GU*GU*CU*AAGCU*U*GGGU*CCCACCU*GACCCCAU*GCCGAACU*CAGAAG<br>U*GAAACGCCGU*AGCGCCGAU*GGU*AGU*GU*GGGGU*CU*CCCCAU*GCGAG<br>AGU*AGGGAACU*GCCAGGCAU*CAAAU*AAAACGAAAGGCU*CAGU*CGAAA<br>GACU*GGGCCU*U*U*CGU*U*U*U*AU*CU*GU*U*GU*U*U*GU*CGGU*GAACGC<br>U*CU*CC	235
**235DNA_C^X** [b]	CGTCTTCAAGAATTCTATTTGAC*AAAAATGGGC*TC*GTGTTGTAC*AATAAATGTG<br>TC*TAAGC*TTGGGTC*C*C*AC*C*TGAC*C*C*C*ATGC*C*GAAC*TC*AGAAG<br>TGAAAC*GC*C*GTAGC*GC*C*GATGGTAGTGTGGGGTC*TC*C*C*C*ATGC*GA<br>GAGTAGGGAAC*TGC*C*AGGC*ATC*AAATAAAAC*GAAAGGC*TC*AGTC*GA<br>AAGAC*TGGGC*C*TTTC*GTTTTATC*TGTTGTTTGTC*GGTGAAC*GC*TC*TC*C*	235

[a] set of modified **dU^X** used, [b] set of modified **dC^X** used, [c] * position of modified nucleotide, [d] promotor sequence in italic, ON – single-stranded DNA; DNA – double-stranded DNA

2.5. Enzymatic synthesis (PEX) of dU^X/dC^X-modified DNA

2.5.1. Single incorporation of one modified dN^{X}TP using 19-mer template – analytical scale

The reaction mixture (20 µl) contained KOD XL DNA polymerase (0.05 U), natural dNTPs (dATP, dGTP and either dCTP or dTTP, 60 µM each), primer **Prim^{19ON} -FAM** (200 nM), one of templates **Temp^{19ON_T}**/ **Temp^{19ON_C}** (300 nM), appropriate **dU^{X}TP** or **dC^{X}TP** (60 µM, see Figure S1 for all modifications) and KOD XL polymerase reaction buffer (2 µL). The reactions were incubated for 30 min at 60 °C in a thermal cycler and then stopped by addition of PAGE stop solution (20 µL), denatured at 95 °C for 5 min. Samples were separated with a 12.5% denaturing PAGE gel and visualized using fluorescence imaging (Figures S6 and S7).

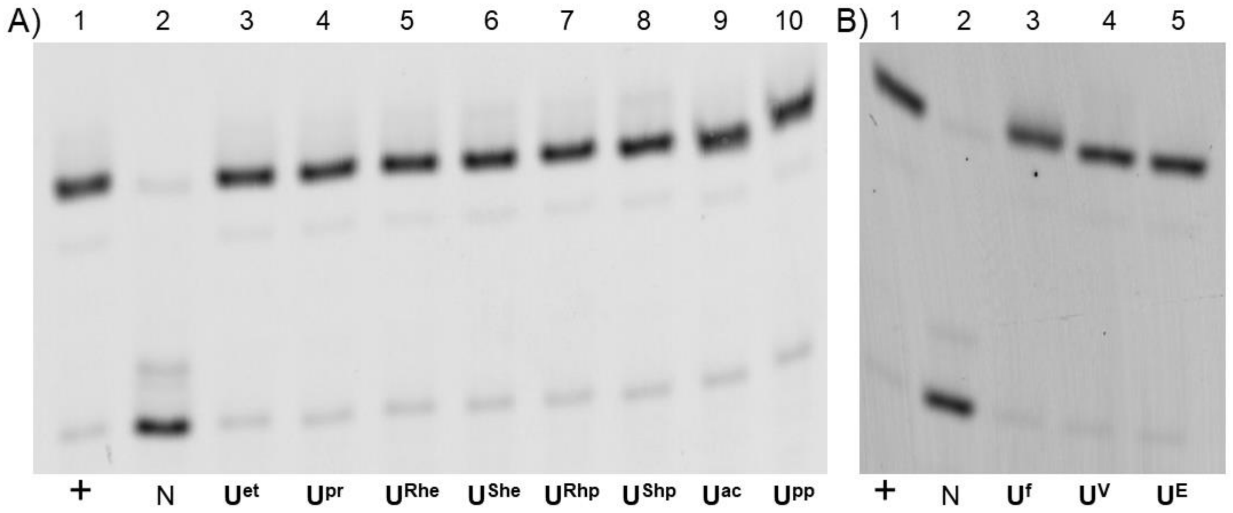

Figure S6. Denaturing PAGE analysis of PEX experiment using KOD XL DNA Polymerase, **Prim^{19ON}-FAM** and **Temp^{19ON_T}**. Both A) and B): lane 1 (+): product with natural dTTP; lane 2 (N): product in the absence of dTTP and **dU^{X}TP**; lanes 3-10: products in presence of appropriate modified **dU^{X}TP**. Residual primer **Prim^{19ON}-FAM** is visible in all cases.

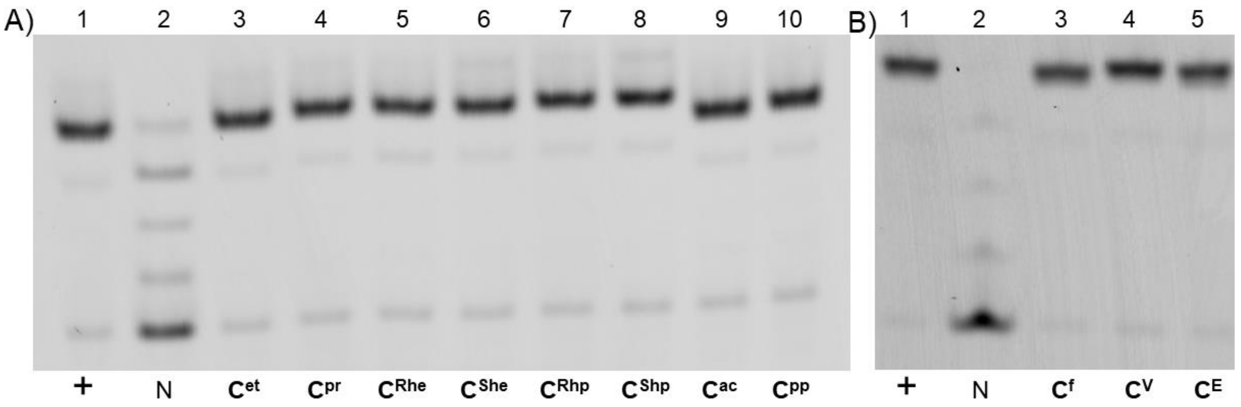

Figure S7. Denaturing PAGE analysis of PEX experiment using KOD XL DNA Polymerase, **Prim^{19ON}-FAM** and **Temp^{19ON_C}**. Both A) and B): lane 1 (+): product with natural dCTP; lane 2 (N): product in the absence of dCTP and **dC^{X}TP**; lanes 3-10: products in presence of appropriate modified **dC^{X}TP**. Residual primer **Prim^{19ON}-FAM** is visible in all cases.

2.5.2. Single incorporation of one modified dN^{X}TP using 19-mer template – semi-preparative scale

The reaction mixture (50 μl) contained KOD XL DNA polymerase (0.25 U), natural dNTPs (dATP, dGTP and either dCTP or dTTP, 320 μM each), primer **Prim^{19ON}** (3 μM), one of templates **Temp^{19ON_T}-2xbio/Temp^{19ON_C}-2xbio** (3 μM), appropriate **dU^{X}TP** or **dC^{X}TP** (320 μM, see Figure S1 for all modifications) and KOD XL polymerase reaction buffer (5 μL).

The reactions were incubated for 30 min at 60 °C in a thermal cycler. Biotinylated PEX products containing **dU^{X}/dC^{X}** modifications were purified and single-stranded DNA was genereated using magnetoseparation procedure (see section 2.2.). Samples were analyzed by MALDI-TOF measurement (section 4, Table S5, Figures S17-S40).

Table S5. **Summary of MALDI-TOF measurements**

Name	Mw calculated^{a} [Da]	Mw found [Da]	Δ [Da]	Figure number
**19ON_U^{et}**	5980.9	5982.5	1.6	S17
**19ON_U^{pr}**	5994.9	5997.7	2.8	S18
**19ON_U^{Rhe}**	5996.9	5999.6	2.7	S19
**19ON_U^{She}**	5996.9	5997.2	0.3	S20
**19ON_U^{Rhp}**	6010.9	6013.6	2.7	S21
**19ON_U^{Shp}**	6010.9	6013.4	2.5	S22
**19ON_U^{ac}**	5994.9	5996.0	1.1	S23
**19ON_U^{pp}**	6008.9	6011.2	2.3	S24
**19ON_C^{et}**	5979.9	5982.1	2.2	S25
**19ON_C^{pr}**	5993.9	5992.5	1.4	S26
**19ON_C^{Rhe}**	5995.9	5995.7	0.2	S27
**19ON_C^{She}**	5995.9	5994.2	1.7	S28
**19ON_C^{Rhp}**	6009.9	6007.7	2.2	S29
**19ON_C^{Shp}**	6009.9	6010.6	0.7	S30
**19ON_C^{ac}**	5993.9	5994.6	0.7	S31
**19ON_C^{pp}**	6007.9	6006.5	1.4	S32
**19ON_U^{f}**	5980.9	5983.3	2.4	S33
**19ON_U^{V}**	5978.9	5981.1	2.2	S34
**19ON_U^{E}**	5976.9	5979.4	2.5	S35
**19ON_C^{f}**	5979.9	5979.6	0.3	S36
**19ON_C^{V}**	5977.9	5978.1	0.2	S37
**19ON_C^{E}**	5975.9	5977.3	1.4	S38

^{a} $[M+H]^{+}$

2.6. Polymerase chain reaction (PCR)

2.6.1. Preparation of Temp^{235}

The PCR reaction mixture (50 µL) contained primers (**Prim$^{FOR\text{-}235\text{-}long}$** and **Prim$^{REV\text{-}235}$**, each 0.8 µM), natural dNTPs mix (200 µM), Pveg plasmid (containing specific promoter region cloned in p770 between *Eco*RI and *Hind*III sites, 10 pM), Q5 polymerase reaction buffer (10 µL) and Q5

HotStart polymerase (1.5 U). Reaction mixture was then run under following cycling protocol: preheating at 98 °C for 30 sec, followed by 30 cycles of denaturation at 98 °C for 10 sec, annealing at 61 °C for 20 sec and extension at 72 °C for 20 sec, finished by final extension at 72 °C for 2 min. A 235bp long product **Temp^{235}** was purified using QIAquick PCR purification kit and analyzed on a 1.6% agarose gel in 0.5X TBE buffer, stained with GelRed (Figure S8) and further used as a template for next PCR experiments (see section 2.6.2.).

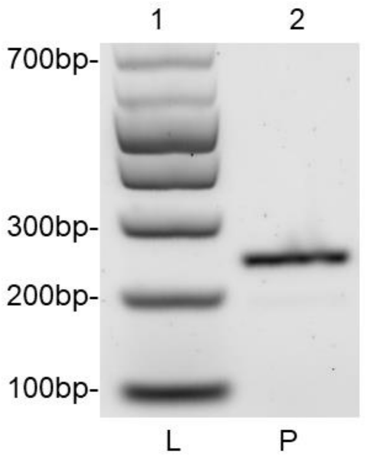

Figure S8. Agarose gel analysis of **Temp^{235}** PCR product, using Q5 Hot Start DNA Polymerase. lane 1 (L): 100bp ladder; lane 2 (P): 235-bp product. Gel stained with GelRed.

2.6.2. PCR using Temp^{235} template and dU^{X}TPs/dC^{X}TPs

The PCR mixture (20 μL) contained primers (**Prim$^{FOR-235}$-FAM** and A**Prim$^{REV-235}$-FAM**, each 1 μM), mixture of three natural dNTPs (dATP, dGTP and either dTTP or dCTP, 150 – 200 μM, see Table S6) and either **dU^{X}TP** or **dC^{X}TP** (for natural control either dTTP or dCTP, 150 – 500 μM, see Table S6), template **Temp^{235}** (20 ng/μL, 0.5 μL), KOD XL buffer (2 μL) (or Thermopol buffer in case of **dC^{E}TP**, 2 μL) and KOD XL DNA polymerase (1 – 3 U, see Table S6) (or Vent (exo^{-}) polymerase in case of **dC^{E}TP**, 1.5 U). All reaction mixtures were run under following cycling conditions: preheating at 94 °C for 3 min, followed by 30 cycles of denaturation at 94 °C for 30 sec, annealing at 52 °C for 30 sec and extension at 72 °C for 1 min, finished by final extension at 72 °C for 5 min. Products (natural control, **235DNA_U^{X}** and **235DNA_C^{X}**) were purified using AMPure XP beads (see section 2.3.), analyzed on a 1.6% agarose gel in 0.5X TBE buffer stained with GelRed (Figure S9) and further used for:

1) transcription studies (see section 2.7.)

2) re-PCR reaction for Sanger sequencing (see section 2.6.3.)

Table S6. **Amounts of triphosphates and polymerase used for the PCR using Temp^{235} template and modified dN^{X}TPs.**

Modification	nat. dNTP [μM]	**dN^{X}TP** [μM]	KOD XL [U]
dT	150	0	1
**dU^{et}**	150	150	1
**dU^{pr}**	150	150	1
**dU^{Rhe}**	200	500	3
**dU^{She}**	150	300	2
**dU^{Rhp}**	200	500	3
**dU^{Shp}**	200	400	3
**dU^{ac}**	200	400	3
**dU^{pp}**	200	400	3
**dC^{et}**	150	150	1
**dC^{pr}**	150	150	1
**dC^{Rhe}**	150	300	2
**dC^{She}**	150	300	2
**dC^{Rhp}**	200	400	3
**dC^{Shp}**	200	400	3
**dC^{ac}**	200	400	3
**dC^{pp}**	200	400	3
**dU^{f}**	200	400	2
**dU^{V}**	200	300	1.5
**dU^{E}**	200	200	1.5
**dC^{f}**	200	200	1.5
**dC^{V}**	200	300	1.5
**dC^{E}**	200	400	1.5[a]

[a] Vent (exo^{-}) DNA polymerase used instead of KOD XL

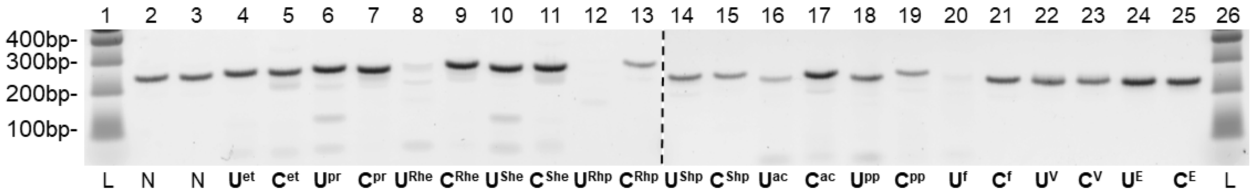

Figure S9. Agarose gel analysis of PCR products using **Temp^{235}** amplified by KOD XL (lanes 2-24) or Vent (exo^{-}) (lane 25) DNA polymerase: lanes 1, 26 (L): 100 bp ladder; lanes 2, 3 (N): natural dNTPs; lanes 4-25: modified **dN^{X}TPs**. Lanes 8, 12 and 20 show insufficient amplification of the desired DNA. Gel stained with GelRed.

2.6.3. PCR using 235DNA_U^{X} and 235DNA_C^{X} templates

To confirm that all prepared **dU^{X}/dC^{X}** – modified DNA samples have the correct sequence, each of them was used as a template in a PCR reaction with natural dNTPs. The 20 μL mixture contained KOD XL polymerase (0.6 U), primers (**Prim$^{FOR-235}$** and **Prim$^{REV-235}$**, both 1 μM), mixture of natural dNTPs (200 μM), KOD XL buffer (2 μL) and templates (**Temp^{235}**, **235DNA_U^{X}** or **235DNA_C^{X}**, 20 ng/μL, 0.5 μL for each). The reaction mixtures were run under following conditions: preheating at 94 °C for 3 min, followed by 15 cycles of denaturation at 94 °C for 30 sec, annealing at 52 °C for 30 sec and extension at 72 °C for 45 sec, finished by final extension at 72 °C for 5 min. Products were purified using QIAquick PCR purification kit and analyzed on a 1.6% agarose gel in 0.5X TBE buffer (Figure S10) stained with GelRed and further used for Sanger sequencing (see section 7).

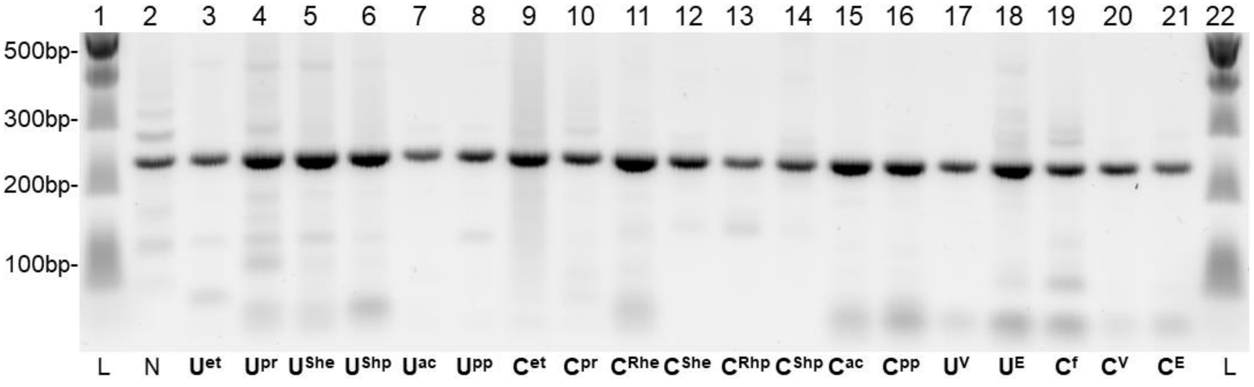

Figure S10. Agarose gel analysis of natural PCR products using templates **Temp^{235}**, **235DNA_U^{X}** or **235DNA_C^{X}**: lanes 1, 22 (L): 100 bp ladder; lane 2 (N): product of natural template re-amplification; lanes 3-21: products of re-amplification using **235DNA_U^{X}** or **235DNA_C^{X}** as templates and natural dNTPs. Gel stained with GelRed.

2.7. Transcription studies

2.7.1. Multiple round transcription experiments

In vitro transcription assays were performed by mixing Tris-HCl (40 mM, pH 8), $MgCl_2$ (10 mM), DTT (1 mM), KCl (90 mM), BSA (1X), RNAP-σ^{70} holoenzyme (NEB, 30 nM) and either natural or modified DNA template (**Temp^{235}**, **235DNA_U^{X}** or **235DNA_C^{X}**, 5 ng) bearing the Pveg promoter[24]. This mix was incubated 10 min at 37 °C to allow for open complex formation between RNAP and the promoter-bearing template DNA. Transcription reactions were initiated by the addition of rNTPs mix (200 µM ATP, 1000 µM GTP, 200 µM CTP, 10 µM UTP, 37 kBq [α-^{32}P] UTP) to a final volume of 10 µL.The reaction mixture was incubated at 37 °C for 10 min and then stopped by the addition of stop solution (95% formamide, 20 mM EDTA, 0.05% bromophenol blue, 0.05% xylene cyanol; 10 µL) and stored at -20 °C. The samples were then denatured at 95 °C for 5 min, chilled on ice and 10 µl were loaded onto 7% polyacrylamide denaturing gel that was run at 180 V for 2 hours. Gel was dried in vacuum at 85 °C for 45 min, let to cool down and exposed to Fuji MS phosphor storage screen overnight. The screen was scanned with Amersham Typhoon scanner (Cytiva) and the signals were quantified with Quantity One software (Biorad).

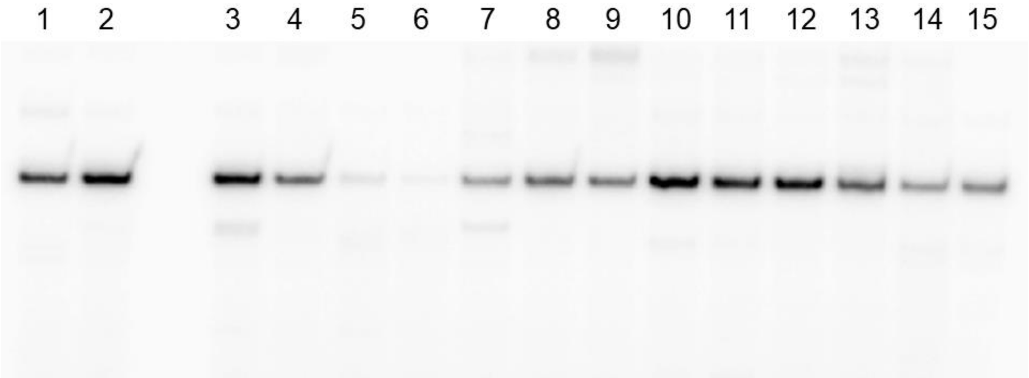

Figure S11. PAGE analysis of radiolabelled transcription products using templates **Temp^{235}**, **235DNA_U^{X}** or **235DNA_C^{X}**: Lane 1 (K+): RNA produced by using **Temp^{235}**; lanes 2-15: RNA produced using modified **235DNA_U^{X}** or **235DNA_C^{X}**.

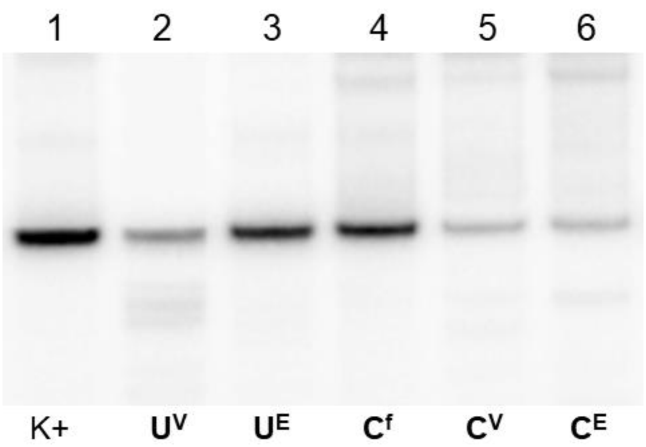

Figure S12. PAGE analysis of radiolabelled transcription products using templates **Temp^{235}**, **235DNA_U^{X}** or **235DNA_C^{X}**: Lane 1 (K+): RNA produced by using **Temp^{235}**; lanes 2-6: RNA produced using modified **235DNA_U^{X}** or **235DNA_C^{X}**.

3. Copies of HPLC chromatograms

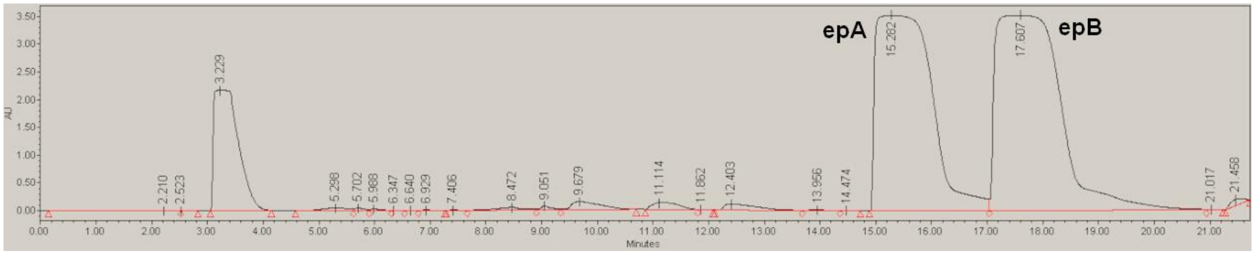

Figure S13. HPLC chromatogram of 5-(1-hydroxyethyl)-2′-deoxyuridine (**dU^{he}**) epimers separation. First epimer (epA, **dU^{he}_epA**), R_t = 15.5 min; second epimer (epB, **dU^{he}_epB**), R_t = 17.8 min. Column Kinetex EVO, gradient from 0 to 30% MeOH in H_2O, 40 min, 15 mL/min.

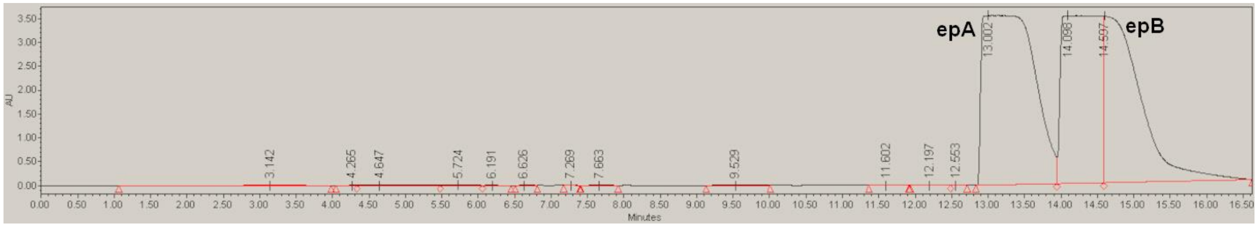

Figure S14. HPLC chromatogram of 5-(1-hydroxyethyl)-2′-deoxycytidine (**dC^{he}**) epimers separation. First epimer (epA, **dC^{he}_epA**), R_t = 13.3 min; second epimer (epB, **dC^{he}_epB**), R_t = 14.5 min. Column Kinetex EVO, gradient from 0 to 50% MeOH in H_2O, 50 min, 10 mL/min.

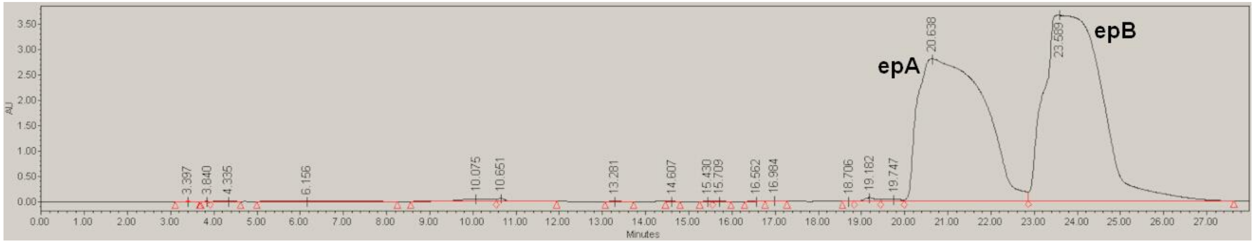

Figure S15. HPLC chromatogram of 5-(1-hydroxypropyl)-2′-deoxyuridine (**dU^{hp}**) epimers separation. First epimer (epA, **dU^{hp}_epA**), R_t = 21.0 min; second epimer (epB, **dU^{hp}_epB**), R_t = 24.0 min. Column Kinetex EVO, gradient from 0 to 30% MeOH in H_2O, 40 min, 15 mL/min.

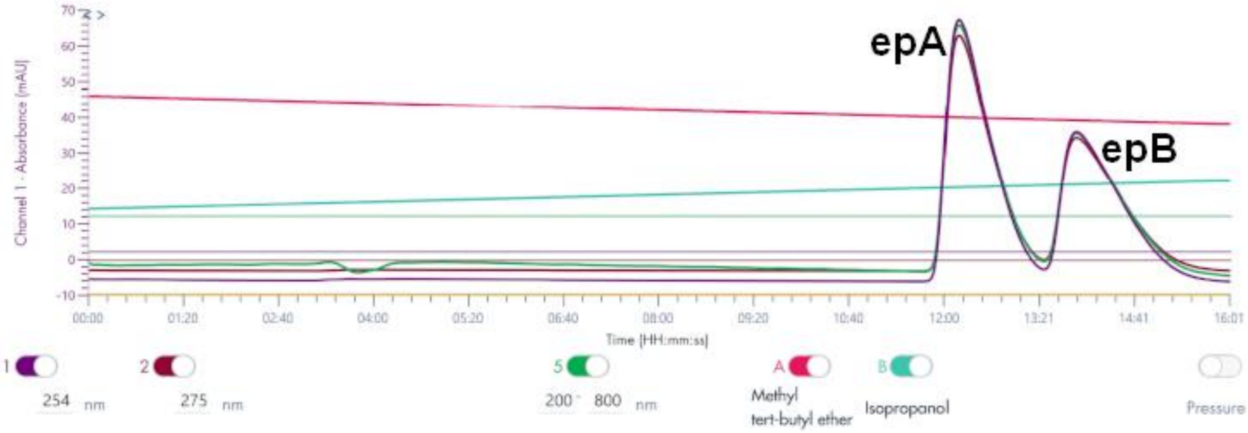

Figure S16. HPLC chromatogram of 5-(1-hydroxypropyl)-2′-deoxycytidine (**dC^{hp}**) epimers separation. First epimer (epA, **dC^{hp}_epA**), R_t = 12.2 min; second epimer (epB, **dC^{hp}_epB**), R_t = 14.0 min. Column DAICEL Chiralpak IE, gradient from 30 to 40% isopropanol in MTBE with 0.5% Et_2NH, 16 min, 20 mL/min.

4. MALDI-TOF measurements

All samples were prepared according to procedure described above (see section 2.5.2.) and the results are summarized in Table S5.

4.1. Copies of MALDI-TOF spectra

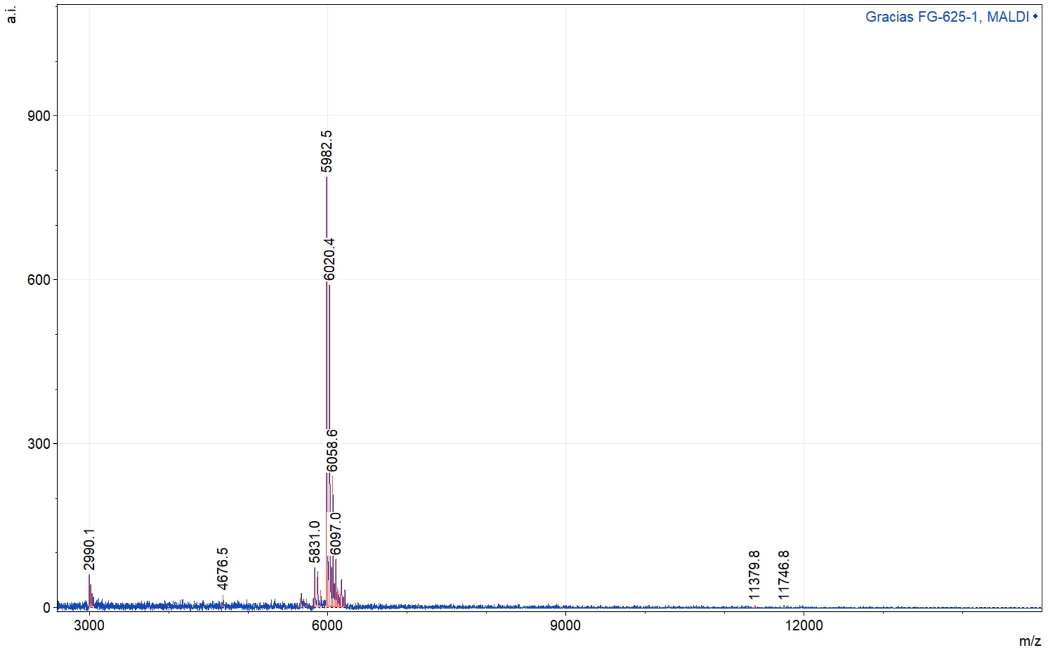

Figure S17. MALDI-TOF MS spectrum of **19ON_U$^{\mathbf{et}}$**: *m/z* calcd for $[M+H]^+$: 5980.9 Da; found: 5982.5; Δ = 1.6 Da.

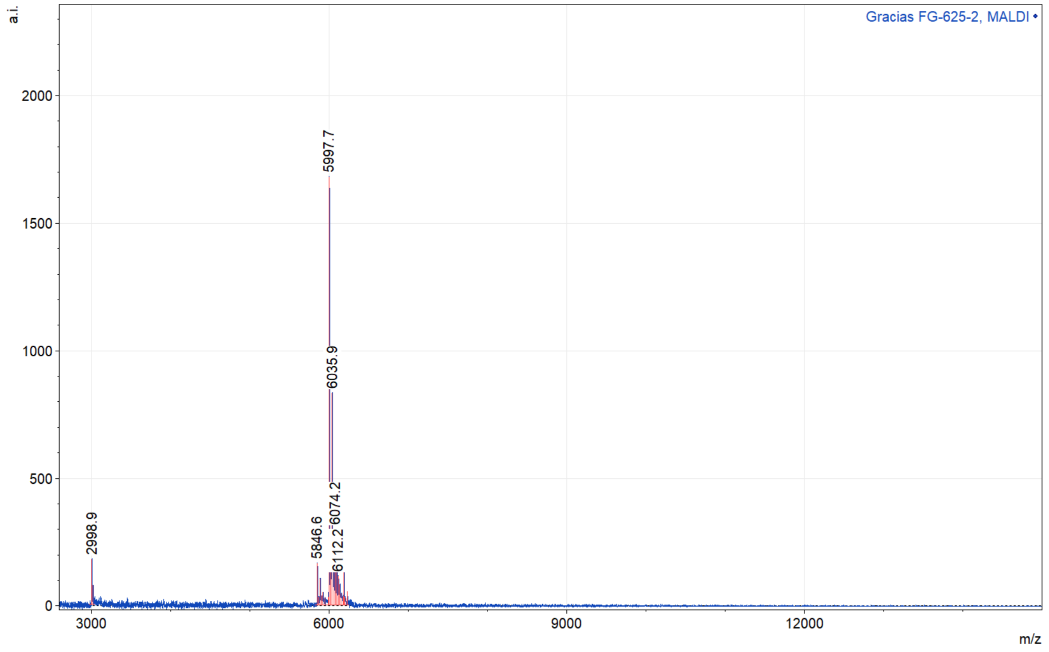

Figure S18. MALDI-TOF MS spectrum of **19ON_U$^{\mathbf{pr}}$**: *m/z* calcd for $[M+H]^+$: 5994.9 Da; found: 5997.7; Δ = 2.8 Da.

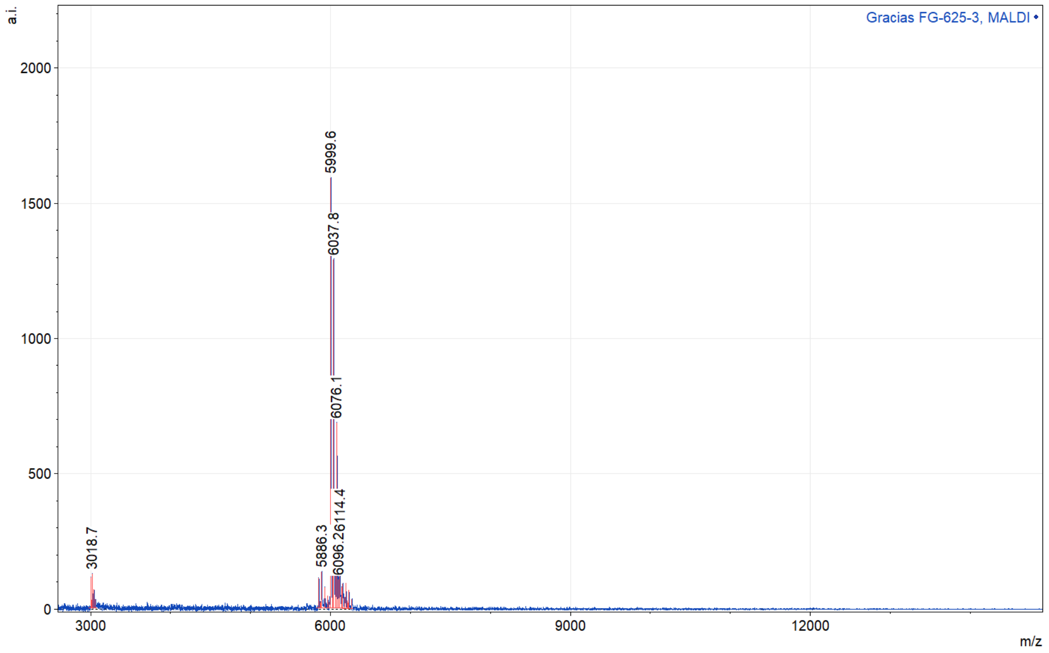

Figure S19. MALDI-TOF MS spectrum of **19ON_U$^{\mathbf{Rhe}}$**: *m/z* calcd for $[M+H]^+$: 5996.9 Da; found: 5999.6; Δ = 2.7 Da.

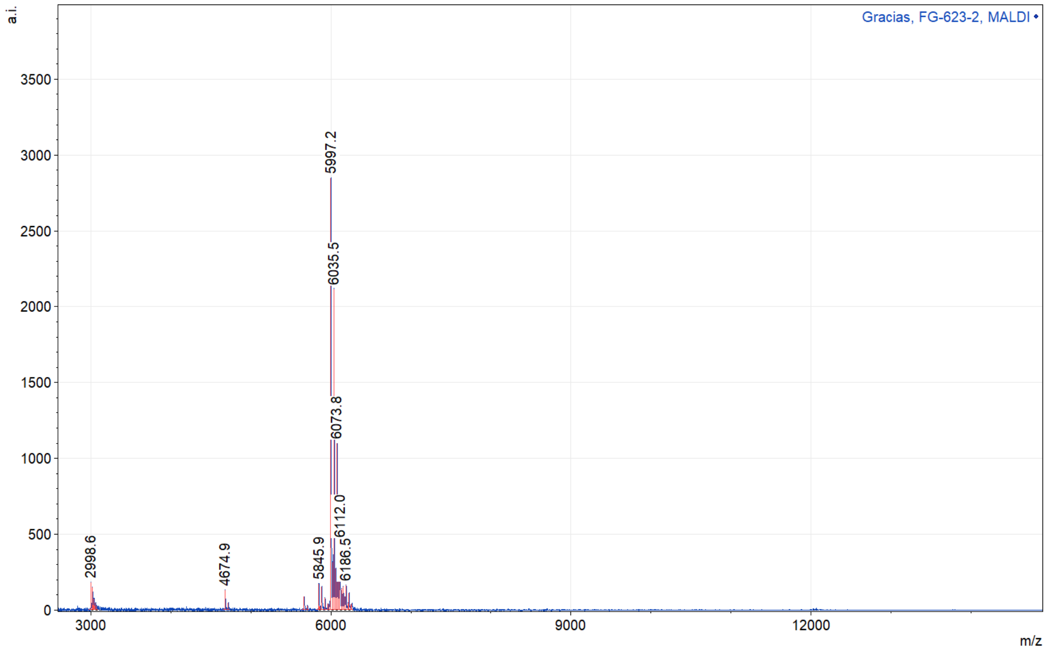

Figure S20. MALDI-TOF MS spectrum of **19ON_U$^{\mathbf{She}}$**: *m/z* calcd for $[M+H]^+$: 5996.9 Da; found: 5997.2; Δ = 0.3 Da.

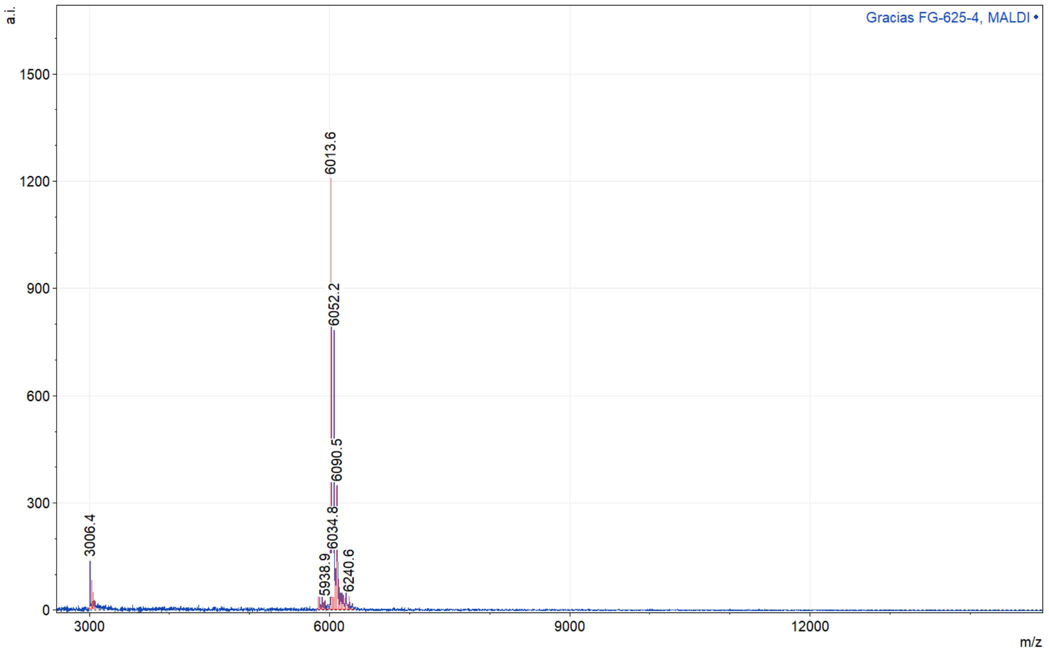

Figure S21. MALDI-TOF MS spectrum of **19ON_U$^{\mathbf{Rhp}}$**: *m/z* calcd for $[M+H]^+$: 6010.9 Da; found: 6013.6; Δ = 2.7 Da.

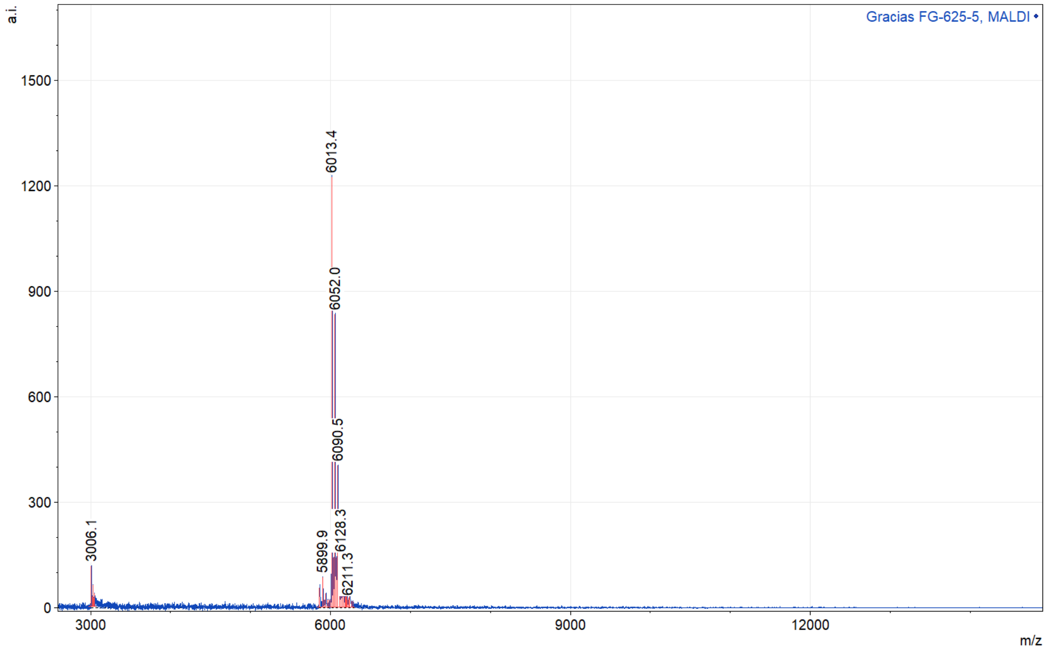

Figure S22. MALDI-TOF MS spectrum of **19ON_U$^{\mathbf{Shp}}$**: *m/z* calcd for $[M+H]^+$: 6010.9 Da; found: 6013.4; Δ = 2.5 Da.

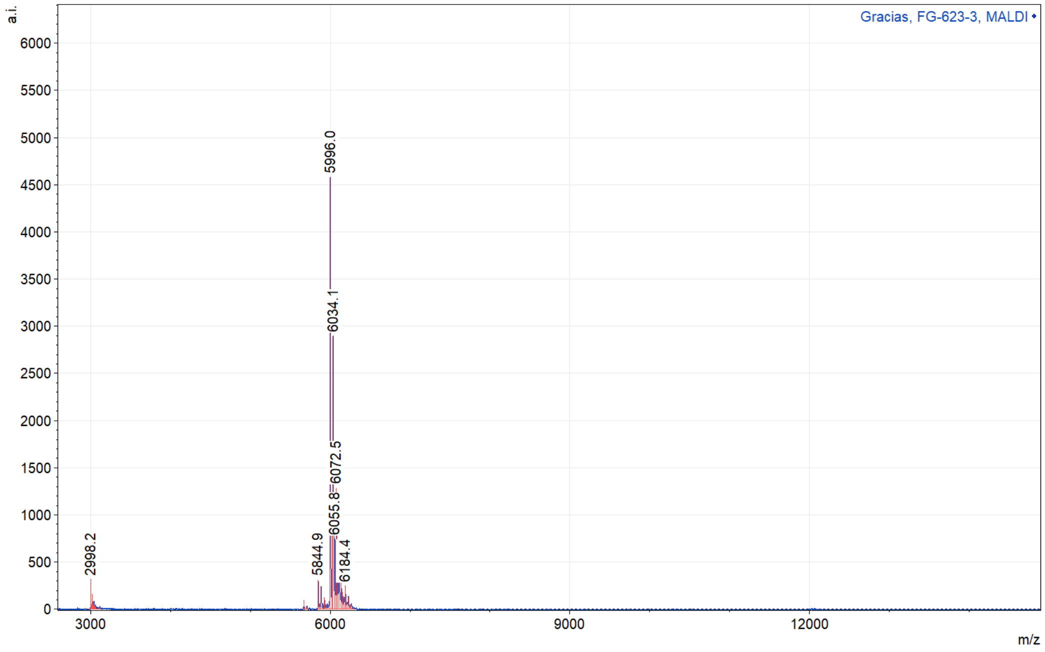

Figure S23. MALDI-TOF MS spectrum of **19ON_U^{ac}**: *m/z* calcd for $[M+H]^+$: 5994.9 Da; found: 5996.0; Δ = 1.1 Da.

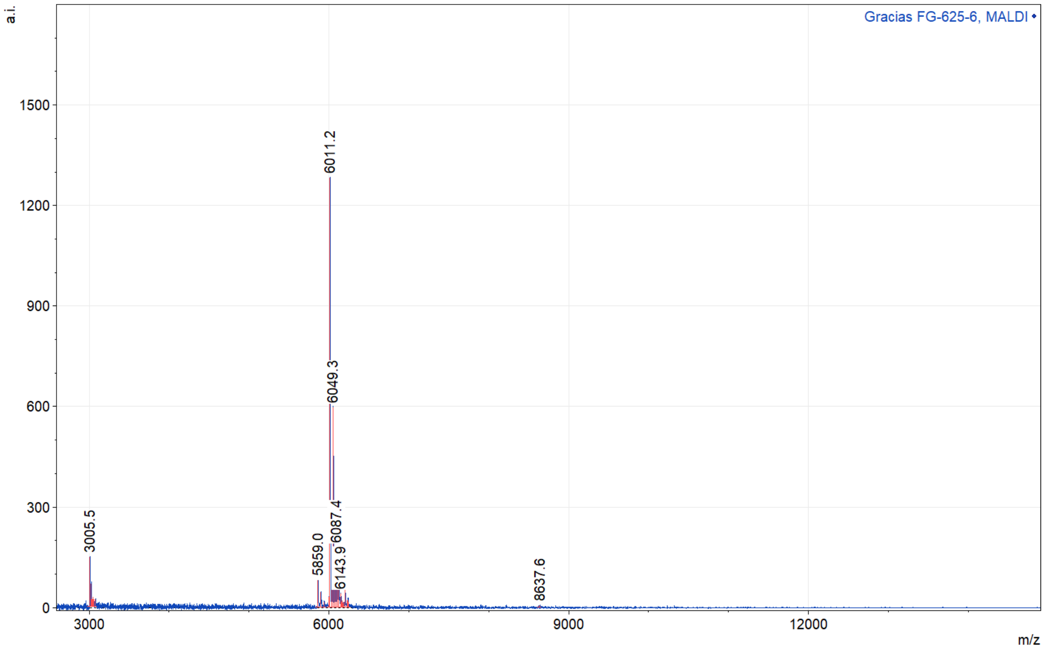

Figure S24. MALDI-TOF MS spectrum of **19ON_U^{pp}**: *m/z* calcd for $[M+H]^+$: 6008.9 Da; found: 6011.2; Δ = 2.3 Da.

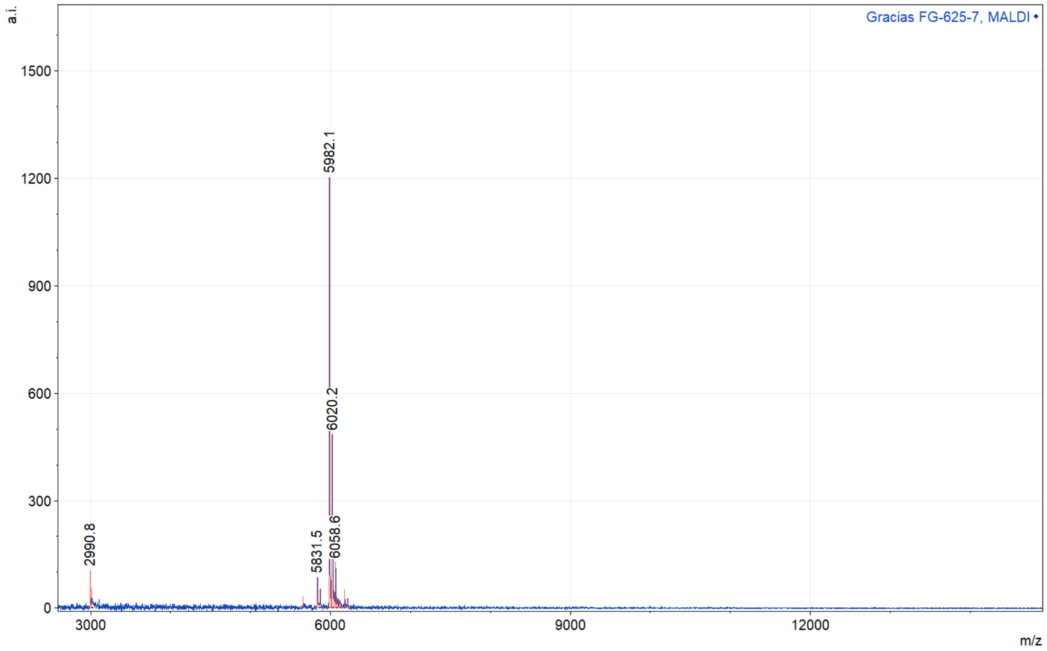

Figure S25. MALDI-TOF MS spectrum of **19ON_C^{et}**: *m/z* calcd for $[M+H]^+$: 5979.9 Da; found: 5982.1; Δ = 2.2 Da.

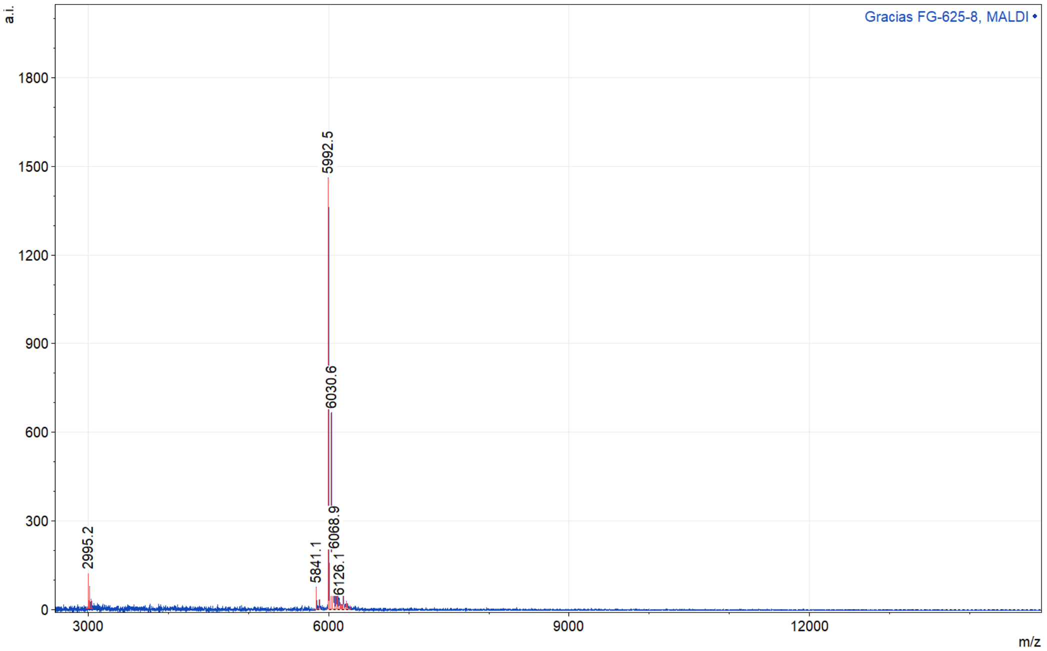

Figure S26. MALDI-TOF MS spectrum of **19ON_C^{pr}**: *m/z* calcd for $[M+H]^+$: 5993.9 Da; found: 5992.5; Δ = 1.4 Da.

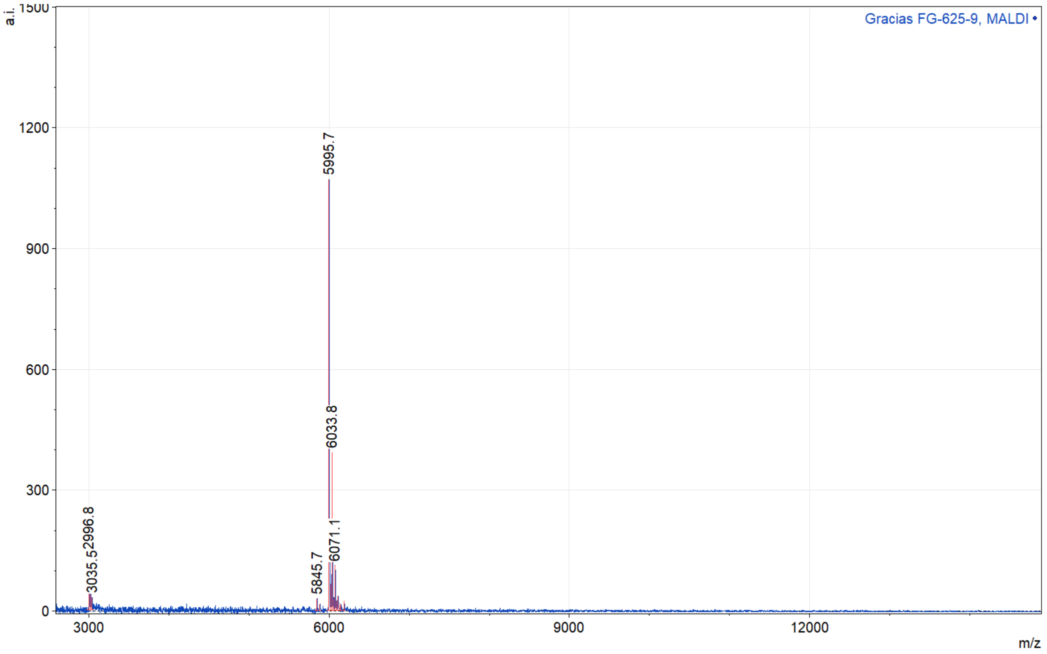

Figure S27. MALDI-TOF MS spectrum of **19ON_C$^{\mathbf{Rhe}}$**: *m/z* calcd for $[M+H]^+$: 5995.9 Da; found: 5995.7; Δ = 0.2 Da.

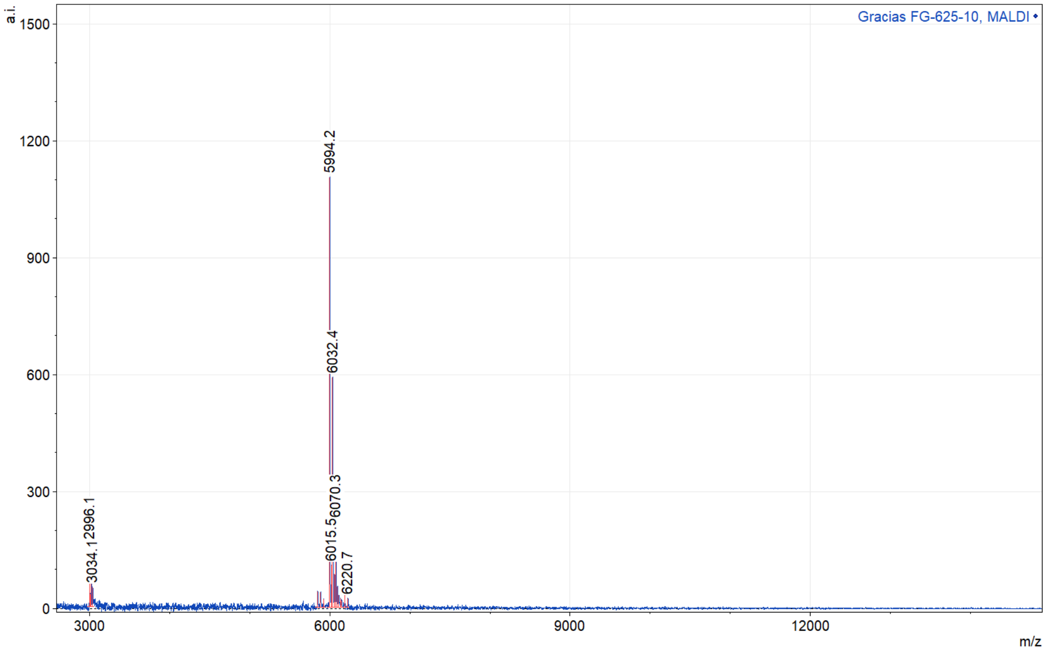

Figure S28. MALDI-TOF MS spectrum of **19ON_C$^{\mathbf{She}}$**: *m/z* calcd for $[M+H]^+$: 5995.9 Da; found: 5994.2; Δ = 1.7 Da.

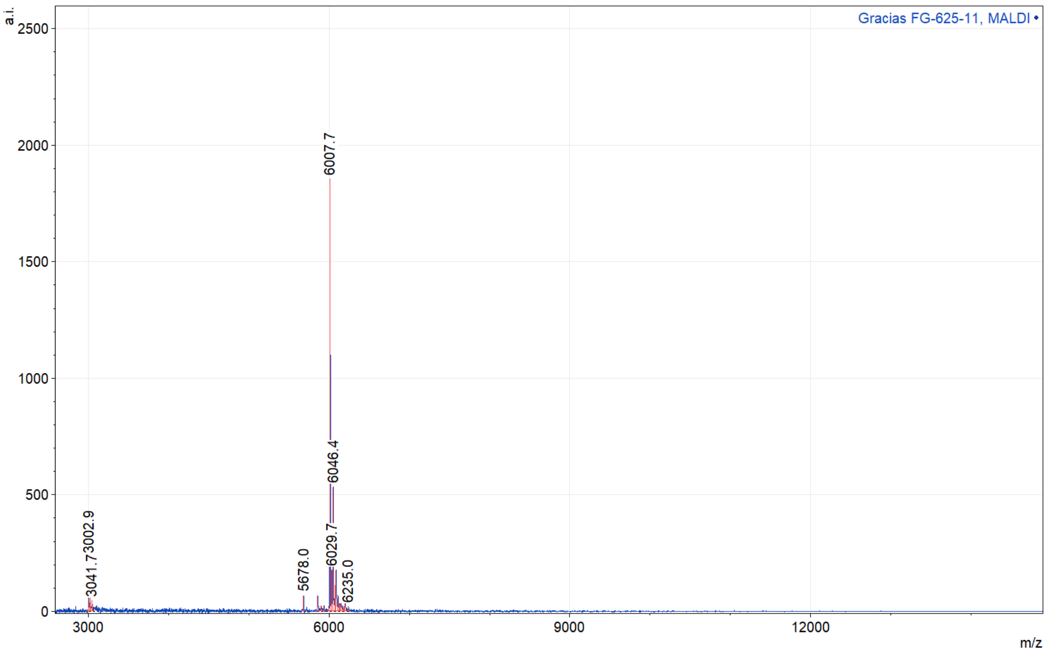

Figure S29. MALDI-TOF MS spectrum of **19ON_C$^{\mathbf{Rhp}}$**: *m/z* calcd for $[M+H]^+$: 6009.9 Da; found: 6007.7; Δ = 2.2 Da.

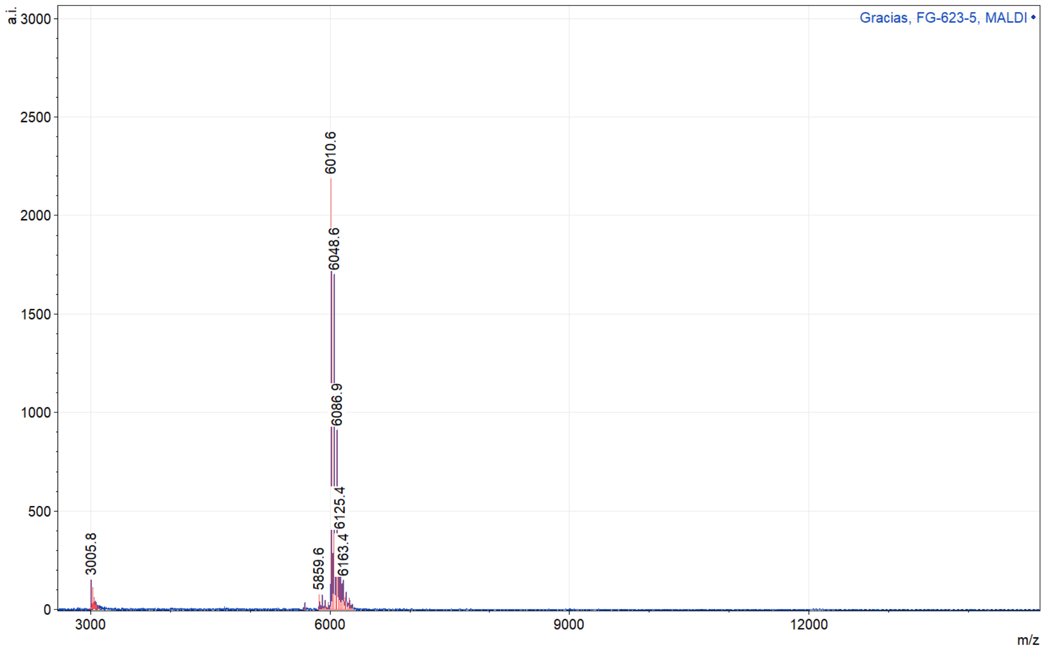

Figure S30. MALDI-TOF MS spectrum of **19ON_C$^{\mathbf{Shp}}$**: *m/z* calcd for $[M+H]^+$: 6009.9 Da; found: 6010.6; Δ = 0.7 Da.

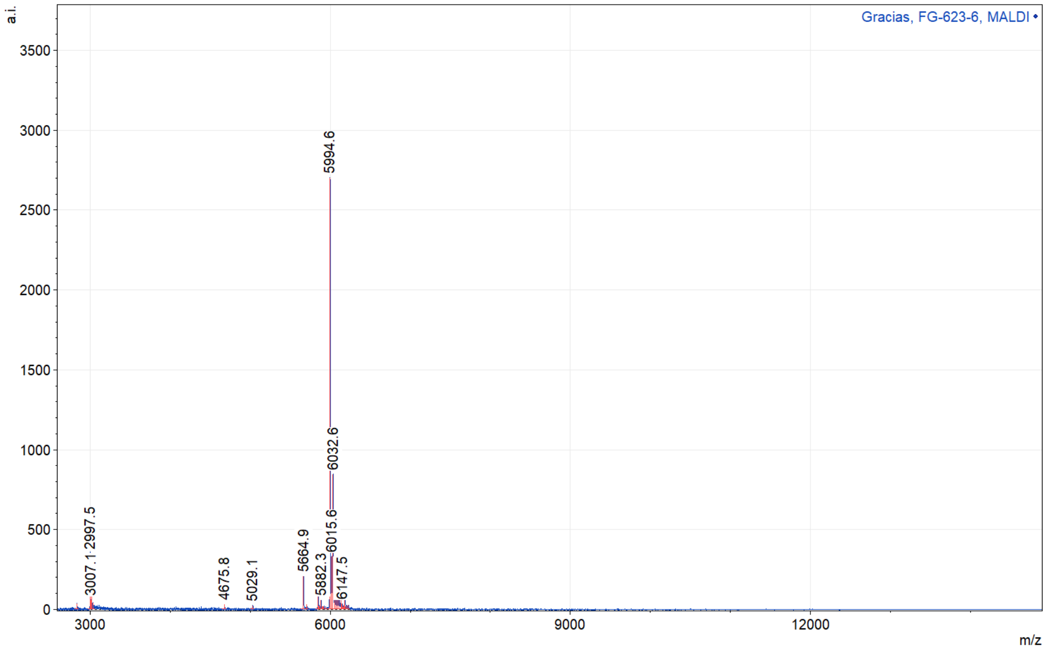

Figure S31. MALDI-TOF MS spectrum of **19ON_C**^{ac}: *m/z* calcd for $[M+H]^+$: 5993.9 Da; found: 5994.6; Δ = 0.7 Da.

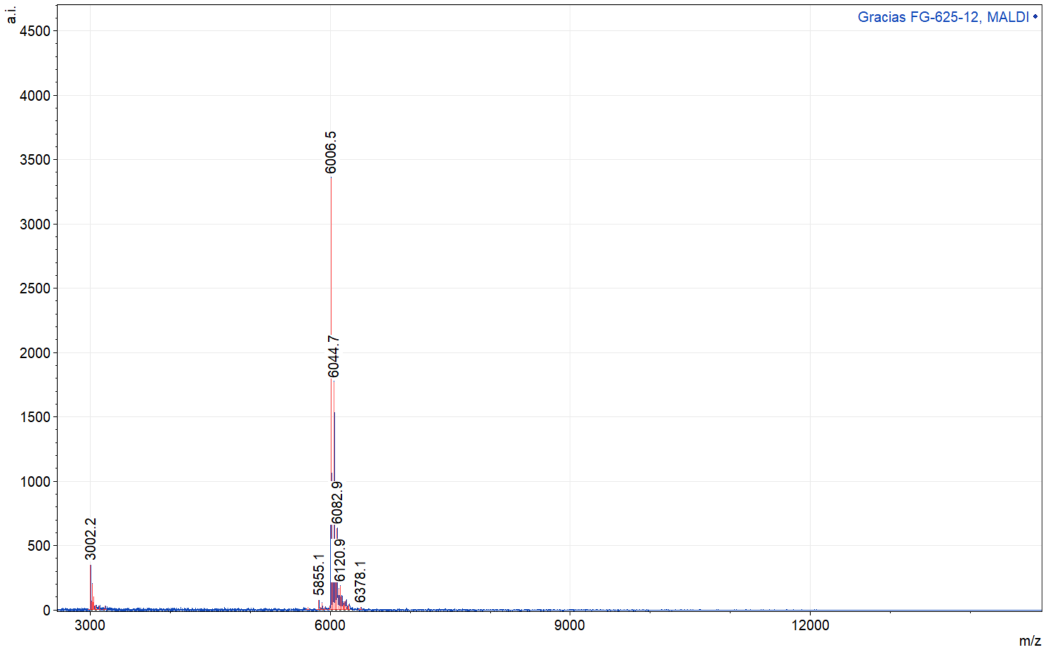

Figure S32. MALDI-TOF MS spectrum of **19ON_C**^{pp}: *m/z* calcd for $[M+H]^+$: 6007.9 Da; found: 6006.5; Δ = 1.4 Da.

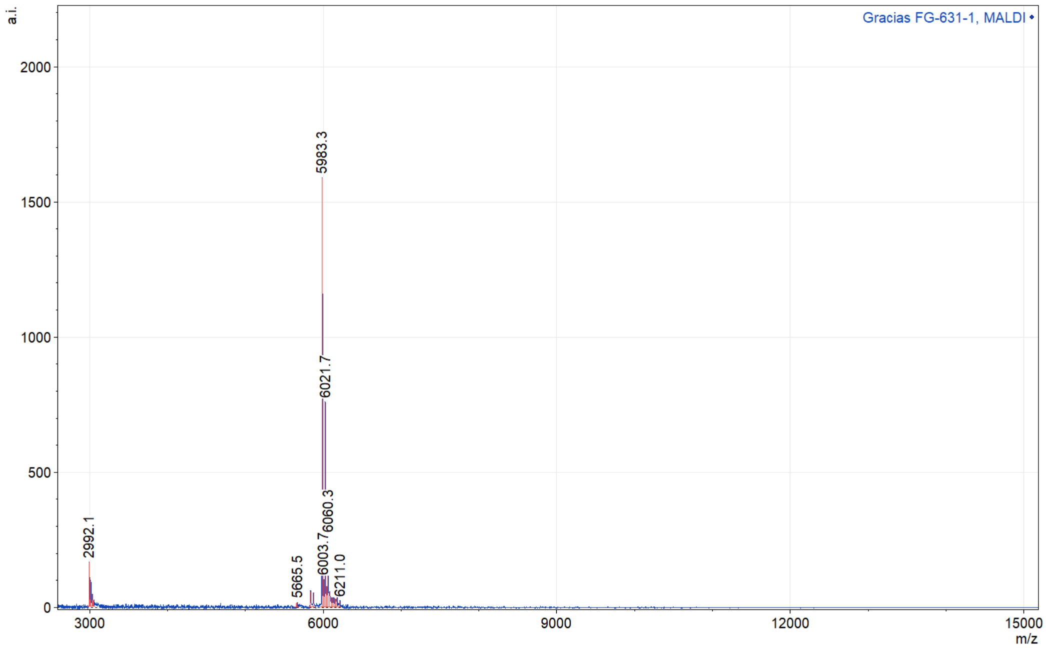

Figure S33. MALDI-TOF MS spectrum of **19ON_U^{f}**: *m/z* calcd for $[M+H]^+$: 5980.9 Da; found: 5983.3; Δ = 2.4 Da.

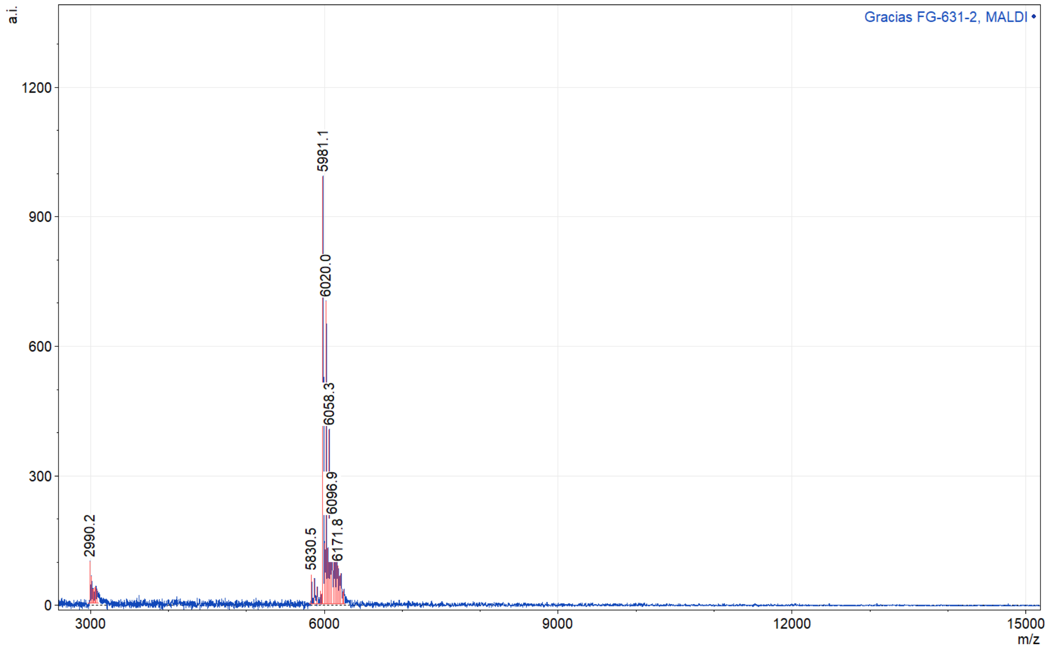

Figure S34. MALDI-TOF MS spectrum of **19ON_U^{V}**: *m/z* calcd for $[M+H]^+$: 5978.9 Da; found: 5981.1; Δ = 2.2 Da.

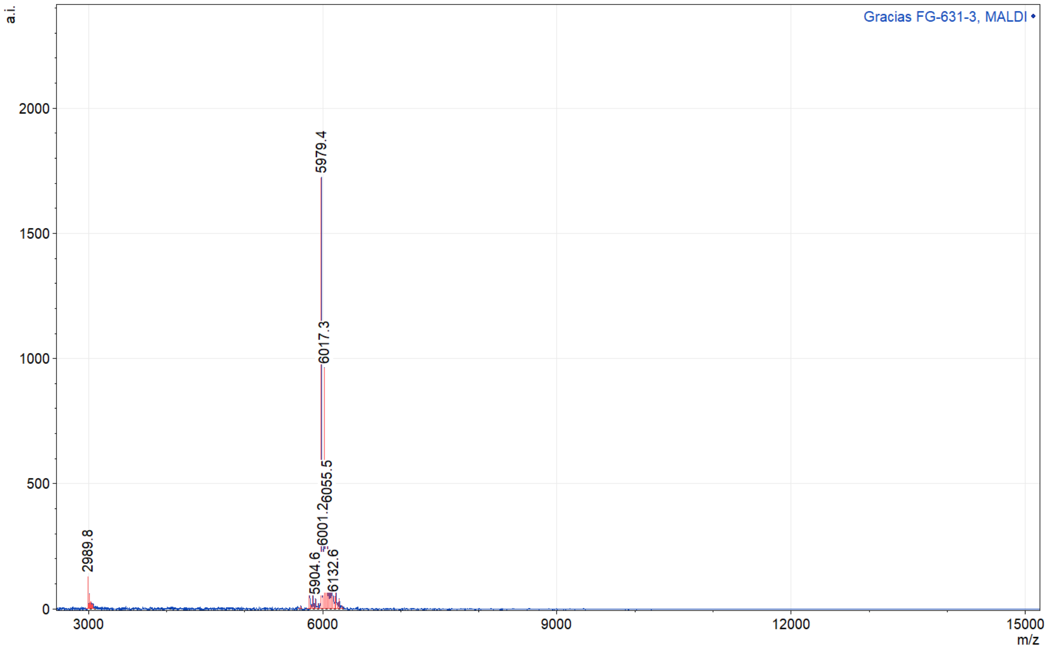

Figure S35. MALDI-TOF MS spectrum of **19ON_U^{E}**: *m/z* calcd for $[M+H]^+$: 5976.9 Da; found: 5979.4; Δ = 2.5 Da.

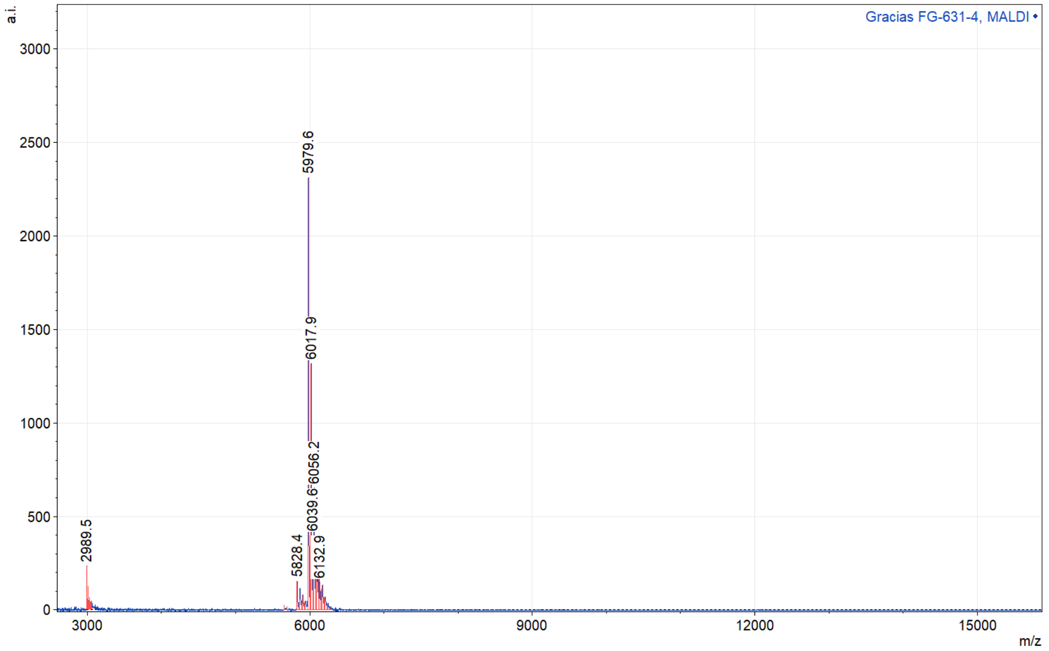

Figure S36. MALDI-TOF MS spectrum of **19ON_C^{f}**: *m/z* calcd for $[M+H]^+$: 5979.9 Da; found: 5979.6; Δ = 0.3 Da.

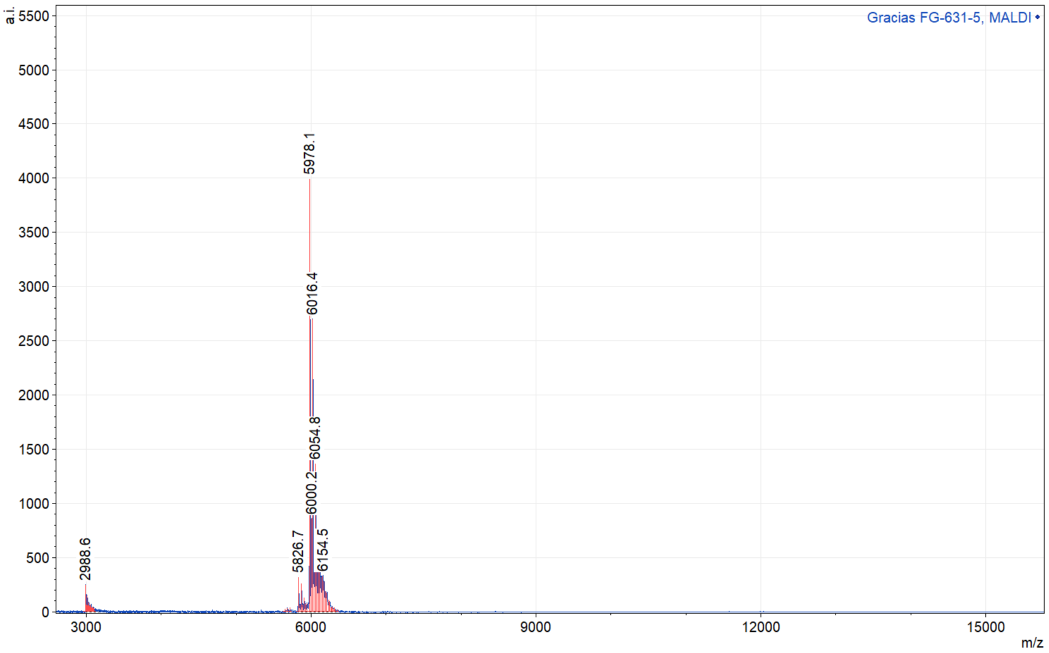

Figure S37. MALDI-TOF MS spectrum of **19ON_C^{V}**: *m/z* calcd for $[M+H]^{+}$: 5977.9 Da; found: 5978.1; Δ = 0.2 Da.

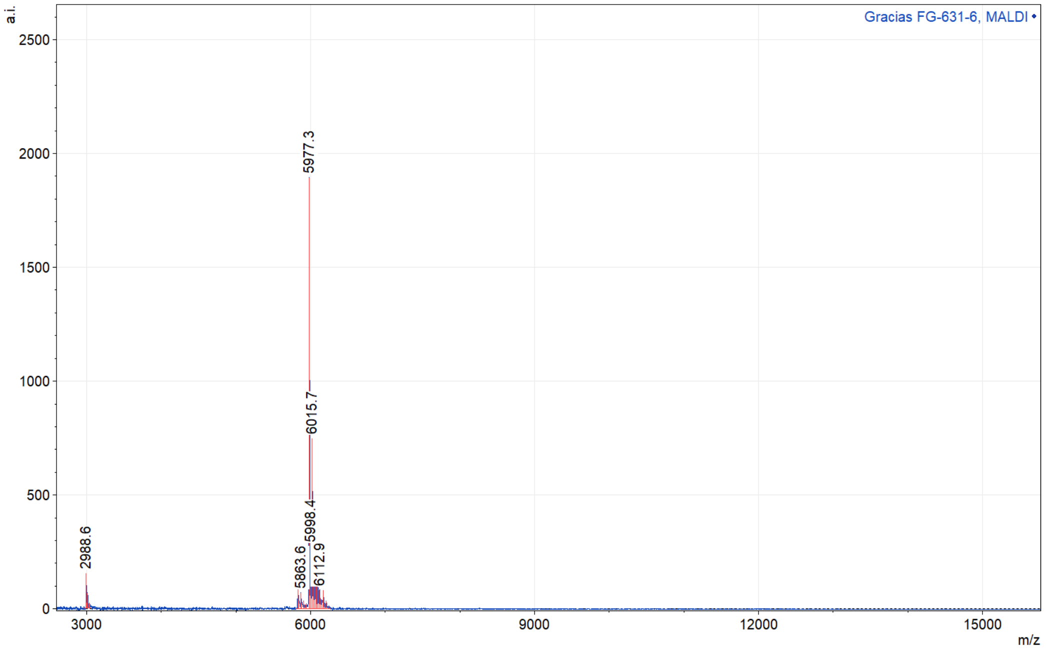

Figure S38. MALDI-TOF MS spectrum of **19ON_C^{E}**: *m/z* calcd for $[M+H]^{+}$: 5975.9 Da; found: 5977.3; Δ = 1.4 Da.

5. X-ray diffraction analysis

Single-crystal diffraction data of **dC^{he}_epA** (**dC^{She}**) and **dC^{hp}_epB** (**dC^{Shp}**) were collected on Xcalibur PX diffractometr with monochromatized Cu$_{K\alpha}$radiation (λ=1.54180 Å) at 180 K. CrysAlisProCCD[25] was used for data collection, cell refinement and data reduction. The structures were solved by direct methods with SIR92[26] and were refined by full-matrix least-squares on F with CRYSTALS.[27] The positional and anisotropic thermal parameters of all non-hydrogen atoms were refined. All hydrogen atoms were located in a difference Fourier map, but those attached to carbon atoms were repositioned geometrically. They were initially refined with soft restraints on the bond lengths and angles to regularise their geometry, then their positions were refined with riding constraints.

Crystal data for dC^{She} (colourless, 0.112 x 0.186 x 0.292 mm):

$C_{11}H_{17}N_3O_5.0.5H_2O$, triclinic, space group $P1$, a = 6.3200(10) Å, b = 9.739(3) Å, c = 11.787(2) Å, α = 114.044(12)°, β = 100.295(13)°, γ = 94.954(16)°, V = 641.6(3) Å^3, Z = 2, M = 560.56, 14217 reflections measured, 4416 independent reflections. Final R = 0.033, wR = 0.039, GoF = 1.108 for 4371 reflections with $I > 2\sigma(I)$ and 354 parameters. Flack parameter x = 0.13(13). The asymmetric unit contains two crystallographically independent molecules of **dC^{She}** and one water molecule. CCDC 2166141.

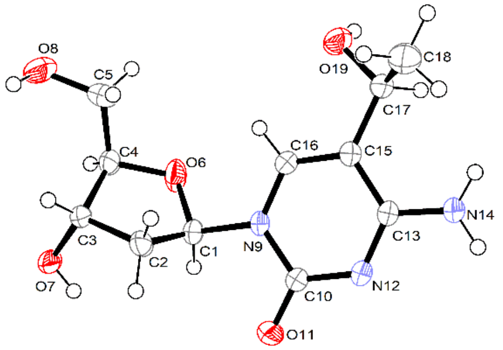

Figure S39. Crystal structure of **dC^{She}**.

Crystal data for dC^{Shp} (colourless, 0.204 x 0.244 x 0.547 mm):

$C_{12}H_{19}N_3O_5$, triclinic, space group *P*1, *a* = 7.6129(6) Å, *b* = 9.1399(7) Å, *c* = 11.2377(9) Å, α = 91.092(2)°, *β* = 103.921(2)°, γ = 112.0703(19)°, *V* = 698.12(10)Å^3, *Z* = 2, *M* = 570.60, 19653 reflections measured, 4807 independent reflections. Final *R* = 0.026, *wR* = 0.031, *GoF* = 0.880 for 4786 reflections with $I > 2\sigma(I)$ and 363 parameters. Flack parameter *x* = 0.03(9). The asymmetric unit contains two crystallographically independent molecules of **dC^{Shp}**. CCDC 2166142.

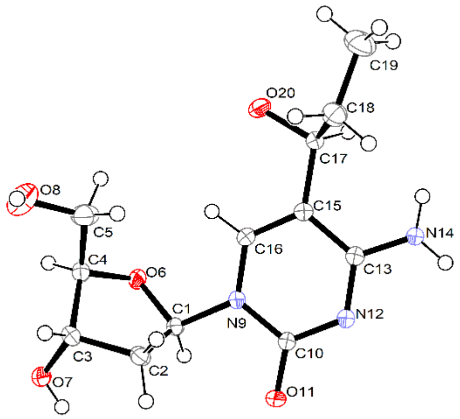

Figure S40. Crystal structure of **dC^{Shp}**.

6. Copies of NMR spectra

6.1. NMR spectra for full characterization

^{1}H and ^{13}C NMR spectra of **dU^{pr}**

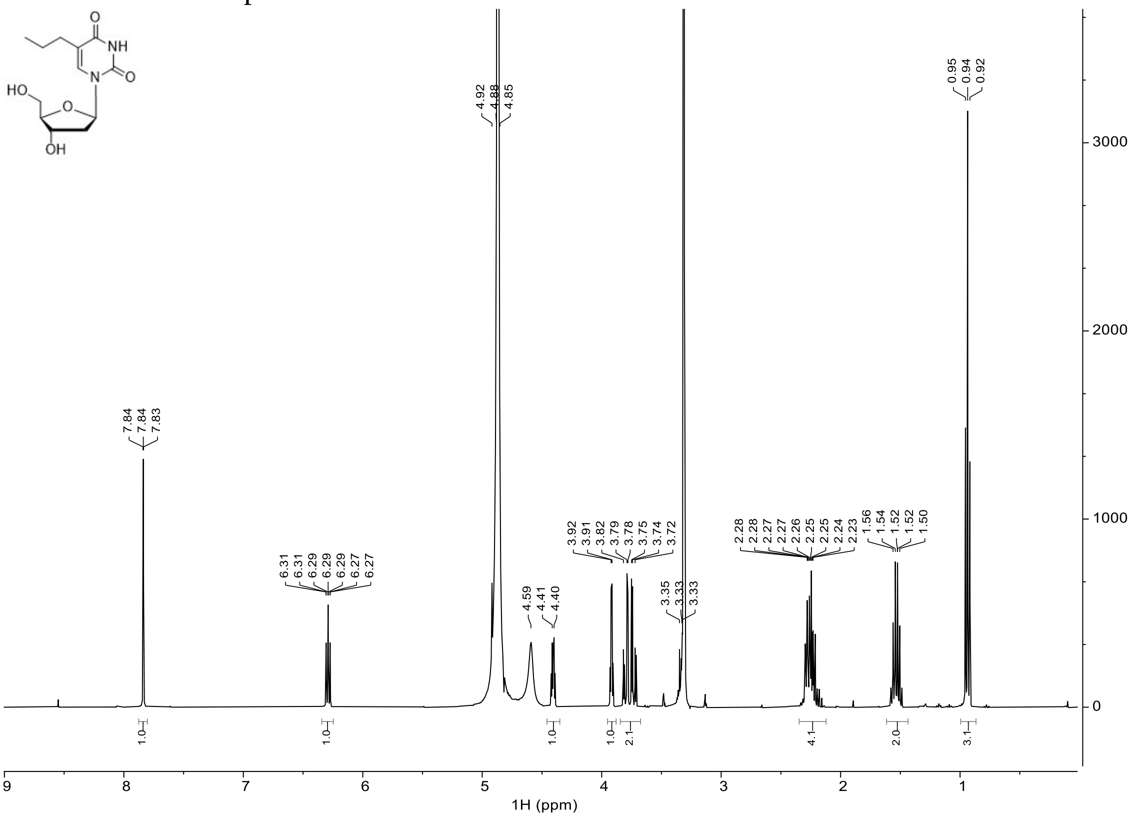

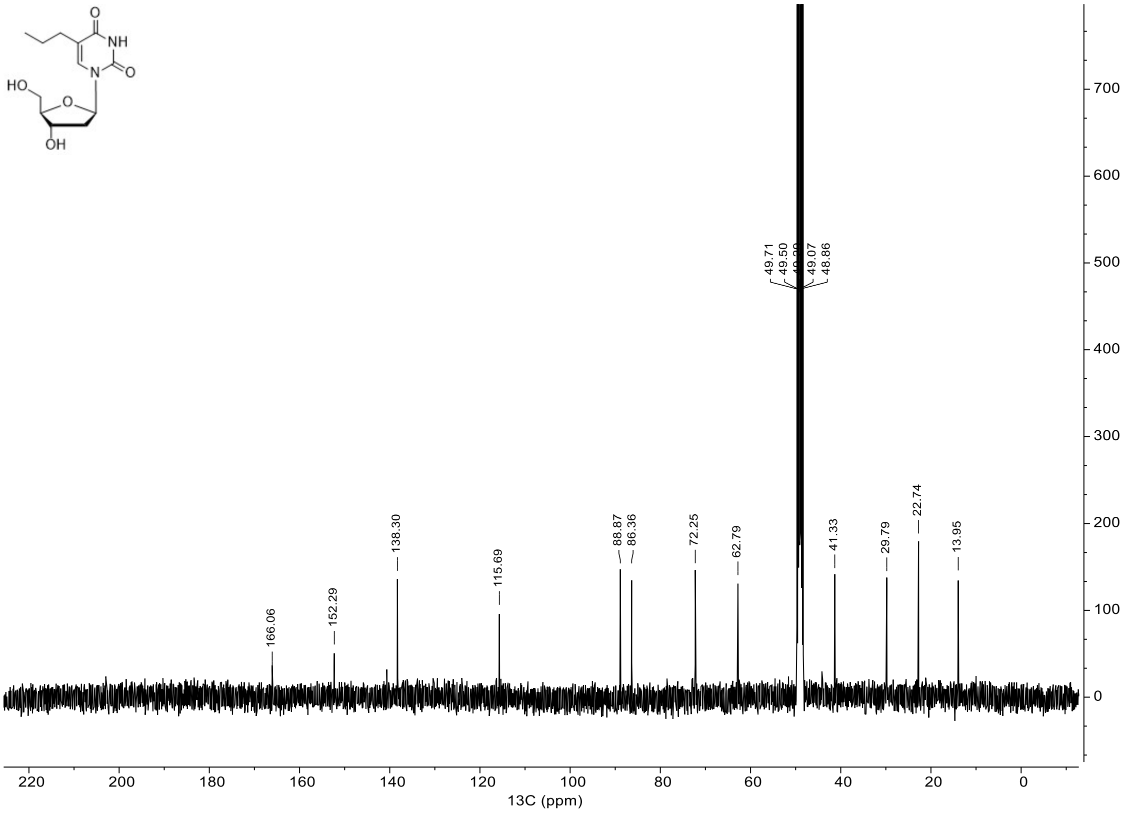

^{1}H and ^{13}C NMR spectra of **dC^pr^**

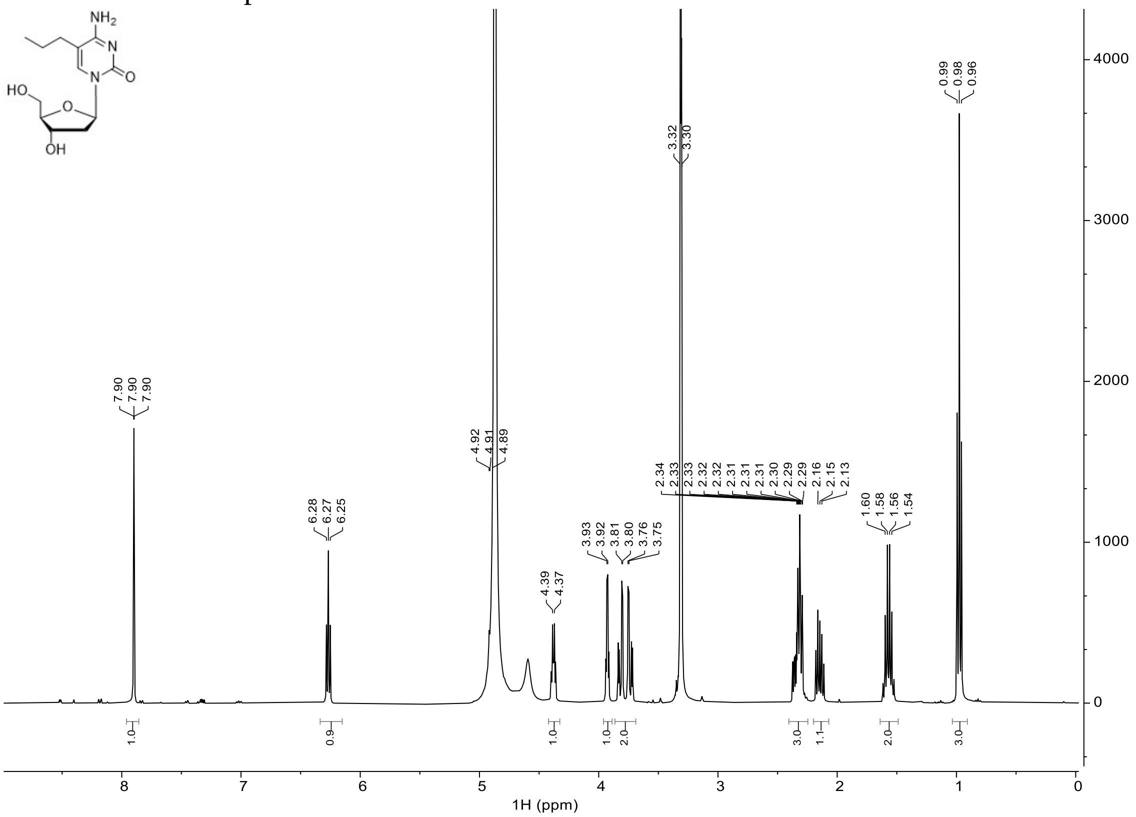

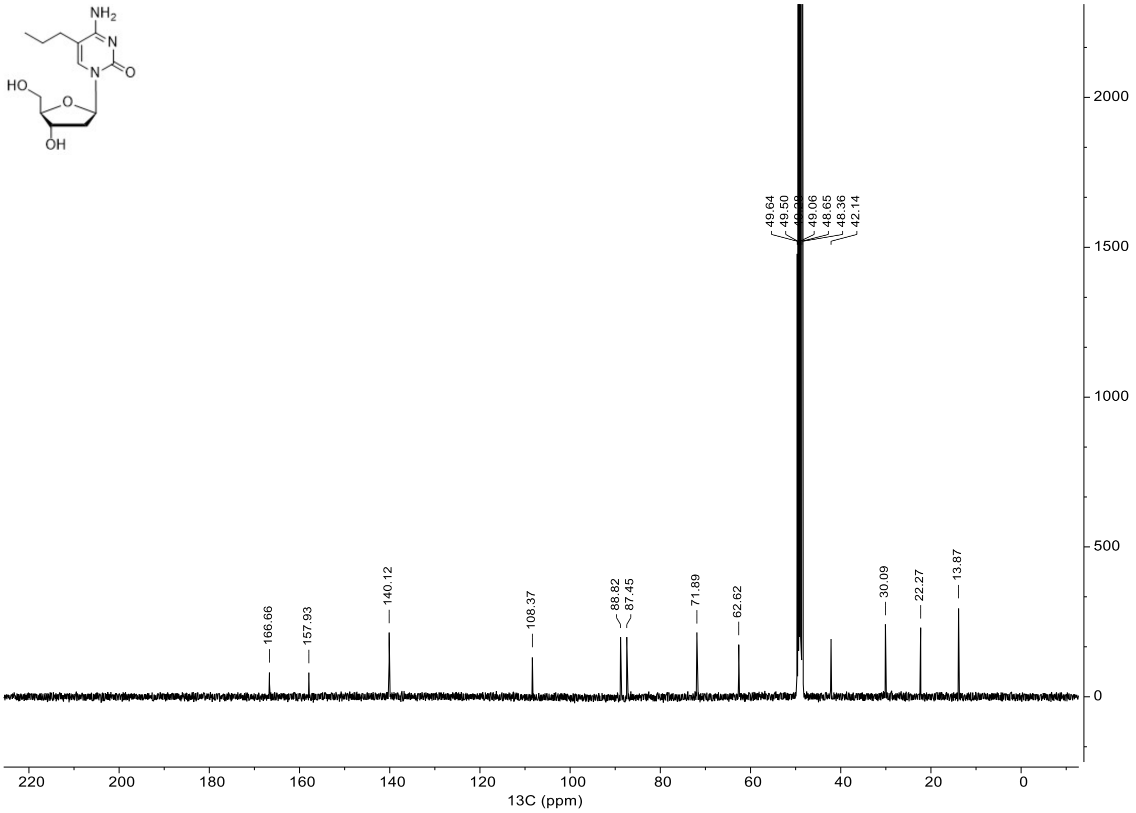

^{1}H and ^{13}C NMR spectra of **3**

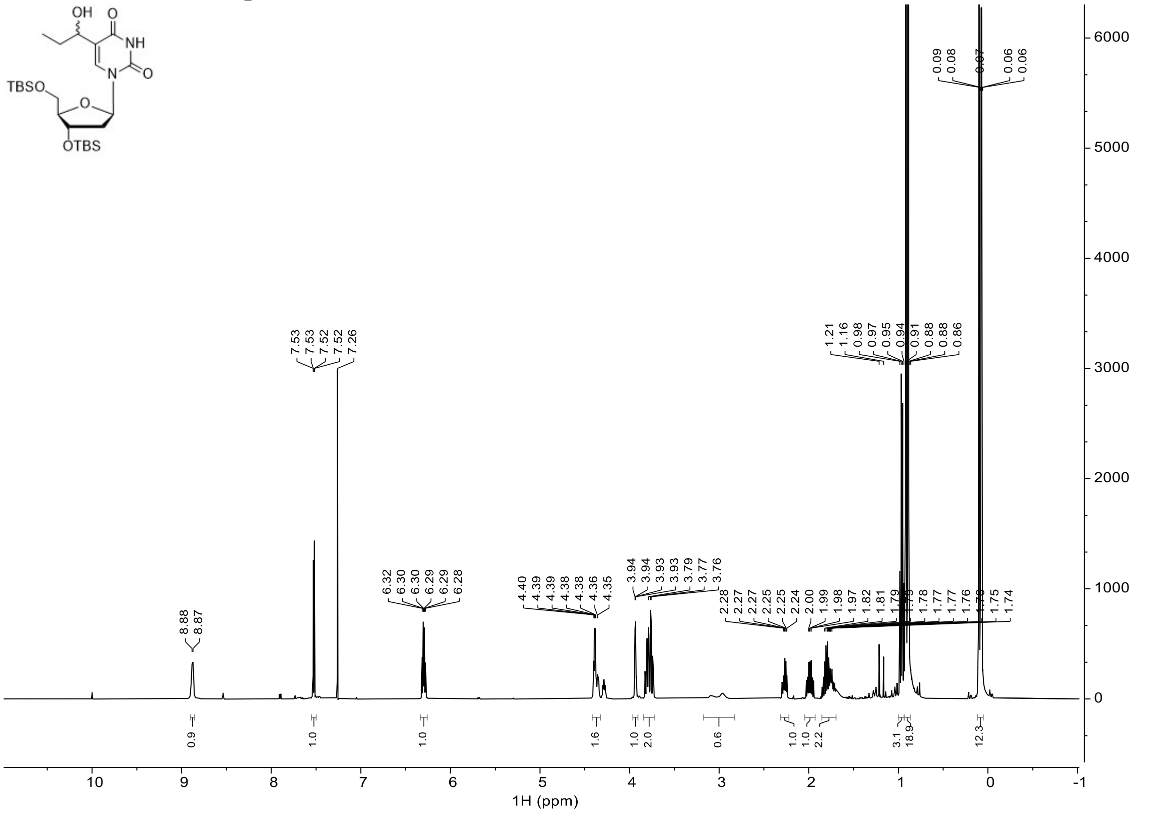

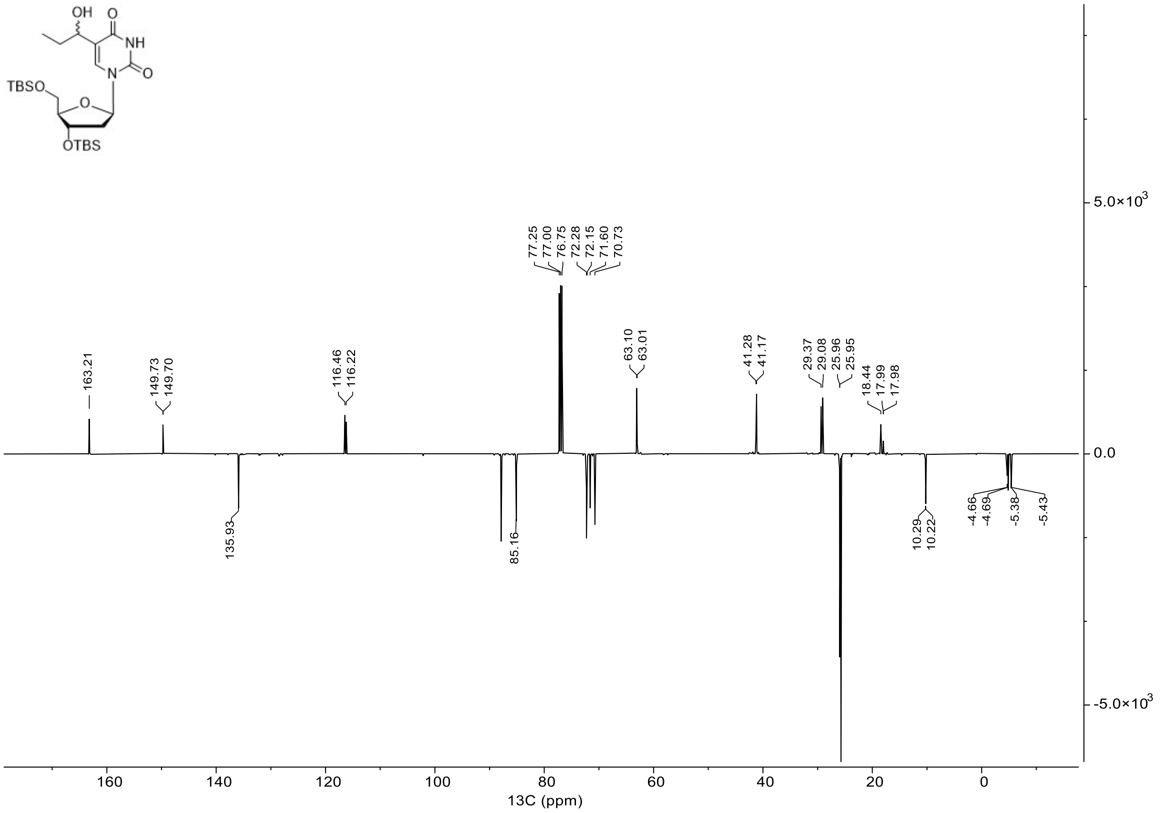

^{1}H and ^{13}C NMR spectra of **4**

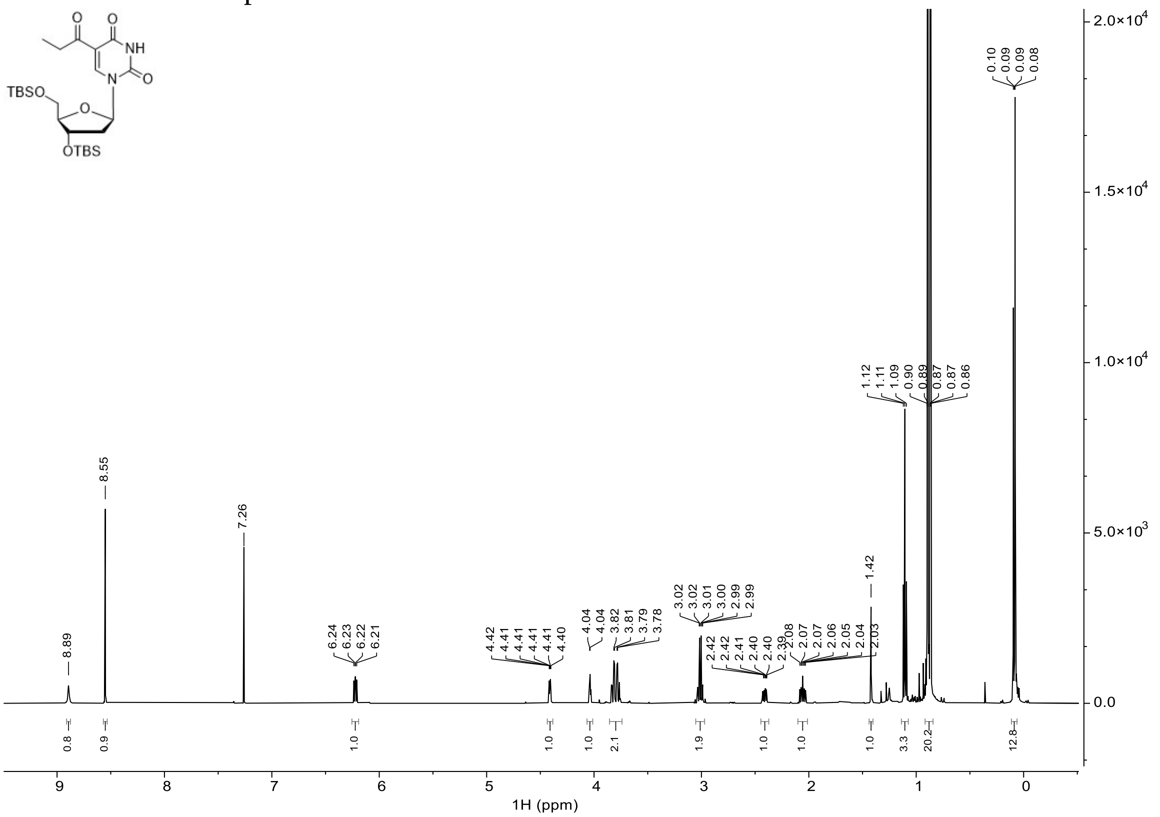

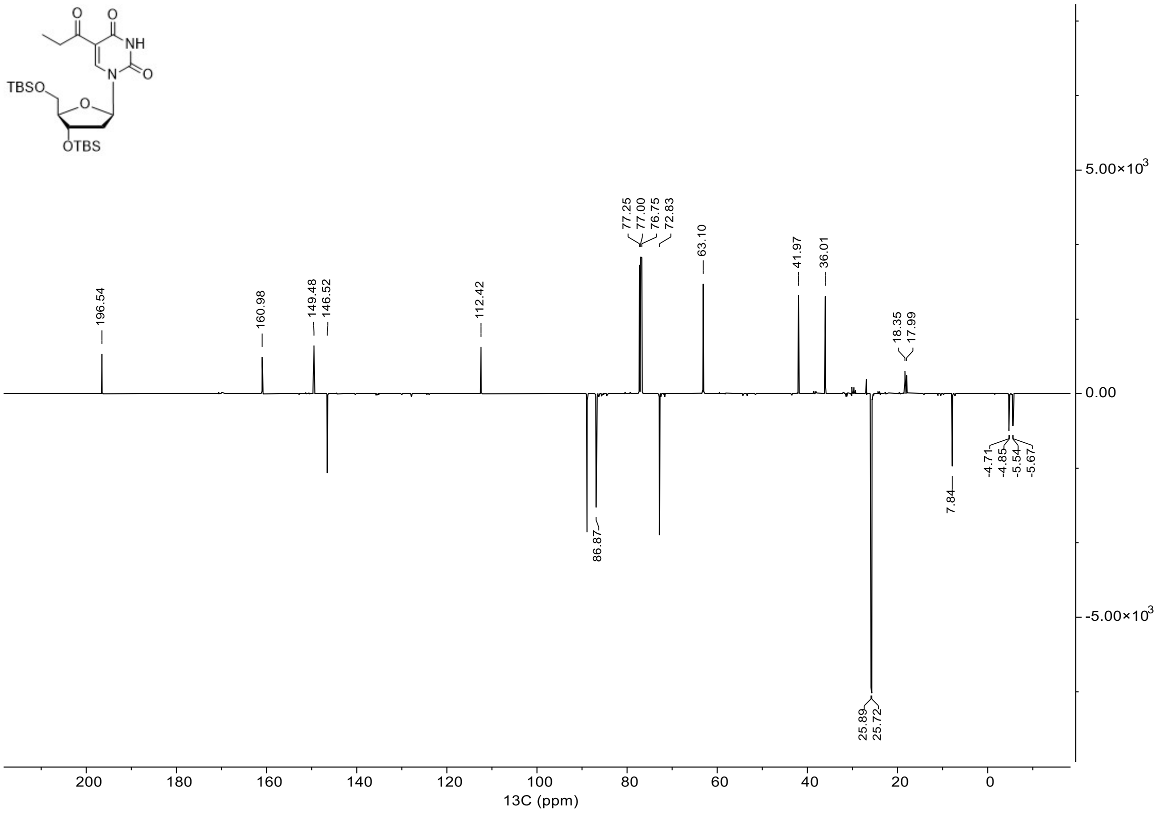

^{1}H and ^{13}C NMR spectra of **5**

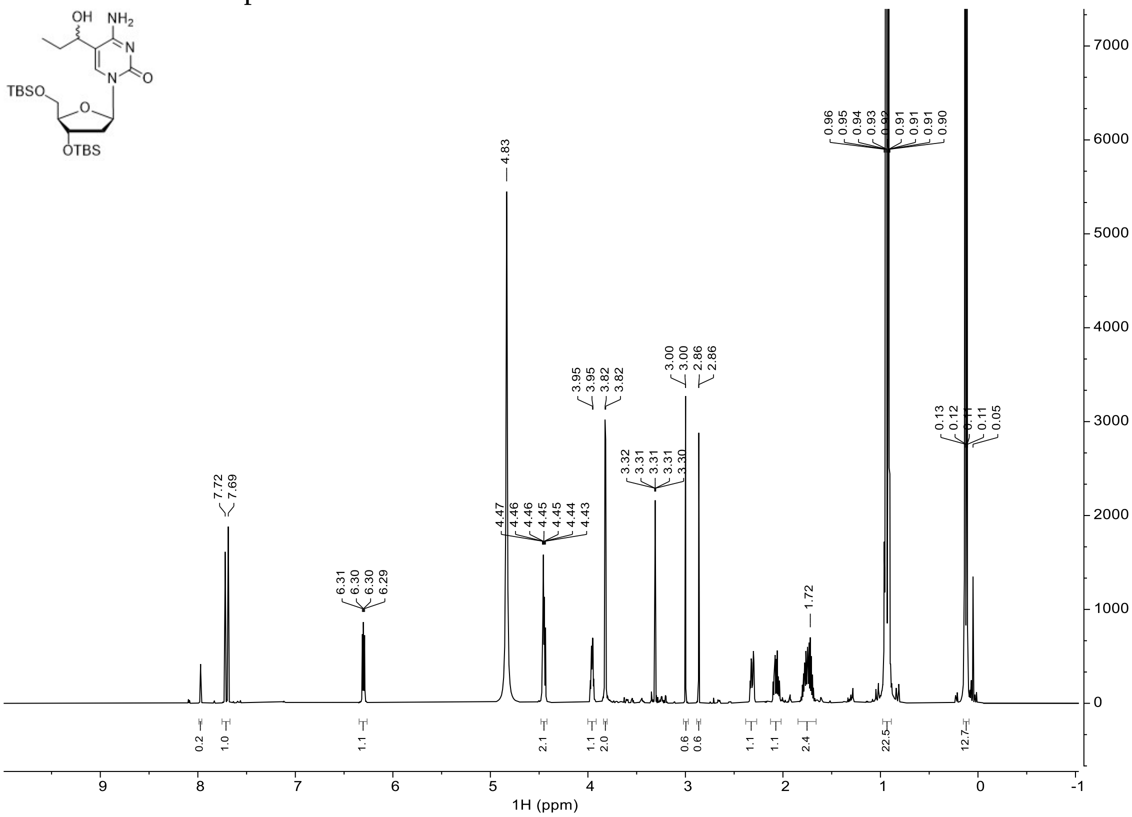

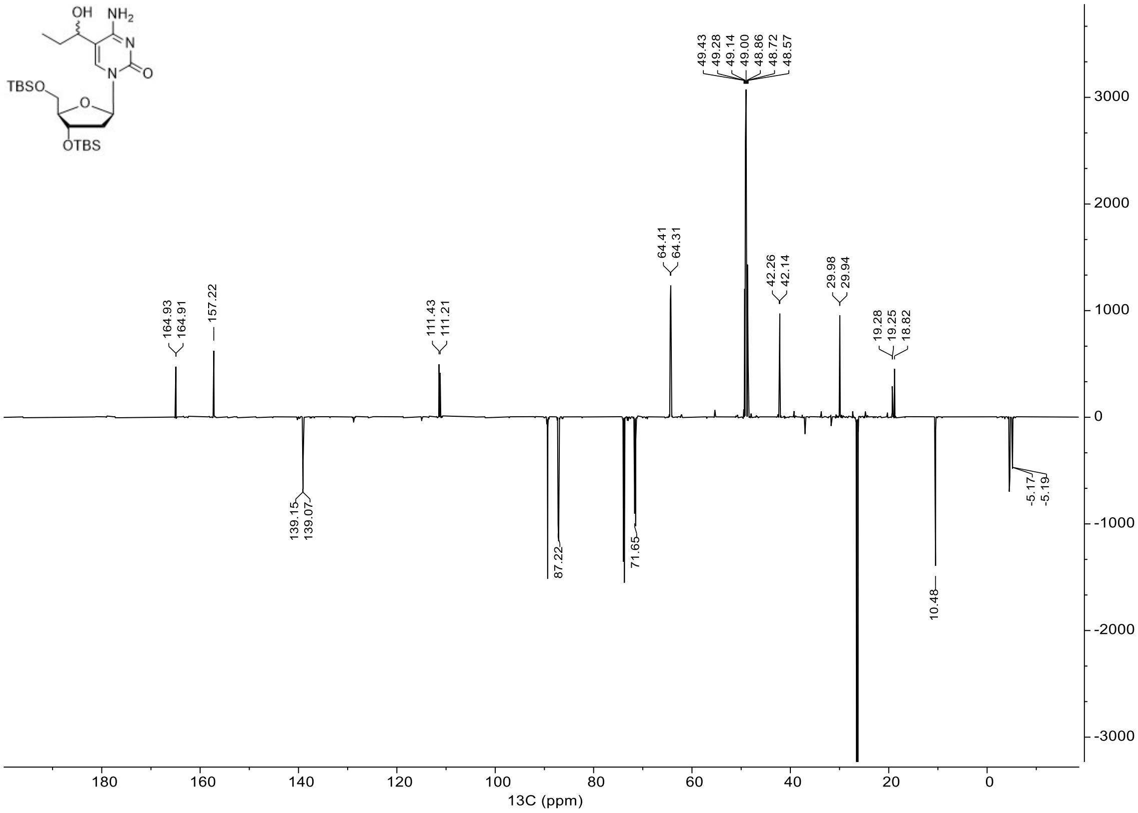

^{1}H and ^{13}C NMR spectra of **6**

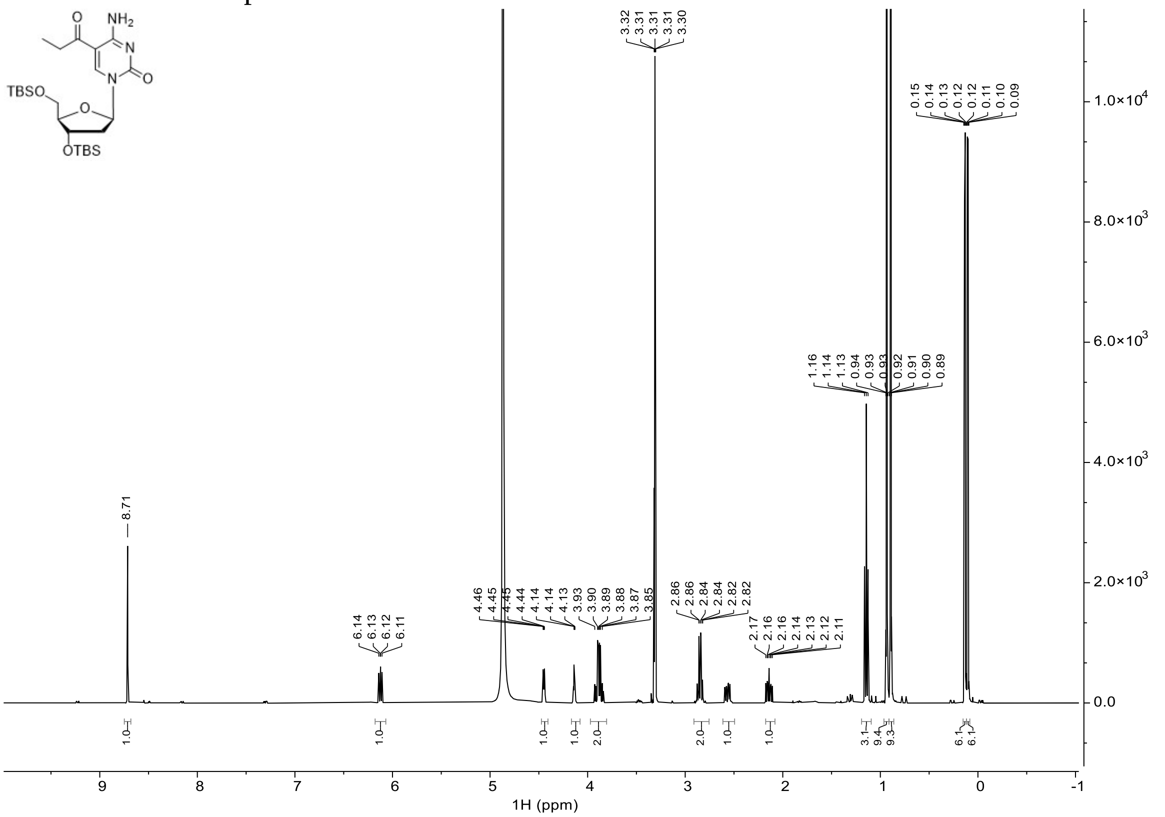

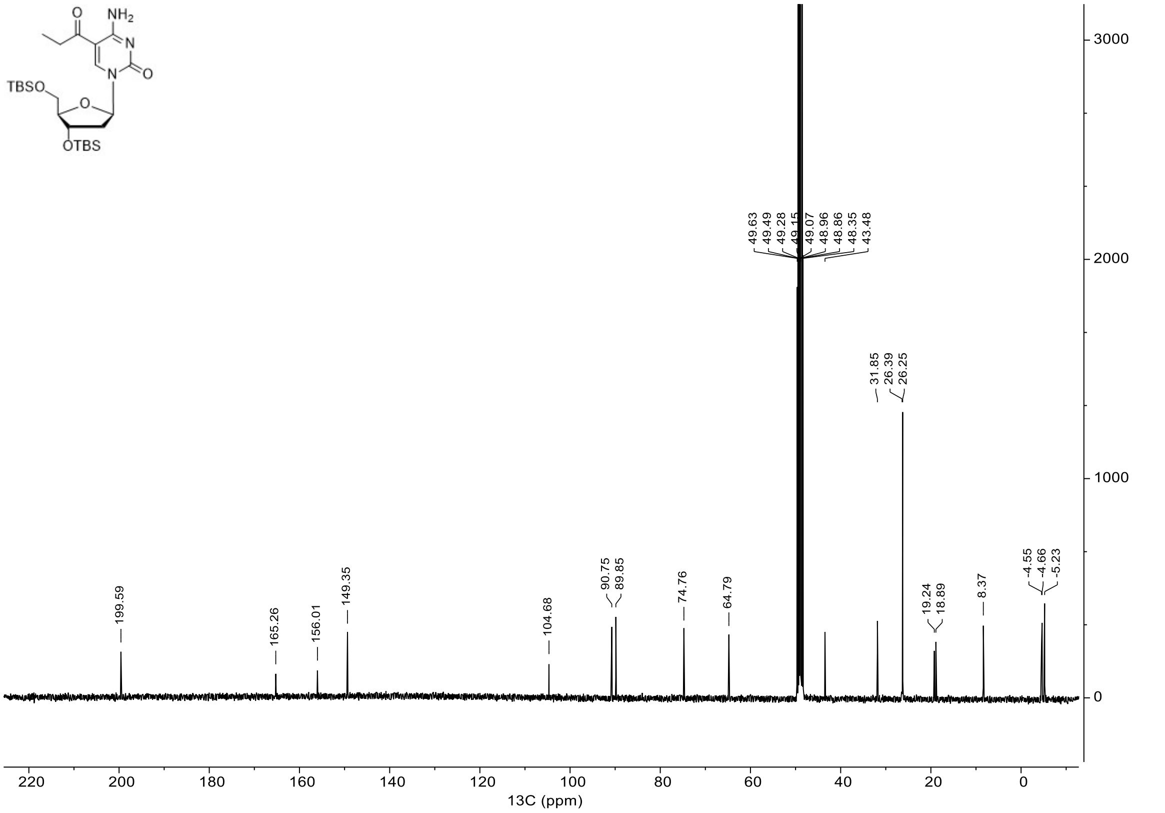

^{1}H and ^{13}C NMR spectra of **dU^{hp}**

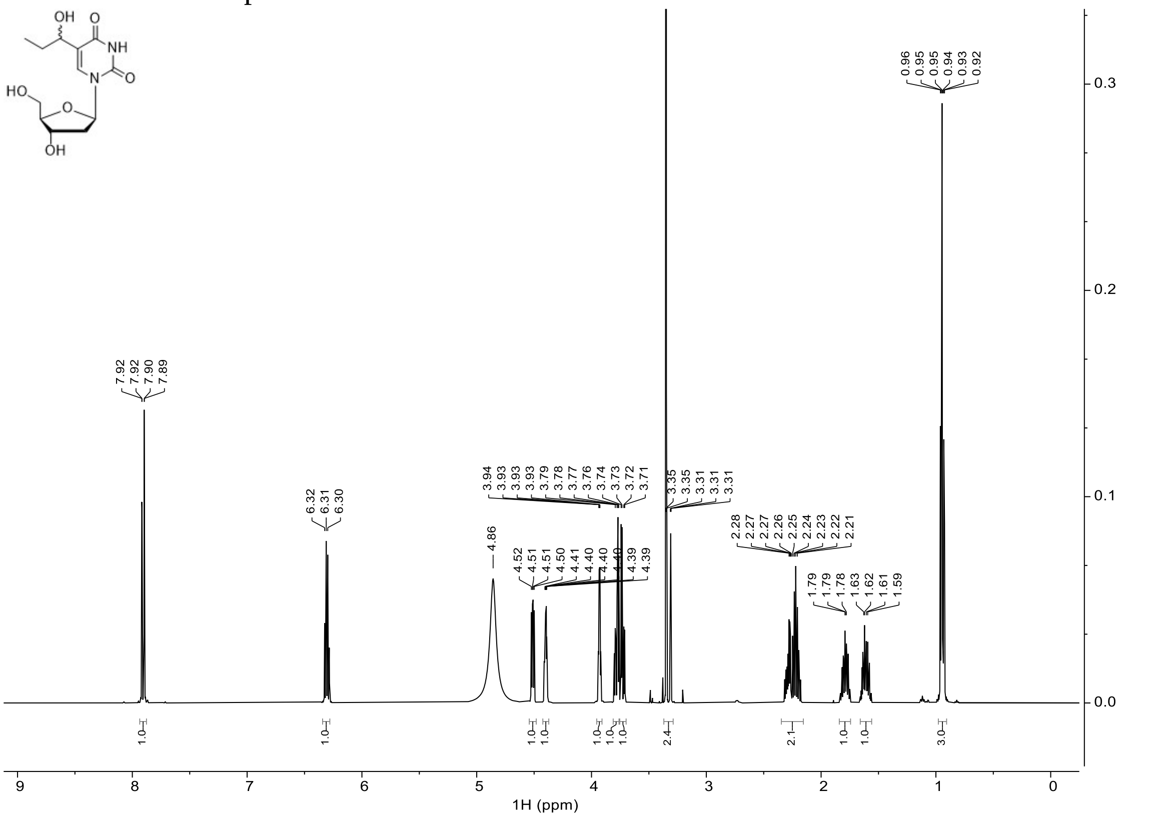

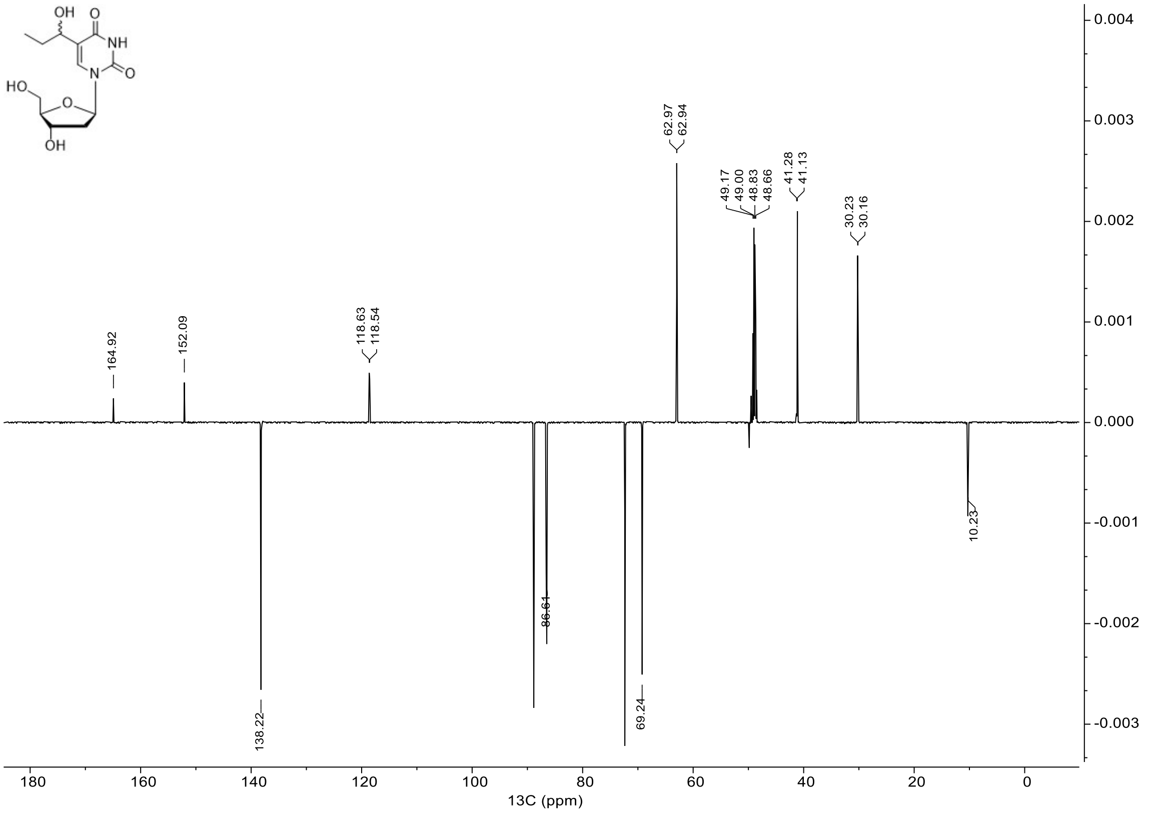

^{1}H and ^{13}C NMR spectra of **dU[pp]**

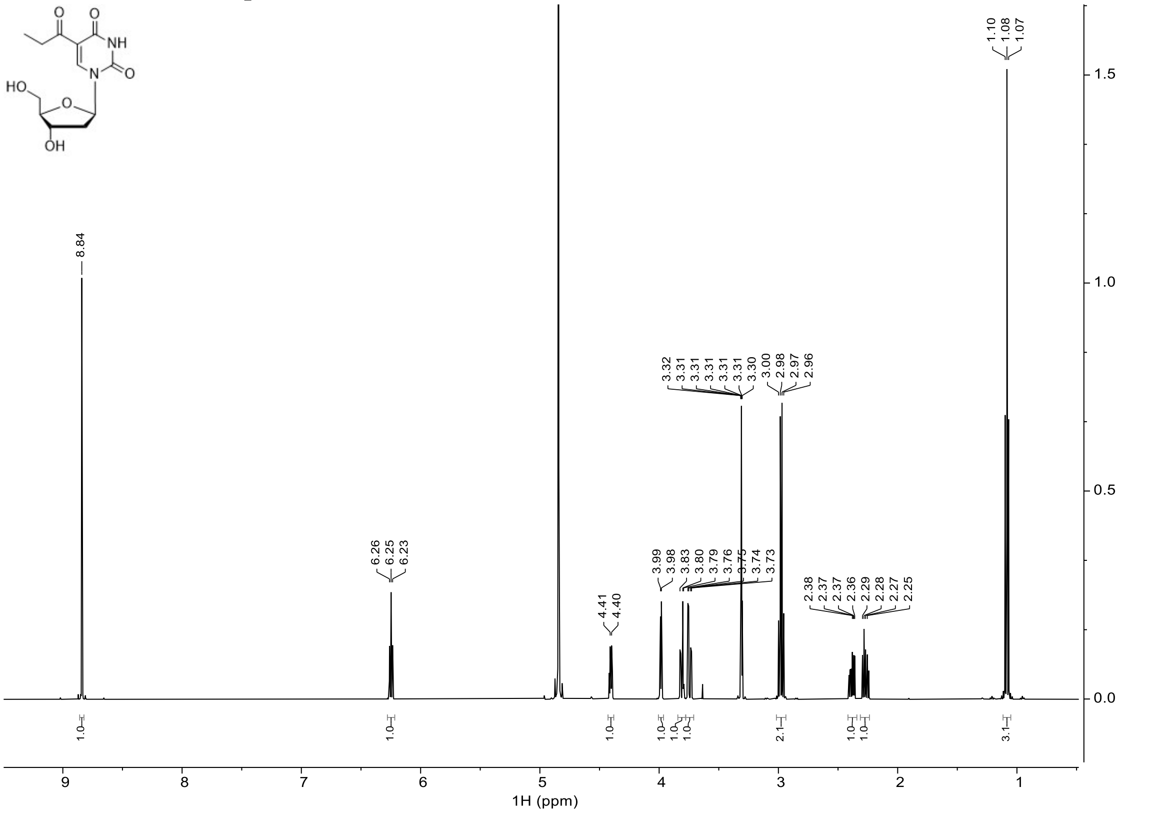

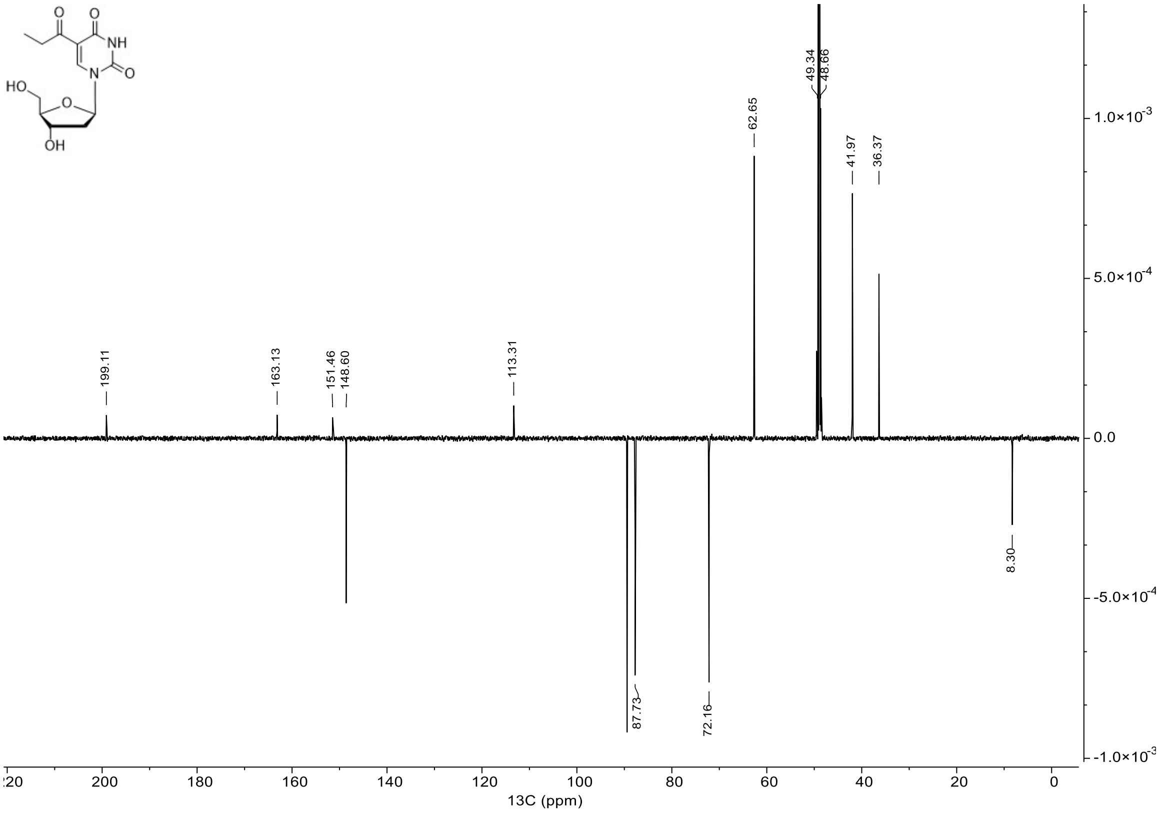

^{1}H and ^{13}C NMR spectra of **dC^{hp}**

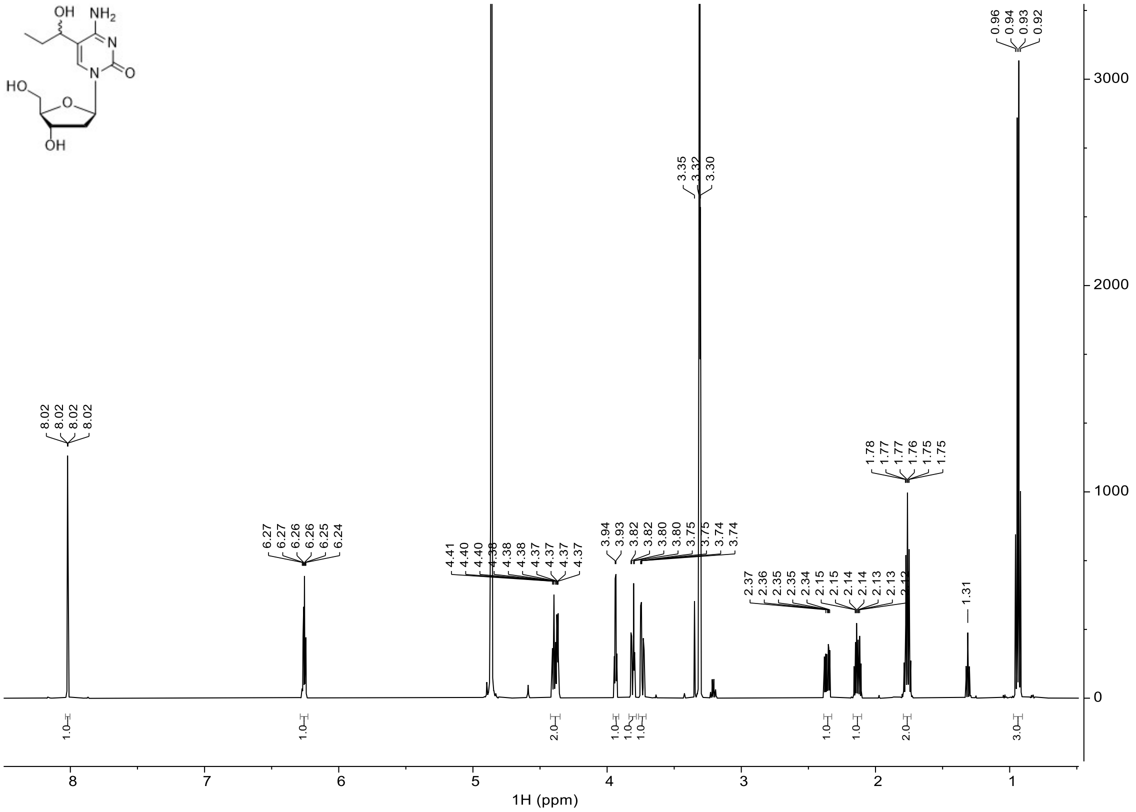

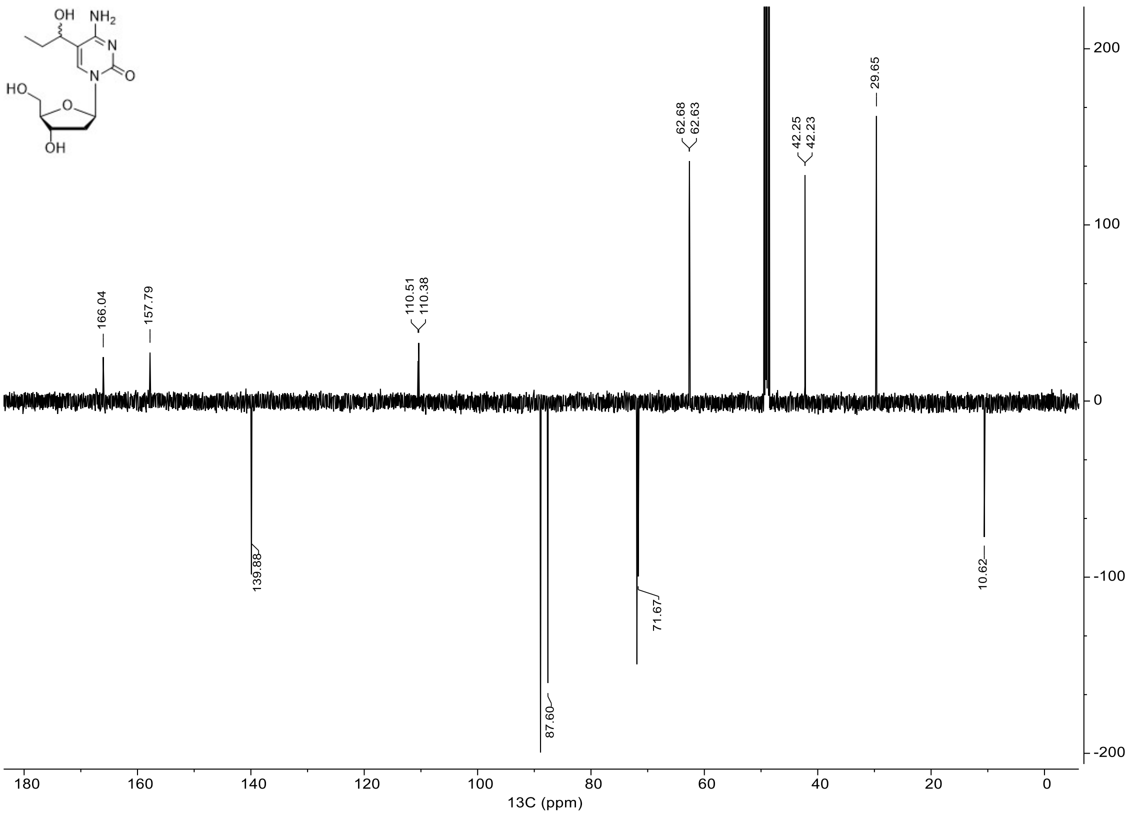

^{1}H and ^{13}C NMR spectra of **dC^pp^**

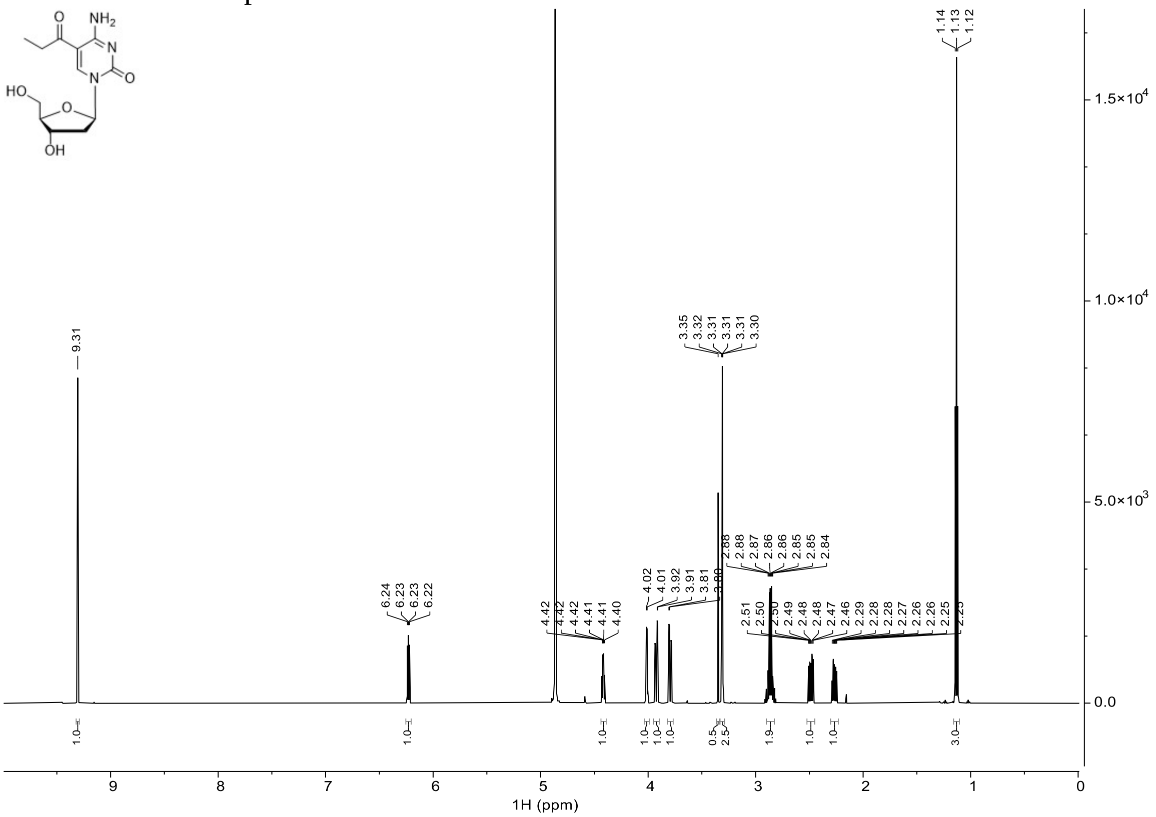

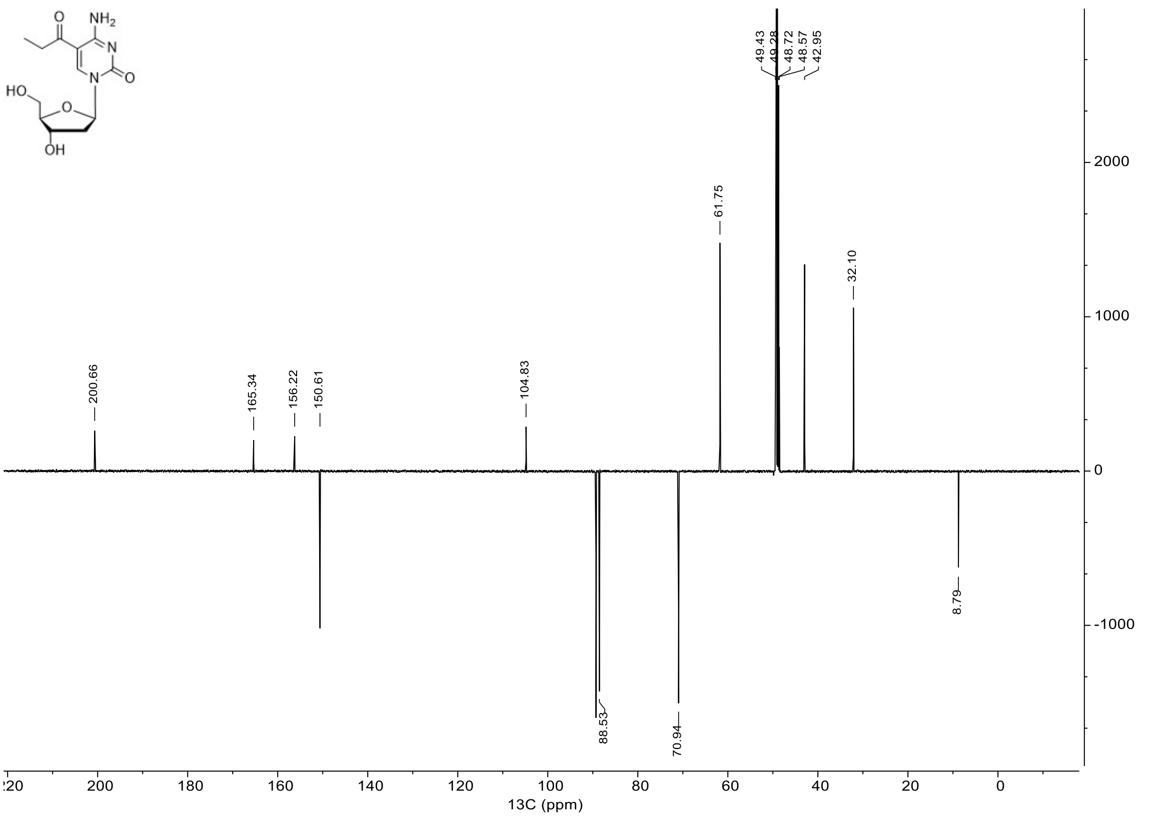

^{1}H, ^{13}C and ^{31}P NMR spectra of **dU^{et}TP**

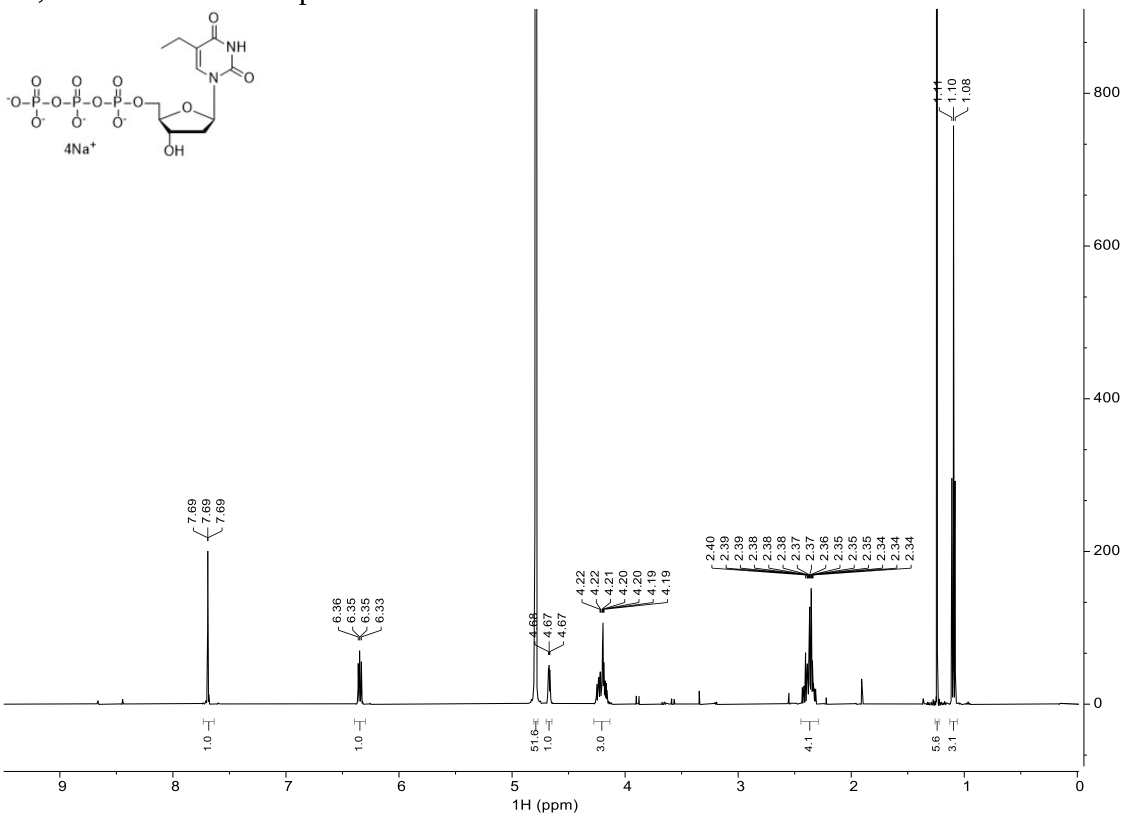

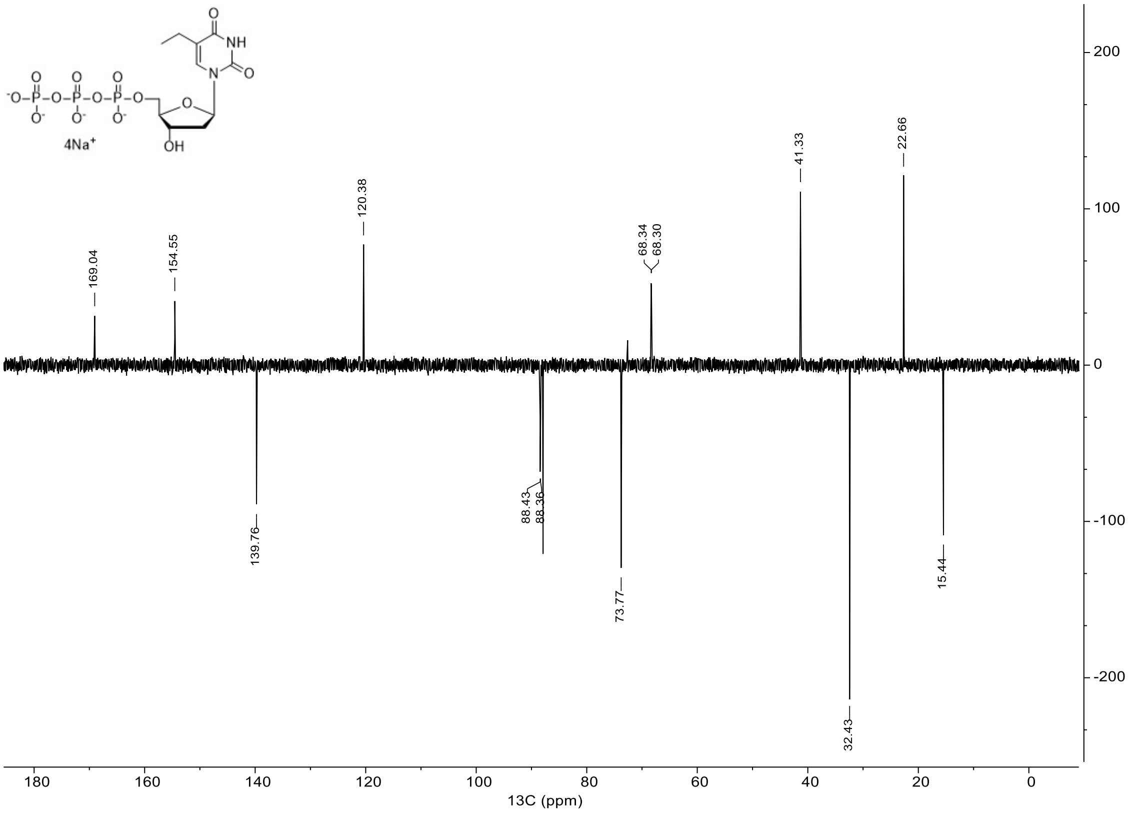

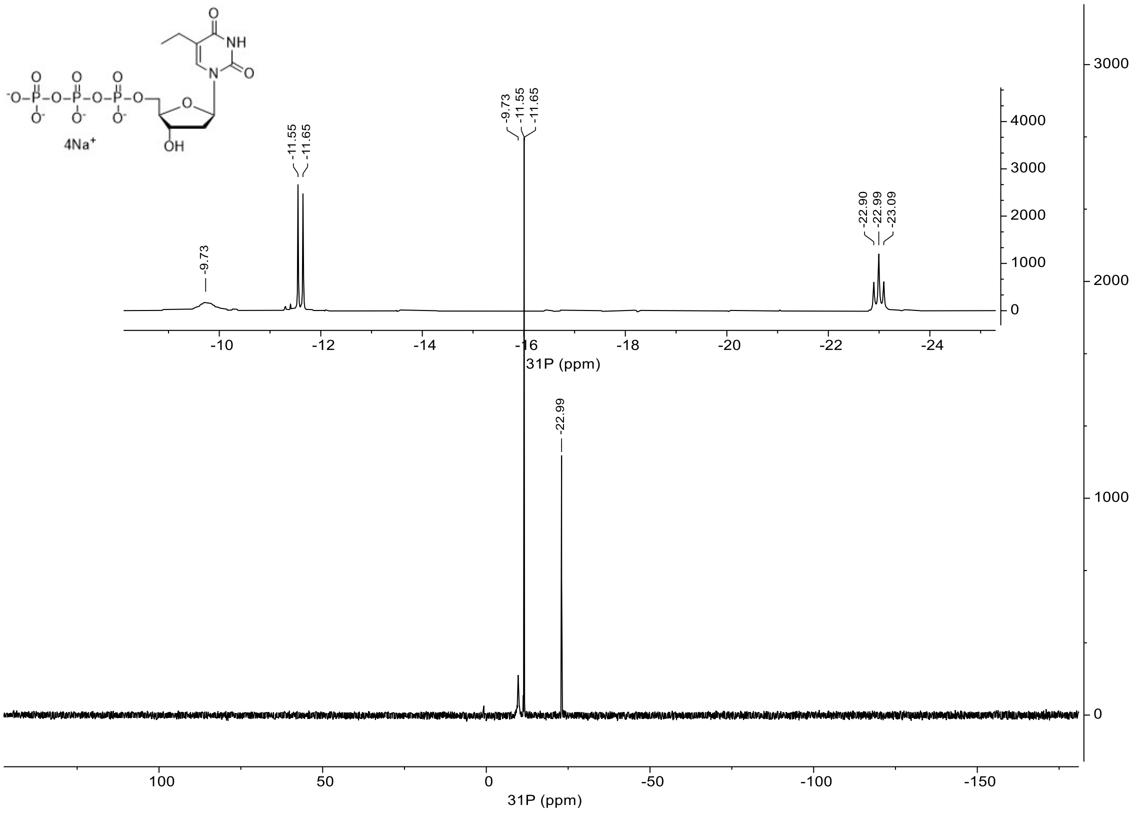

^{1}H, ^{13}C and ^{31}P NMR spectra of **dU^{ac}TP**

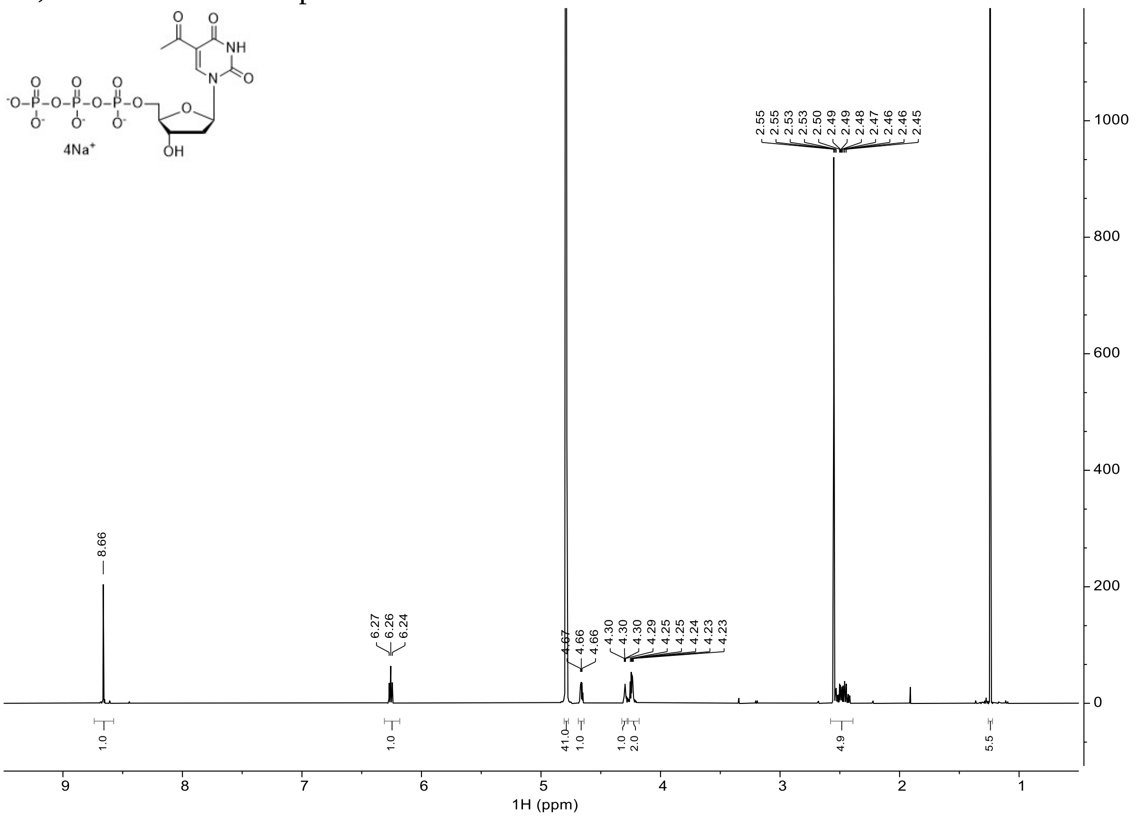

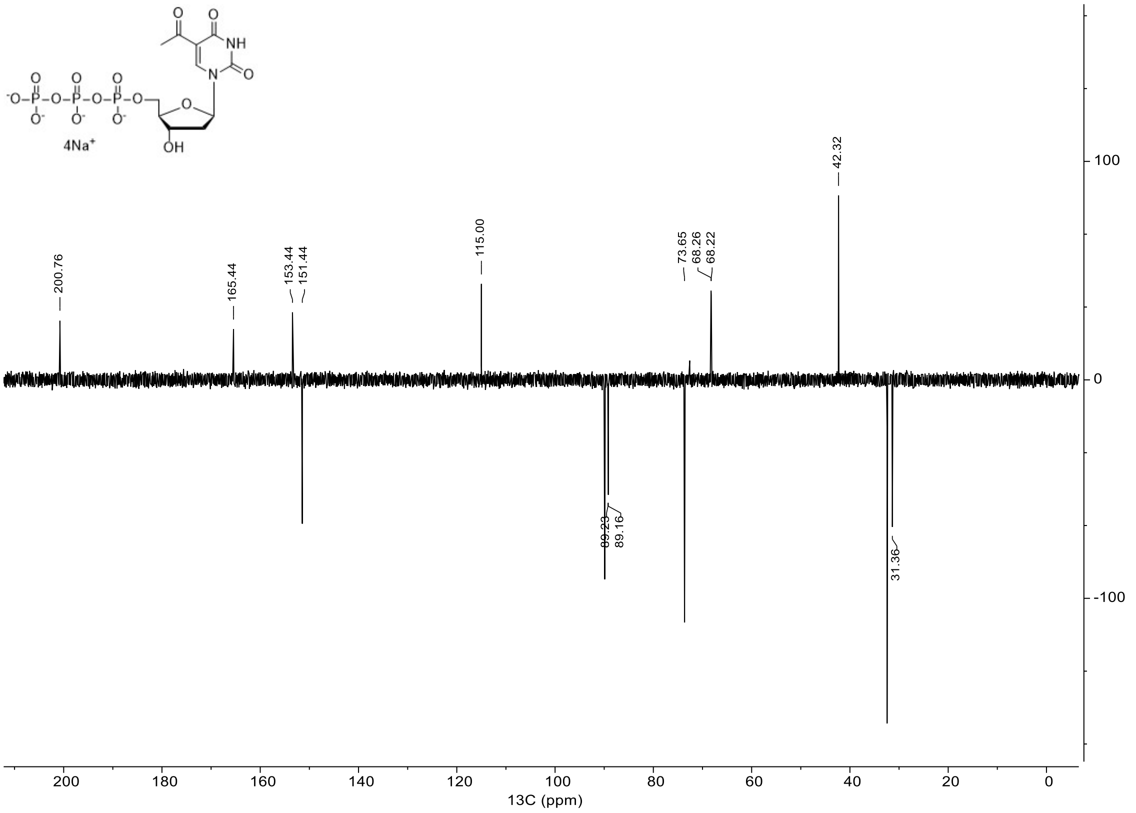
4Na+
OH
200.76
165.44
153.44
151.44
115.00
73.65
68.26
68.22
42.32
89.23
89.16
31.36
13C (ppm)

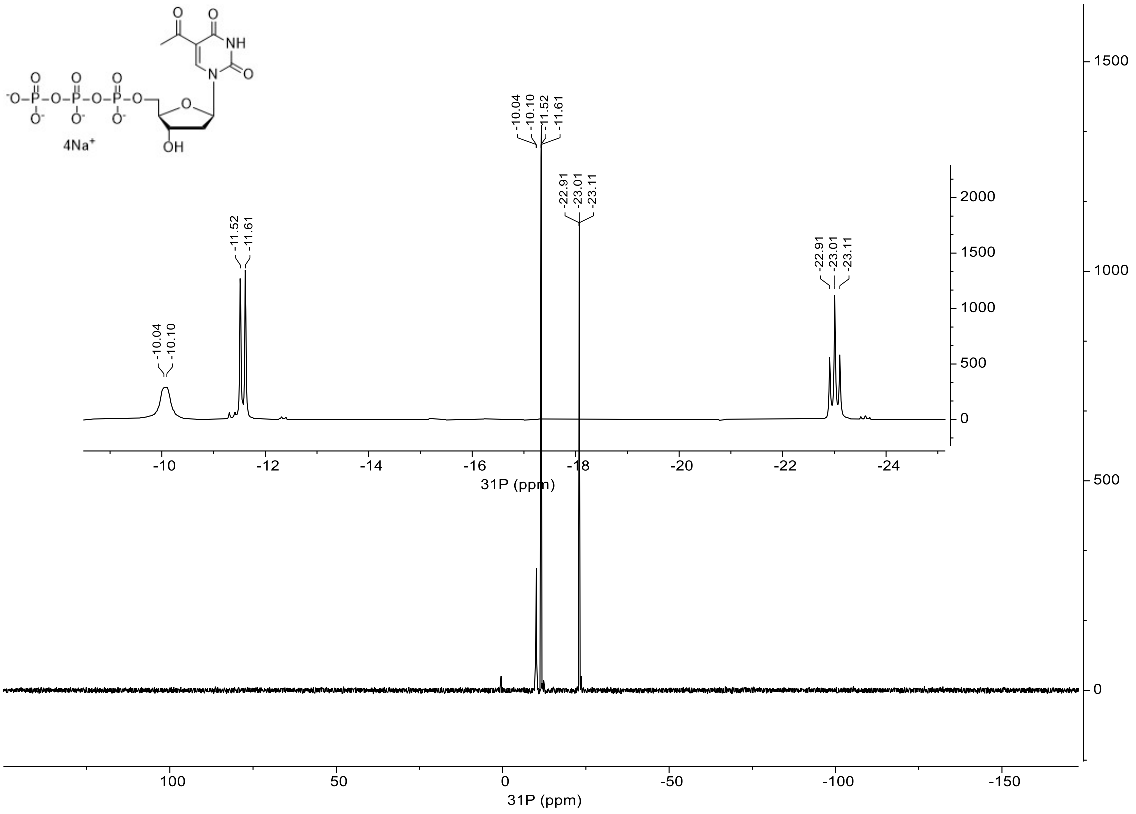
4Na+
OH
-10.04
-10.10
-11.52
-11.61
-22.91
-23.01
-23.11
31P (ppm)
31P (ppm)

^{1}H, ^{13}C and ^{31}P NMR spectra of **dC^{et}TP**

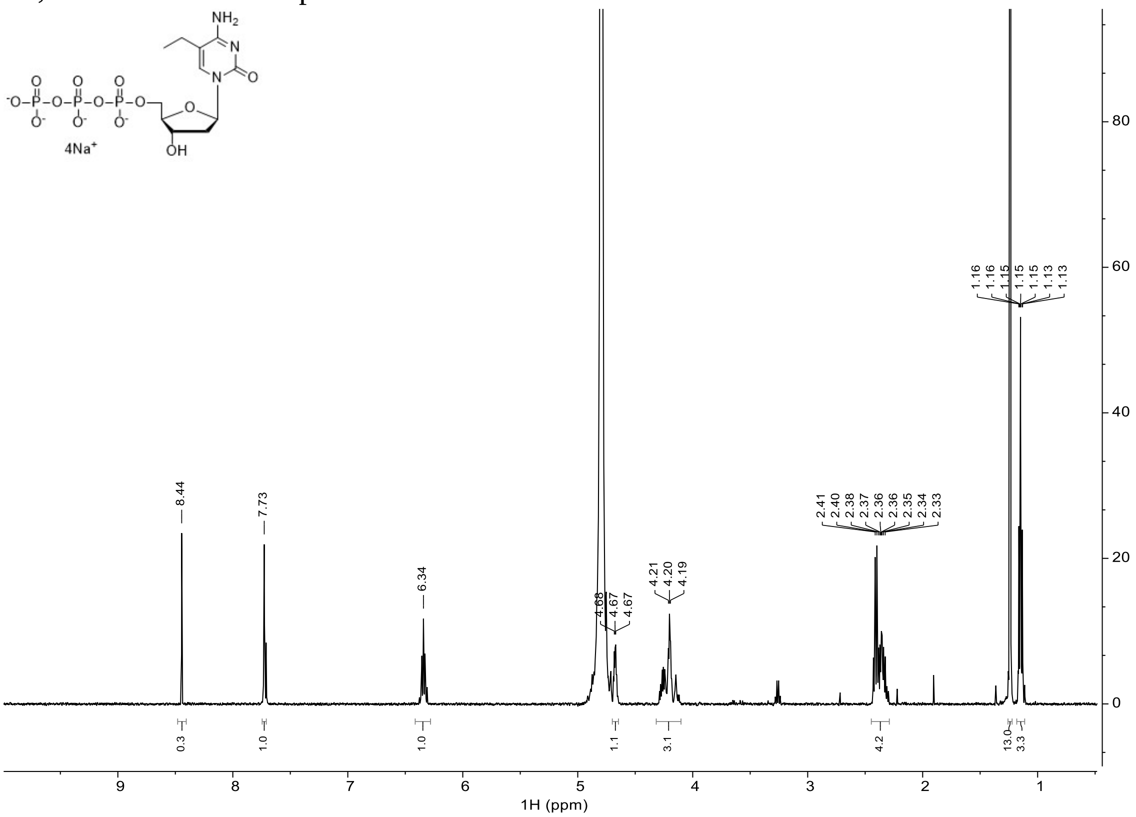

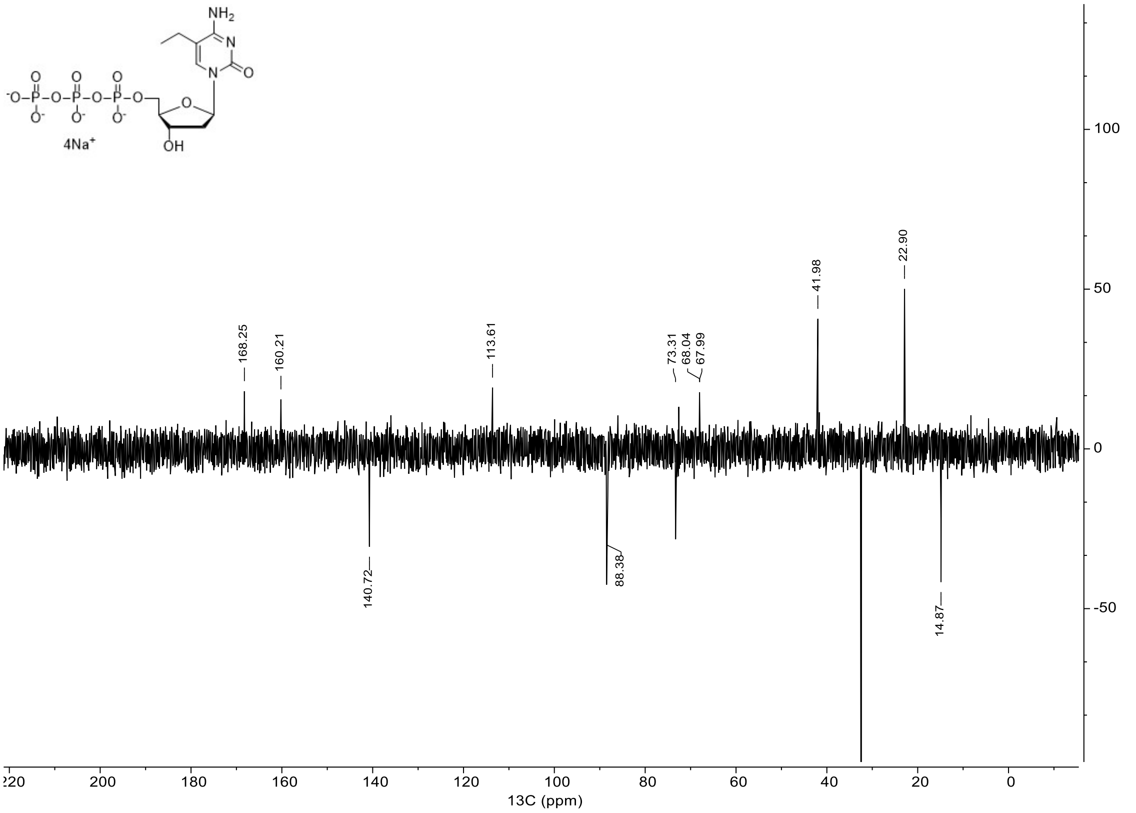

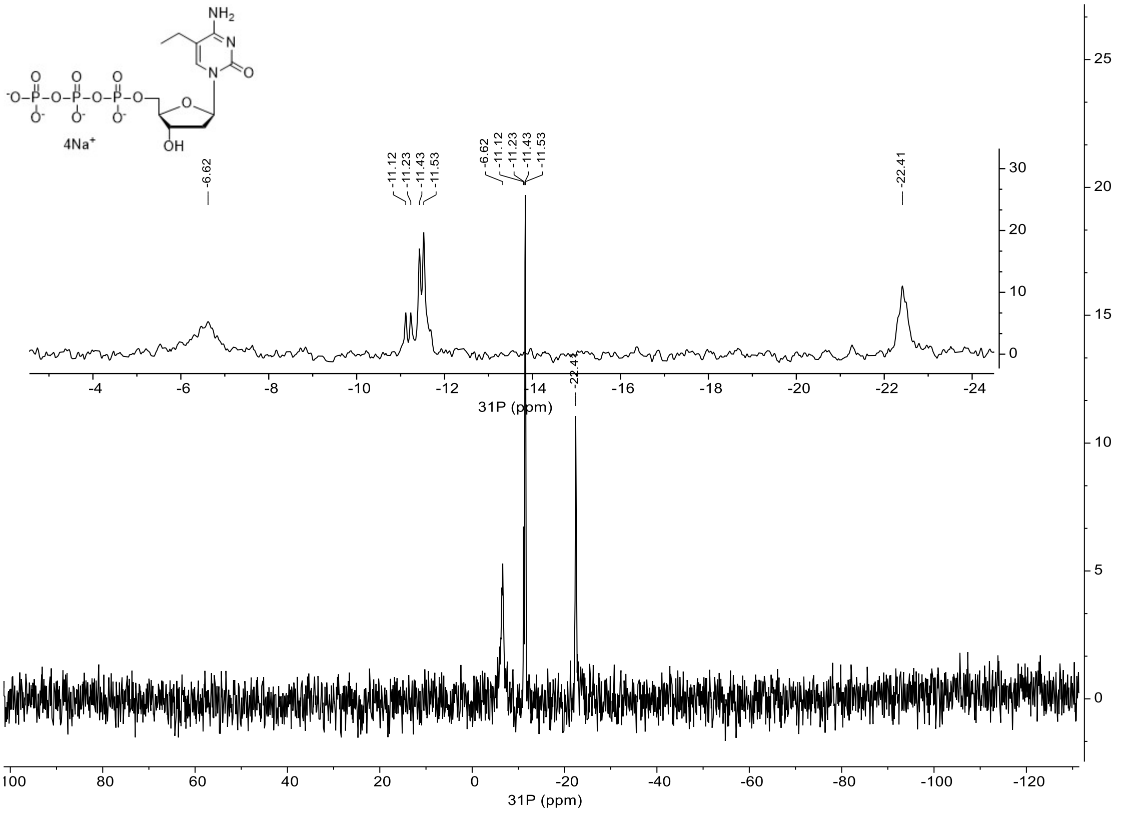

^{1}H, ^{13}C and ^{31}P NMR spectra of **dC^{ac}TP**

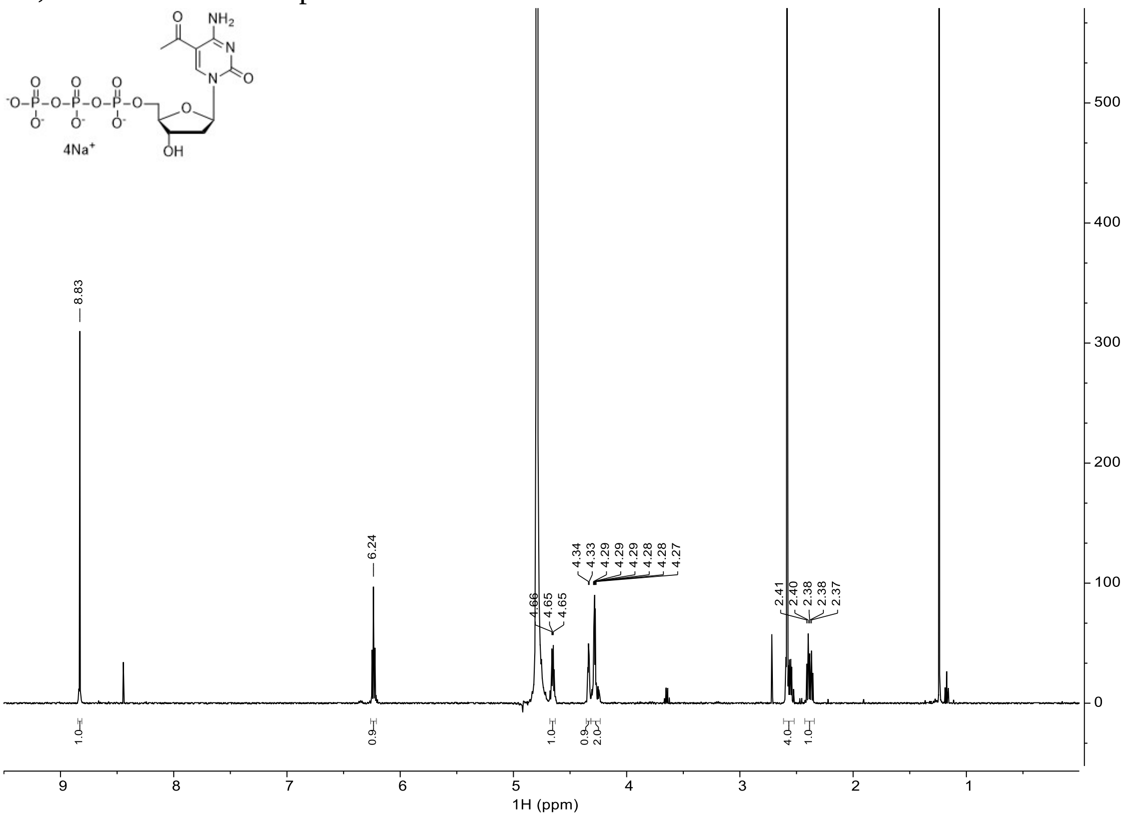

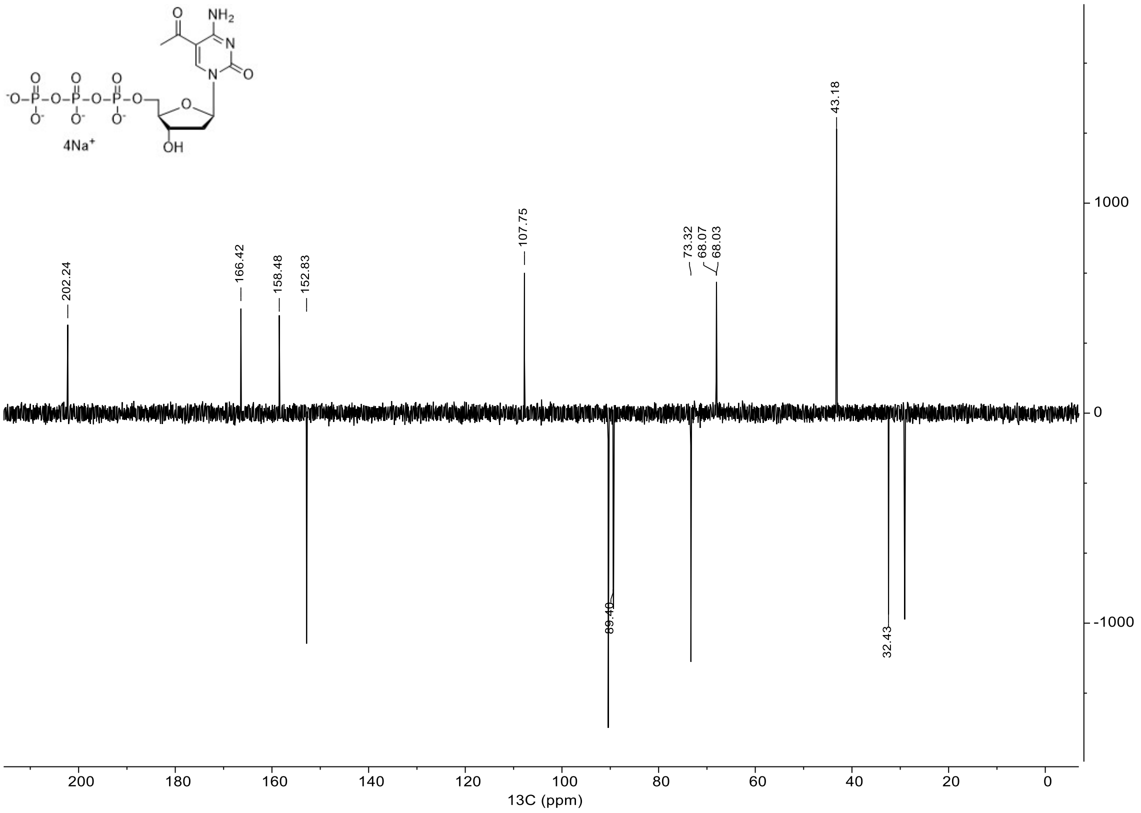

NH2
O
N
4Na+
OH
202.24
166.42
158.48
152.83
107.75
73.32
68.07
68.03
43.18
89.40
32.43
1000
0
-1000
13C (ppm)

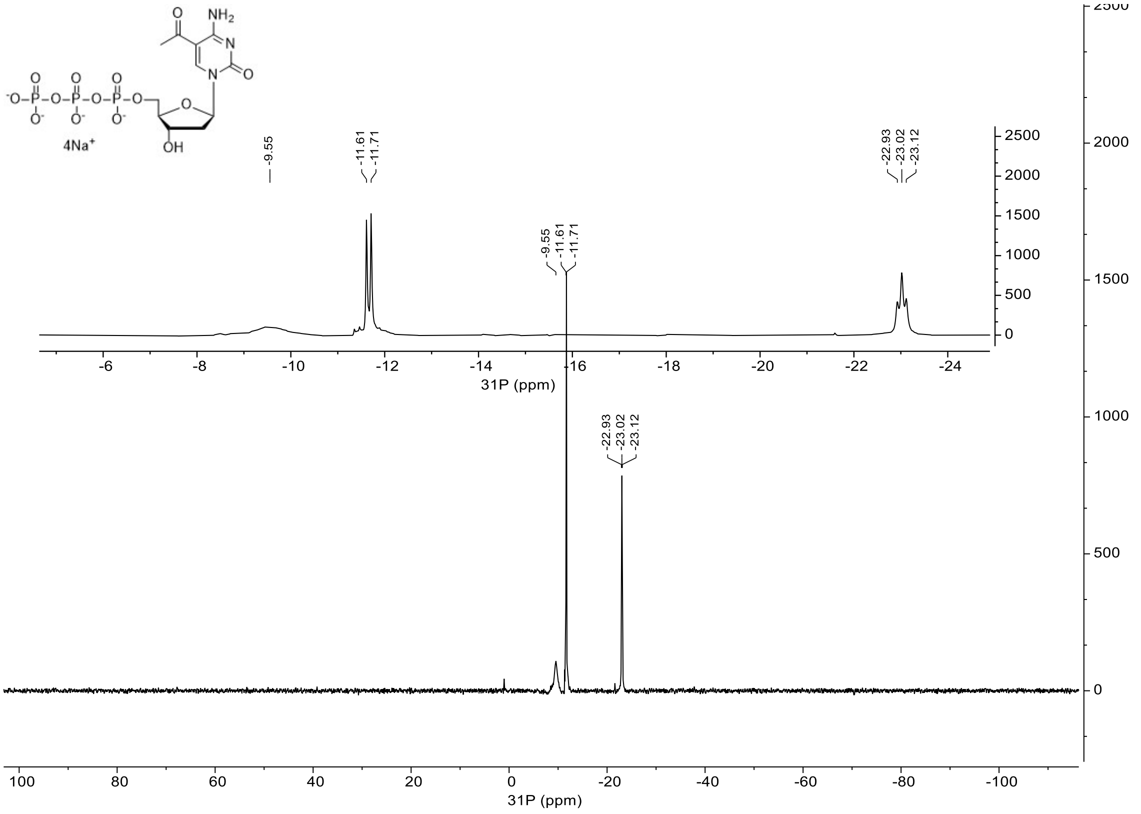

NH2
4Na+
OH
-9.55
-11.61
-11.71
-22.93
-23.02
-23.12
31P (ppm)

^{1}H, ^{13}C and ^{31}P NMR spectra of **dU$^{\mathbf{Rhe}}$TP**

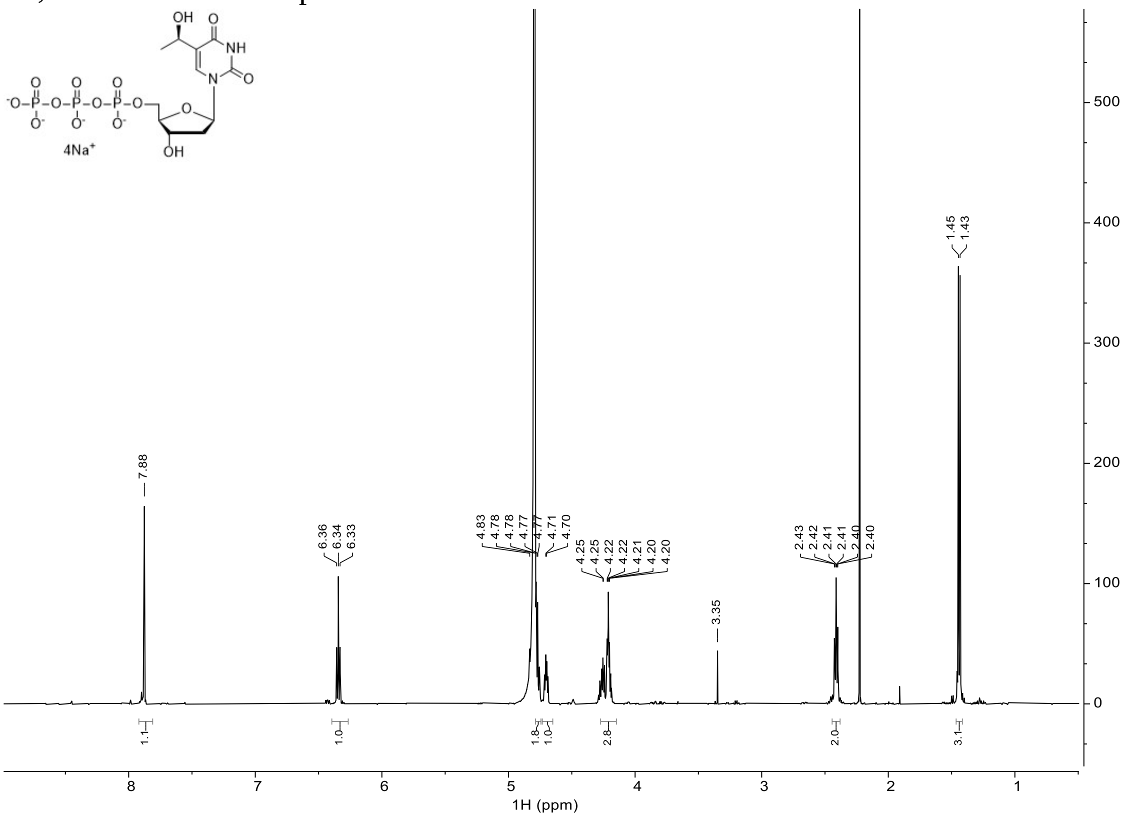

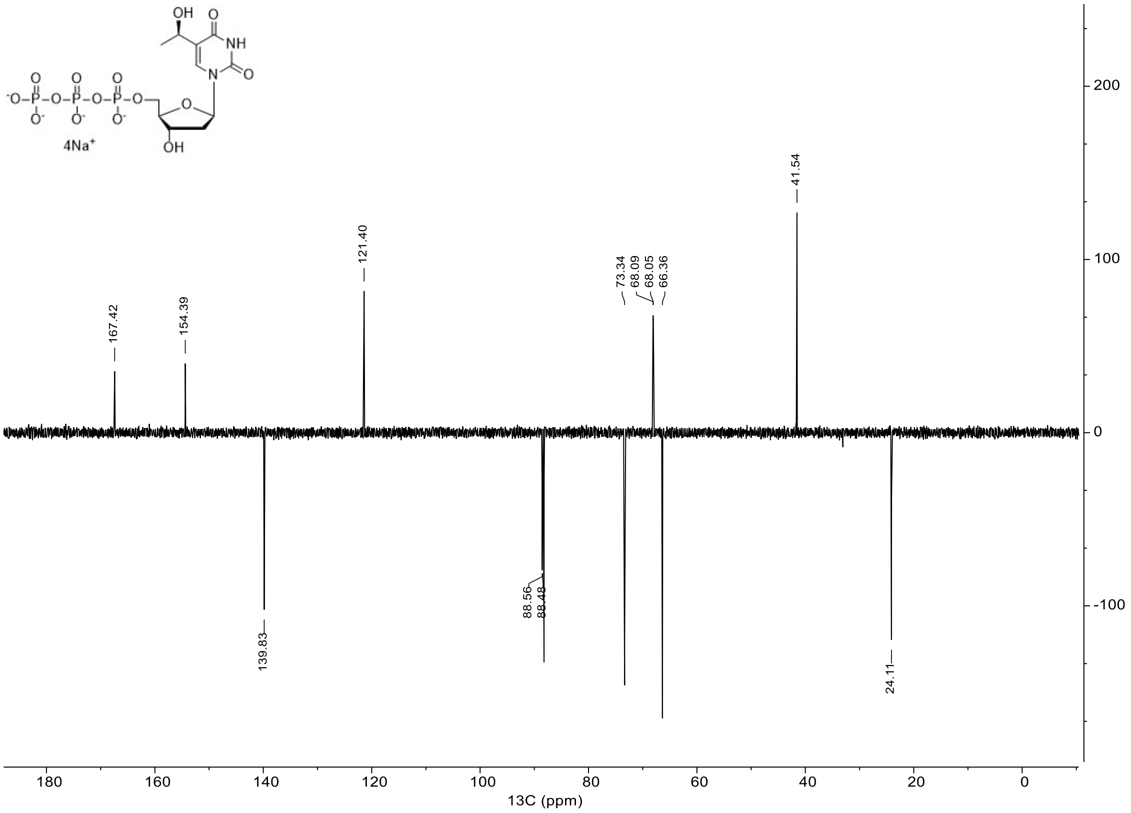

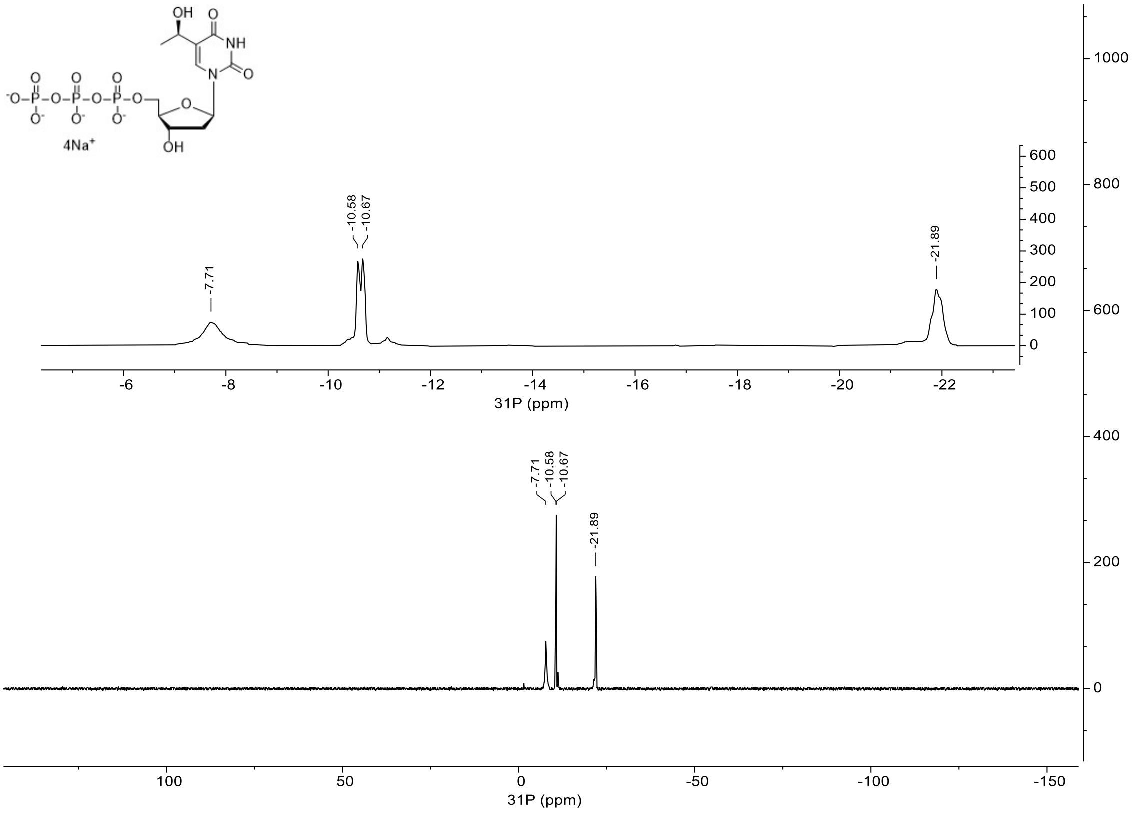

^{1}H, ^{13}C and ^{31}P NMR spectra of **dU^{She}TP**

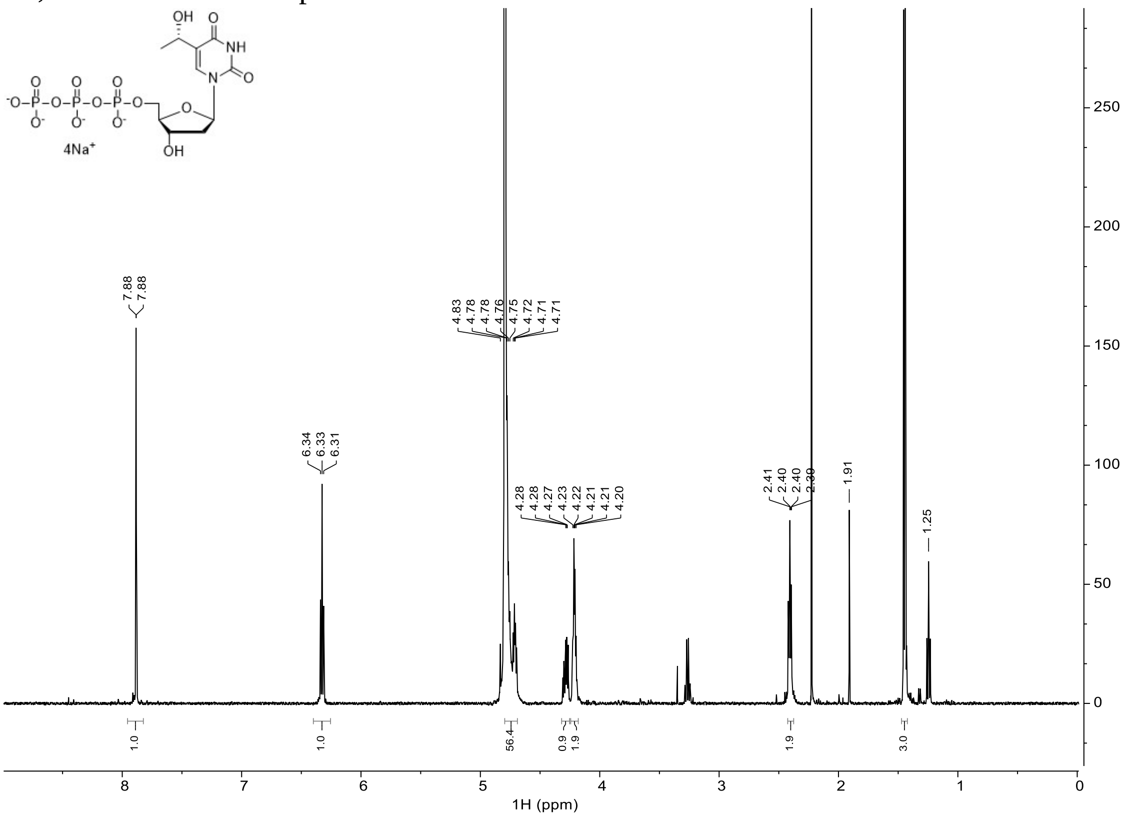

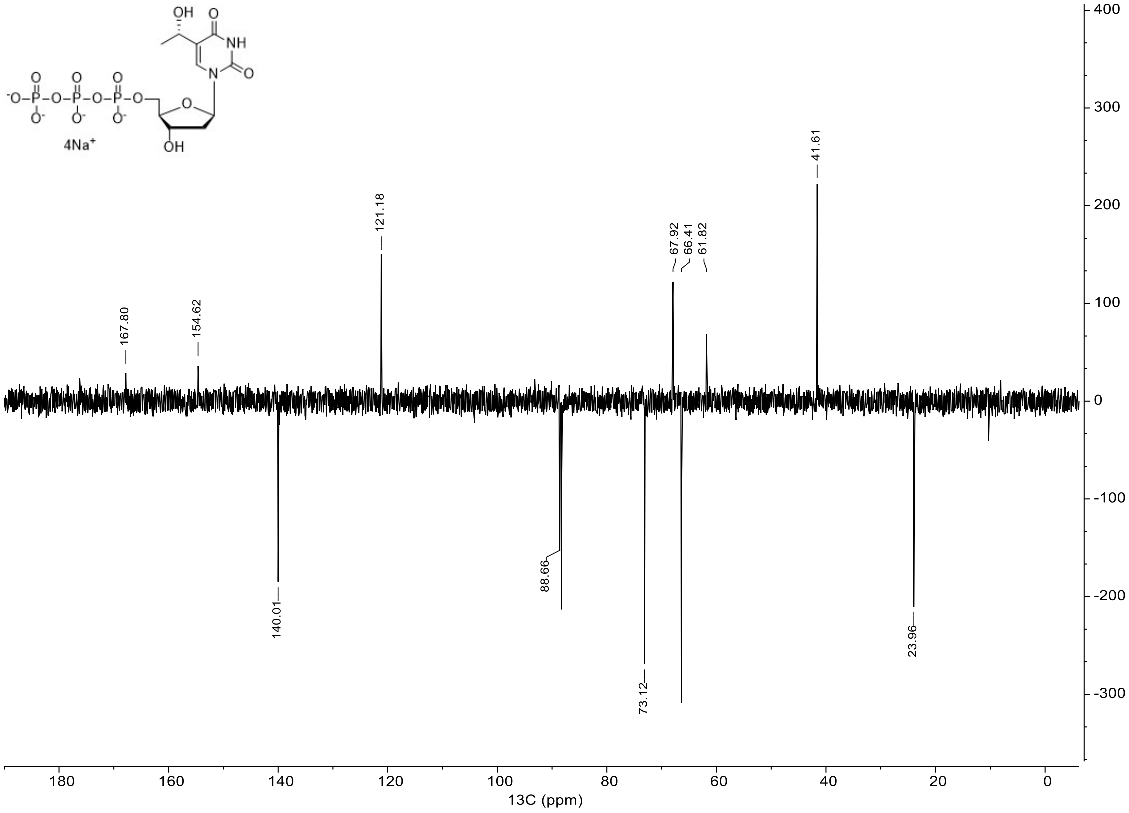
OH
O
NH
N
O
-O-P-O-P-O-P-O
O-
OH
4Na+
167.80
154.62
121.18
140.01
88.66
73.12
67.92
66.41
61.82
41.61
23.96
13C (ppm)

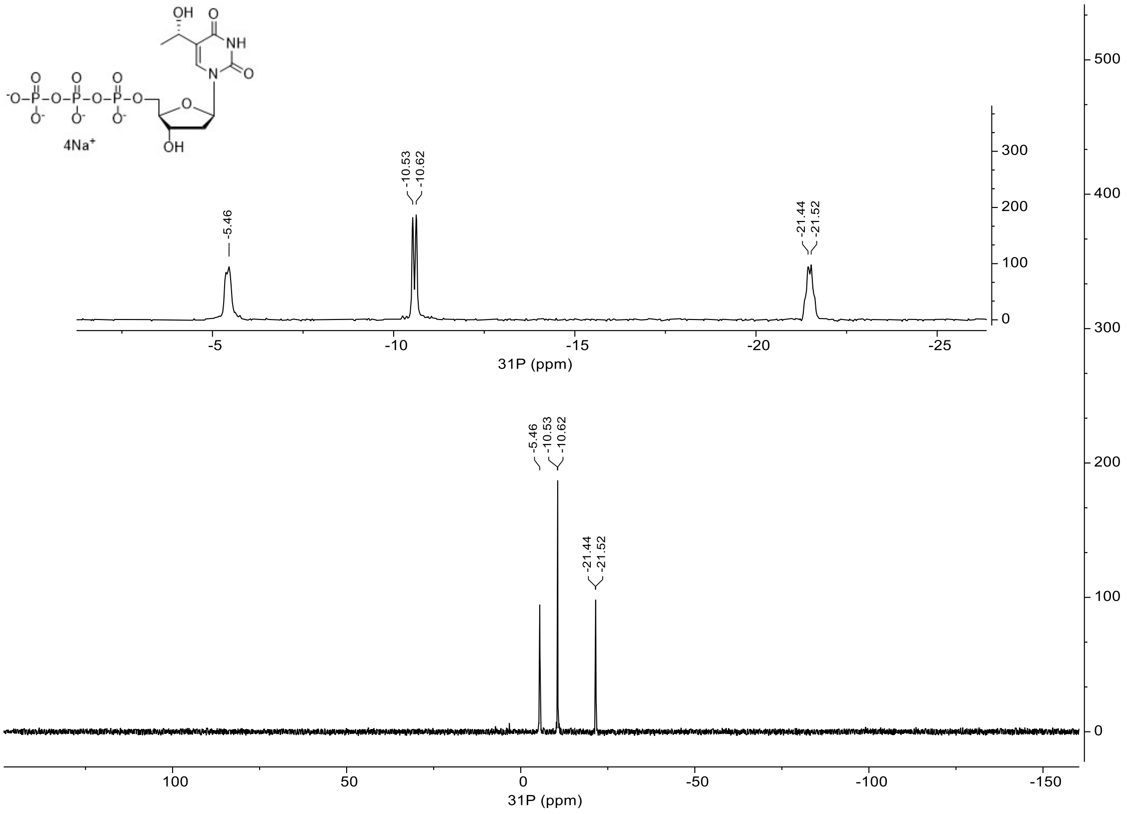
OH
O
NH
N
O
-O-P-O-P-O-P-O
O-
OH
4Na+
-5.46
-10.53
-10.62
-21.44
-21.52
31P (ppm)
31P (ppm)

^{1}H, ^{13}C and ^{31}P NMR spectra of **dC^{Rhe}TP**

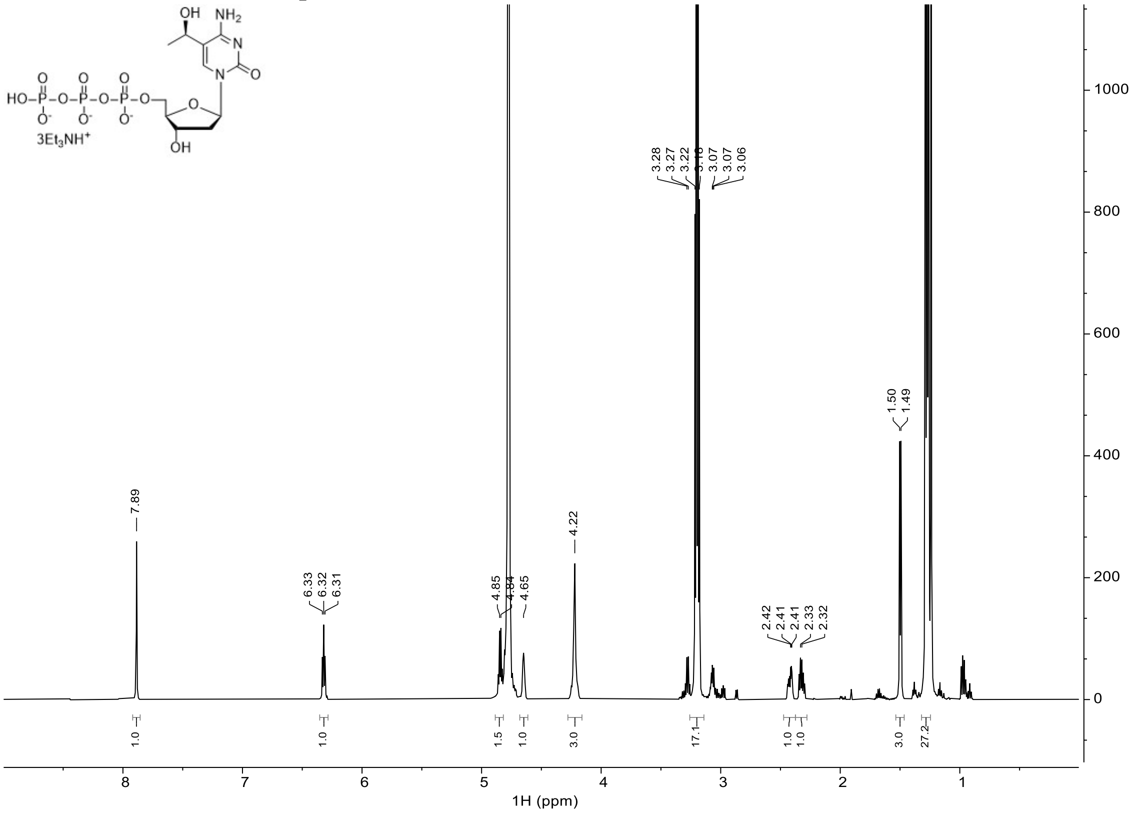

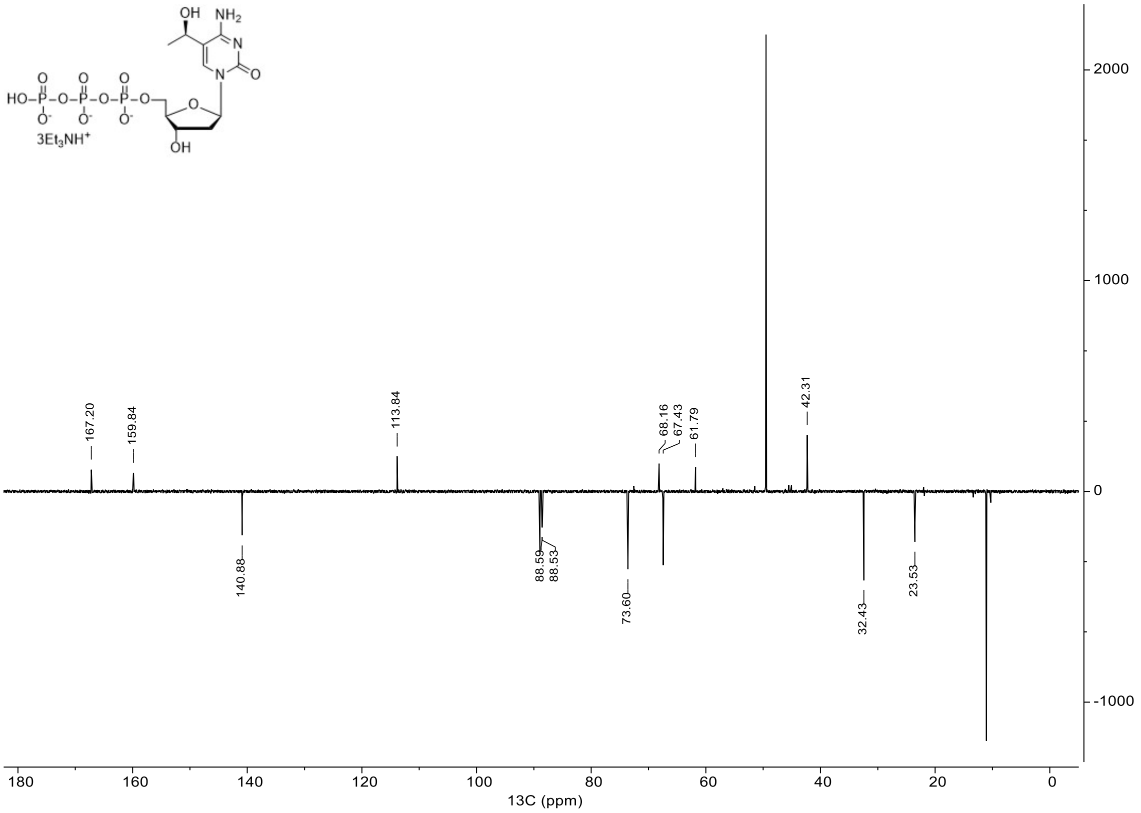

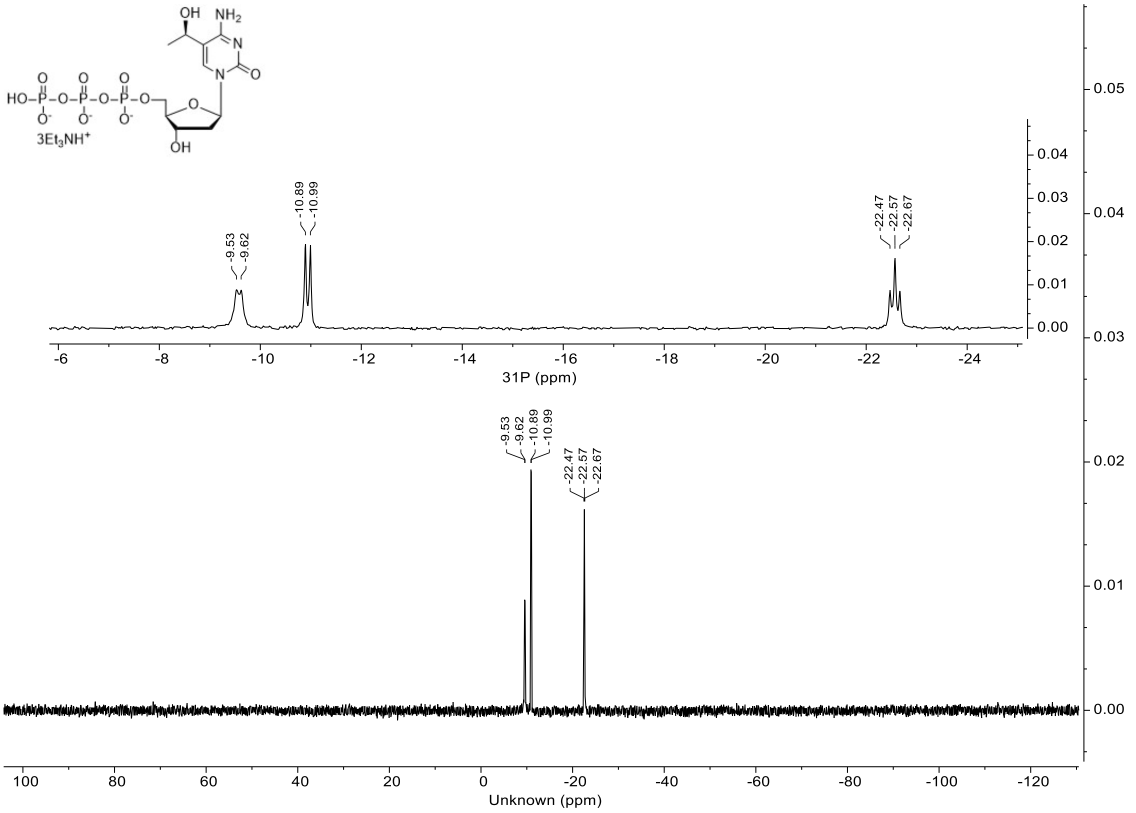

^{1}H, ^{13}C and ^{31}P NMR spectra of **dC$^{\mathbf{She}}$TP**

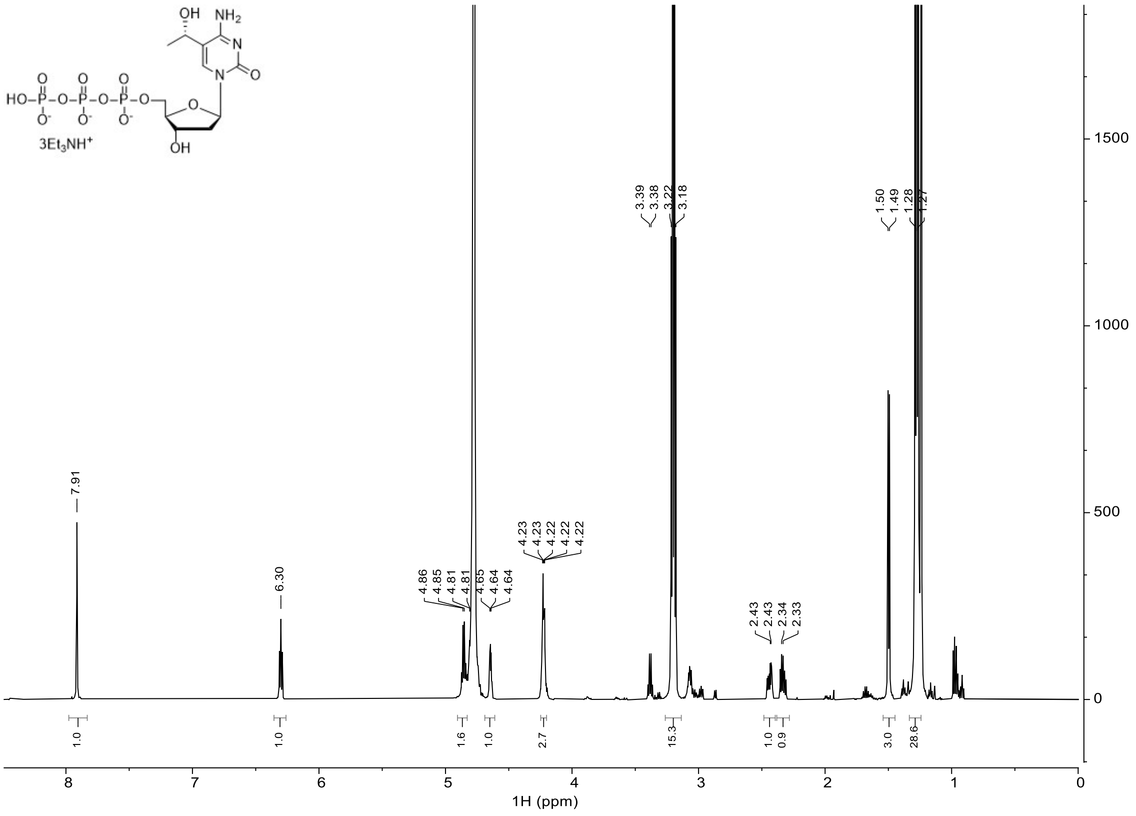

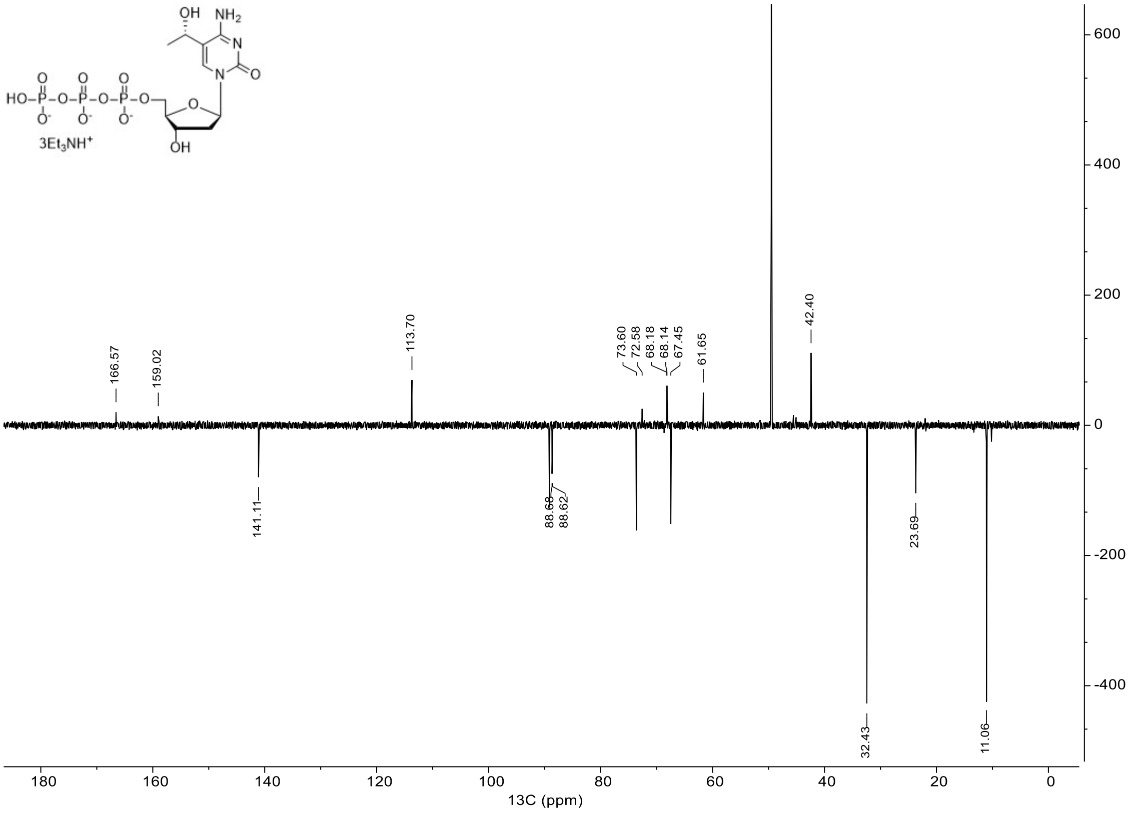
OH
NH2
N
N
O
HO-P-O-P-O-P-O
O
O-
3Et3NH+
OH
166.57
159.02
113.70
73.60
72.58
68.18
68.14
67.45
61.65
42.40
141.11
88.68
88.62
23.69
32.43
11.06
600
400
200
0
-200
-400
180
160
140
120
100
80
60
40
20
0
13C (ppm)

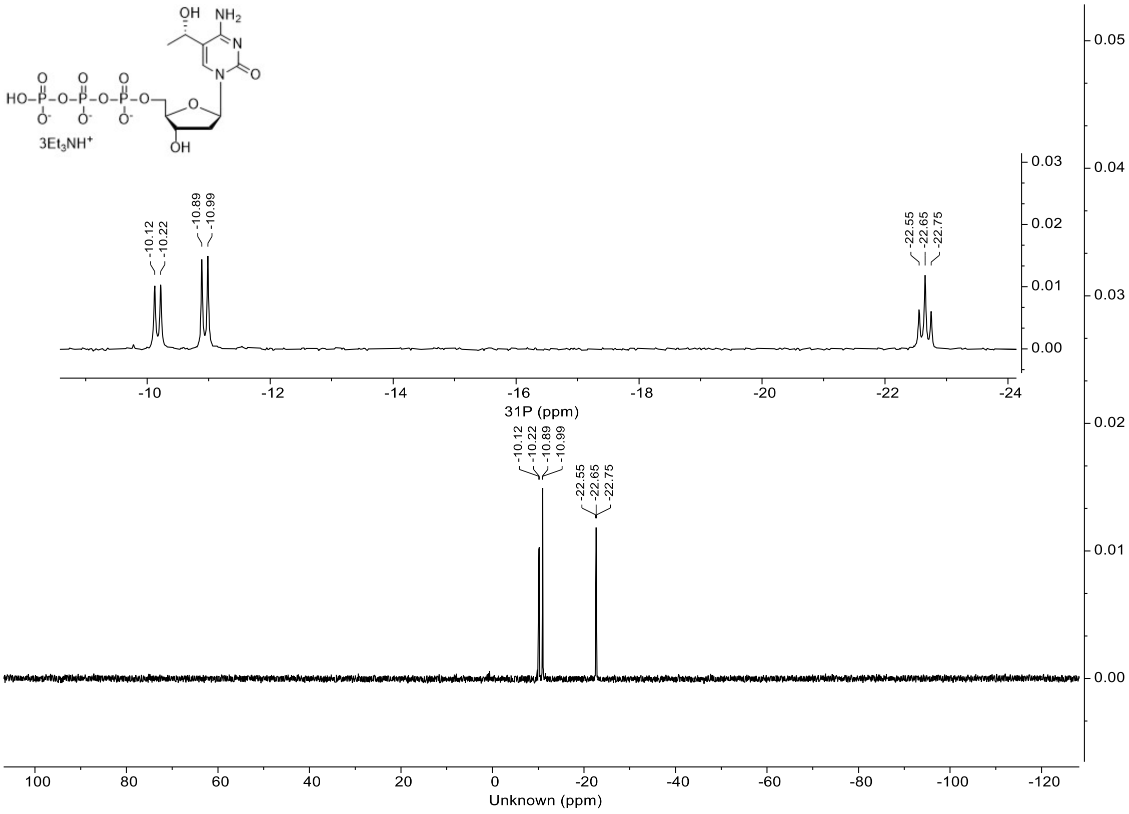
OH
NH2
N
N
O
HO-P-O-P-O-P-O
O
O-
3Et3NH+
OH
-10.12
-10.22
-10.89
-10.99
-22.55
-22.65
-22.75
0.03
0.02
0.01
0.00
-10
-12
-14
-16
-18
-20
-22
-24
31P (ppm)
0.05
0.04
0.03
0.02
0.01
0.00
100
80
60
40
20
0
-20
-40
-60
-80
-100
-120
Unknown (ppm)

^{1}H, ^{13}C and ^{31}P NMR spectra of **dU^{pr}TP**

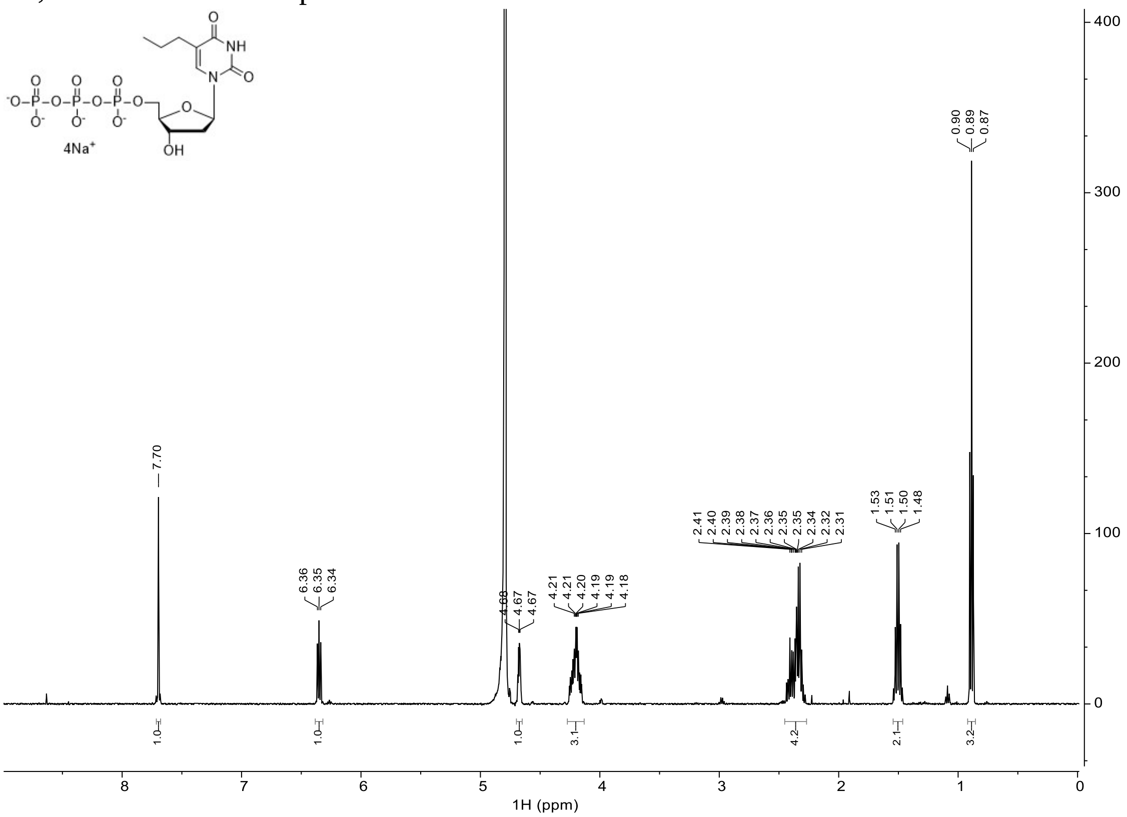

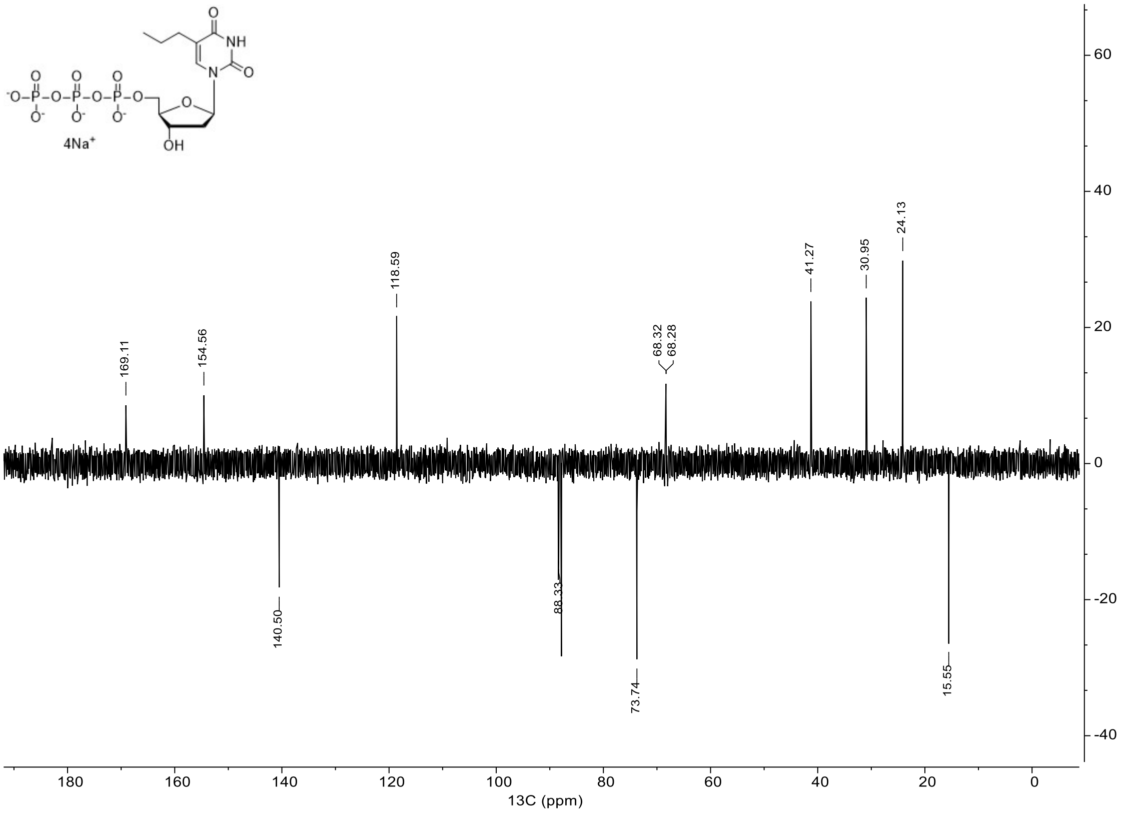

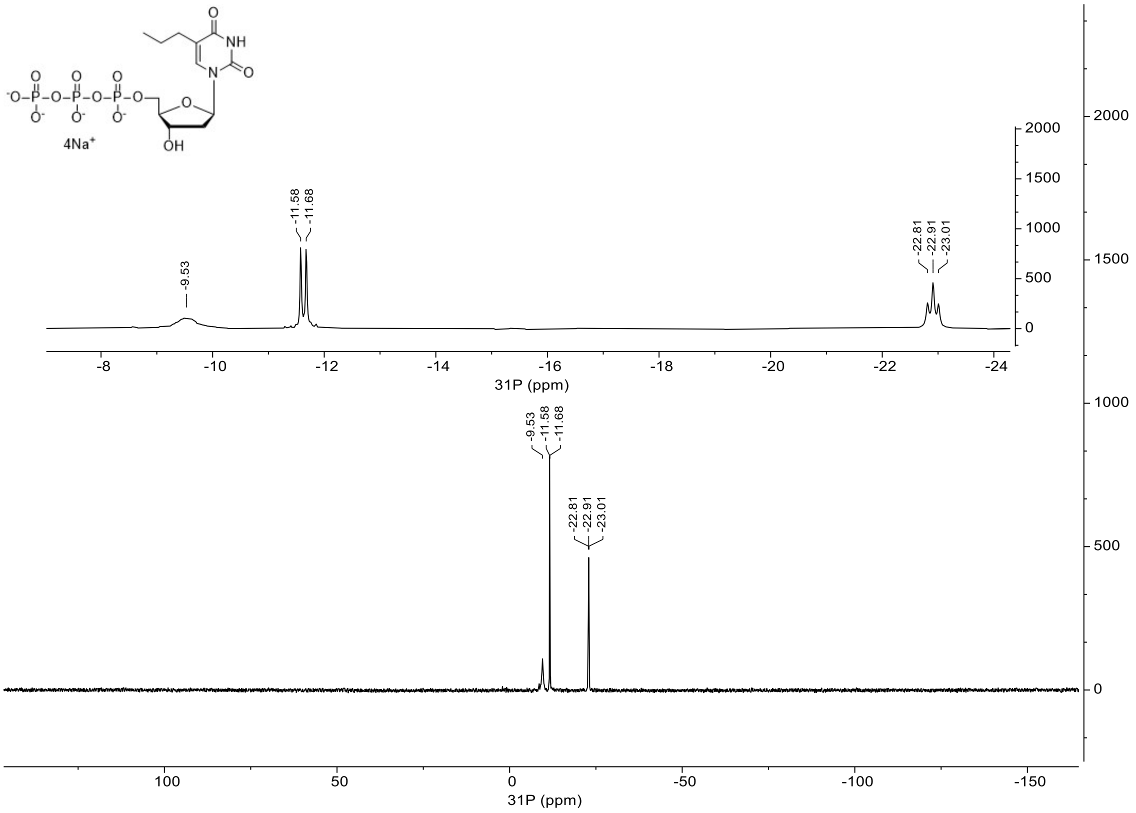

^{1}H, ^{13}C and ^{31}P NMR spectra of **dU^{pp}TP**

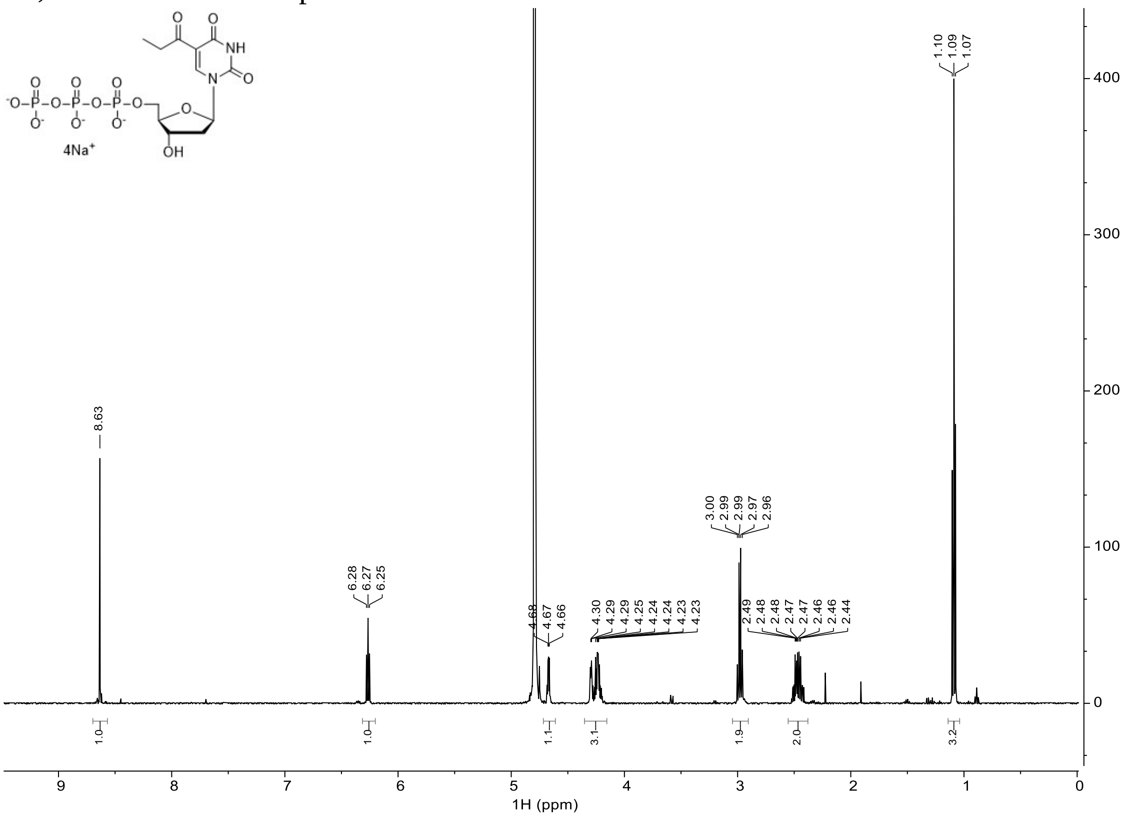

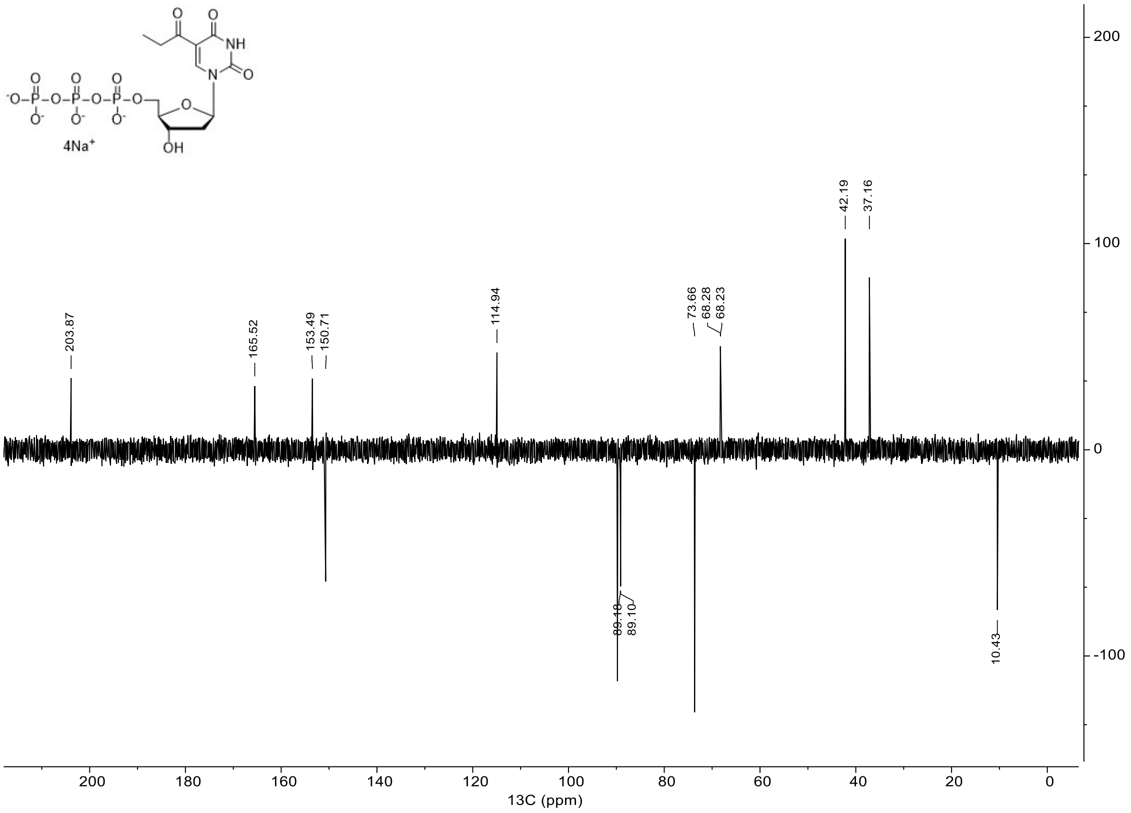
4Na+
OH
NH
203.87
165.52
153.49
150.71
114.94
73.66
68.28
68.23
42.19
37.16
89.18
89.10
10.43
13C (ppm)

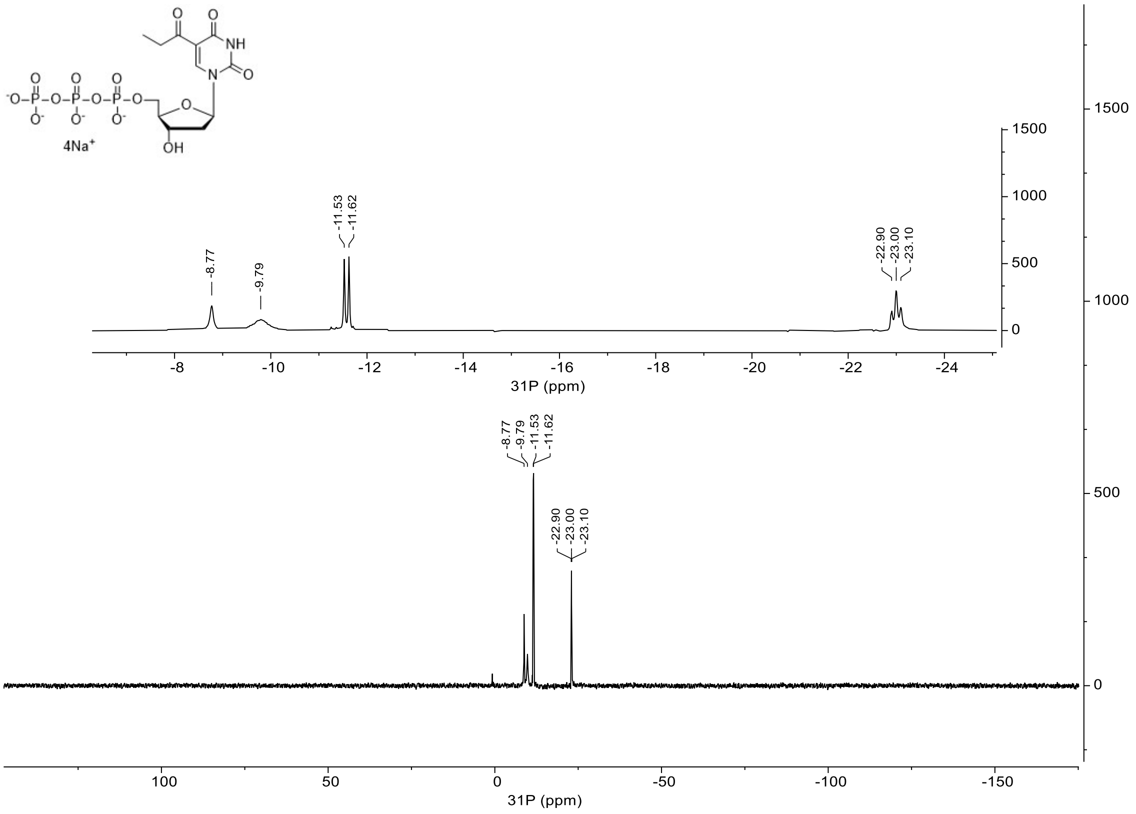
4Na+
OH
NH
-8.77
-9.79
-11.53
-11.62
-22.90
-23.00
-23.10
31P (ppm)

^{1}H, ^{13}C and ^{31}P NMR spectra of **dC^{pr}TP**

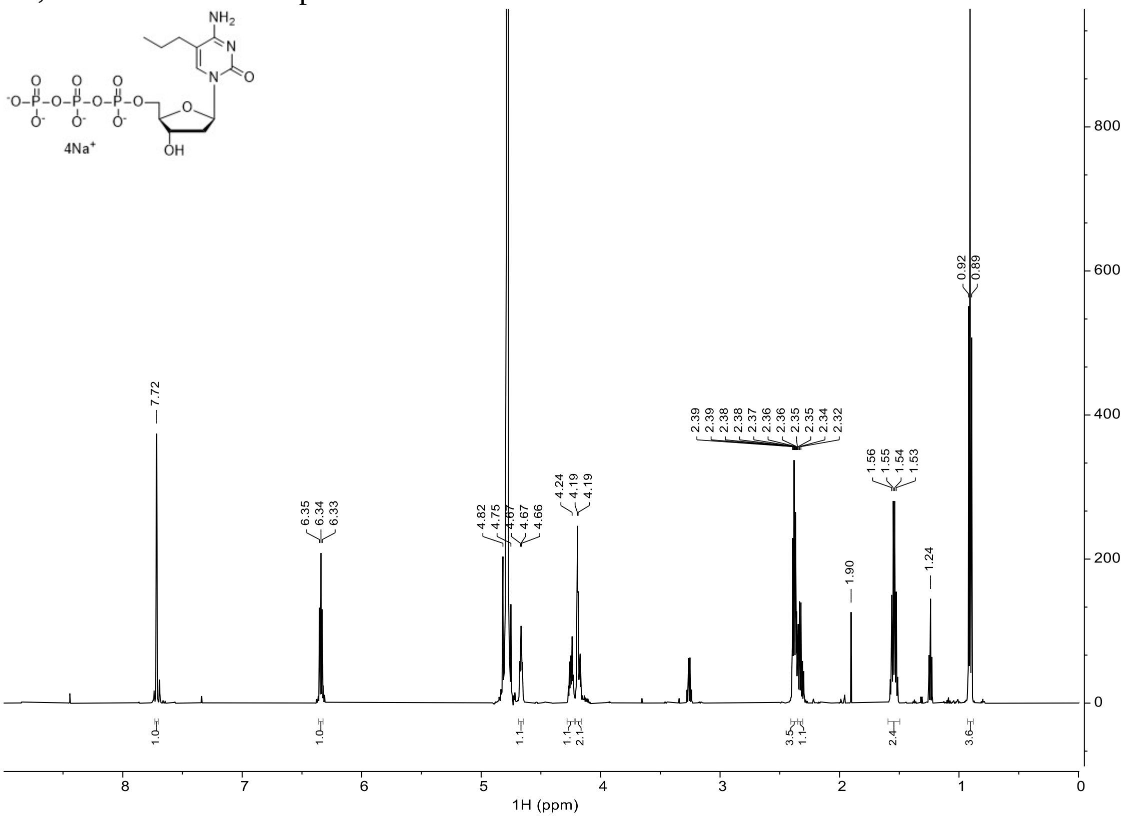

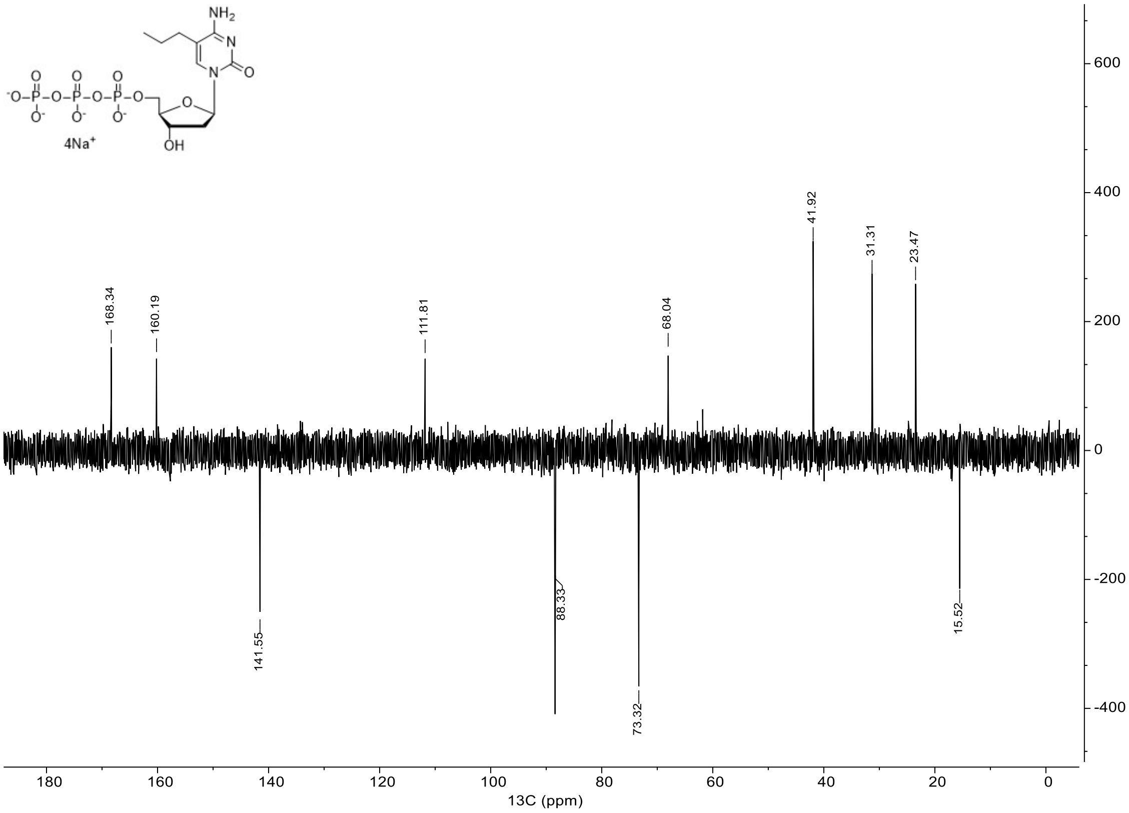

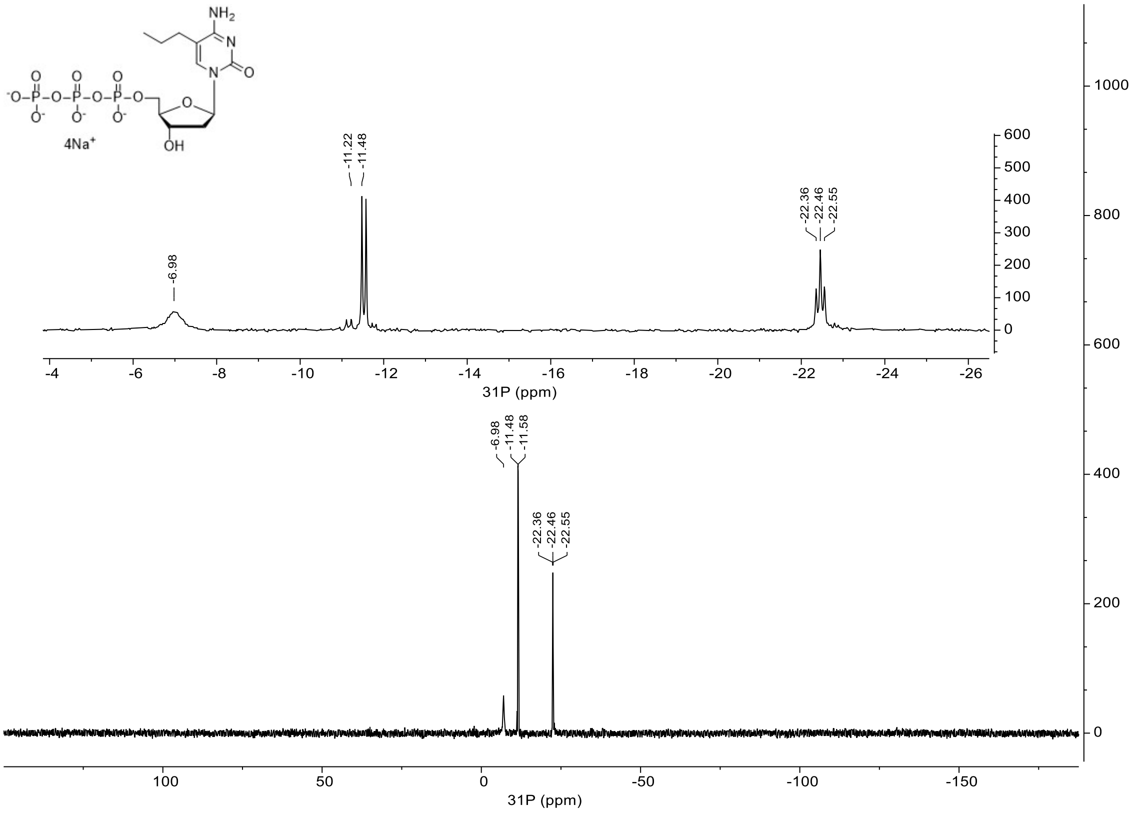

^{1}H, ^{13}C and ^{31}P NMR spectra of **dC^ppTP**

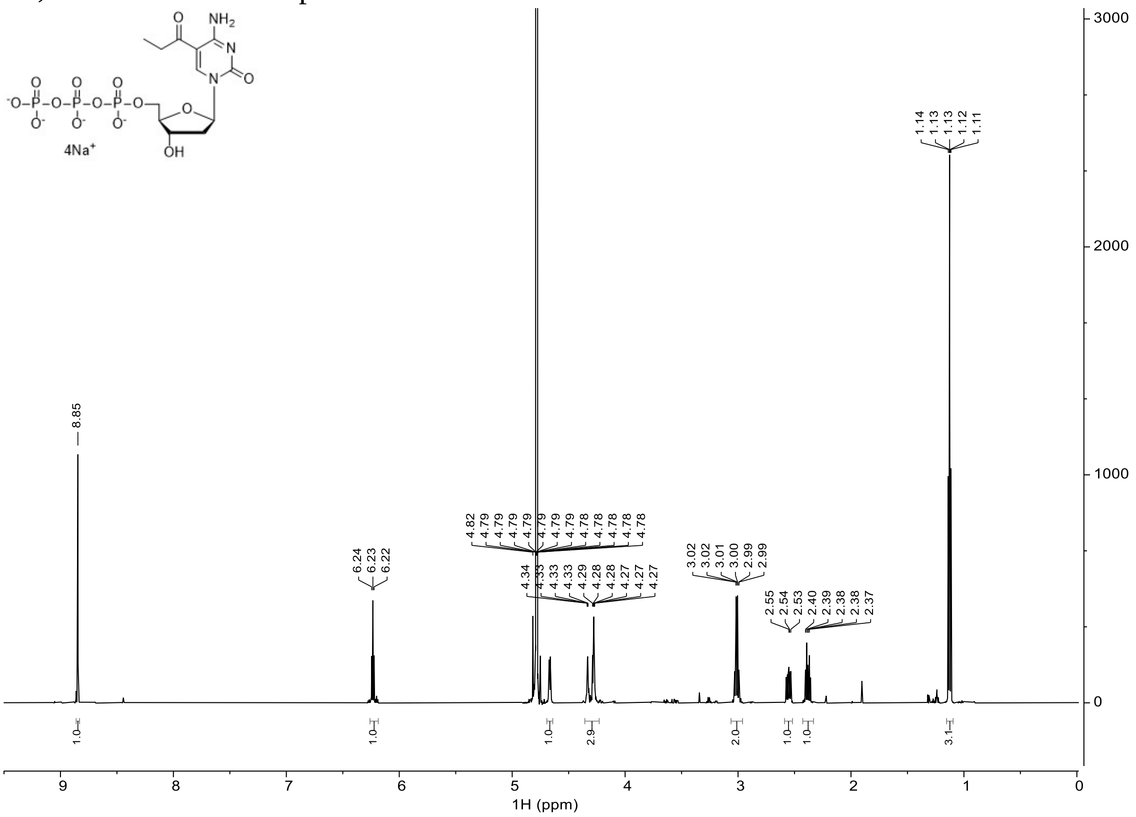

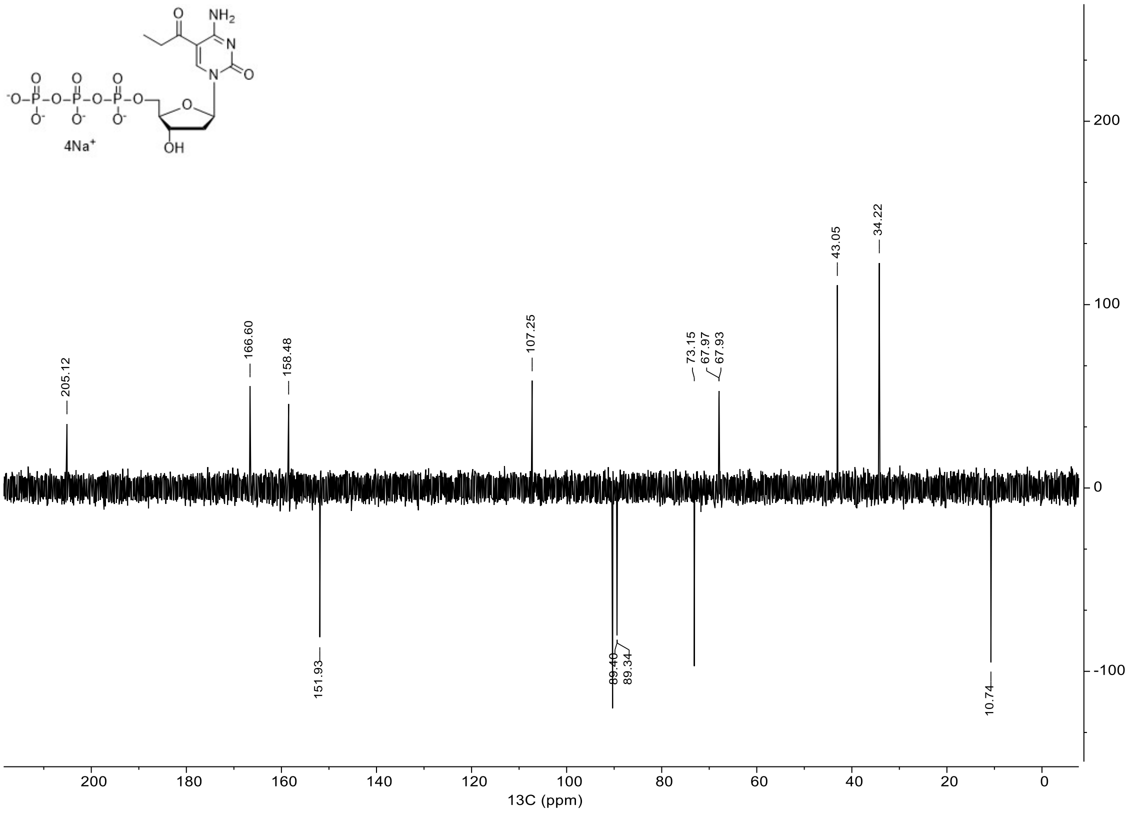
NH2
4Na+
OH
205.12
166.60
158.48
151.93
107.25
89.40
89.34
73.15
67.97
67.93
43.05
34.22
10.74
13C (ppm)

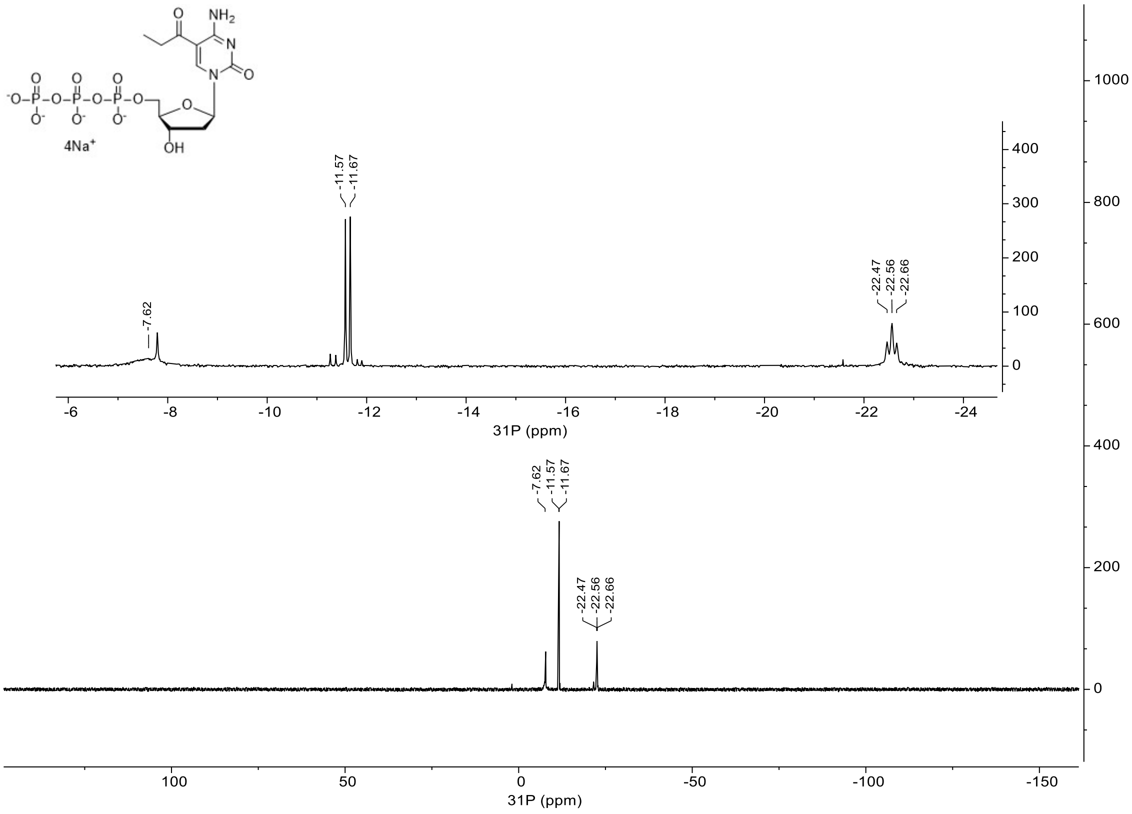
NH2
4Na+
OH
-7.62
-11.57
-11.67
-22.47
-22.56
-22.66
31P (ppm)

^{1}H, ^{13}C and ^{31}P NMR spectra of **dU^{Rhp}TP**

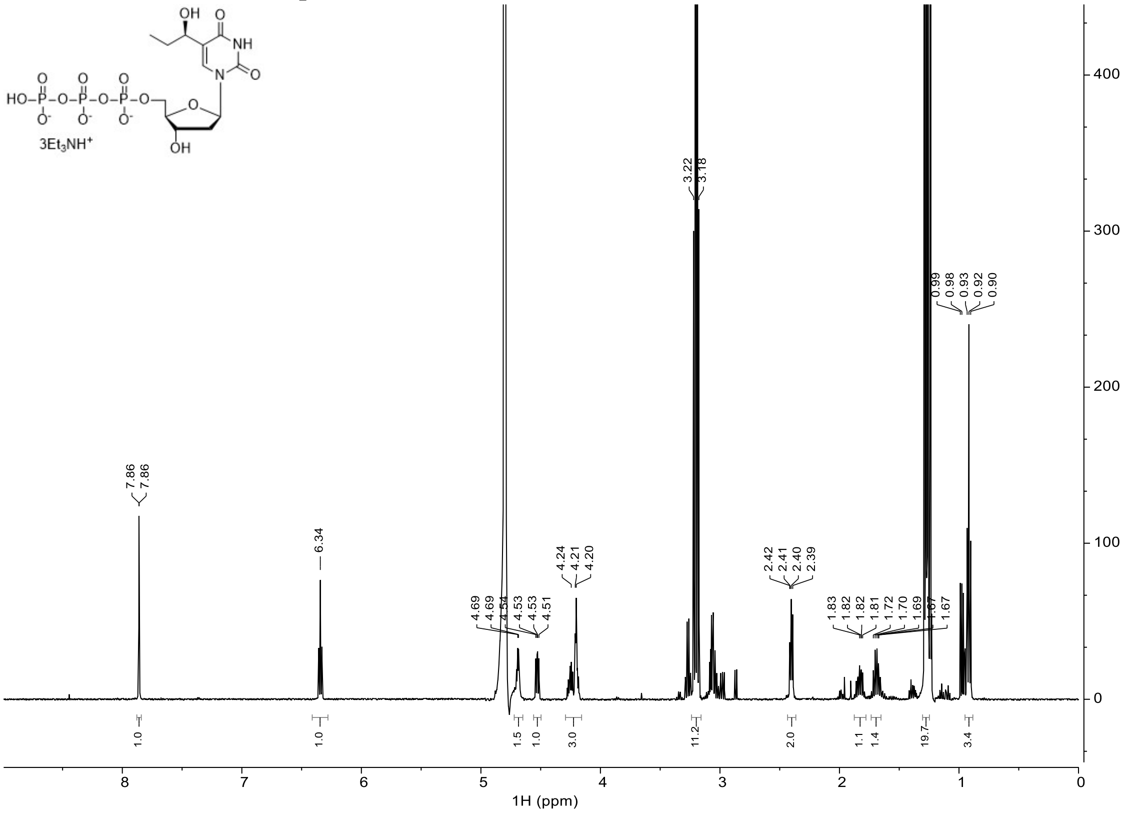

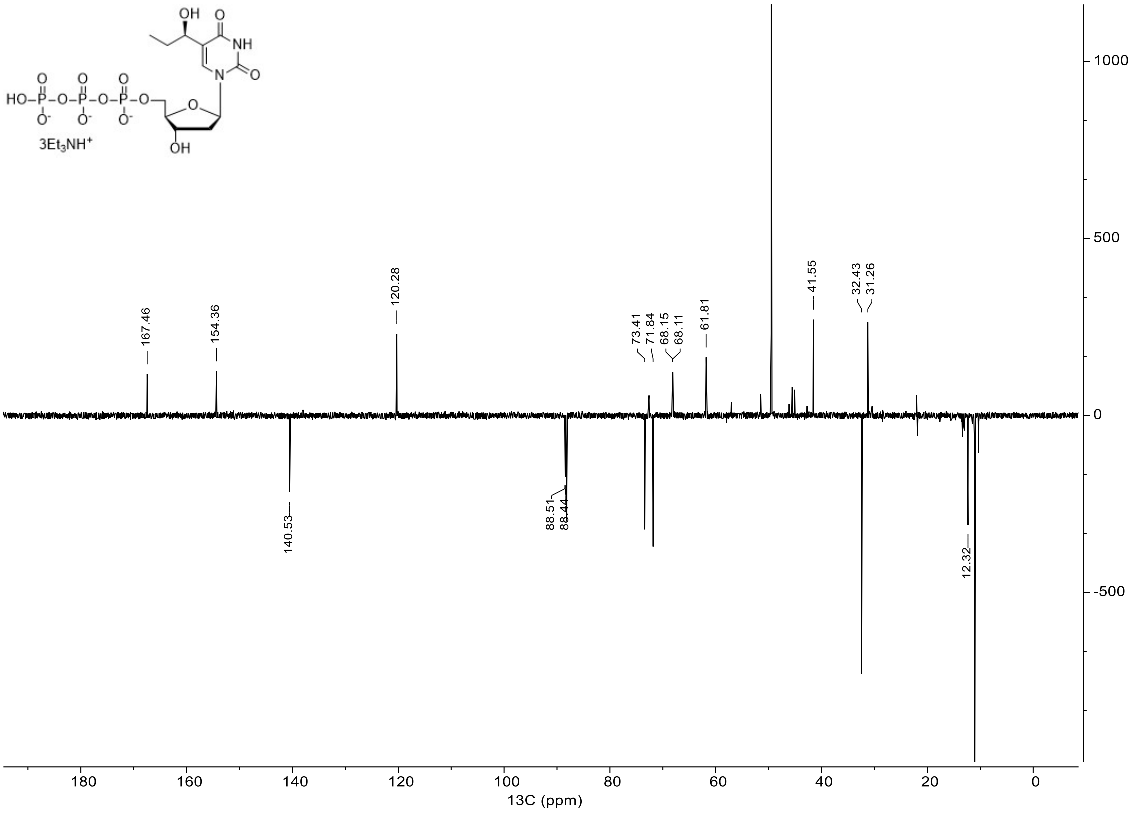

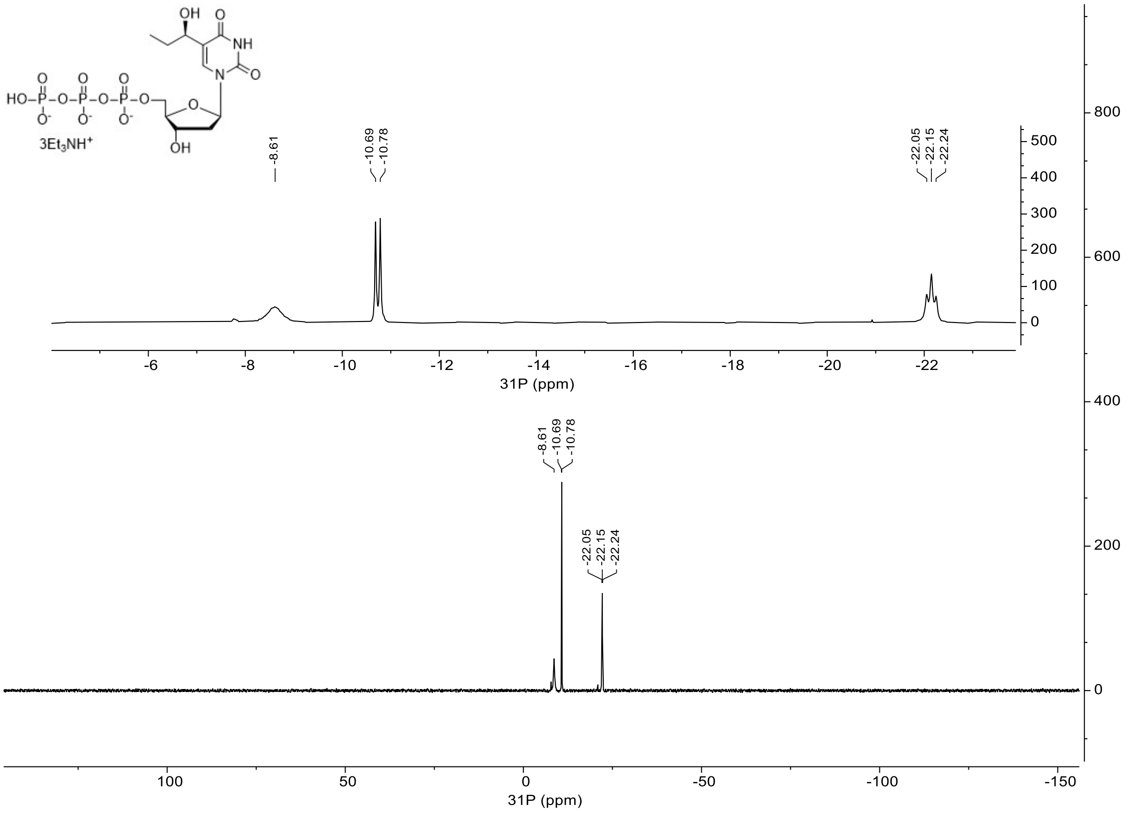

^{1}H, ^{13}C and ^{31}P NMR spectra of **dU^{Shp}TP**

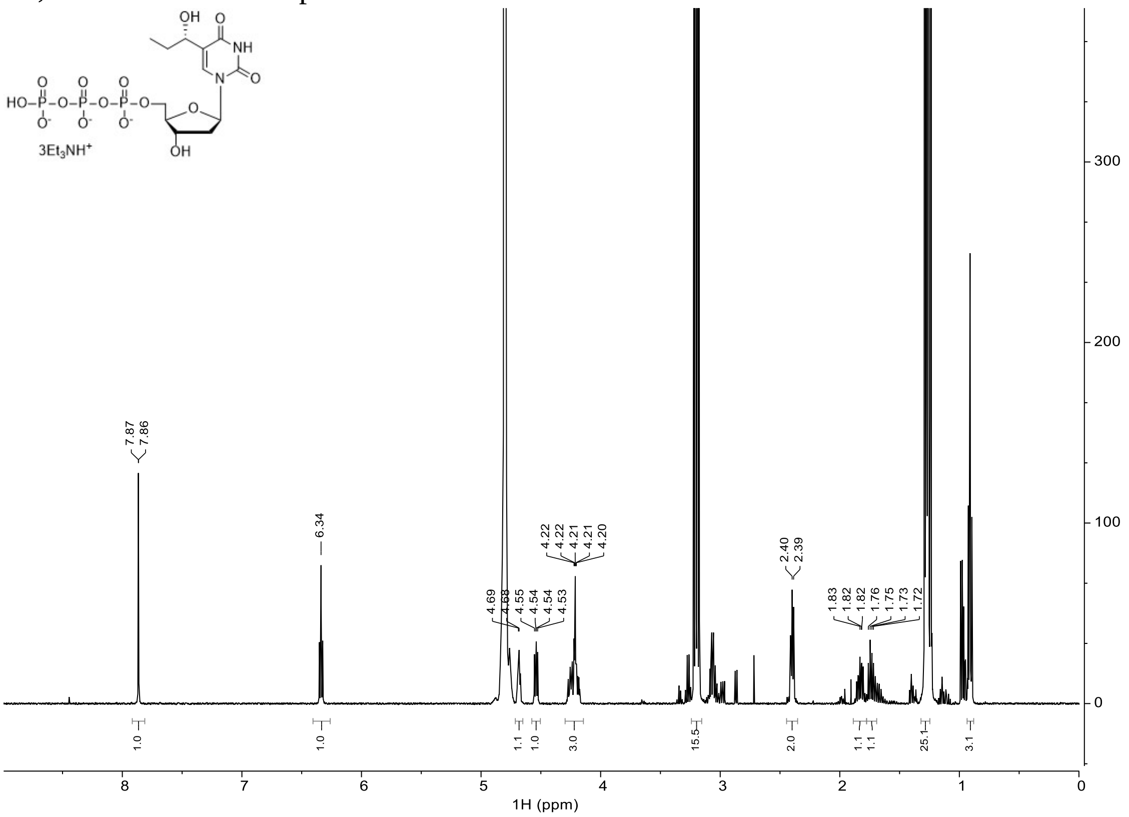

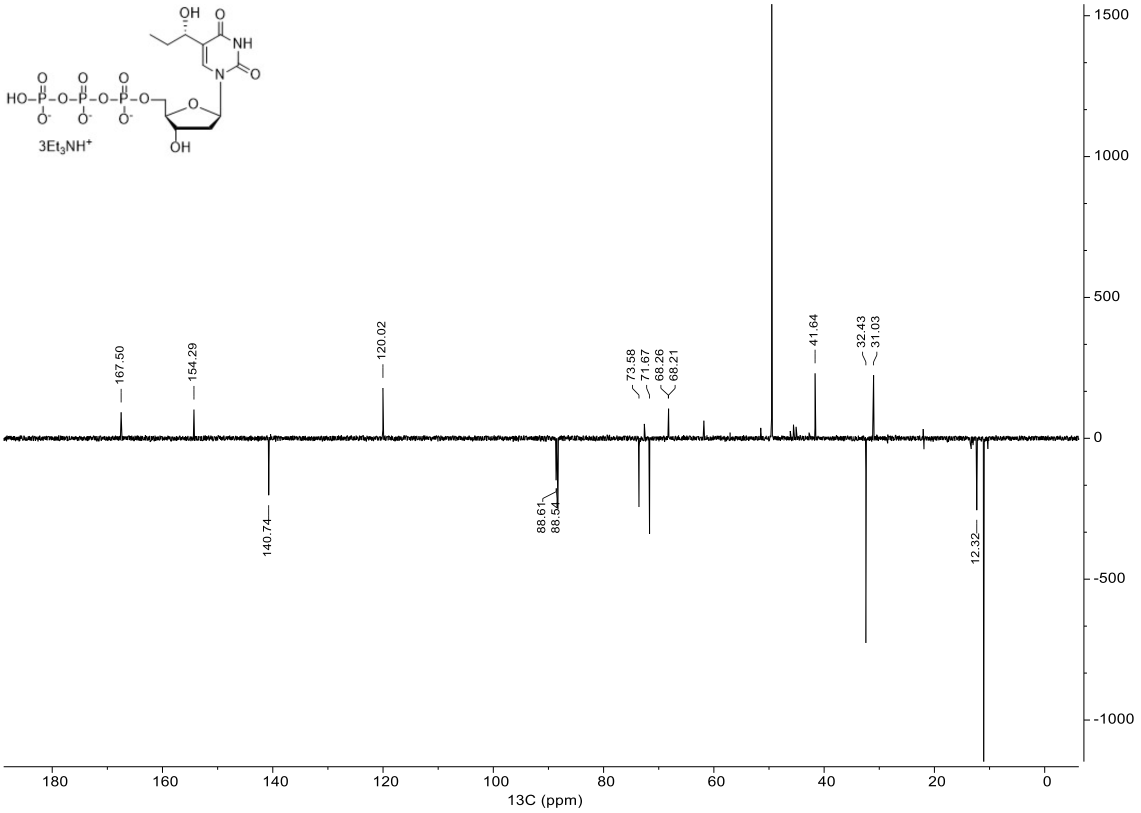
OH
O
NH
N
O
HO-P-O-P-O-P-O
O
OH
3Et$_3$NH$^+$
167.50
154.29
120.02
140.74
88.61
88.54
73.58
71.67
68.26
68.21
41.64
32.43
31.03
12.32
1500
1000
500
0
-500
-1000
180
160
140
120
100
80
60
40
20
0
13C (ppm)

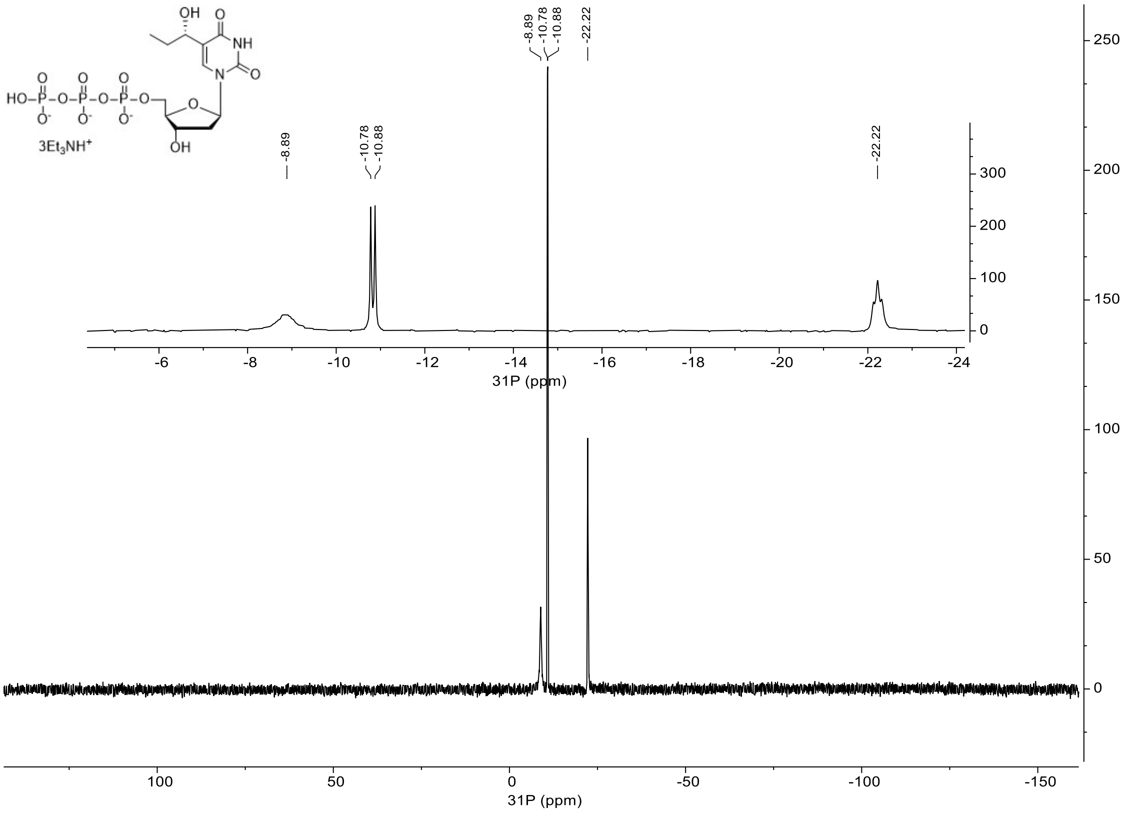
OH
O
NH
N
O
HO-P-O-P-O-P-O
O
OH
3Et$_3$NH$^+$
-8.89
-10.78
-10.88
-22.22
300
200
100
0
-6
-8
-10
-12
-14
-16
-18
-20
-22
-24
31P (ppm)
250
200
150
100
50
0
100
50
0
-50
-100
-150
31P (ppm)

^{1}H, ^{13}C and ^{31}P NMR spectra of **$dC^{Rhp}TP$**

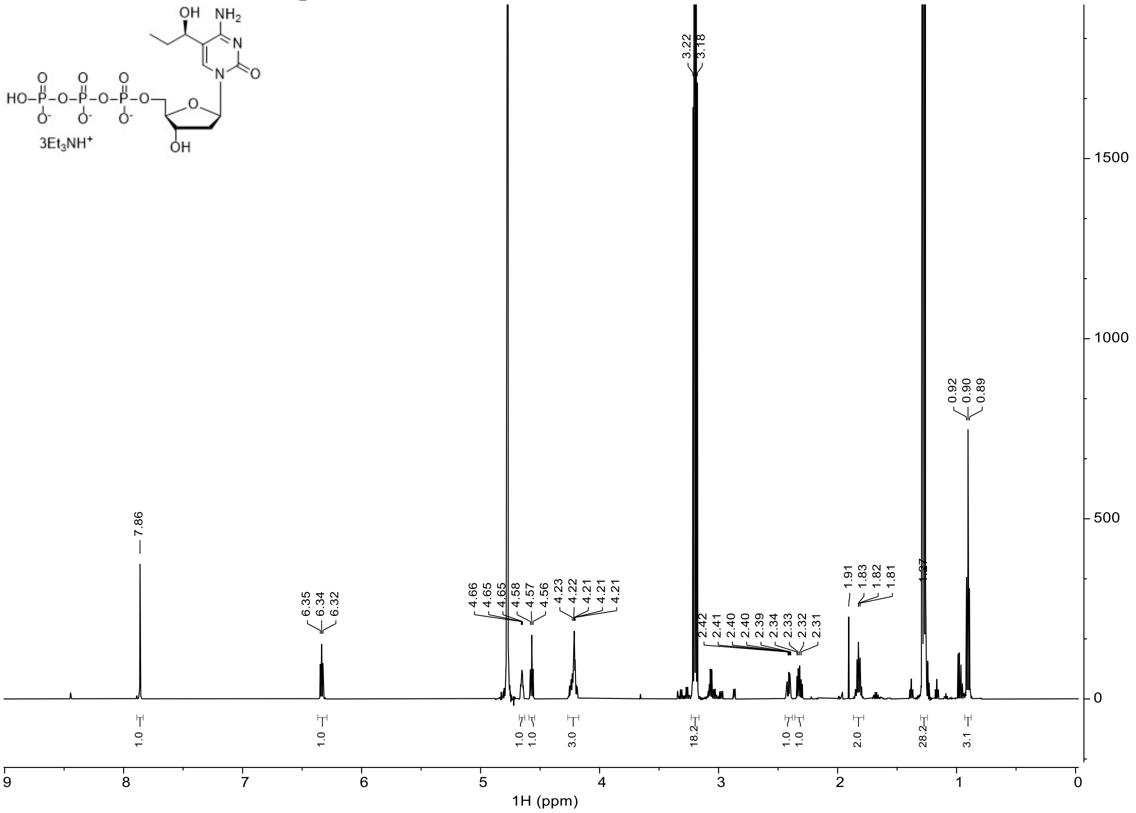

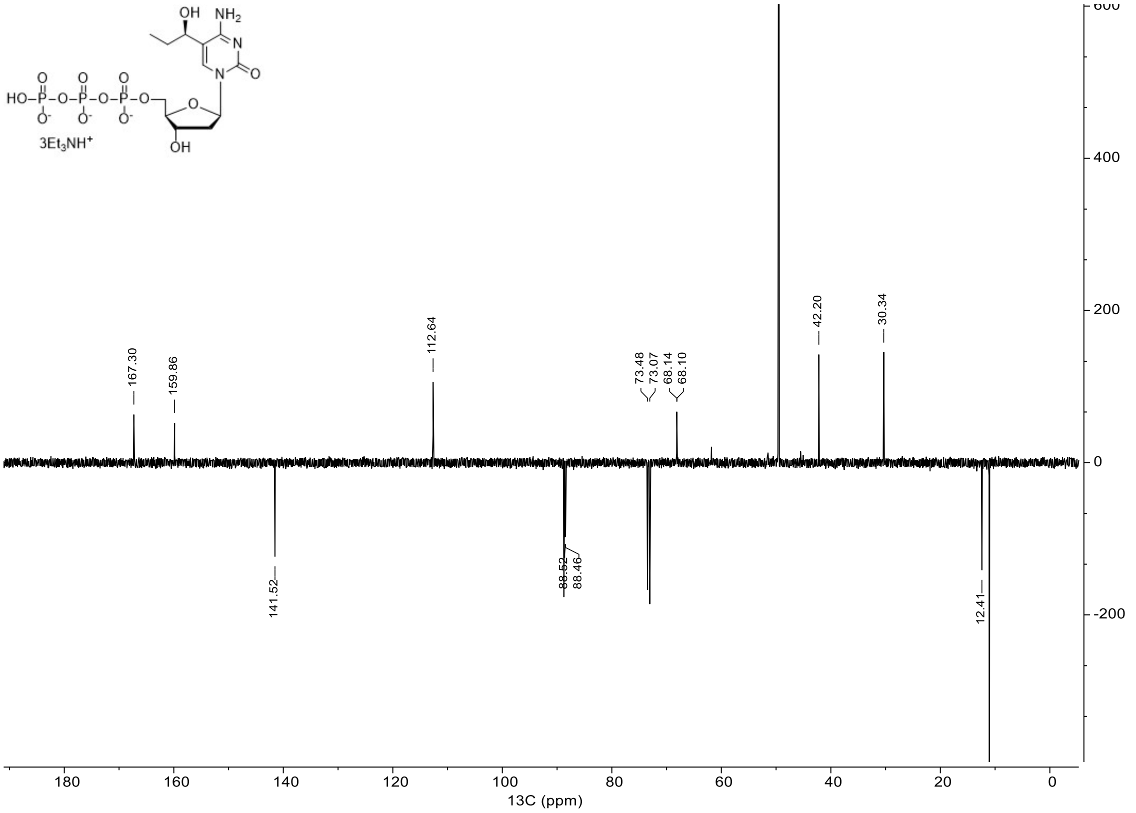

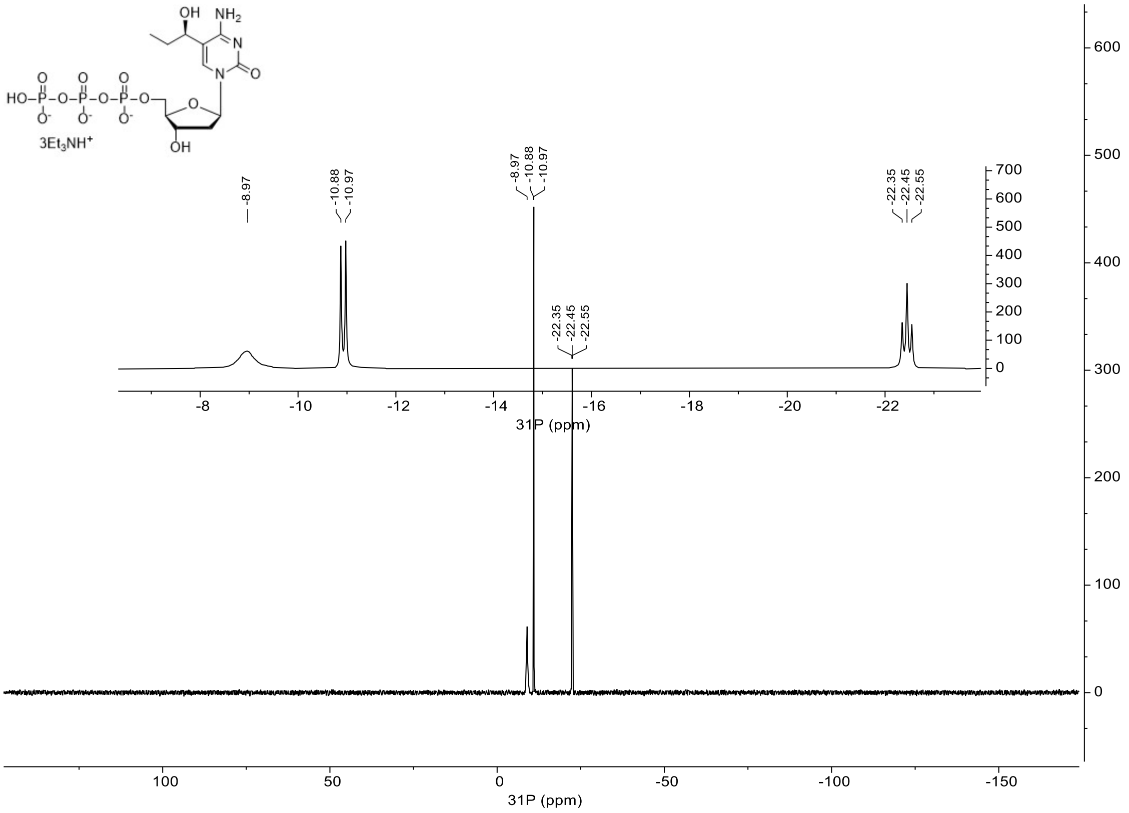

^{1}H, ^{13}C and ^{31}P NMR spectra of **dC^{Shp}TP**

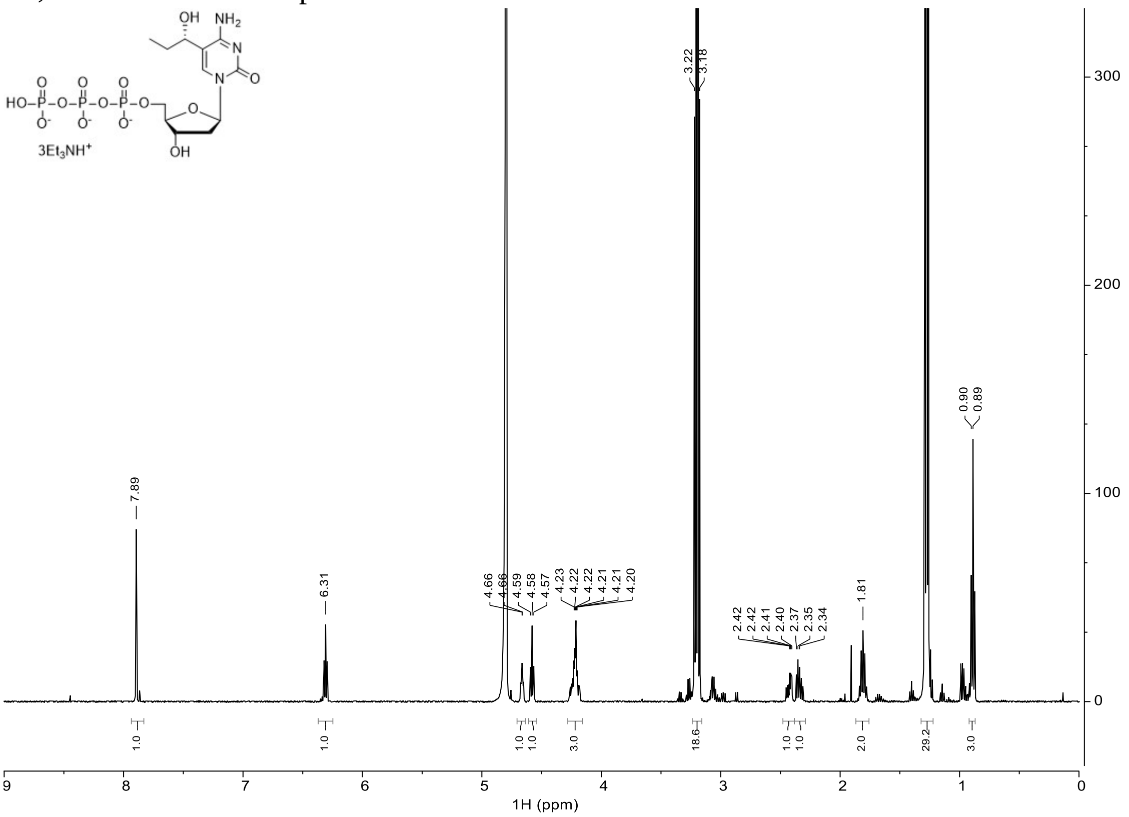

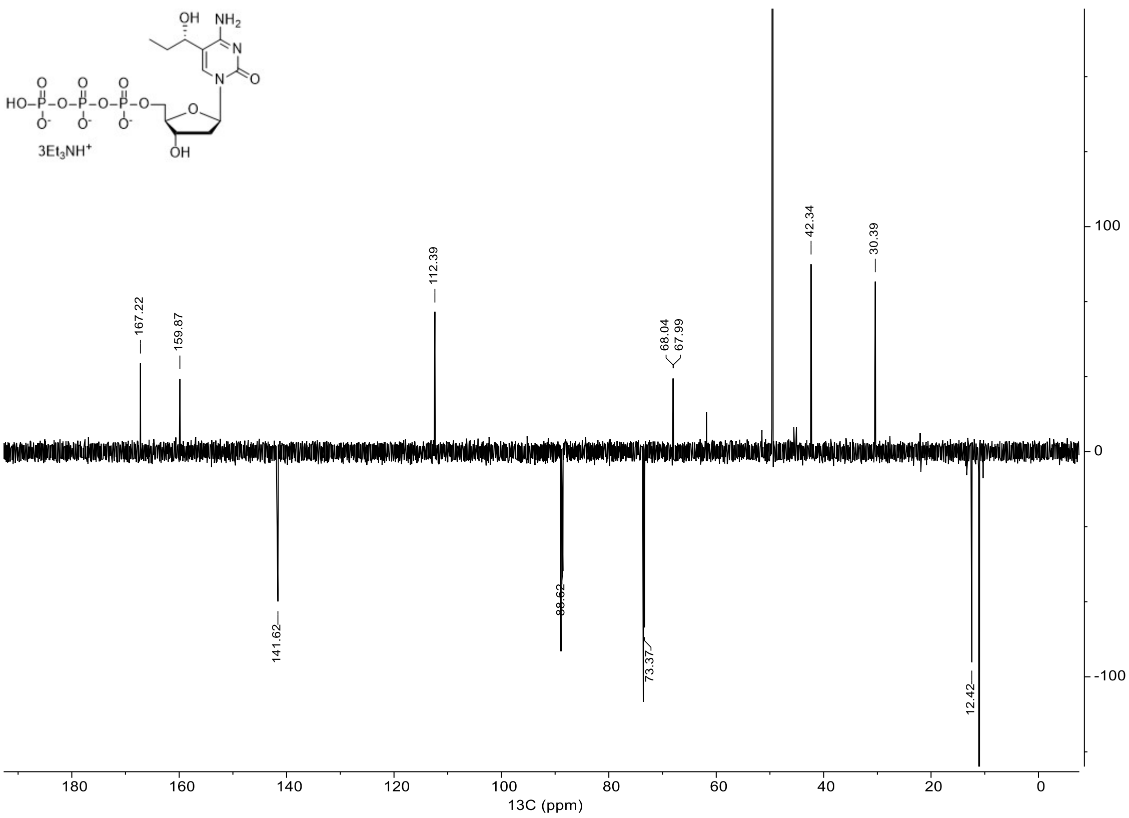
3Et3NH+
167.22
159.87
141.62
112.39
88.62
73.37
68.04
67.99
42.34
30.39
12.42
13C (ppm)

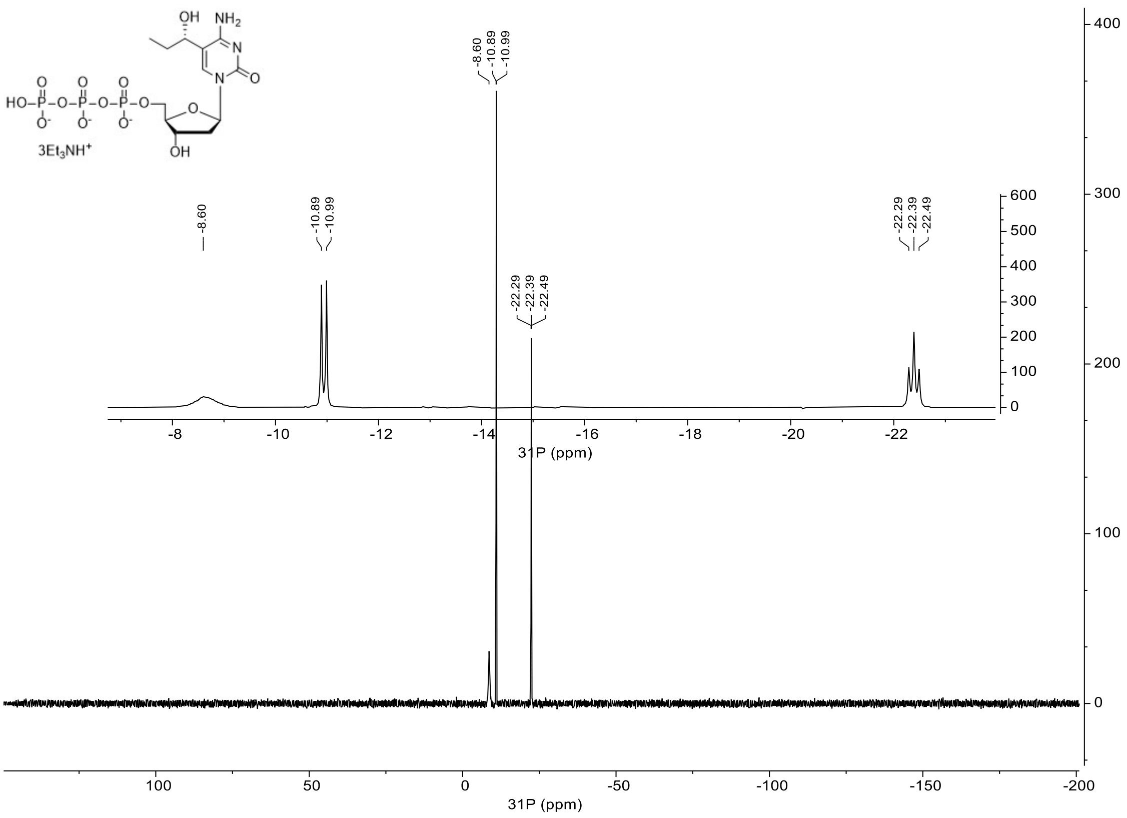
3Et3NH+
-8.60
-10.89
-10.99
-22.29
-22.39
-22.49
31P (ppm)

^{1}H, ^{13}C and ^{31}P NMR spectra of **dU^{f}TP**

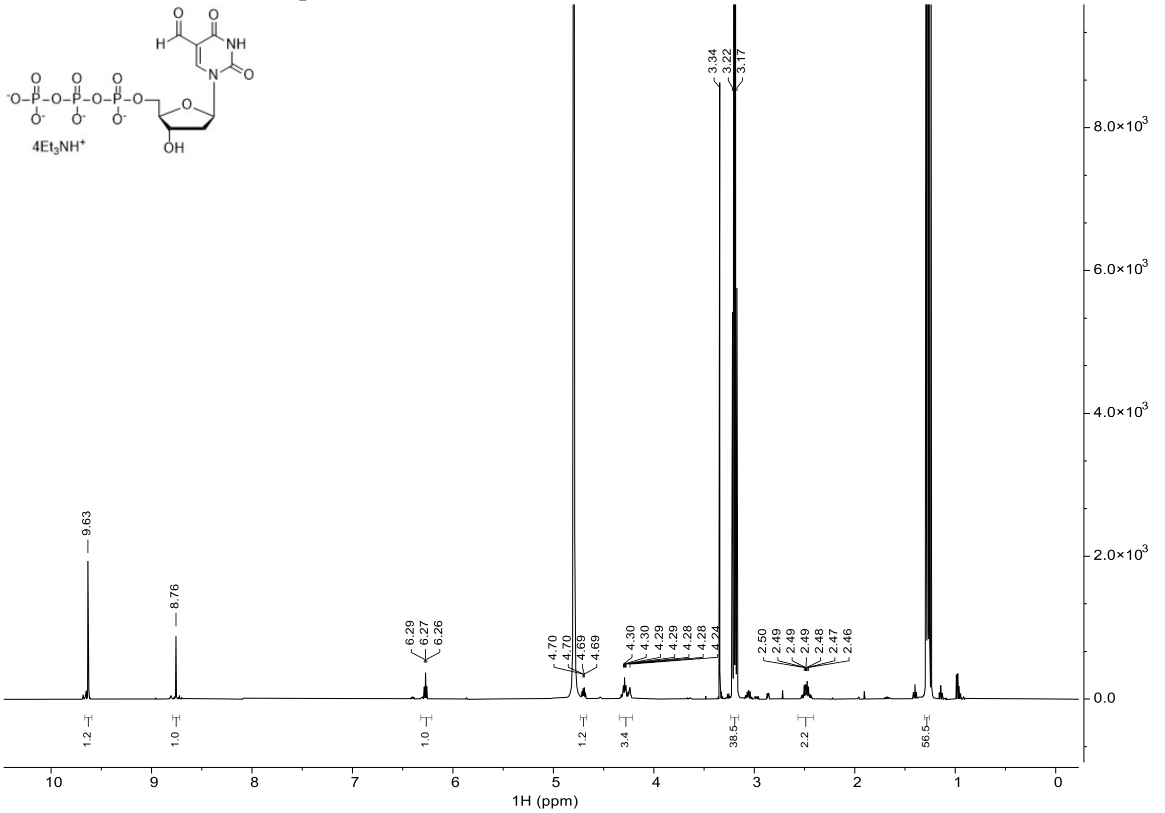

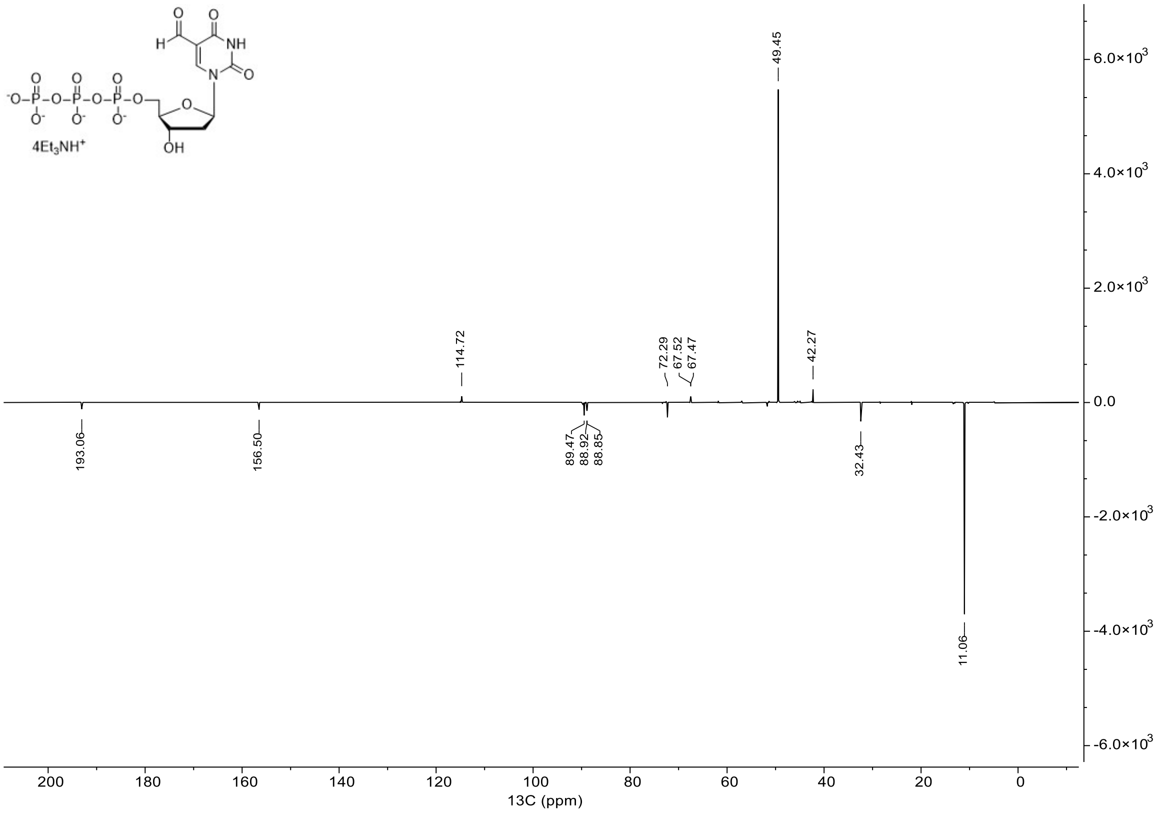

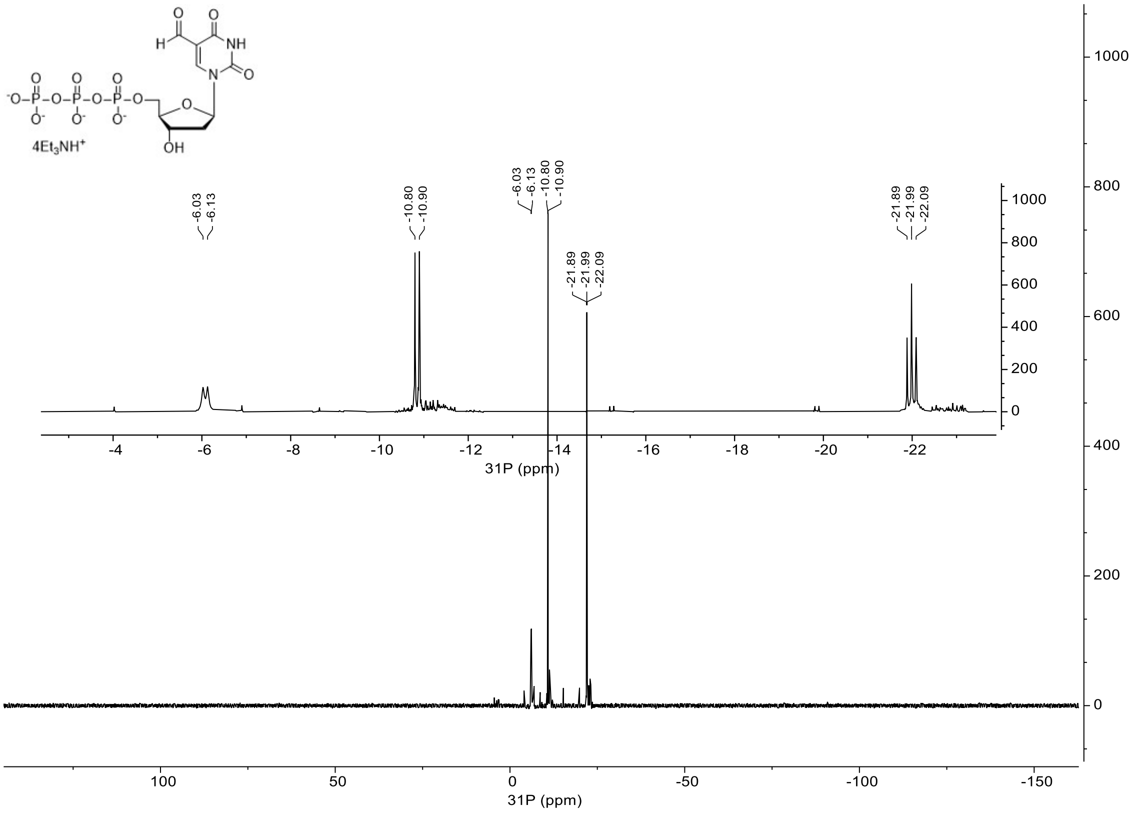

6.2. NMR spectra used for the assignment of epimers

^{1}H spectrum of **dC$^{\mathbf{Rhe}}$**

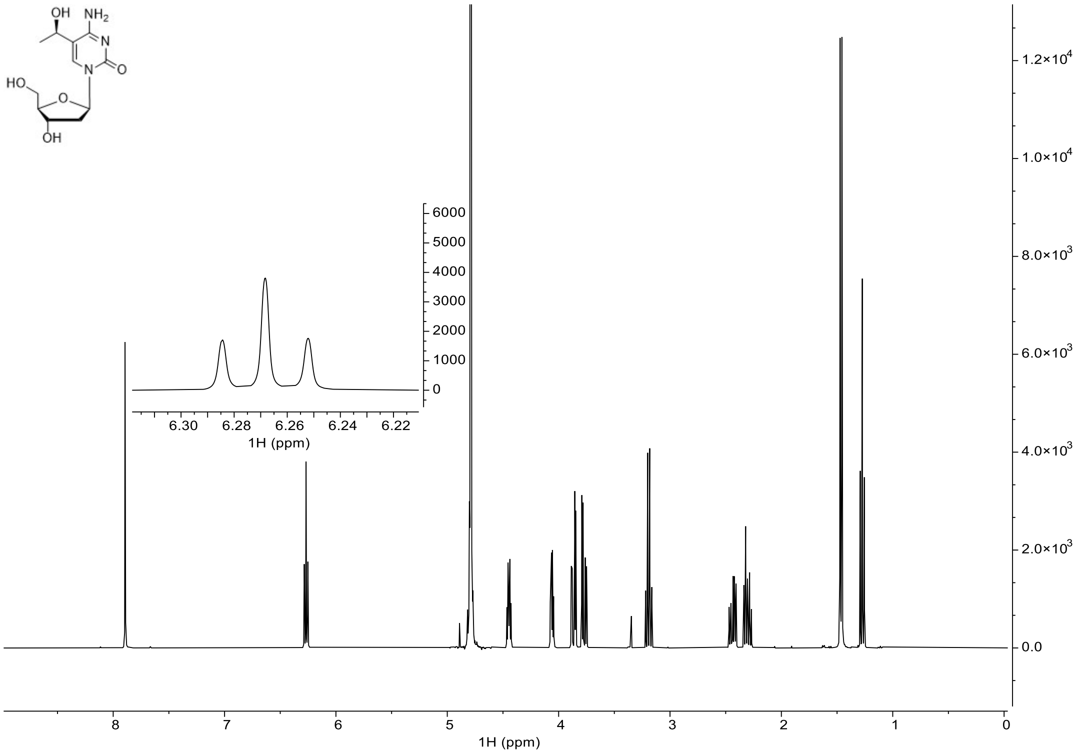

^1H spectrum of **dC^he** prepared from **dU^he_epA**

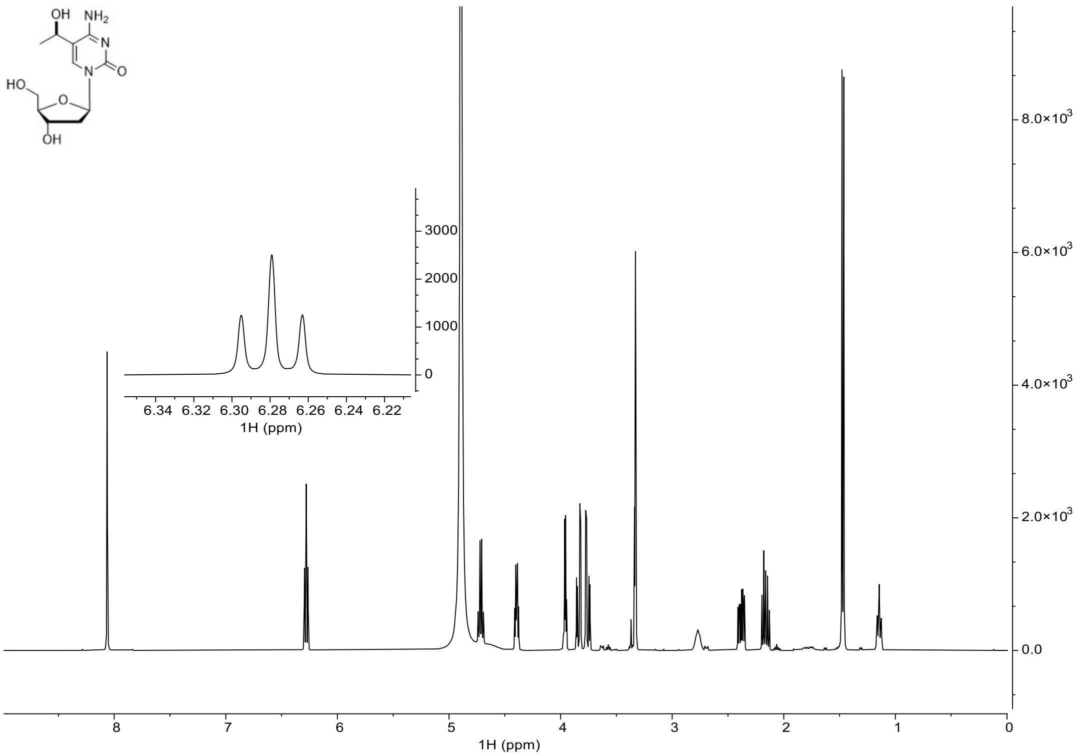

^1H spectrum of **dC^he** prepared from **dU^he_epA**, and **dC^Rhe**

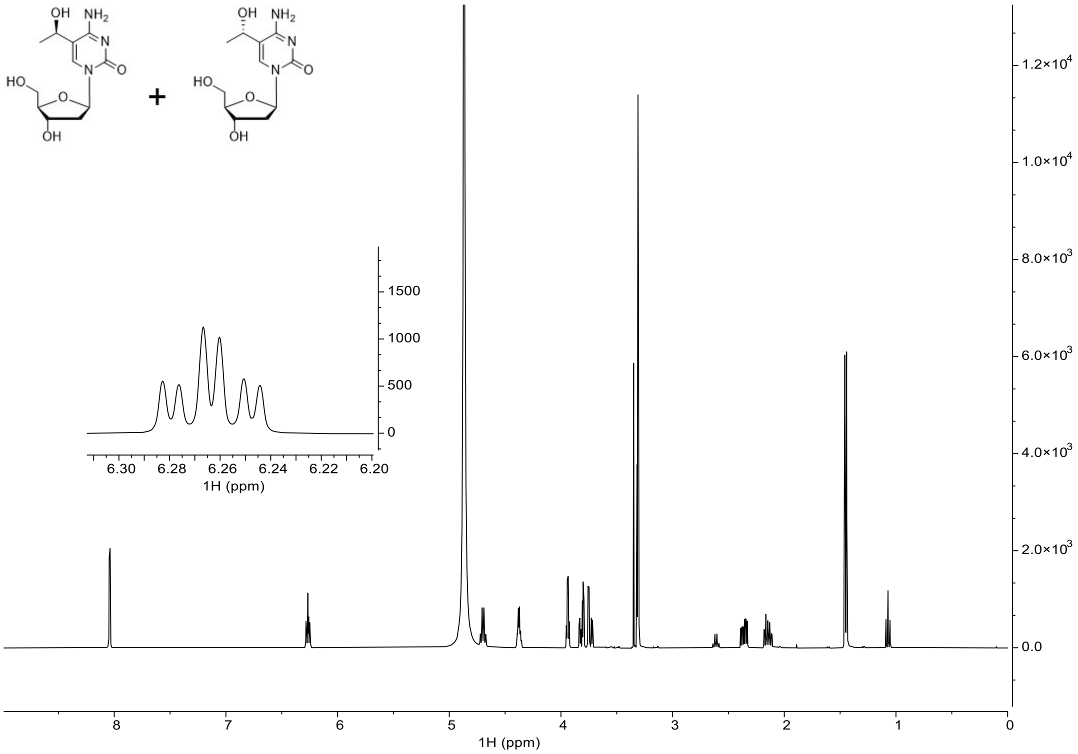

^{1}H spectrum of **dC$^{\mathbf{Shp}}$**

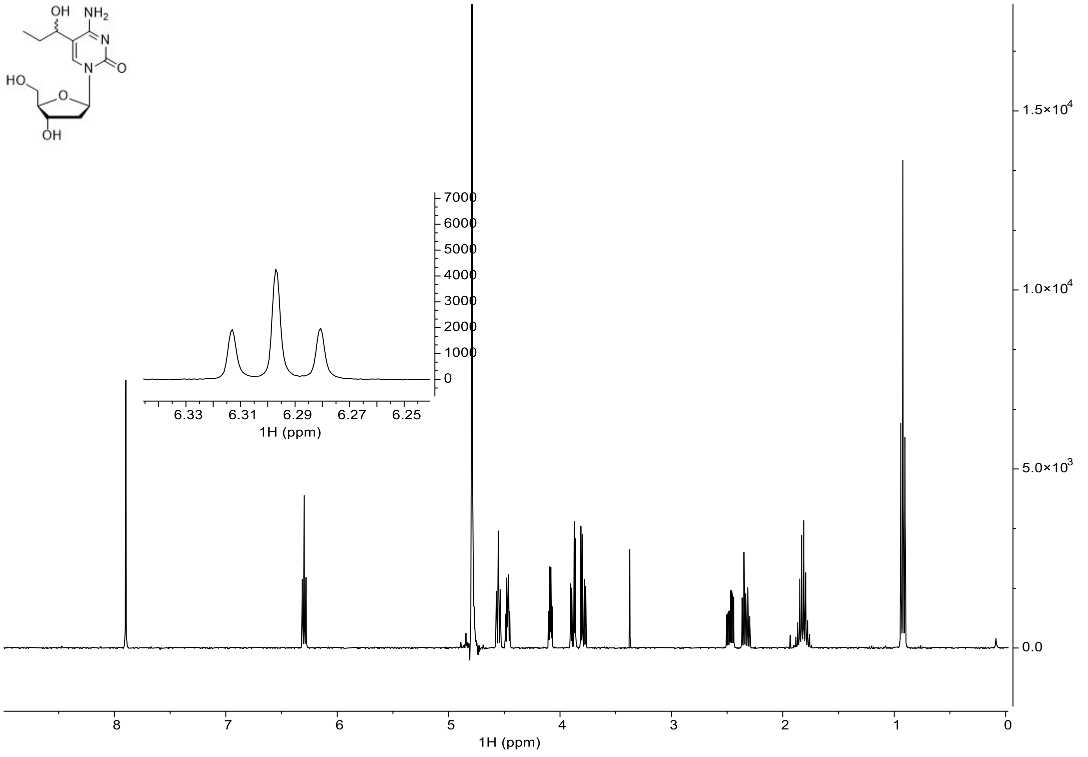

^{1}H spectrum of **dC$^{\mathbf{hp}}$** prepared from **dU$^{\mathbf{hp}}$_epB**

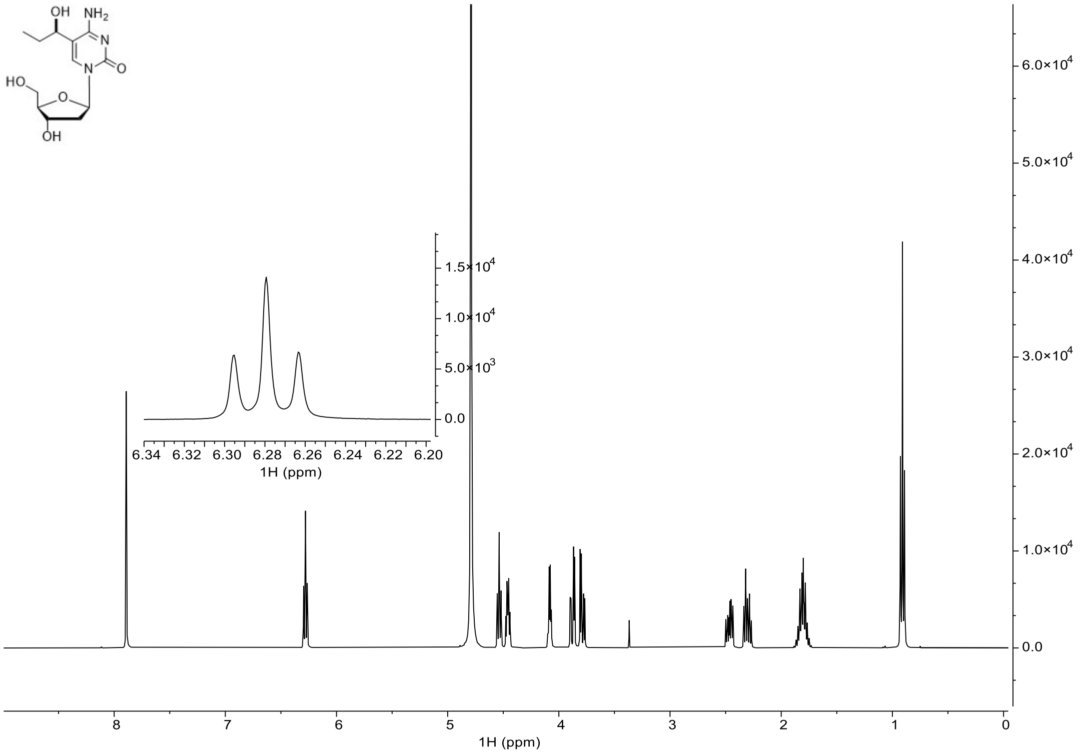

^{1}H spectrum of **dC^hp^** prepared from **dU^hp^_epB**, and **dC^Shp^**

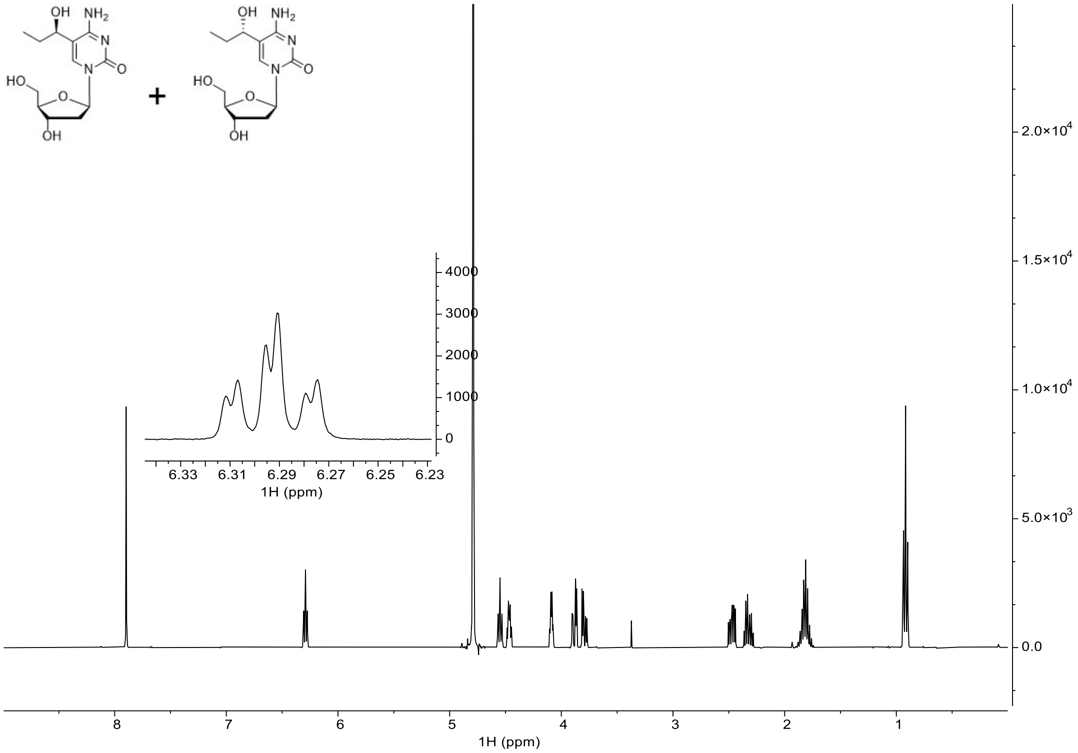

7. Sanger sequencing

Natural and modified templates used for transcription studies were sequenced from both directions (see sections 2.6.3. and 2.6.7.). Just in case of **235DNA_C^{She}**, **235DNA_C^{Rhp}** and **235DNA_C^{Shp}**, the sequencing didn't provide good enough results even after multiple attempts (see below) and therefore the full sequence could not be read properly.

7.1. Results of Sanger sequencing

Temp^{235} (natural control)

A) Sequencing chromatogram with forward primer Prim$^{FOR\text{-}235\text{-}long}$

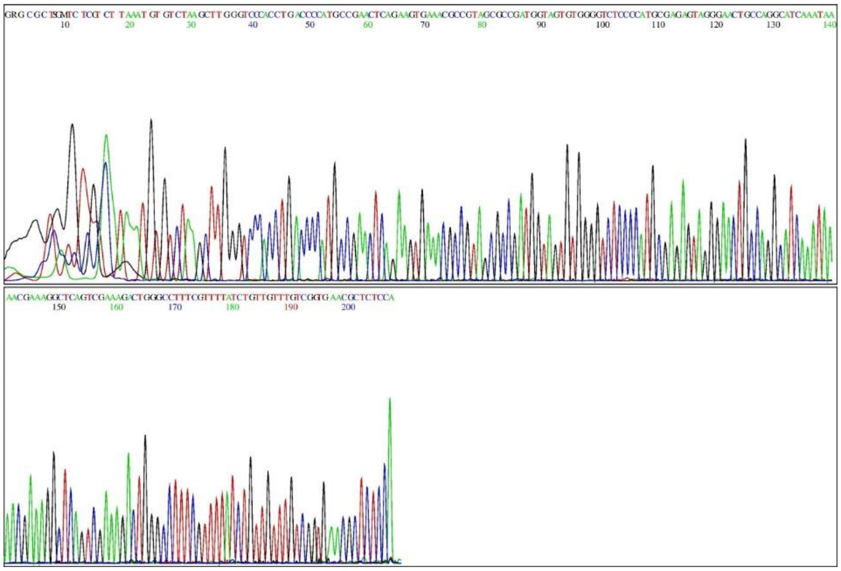

B) Sequencing chromatogram with reverse primer Prim$^{REV\text{-}235}$

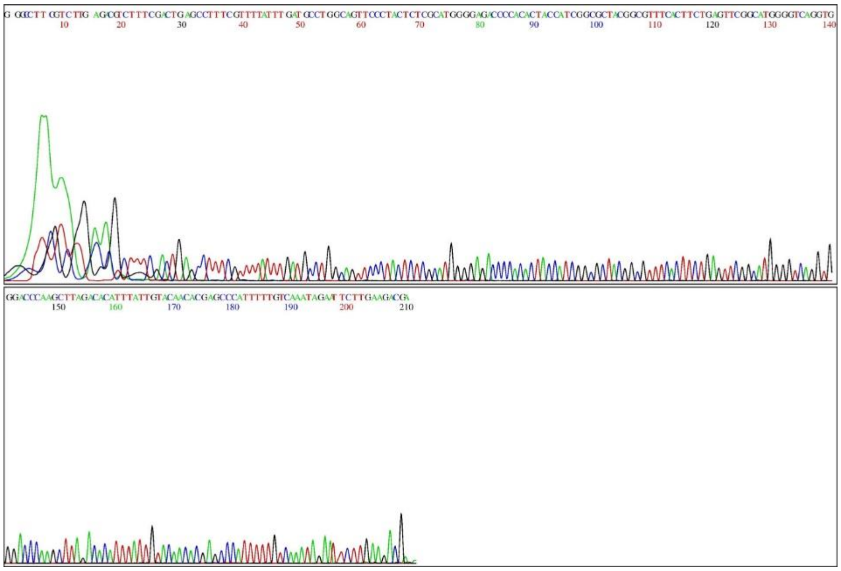

C) region of properly analyzed sequence depicted in blue box

CGTCTTCAAGAATTCTATTTGACAAAAATGGGCTCGTGTTGTACAATAAATGTGTCT
AAGCTTGGGTCCCACCTGACCCCATGCCGAACTCAGAAGTGAAACGCCGTAGCGCC
GATGGTAGTGTGGGGTCTCCCCATGCGAGAGTAGGGAACTGCCAGGCATCAAATAA
AACGAAAGGCTCAGTCGAAAGACTGGGCCTTTCGTTTTATCTGTTGTTTGTCGGTGA
ACGCTCTCC

235DNA_dU^{et}

A) Sequencing chromatogram with forward primer Prim$^{FOR\text{-}235\text{-}long}$

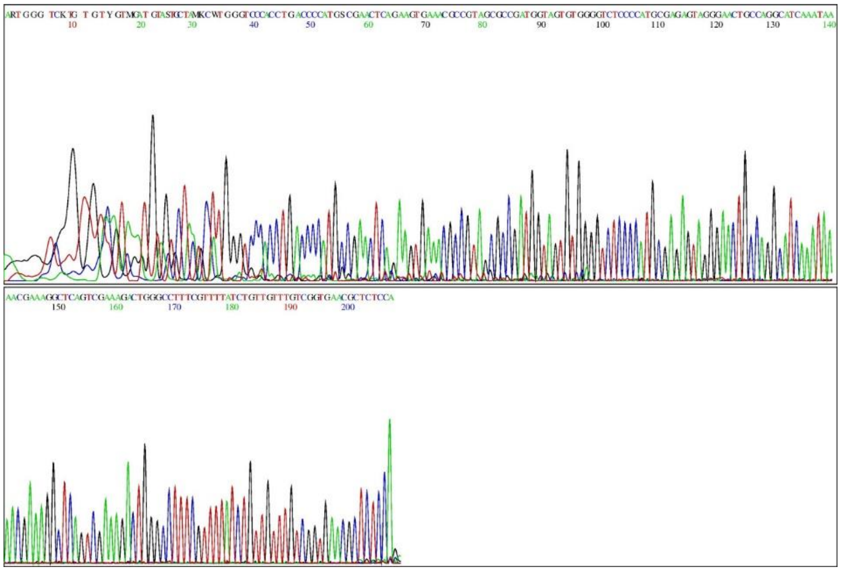

B) Sequencing chromatogram with reverse primer Prim$^{REV\text{-}235}$

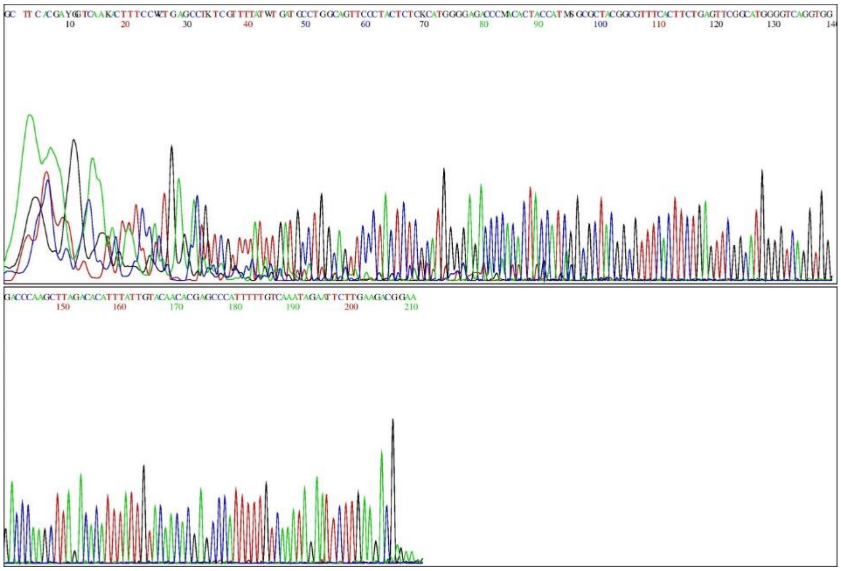

C) region of properly analyzed sequence depicted in blue box

CGTCTTCAAGAATTCTATTTGACAAAAATGGGCTCGTGTTGTACAATAAATGTGTCT
AAGCTTGGGTCCCACCTGACCCCATGCCGAACTCAGAAGTGAAACGCCGTAGCGCC
GATGGTAGTGTGGGGTCTCCCCATGCGAGAGTAGGGAACTGCCAGGCATCAAATAA
AACGAAAGGCTCAGTCGAAAGACTGGGCCTTTCGTTTTATCTGTTGTTTGTCGGTGA
ACGCTCTCC

235DNA_dU^{pr}

A) Sequencing chromatogram with forward primer $Prim^{FOR-235-long}$

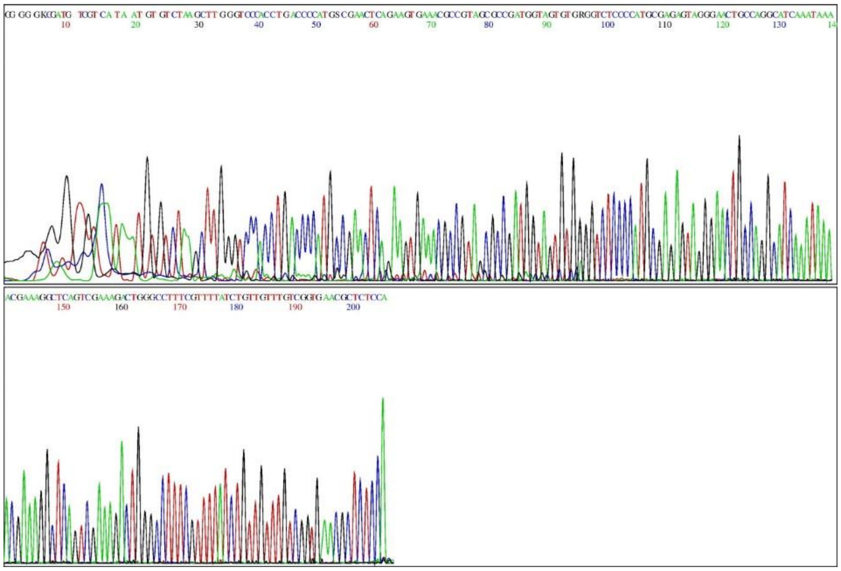

B) Sequencing chromatogram with reverse primer $Prim^{REV-235}$

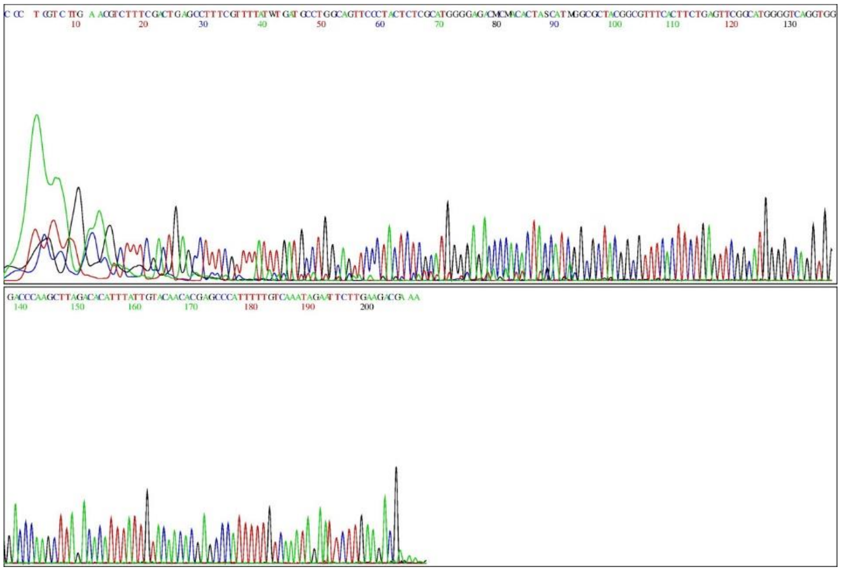

C) region of properly analyzed sequence depicted in blue box

CGTCTTCAAGAATTCTATTTGACAAAAATGGGCTCGTGTTGTACAATAAATGTGTCT
AAGCTTGGGTCCCACCTGACCCCATGCCGAACTCAGAAGTGAAACGCCGTAGCGCC
GATGGTAGTGTGGGGTCTCCCCATGCGAGAGTAGGGAACTGCCAGGCATCAAATAA
AACGAAAGGCTCAGTCGAAAGACTGGGCCTTTCGTTTTATCTGTTGTTTGTCGGTGA
ACGCTCTCC

235DNA_dU^{She}

A) Sequencing chromatogram with forward primer Prim$^{FOR-235-long}$

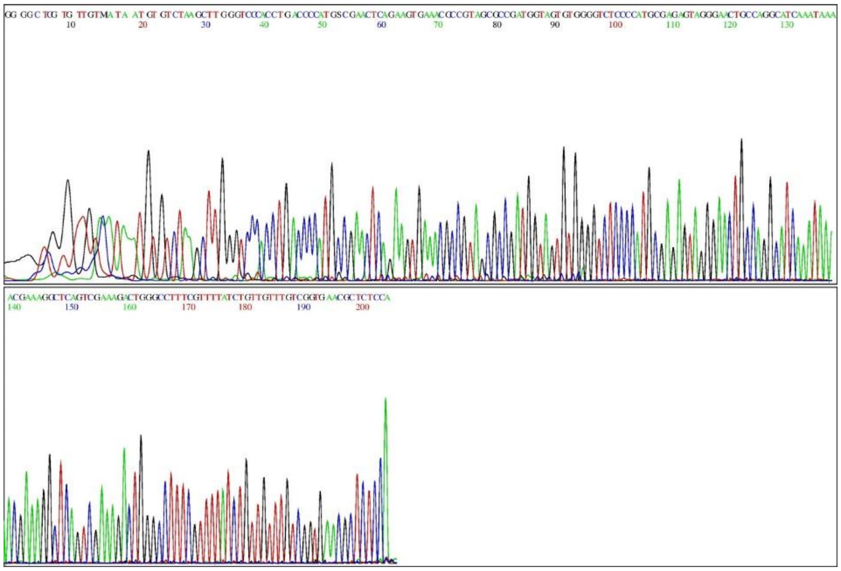

B) Sequencing chromatogram with reverse primer Prim$^{REV-235}$

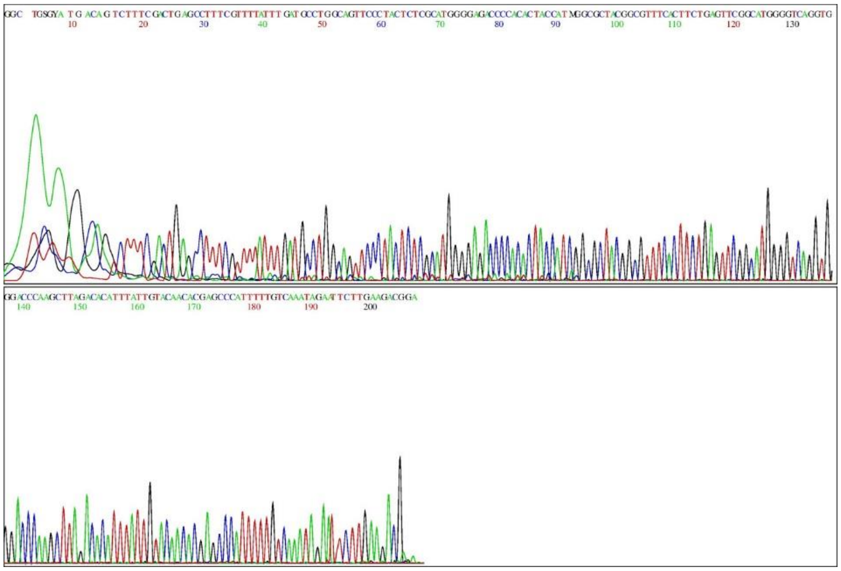

C) region of properly analyzed sequence depicted in blue box

CGTCTTCAAGAATTCTATTTGACAAAAATGGGCTCGTGTTGTACAATAAATGTGTCT
AAGCTTGGGTCCCACCTGACCCCATGCCGAACTCAGAAGTGAAACGCCGTAGCGCC
GATGGTAGTGTGGGGTCTCCCCATGCGAGAGTAGGGAACTGCCAGGCATCAAATAA
AACGAAAGGCTCAGTCGAAAGACTGGGCCTTTCGTTTTATCTGTTGTTTGTCGGTGA
ACGCTCTCC

235DNA_dU^{Shp}

A) Sequencing chromatogram with forward primer Prim$^{FOR\text{-}235\text{-}long}$

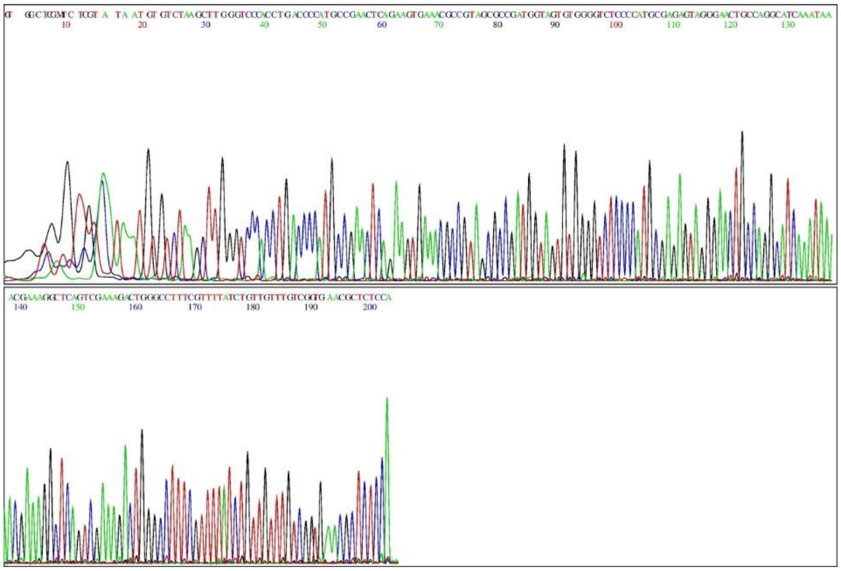

B) Sequencing chromatogram with reverse primer Prim$^{REV\text{-}235}$

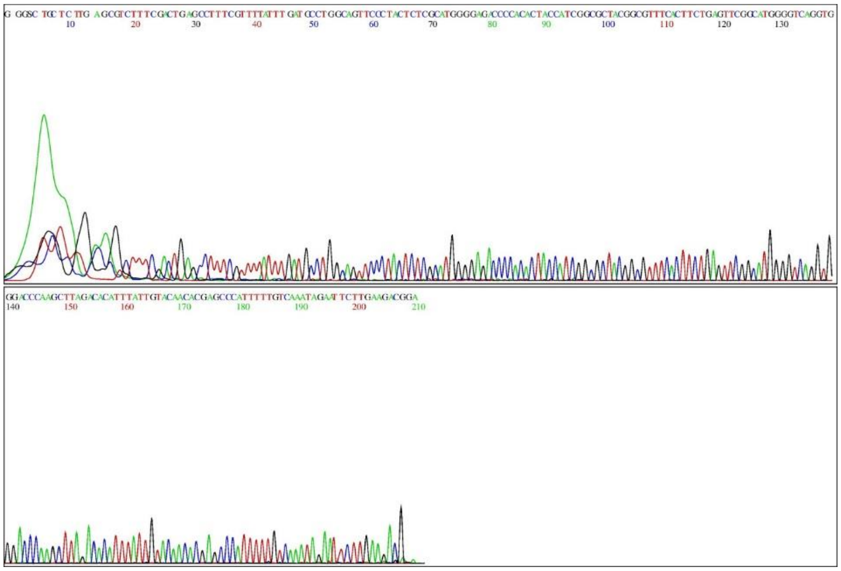

C) region of properly analyzed sequence depicted in blue box

CGTCTTCAAGAATTCTATTTGACAAAAATGGGCTCGTGTTGTACAATAAATGTGTCT
AAGCTTGGGTCCCACCTGACCCCATGCCGAACTCAGAAGTGAAACGCCGTAGCGCC
GATGGTAGTGTGGGGTCTCCCCATGCGAGAGTAGGGAACTGCCAGGCATCAAATAA
AACGAAAGGCTCAGTCGAAAGACTGGGCCTTTCGTTTTATCTGTTGTTTGTCGGTGA
ACGCTCTCC

235DNA_U^{ac}

A) Sequencing chromatogram with forward primer Prim$^{FOR-235-long}$

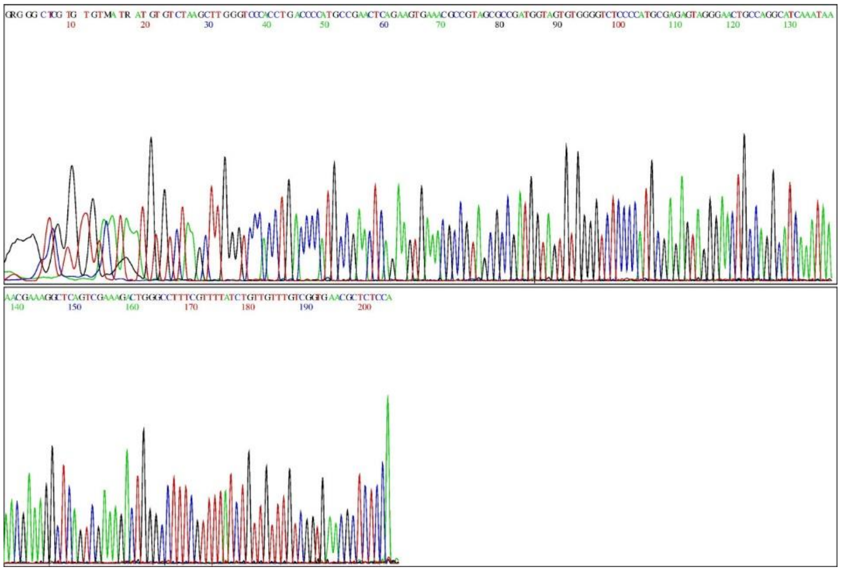

B) Sequencing chromatogram with reverse primer Prim$^{REV-235}$

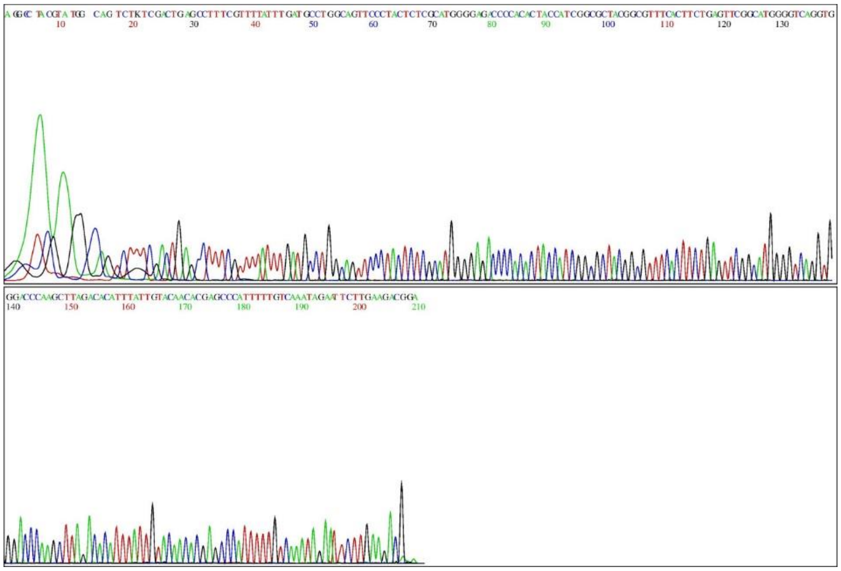

C) region of properly analyzed sequence depicted in blue box

CGTCTTCAAGAATTCTATTTGACAAAAATGGGCTCGTGTTGTACAATAAATGTGTCT
AAGCTTGGGTCCCACCTGACCCCATGCCGAACTCAGAAGTGAAACGCCGTAGCGCC
GATGGTAGTGTGGGGTCTCCCCATGCGAGAGTAGGGAACTGCCAGGCATCAAATAA
AACGAAAGGCTCAGTCGAAAGACTGGGCCTTTCGTTTTATCTGTTGTTTGTCGGTGA
ACGCTCTCC

235DNA_U^{pp}

A) Sequencing chromatogram with forward primer Prim$^{FOR\text{-}235\text{-}long}$

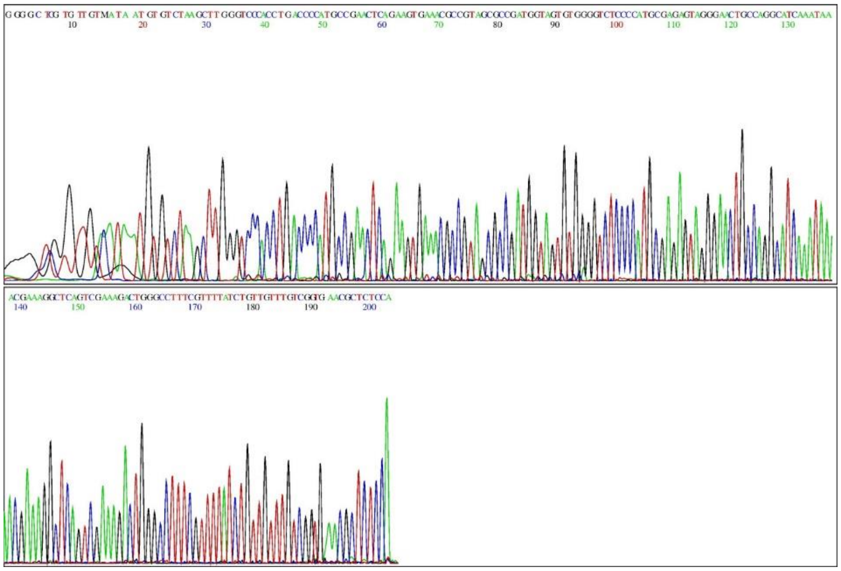

B) Sequencing chromatogram with reverse primer Prim$^{REV\text{-}235}$

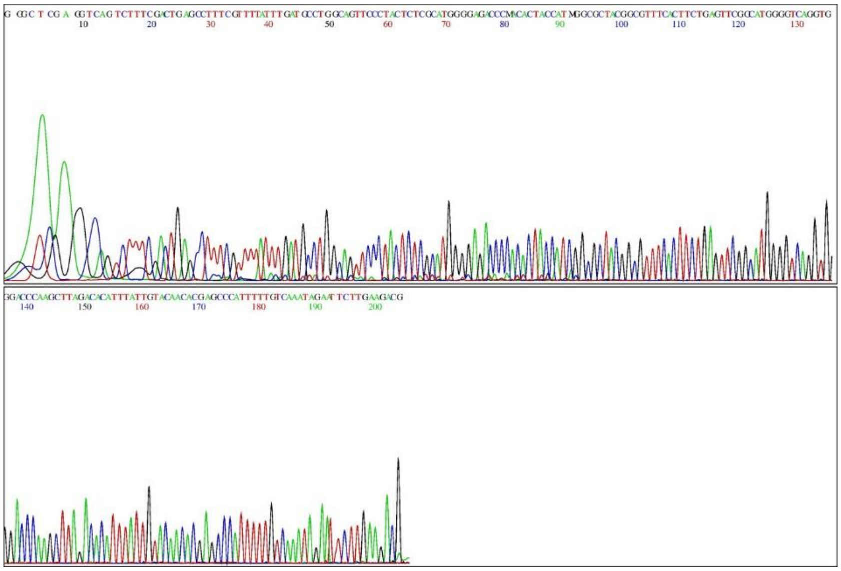

C) region of properly analyzed sequence depicted in blue box

CGTCTTCAAGAATTCTATTTGACAAAAATGGGCTCGTGTTGTACAATAAATGTGTCT
AAGCTTGGGTCCCACCTGACCCCATGCCGAACTCAGAAGTGAAACGCCGTAGCGCC
GATGGTAGTGTGGGGTCTCCCCATGCGAGAGTAGGGAACTGCCAGGCATCAAATAA
AACGAAAGGCTCAGTCGAAAGACTGGGCCTTTCGTTTTATCTGTTGTTTGTCGGTGA
ACGCTCTCC

235DNA_dC^{et}

A) Sequencing chromatogram with forward primer Prim$^{FOR\text{-}235\text{-}long}$

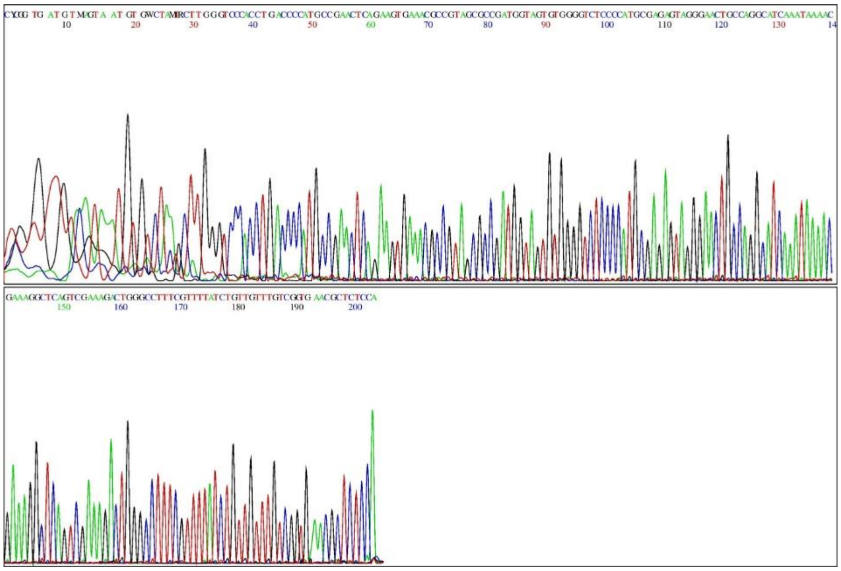

B) Sequencing chromatogram with reverse primer Prim$^{REV\text{-}235}$

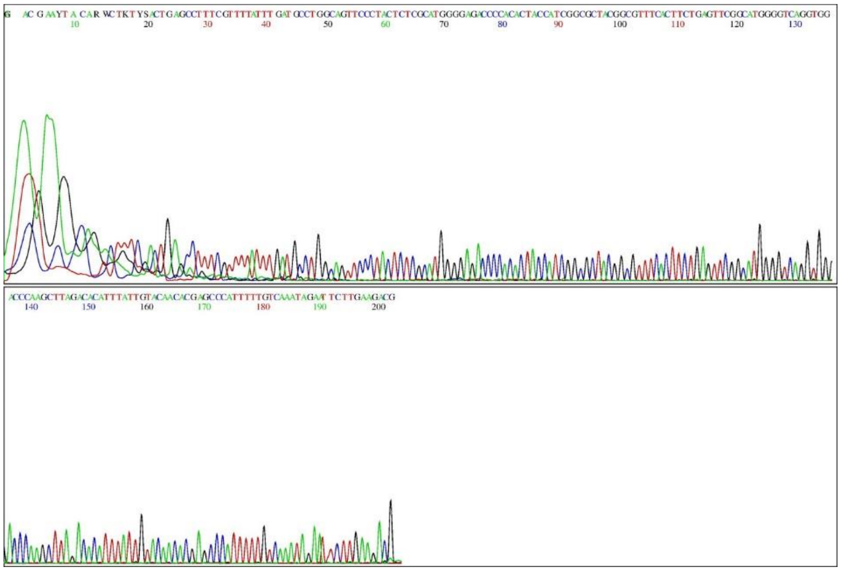

C) region of properly analyzed sequence depicted in blue box

CGTCTTCAAGAATTCTATTTGACAAAAATGGGCTCGTGTTGTACAATAAATGTGTCT
AAGCTTGGGTCCCACCTGACCCCATGCCGAACTCAGAAGTGAAACGCCGTAGCGCC
GATGGTAGTGTGGGGTCTCCCCATGCGAGAGTAGGGAACTGCCAGGCATCAAATAA
AACGAAAGGCTCAGTCGAAAGACTGGGCCTTTCGTTTTATCTGTTGTTTGTCGGTGA
ACGCTCTCC

235DNA_dC^{pr}

A) Sequencing chromatogram with forward primer Prim$^{FOR-235-long}$

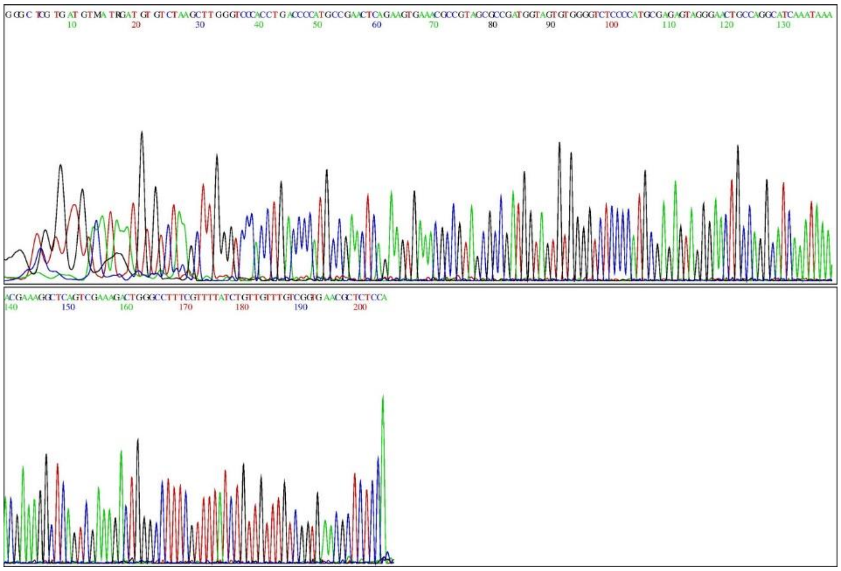

B) Sequencing chromatogram with reverse primer Prim$^{REV-235}$

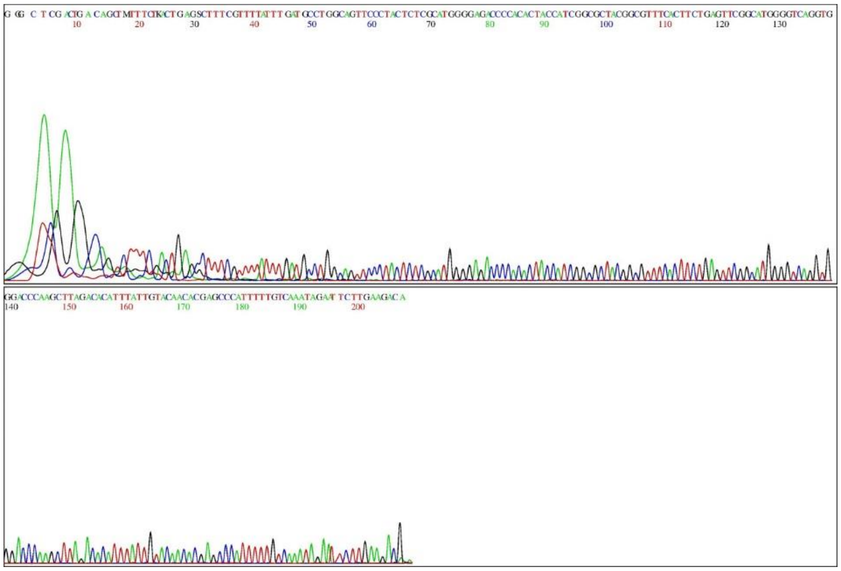

C) region of properly analyzed sequence depicted in blue box

CGTCTTCAAGAATTCTATTTGACAAAAATGGGCTCGTGTTGTACAATAAATGTGTCT
AAGCTTGGGTCCCACCTGACCCCATGCCGAACTCAGAAGTGAAACGCCGTAGCGCC
GATGGTAGTGTGGGGTCTCCCCATGCGAGAGTAGGGAACTGCCAGGCATCAAATAA
AACGAAAGGCTCAGTCGAAAGACTGGGCCTTTCGTTTTATCTGTTGTTTGTCGGTGA
ACGCTCTCC

235DNA_dC^{Rhe}

A) Sequencing chromatogram with forward primer Prim$^{FOR\text{-}235\text{-}long}$

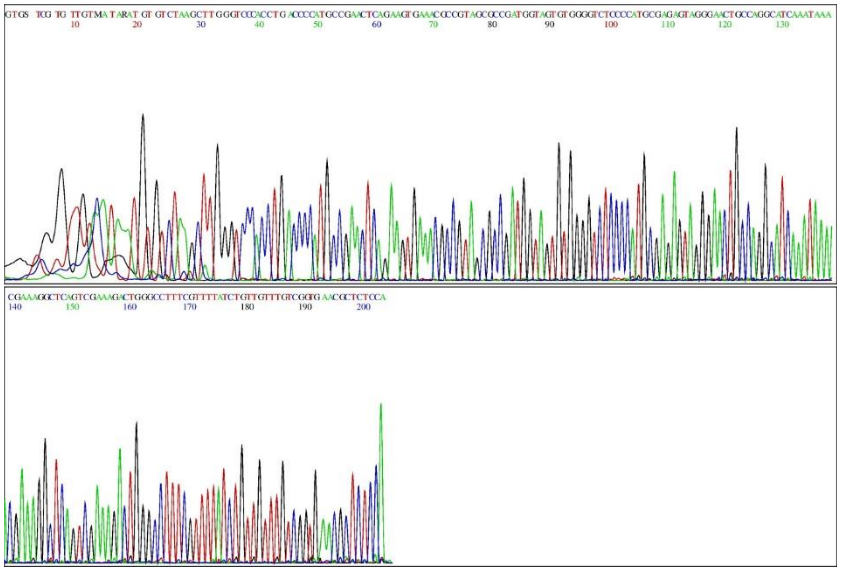

B) Sequencing chromatogram with reverse primer Prim$^{REV\text{-}235}$

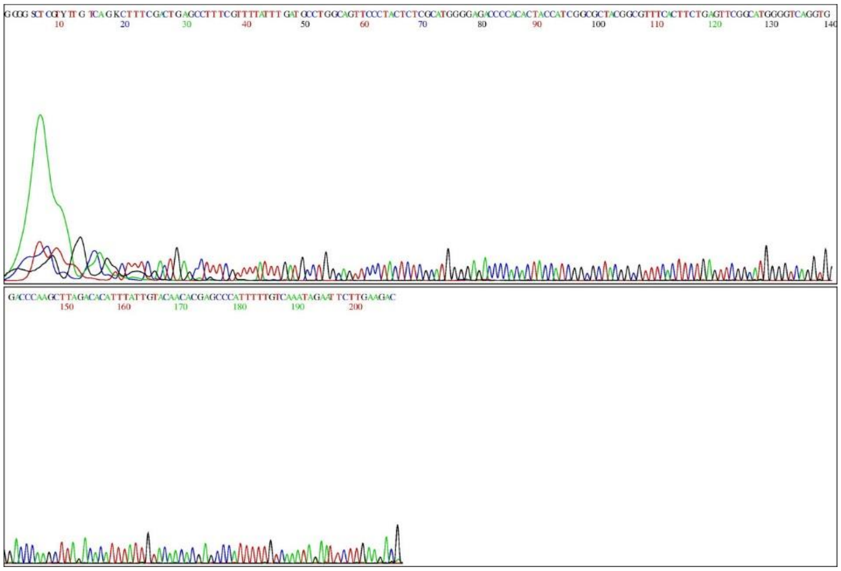

C) region of properly analyzed sequence depicted in blue box

CGTCTTCAAGAATTCTATTTGACAAAAATGGGCTCGTGTTGTACAATAAATGTGTCT
AAGCTTGGGTCCCACCTGACCCCATGCCGAACTCAGAAGTGAAACGCCGTAGCGCC
GATGGTAGTGTGGGGTCTCCCCATGCGAGAGTAGGGAACTGCCAGGCATCAAATAA
AACGAAAGGCTCAGTCGAAAGACTGGGCCTTTCGTTTTATCTGTTGTTTGTCGGTGA
ACGCTCTCC

235DNA_dC^She^

A) Sequencing chromatogram with forward primer Prim^FOR-235-long^

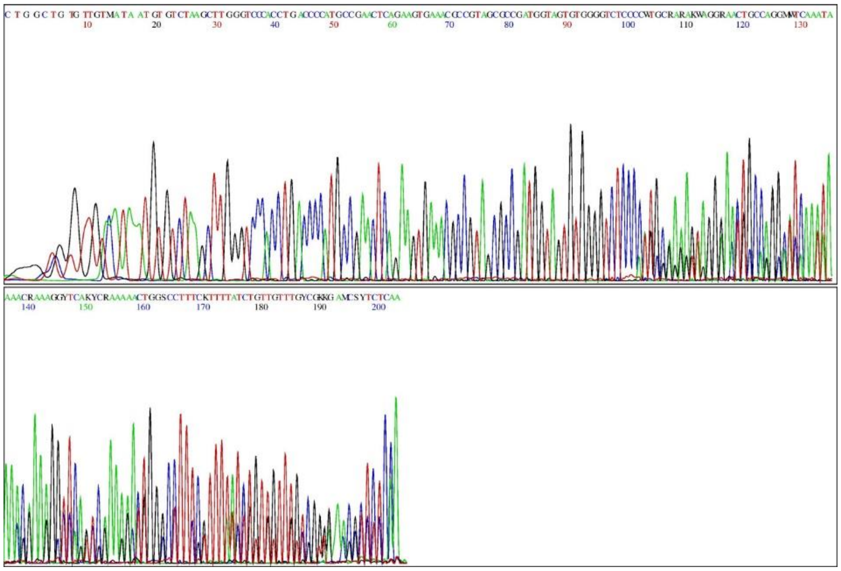

B) Sequencing chromatogram with reverse primer Prim^REV-235^

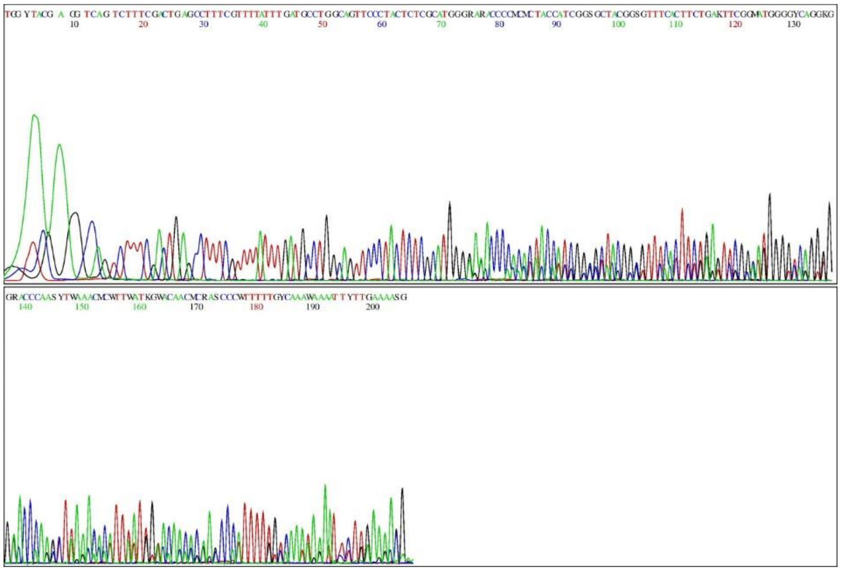

C) region of properly analyzed sequence depicted in blue box

CGTCTTCAAGAATTCTATTTGACAAAAATGGGCTCGTGTTGTACAATAAATGTGTCT
AAGCTTGGGTCCCACCTGACCCCATGCCGAACTCAGAAGTGAAACGCCGTAGCGCC
GATGGTAGTGTGGGGTCTCCCCATGCGAGAGTAGGGAACTGCCAGGCATCAAATAA
AACGAAAGGCTCAGTCGAAAGACTGGGCCTTTCGTTTTATCTGTTGTTTGTCGGTGA
ACGCTCTCC

235DNA_dC^{Rhp}

A) Sequencing chromatogram with forward primer Prim$^{FOR-235-long}$

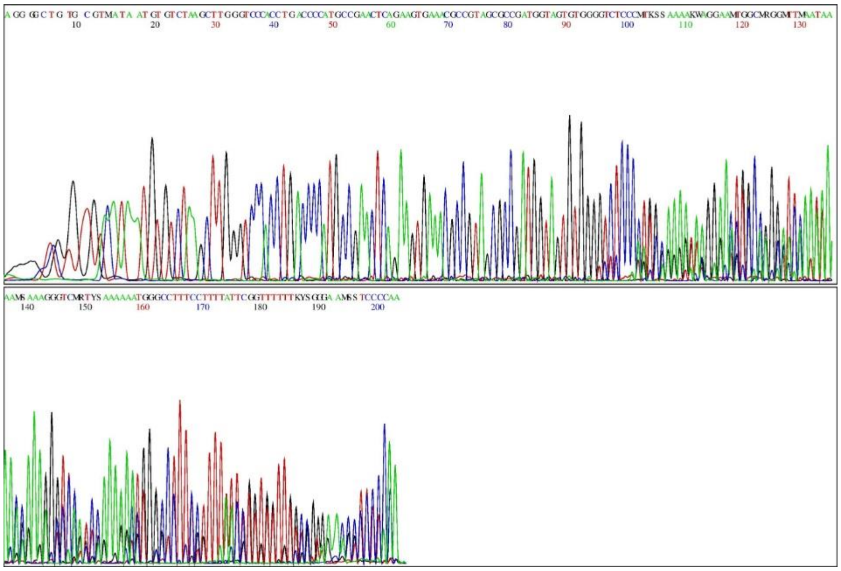

B) Sequencing chromatogram with reverse primer Prim$^{REV-235}$

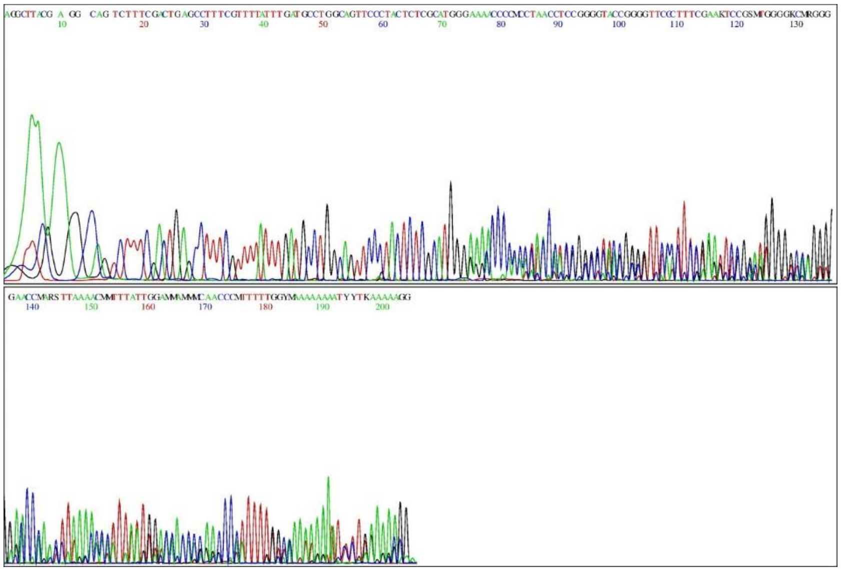

C) region of properly analyzed sequence depicted in blue box

CGTCTTCAAGAATTCTATTTGACAAAAATGGGCTCGTGTTGTACAATAAATGTGTCT
AAGCTTGGGTCCCACCTGACCCCATGCCGAACTCAGAAGTGAAACGCCGTAGCGCC
GATGGTAGTGTGGGGTCTCCCCATGCGAGAGTAGGGAACTGCCAGGCATCAAATAA
AACGAAAGGCTCAGTCGAAAGACTGGGCCTTTCGTTTTATCTGTTGTTTGTCGGTGA
ACGCTCTCC

235DNA_dC^{Shp}

A) Sequencing chromatogram with forward primer Prim$^{FOR\text{-}235\text{-}long}$

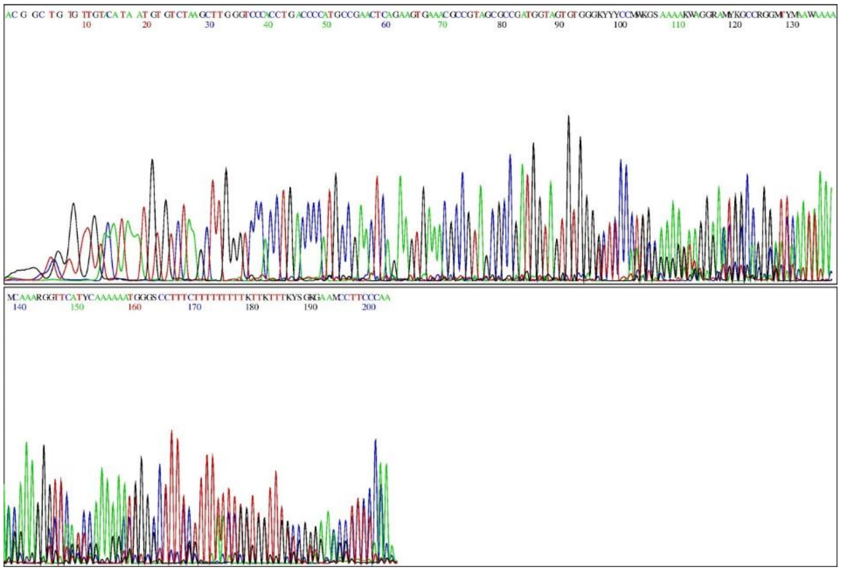

B) Sequencing chromatogram with reverse primer Prim$^{REV\text{-}235}$

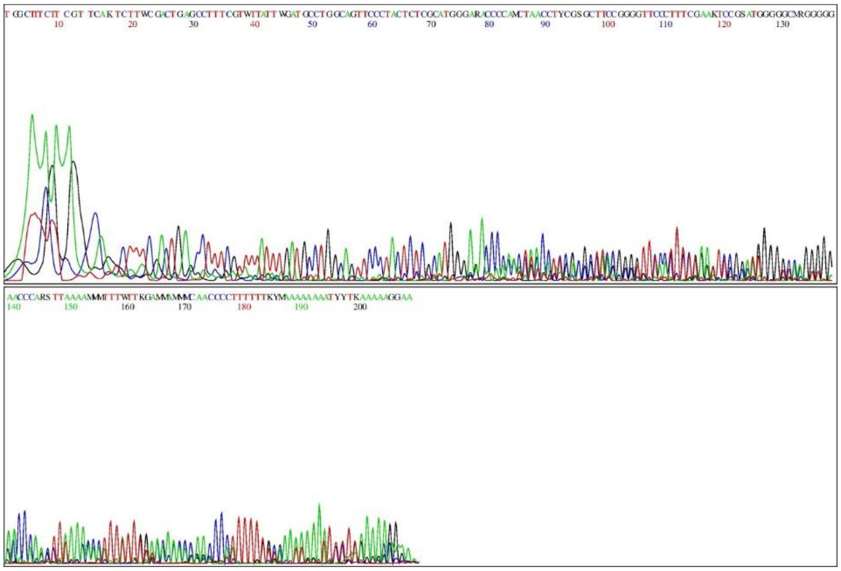

C) region of properly analyzed sequence depicted in blue box

CGTCTTCAAGAATTCTATTTGACAAAAATGGGCTCGTGTTGTACAATAAATGTGTCT
AAGCTTGGGTCCCACCTGACCCCATGCCGAACTCAGAAGTGAAACGCCGTAGCGCC
GATGGTAGTGTGGGGTCTCCCCATGCGAGAGTAGGGAACTGCCAGGCATCAAATAA
AACGAAAGGCTCAGTCGAAAGACTGGGCCTTTCGTTTTATCTGTTGTTTGTCGGTGA
ACGCTCTCC

235DNA_dC^ac

A) Sequencing chromatogram with forward primer Prim^FOR-235-long

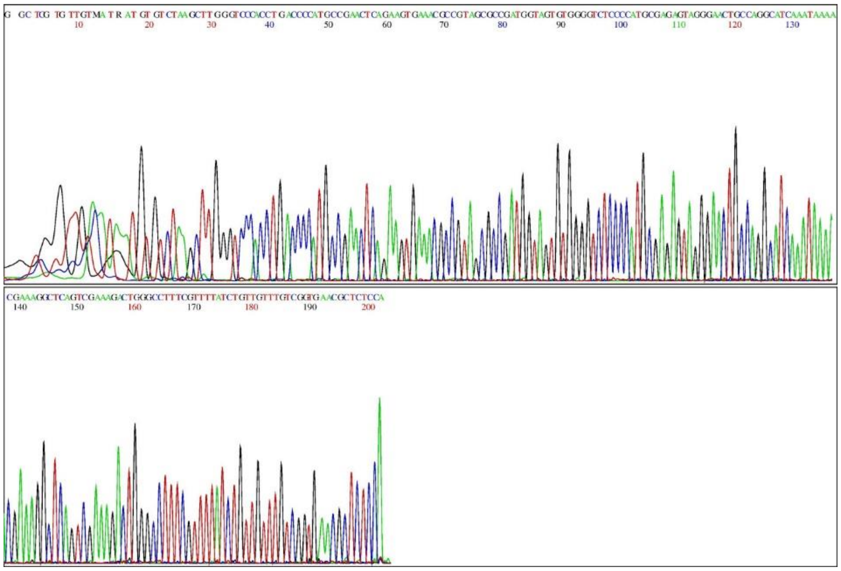

B) Sequencing chromatogram with reverse primer Prim^REV-235

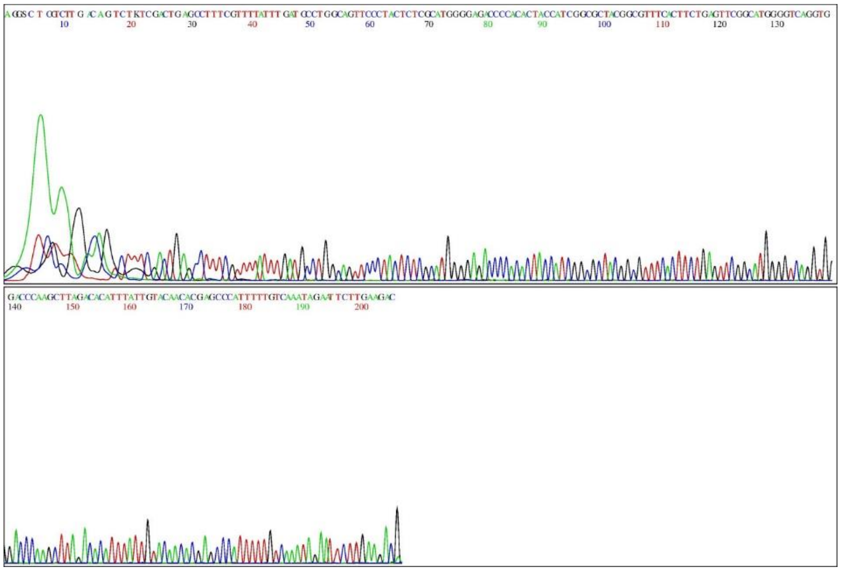

C) region of properly analyzed sequence depicted in blue box

CGTCTTCAAGAATTCTATTTGACAAAAATGGGCTCGTGTTGTACAATAAATGTGTCT
AAGCTTGGGTCCCACCTGACCCCATGCCGAACTCAGAAGTGAAACGCCGTAGCGCC
GATGGTAGTGTGGGGTCTCCCCATGCGAGAGTAGGGAACTGCCAGGCATCAAATAA
AACGAAAGGCTCAGTCGAAAGACTGGGCCTTTCGTTTTATCTGTTGTTTGTCGGTGA
ACGCTCTCC

235DNA_dC^{pp}

A) Sequencing chromatogram with forward primer $Prim^{FOR\text{-}235\text{-}long}$

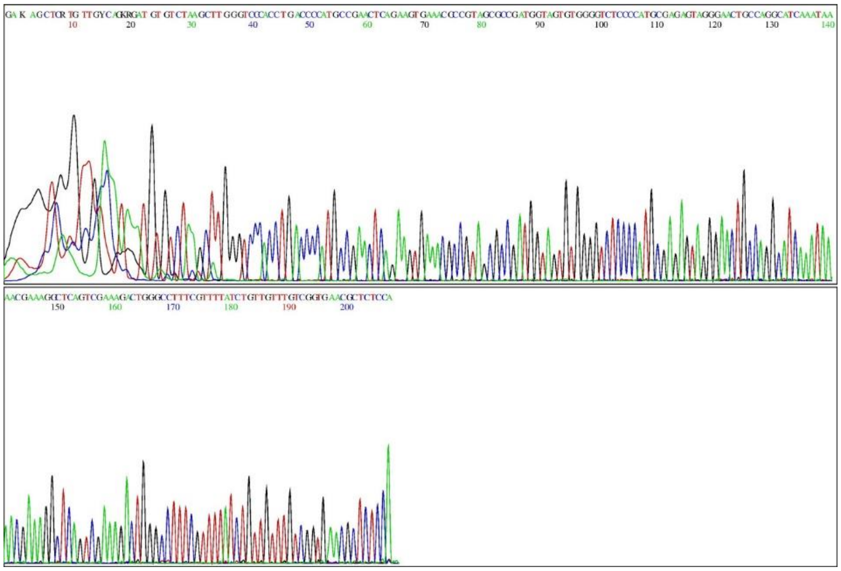

B) Sequencing chromatogram with reverse primer $Prim^{REV\text{-}235}$

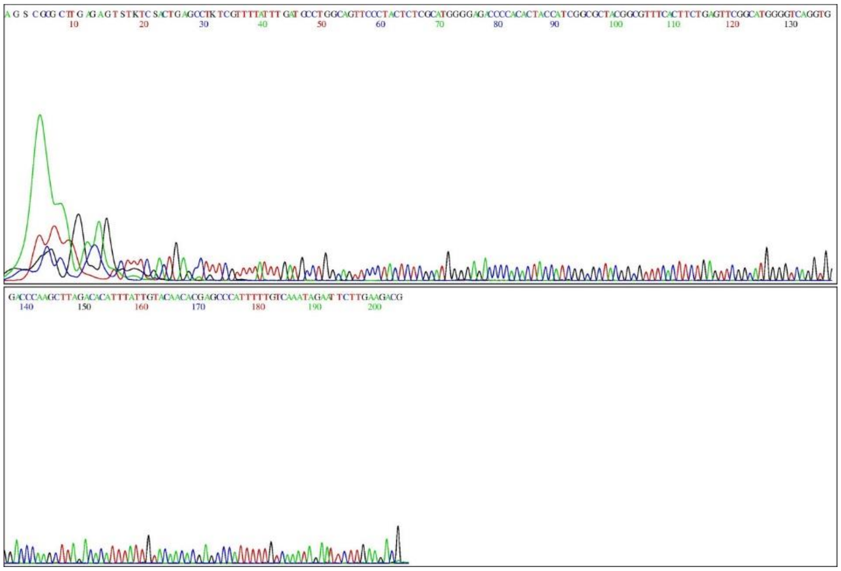

C) region of properly analyzed sequence depicted in blue box

CGTCTTCAAGAATTCTATTTGACAAAAATGGGCTCGTGTTGTACAATAAATGTGTCT
AAGCTTGGGTCCCACCTGACCCCATGCCGAACTCAGAAGTGAAACGCCGTAGCGCC
GATGGTAGTGTGGGGTCTCCCCATGCGAGAGTAGGGAACTGCCAGGCATCAAATAA
AACGAAAGGCTCAGTCGAAAGACTGGGCCTTTCGTTTTATCTGTTGTTTGTCGGTGA
ACGCTCTCC

235DNA_dU^{V}

A) Sequencing chromatogram with forward primer Prim$^{FOR-235-long}$

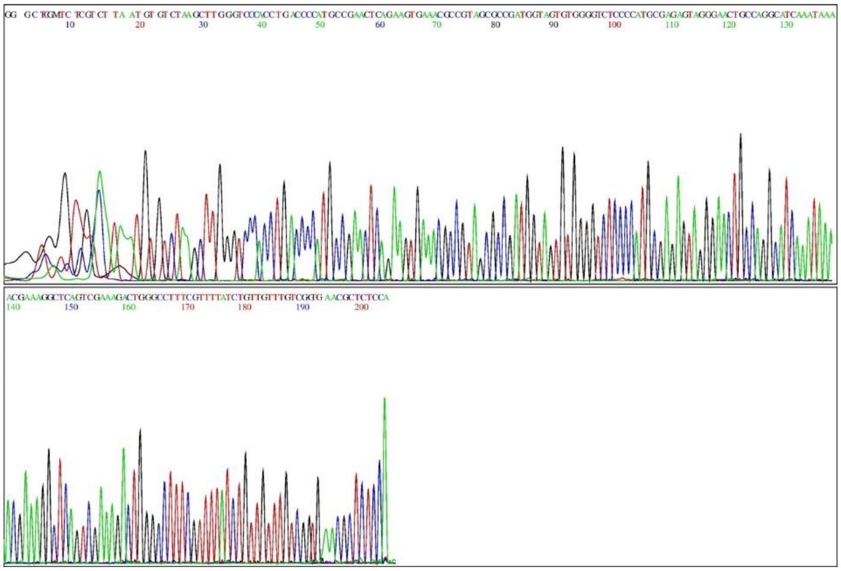

B) Sequencing chromatogram with reverse primer Prim$^{REV-235}$

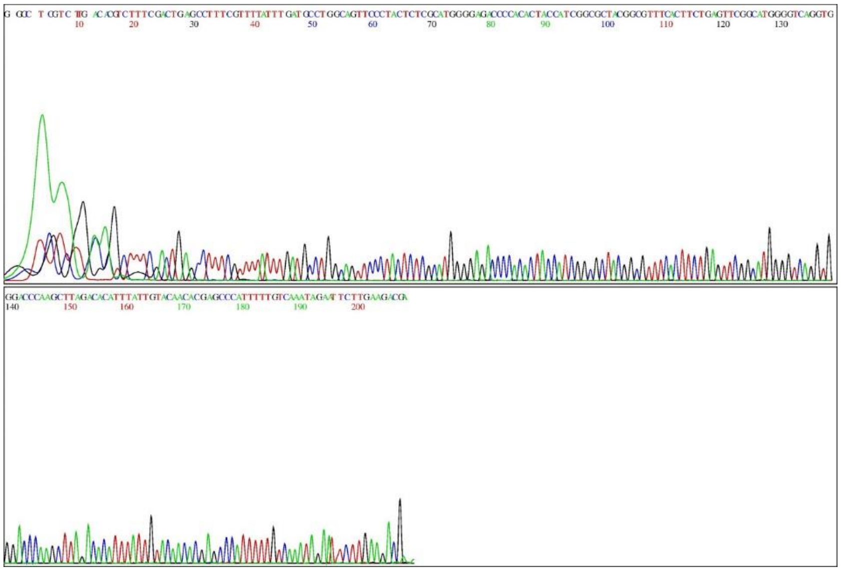

C) region of properly analyzed sequence depicted in blue box

CGTCTTCAAGAATTCTATTTGACAAAAATGGGCTCGTGTTGTACAATAAATGTGTCT
AAGCTTGGGTCCCACCTGACCCCATGCCGAACTCAGAAGTGAAACGCCGTAGCGCC
GATGGTAGTGTGGGGTCTCCCCATGCGAGAGTAGGGAACTGCCAGGCATCAAATAA
AACGAAAGGCTCAGTCGAAAGACTGGGCCTTTCGTTTTATCTGTTGTTTGTCGGTGA
ACGCTCTCC

235DNA_dU^{E}

A) Sequencing chromatogram with forward primer Prim$^{FOR-235-long}$

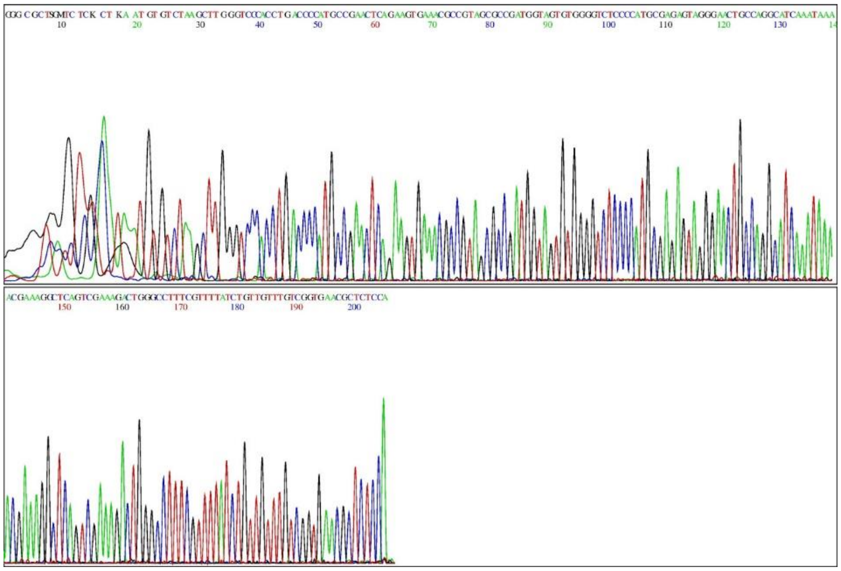

B) Sequencing chromatogram with reverse primer Prim$^{REV-235}$

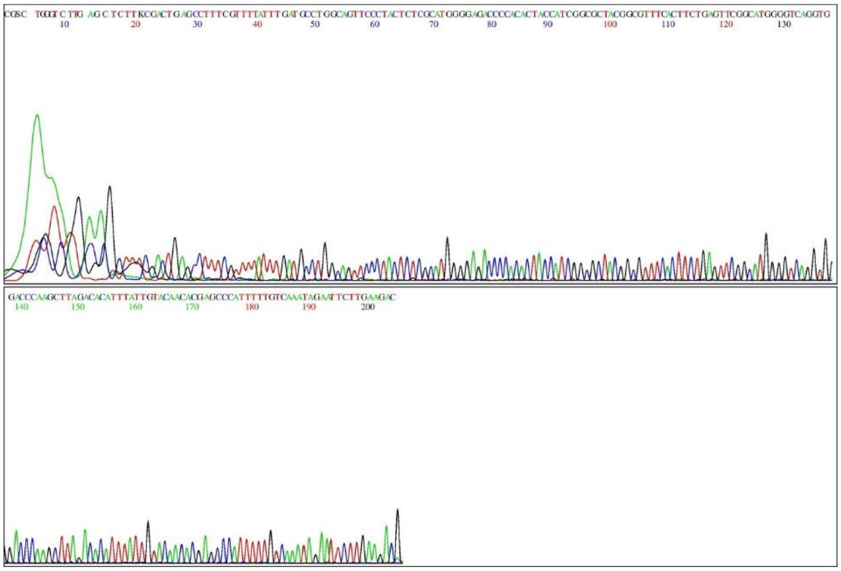

C) region of properly analyzed sequence depicted in blue box

CGTCTTCAAGAATTCTATTTGACAAAAATGGGCTCGTGTTGTACAATAAATGTGTCT
AAGCTTGGGTCCCACCTGACCCCATGCCGAACTCAGAAGTGAAACGCCGTAGCGCC
GATGGTAGTGTGGGGTCTCCCCATGCGAGAGTAGGGAACTGCCAGGCATCAAATAA
AACGAAAGGCTCAGTCGAAAGACTGGGCCTTTCGTTTTATCTGTTGTTTGTCGGTGA
ACGCTCTCC

235DNA_dC^f

A) Sequencing chromatogram with forward primer Prim^FOR-235-long

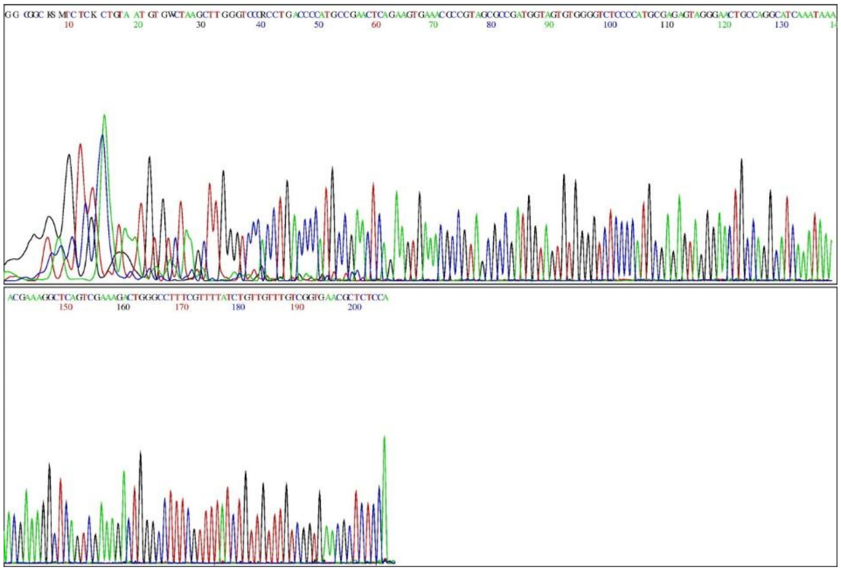

B) Sequencing chromatogram with reverse primer Prim^REV-235

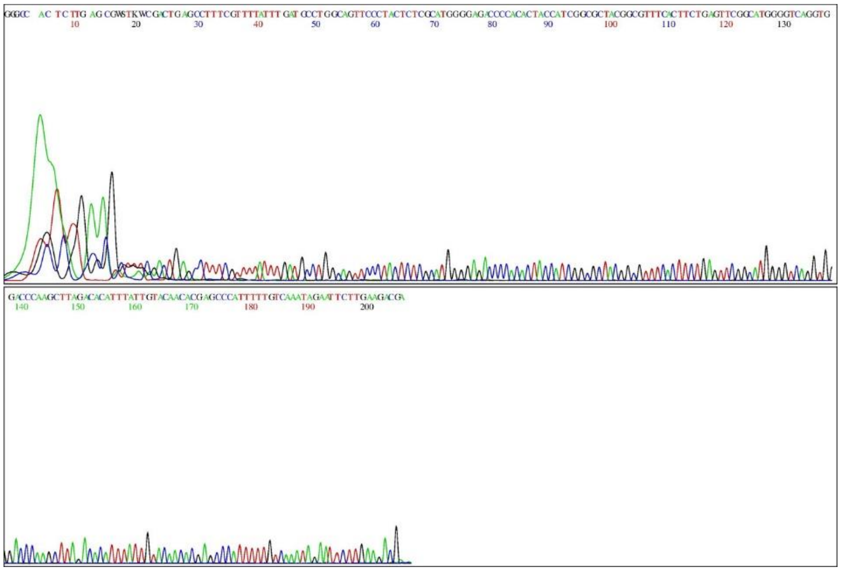

C) region of properly analyzed sequence depicted in blue box

CGTCTTCAAGAATTCTATTTGACAAAAATGGGCTCGTGTTGTACAATAAATGTGTCT
AAGCTTGGGTCCCACCTGACCCCATGCCGAACTCAGAAGTGAAACGCCGTAGCGCC
GATGGTAGTGTGGGGTCTCCCCATGCGAGAGTAGGGAACTGCCAGGCATCAAATAA
AACGAAAGGCTCAGTCGAAAGACTGGGCCTTTCGTTTTATCTGTTGTTTGTCGGTGA
ACGCTCTCC

235DNA_dC^{V}

A) Sequencing chromatogram with forward primer Prim$^{FOR\text{-}235\text{-}long}$

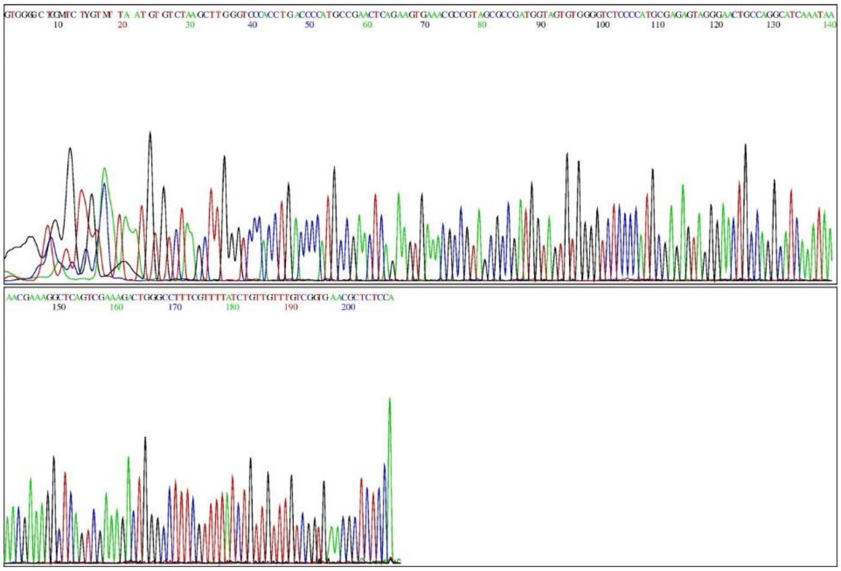

B) Sequencing chromatogram with reverse primer Prim$^{REV\text{-}235}$

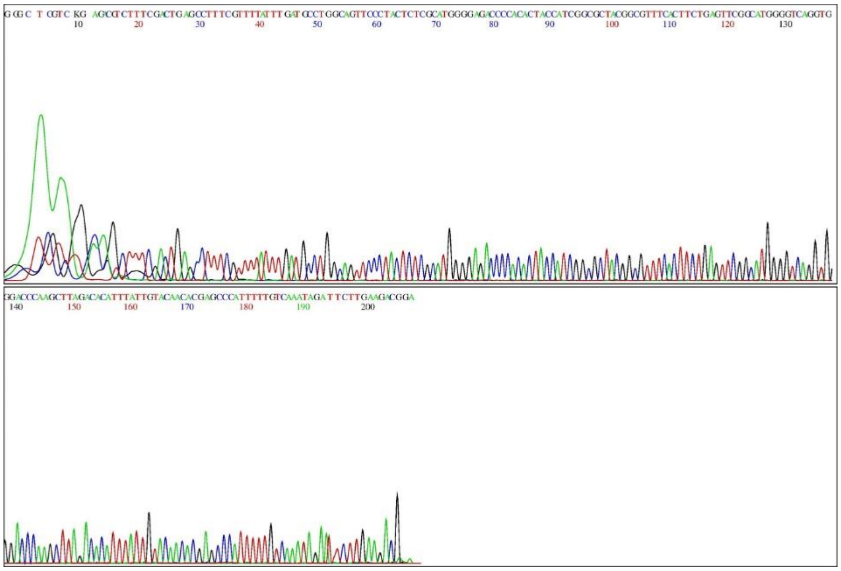

C) region of properly analyzed sequence depicted in blue box

CGTCTTCAAGAATTCTATTTGACAAAAATGGGCTCGTGTTGTACAATAAATGTGTCT
AAGCTTGGGTCCCACCTGACCCCATGCCGAACTCAGAAGTGAAACGCCGTAGCGCC
GATGGTAGTGTGGGGTCTCCCCATGCGAGAGTAGGGAACTGCCAGGCATCAAATAA
AACGAAAGGCTCAGTCGAAAGACTGGGCCTTTCGTTTTATCTGTTGTTTGTCGGTGA
ACGCTCTCC

235DNA_dC^E

A) Sequencing chromatogram with forward primer Prim^FOR-235-long

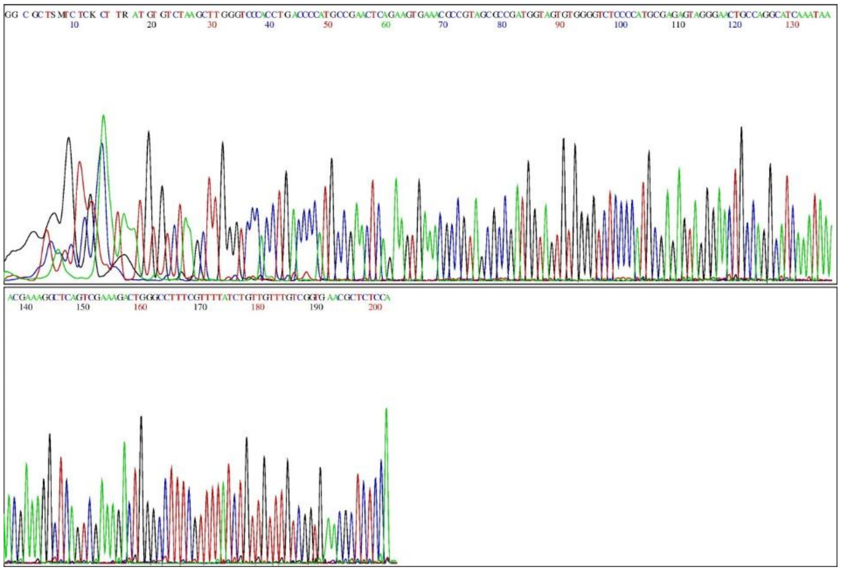

B) Sequencing chromatogram with reverse primer Prim^REV-235

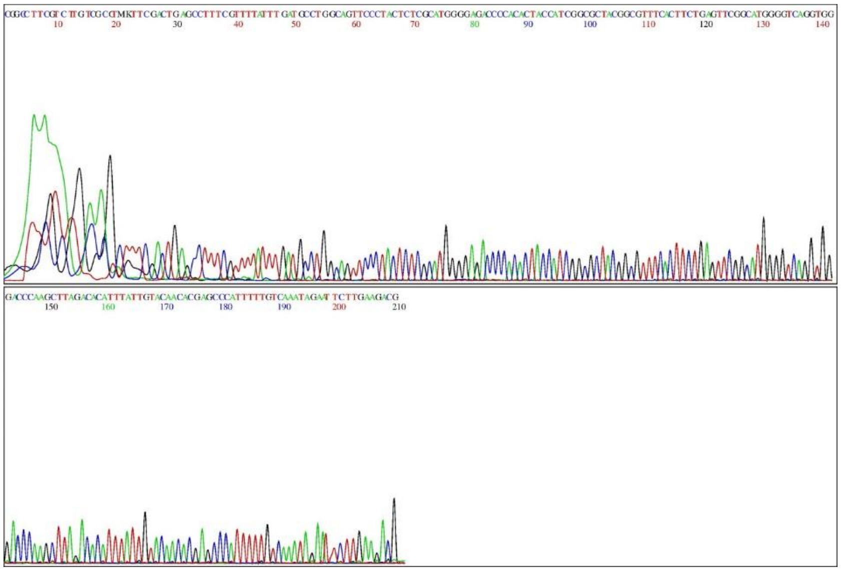

C) region of properly analyzed sequence depicted in blue box

CGTCTTCAAGAATTCTATTTGACAAAAATGGGCTCGTGTTGTACAATAAATGTGTCT
AAGCTTGGGTCCCACCTGACCCCATGCCGAACTCAGAAGTGAAACGCCGTAGCGCC
GATGGTAGTGTGGGGTCTCCCCATGCGAGAGTAGGGAACTGCCAGGCATCAAATAA
AACGAAAGGCTCAGTCGAAAGACTGGGCCTTTCGTTTTATCTGTTGTTTGTCGGTGA
ACGCTCTCC

8. References

1. V. Raindlová, M. Janoušková, M. Slavíčková, P. Perlíková, S. Boháčová, N. Milisavljevič, H. Šanderová, M. Benda, I. Barvík, L. Krásný and M. Hocek, *Nucleic Acids Research*, 2016, **44**, 3000–3012.

2. S. K. Chitneni, T. De Ruymaeker, J. Balzarini, A. M. Verbruggen and G. M. Bormans, *Journal of Labelled Compounds and Radiopharmaceuticals*, 2007, **50**, 649–655.

3. N. K. Andersen, H. Døssing, F. Jensen, B. Vester and P. Nielsen, *The Journal of Organic Chemistry*, 2011, **76**, 6177–6187.
4. G. J. Crouch and B. E. Eaton, *Nucleosides and Nucleotides*, 1994, **13**, 939–944.
5. Q. Dai and C. He, *Organic Letters*, 2011, **13**, 3446–3449.
6. T. Kovács and L. Ötvös, *Tetrahedron Letters*, 1988, **29**, 4525–4528.
7. D. E. Bergstrom and M. K. Ogawa, *Journal of the American Chemical Society*, 1978, **100**, 8106–8112.
8. A. L. Stuart, S. V. P. Kumar, S. V. Gupta, W. M. Zoghaib, S. Napper, K. C. Brown, S. Mannala and L. T. J. Delbaere, *Nucleosides and Nucleotides*, 1997, **16**, 2219–2231.
9. S. A. Ingale, H. Mei, P. Leonard and F. Seela, *The Journal of Organic Chemistry*, 2013, **78**, 11271–11282.
10. X.-A. Zheng, H.-S. Huang, R. Kong, W.-J. Chen, S.-S. Gong and Q. Sun, *Tetrahedron*, 2018, **74**, 7095–7101.
11. A. S. Jones, M. J. Slater and R. T. Walker, *J. Chem. Soc., Perkin Trans. 1*, 1987, 1325–1329.
12. A. Chentsova, E. Kapourani and A. Giannis, *Beilstein Journal of Organic Chemistry*, 2014, **10**, 7–11.
13. B. C. Froehler, S. Wadwani, T. J. Terhorst and S. R. Gerrard, *Tetrahedron Letters*, 1992, **33**, 5307–5310.
14. G. B. Heisig and H. M. Davis, *Journal of the American Chemical Society*, 1935, **57**, 339–340.

15. J. L. Ruth and D. E. Bergstrom, *The Journal of Organic Chemistry*, 1978, **43**, 2870–2876.

16. S. Schiesser, T. Pfaffeneder, K. Sadeghian, B. Hackner, B. Steigenberger, A. S. Schröder, J. Steinbacher, G. Kashiwazaki, G. Höfner, K. T. Wanner, C. Ochsenfeld and T. Carell, *Journal of the American Chemical Society*, 2013, **135**, 14593–14599.

17. P. Röthlisberger, F. Levi-Acobas and M. Hollenstein, *Bioorganic & Medicinal Chemistry Letters*, 2017, **27**, 897–900.

18. Q. Sun, J. Sun, S.-S. Gong, C.-J. Wang, S.-Z. Pu and F.-D. Feng, *RSC Adv.*, 2014, **4**, 36036–36039.

19. B. H. Le, J. C. Koo, H. N. Joo and Y. J. Seo, *Bioorganic & Medicinal Chemistry*, 2017, **25**, 3591–3596.

20. H. Cahová, L. Havran, P. Brázdilová, H. Pivoňková, R. Pohl, M. Fojta and M. Hocek, *Angewandte Chemie International Edition*, 2008, **47**, 2059–2062.

21. J. Dadová, M. Vrábel, M. Adámik, M. Brázdová, R. Pohl, M. Fojta and M. Hocek, *Chemistry – A European Journal*, 2015, **21**, 16091–16102.

22. M. Mačková, R. Pohl and M. Hocek, *ChemBioChem*, 2014, **15**, 2306–2312.

23. M. Janoušková, Z. Vaníková, F. Nici, S. Boháčová, D. Vítovská, H. Šanderová, M. Hocek and L. Krásný, *Chemical Communications*, 2017, **53**, 13253–13255.

24. L. Sojka, T. Kouba, I. Barvík, H. Šanderová, Z. Maderová, J. Jonák and L. Krásný, *Nucleic Acids Res.*, 2011, **39**, 4598–4611.

25. CrysAlisPro, Oxford Diffraction, 2002.

26. A. Altomare, G. Cascarano, C. Giacovazzo, A. Guagliardi, M. C. Burla, G. Polidori and M. Camalli, *Journal of Applied Crystallography*, 1994, **27**, 435–435.

27. P. W. Betteridge, J. R. Carruthers, R. I. Cooper, K. Prout and D. J. Watkin, *Journal of Applied Crystallography*, 2003, **36**, 1487–1487.
